# Direct electrophilic and radical isoperfluoropropylation with *i*-C_3_F_7_-Iodine(III) reagent (PFPI reagent)

**DOI:** 10.1038/s42004-023-00986-3

**Published:** 2023-08-24

**Authors:** Yaxing Wu, Yunchen Jiang, Fei Wang, Bin Wang, Chao Chen

**Affiliations:** 1 Key Laboratory of Bioorganic Phosphorus Chemistry & Chemical Biology (Ministry of Education), Department of Chemistry, Tsinghua University, Beijing, 100084 China; 2 State Key Laboratory of Elemento-Organic Chemistry, Nankai University, Tianjin, 300071 China

**Keywords:** Synthetic chemistry methodology, Synthetic chemistry methodology, Photocatalysis, Catalyst synthesis

## Abstract

The isoperfluoropropyl group (*i*-C_3_F_7_) is an emerging motif in pharmaceuticals, agrichemicals and functional materials. However, isoperfluoropropylated compounds remain largely underexplored, presumably due to the lack of efficient access to these compounds. Herein, we disclose the practical and efficient isoperfluoropropylation of aromatic C-H bonds through the invention of a hypervalent-iodine-based reagent-PFPI reagent, that proceeds via a Ag-X coupling process. The activation of the PFPI reagent without any catalysts or additives was demonstrated in the synthesis of isoperfluoropropylated electron-rich heterocycles, while its activity under photoredox catalysis was shown in the synthesis of isoperfluoropropylated non-activated arenes. Detailed mechanistic experiments and DFT calculations revealed a SET-induced concerted mechanistic pathway in the photoredox reactions. In addition, the unique conformation of *i*-C_3_F_7_ in products, that involved intramolecular hydrogen bond was investigated by X-ray single-crystal diffraction and variable-temperature NMR experiments.

## Abstract (web summary)

The isoperfluoropropyl group (*i*-C_3_F_7_) is an analogue of the trifluoromethyl group (CF_3_) featuring a stronger electron-withdrawing effect and more steric hindrance, giving it a unique role in synthetic chemistry, however, direct isoperfluoropropylation remains challenging. Here, the authors develop a highly reactive electrophilic hypervalent-iodine based *i*-C_3_F_7_ reagent and demonstrate its use for the efficient isoperfluoropropylation of aromatic C–H bonds.

## Introduction

**Fig. 1:**
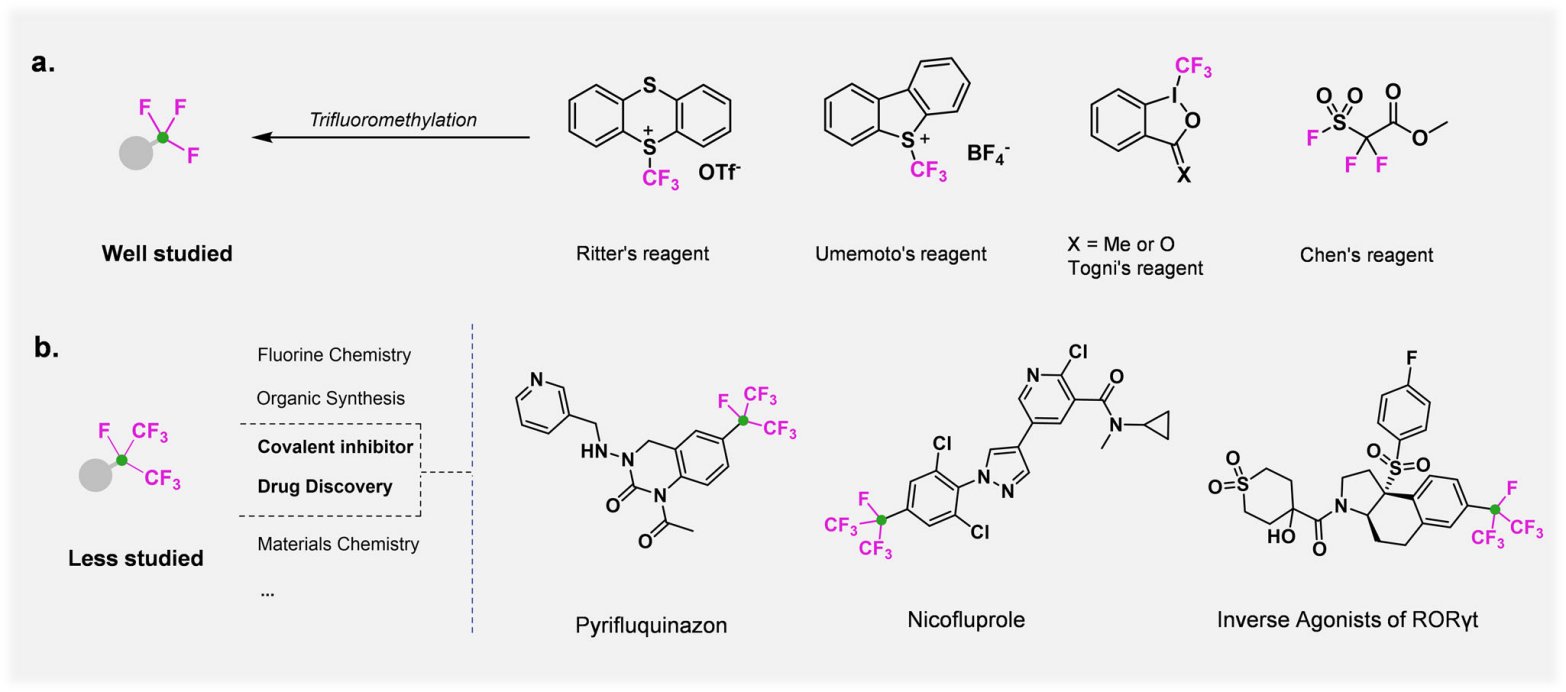
Trifluoromethylation reagents and selected bio-active molecules bearing an isoperfluoropropyl structural motif. **a** Classical trifluoromethylation reagent. **b** Selected bio-active molecules bearing an isoperfluoropropyl structural motif.

As a result of the unique metabolic stability, cell permeability, and lipophicity observed for fluoroalkyl compounds^1–7^, the efficient introduction of polyfluoroalkyl motifs in commonly used building blocks has attracted intense attention^8–11^. As the earliest widely studied fluoroalkyl group, trifluoromethyl group (CF_3_) is now widely accepted as one of the privileged functional groups for modern medicinal chemists. Numerous efforts from research groups around the world have been devoted to this field, and a plethora of sophisticated trifluoromethylating methods have been reported in the past two decades^12–14^. Depending on the desired transformation, different reagents for nucleophilic, radical, or electrophilic trifluoromethylation are used (Fig. 1a). As a large analog of the CF_3_, the isoperfluoropropyl group (*i*-C_3_F_7_), featuring a stronger electron-withdrawing effect and more steric hindrance, is normally considered as a “super” CF_3_ group and plays a unique role in bioactive compounds, functional materials, and organocatalysts (Fig. 1b)^15–21^. For instance, pyrifluquinazon and nicofluprole are commercially available insecticides with excellent activity against broad-spectrum pests. Meanwhile, *i*-C_3_F_7_ can be applied to the development of inverse agonists with better selectivity for identification of biologic-like in vivo efficacy^17^, and it is reasonable to think that *i*-C_3_F_7_ would have potential application for the design and preparation of candidate pharmaceuticals. Nevertheless, unlike introducting CF_3_ into organic molecules, which has diversified over the years, the primary methods of incorporating *i*-C_3_F_7_ have been limited to a few isoperfluoropropylating reagents, such as isoperfluoropropyl iodide (*i*-C_3_F_7_I) and isoperfluoropropyl metal (*i*-C_3_F_7_M) reagents.

**Fig. 2:**
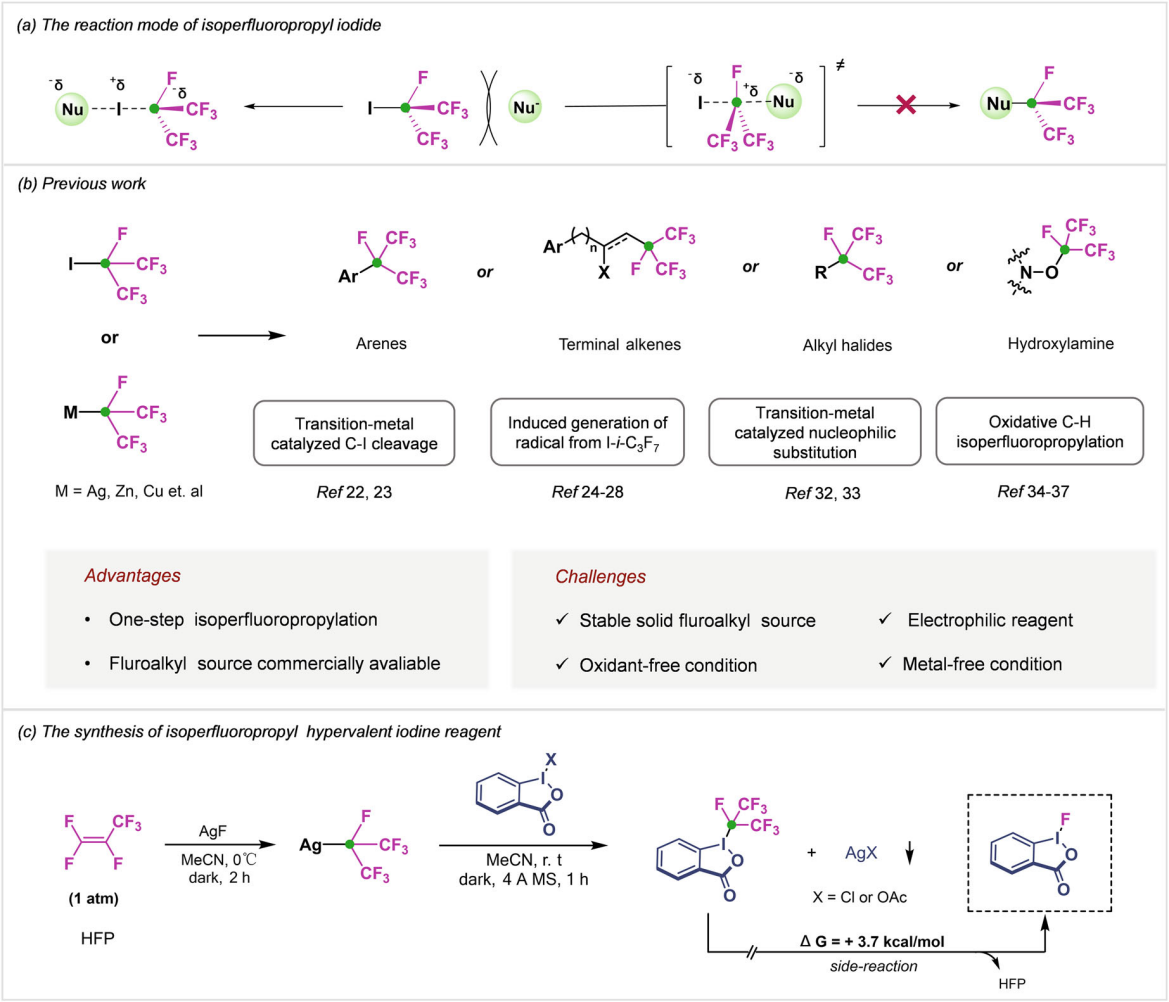
The development of a strategy for isoperfluoropropylation. **a** The reaction mode of isoperfluoropropyl iodide. **b** The synthetic methods of isoperfluoropropyl-containing compounds. **c** This chemistry: the design and synthesis of isoperfluoropropyl hypervalent iodine reagent.

Generally, *i*-C_3_F_7_I is the most convenient and commercially available *i*-C_3_F_7_ source^22–28^. Unlike their analogous alkyl halide, the formation of a Nu-*i*-C_3_F_7_ bond *via* direct S_N_2 type displacement is unfavorable due to strong electron repulsion and steric hindrance between fluoroalkyl and incoming nucleophiles, the formation of an energetically adverse *i*-C_3_F_7_ carbocation transition state structure (Fig. 2a). *i*-C_3_F_7_I is easier to be initiated than the linear isomers by heating or a large number of reducing agents, generating *i*-C_3_F_7_ radical for further conversions^24–28^. Recently, a series of metal-catalyzed isoperfluoropropylation using *i*-C_3_F_7_I as *i*-C_3_F_7_ source have also been reported (Fig. 2b)^22,23^. Nevertheless, most of these methods suffered from some limitations such as the need for harsh reaction conditions, and the use of metal catalysts and iodine active species to interfere with the isoperfluoropropylation process. Several attempts for synthesizing nucleophilic *i*-C_3_F_7_M reagents were reported (Fig. 2b). For instance, Burton and co-works realized the synthesis of Cu-*i*-C_3_F_7_ from *i*-C_3_F_7_I with the assistance of cadmium^29^. In 1968, Burnard et al. found that hexafluoropropylene (HFP) reacted with silver fluoride to form an isoperfluoropropyl carbanion (Ag-*i*-C_3_F_7_)^30^. Although these organometallic reagents were unstable and sensitive to moisture, a series of metal-catalyzed nucleophilic isoperfluoropropylation were developed^31–33^. Very recently, Qing’s and Wu’s group reported an efficient oxidative isoperfluoropropylation of unactivated alkenes, arenes, and arylboronic acid with HFP-derived Ag-*i*-C_3_F_7_, and stoichiometric oxidants and metal were necessary for this strategy^34–37^.

Evidently, an active electrophilic reagent that behaves as a convenient source of the *i*-C_3_F_7_ and is amenable to direct isoperfluoropropylation of a broad range of substrates under mild conditions, while avoiding the use of prefunctionalized substrate or strong oxidants, remains synthetically desirable and challenging, and essential to facilitate the development of new classes of fluoroalkyl compounds with unexplored properties. Due to the strong electron-withdrawing effect of *i*-C_3_F_7_, the synthesis of stable electrophilic reagents is extremely difficult in theory. Hypervalent iodine benziodoxolone has emerged as a powerful tool for oxidative functional group installation^38–40^, and is generally considered environmentally benign and widely available alternatives to traditional transition-metal-based reagents. In view of our group’s interest in hypervalent iodine and fluorine chemistry^41–45^

, we envisaged that the merger of the *i*-C_3_F_7_ with a hypervalent iodine scaffold would provide a platform for the development of such a PFPI reagent (Fig. 2c).

## Results and discussion

### Synthesis of *i*-C_3_F_7_-iodine(III) reagent

**Fig. 3:**
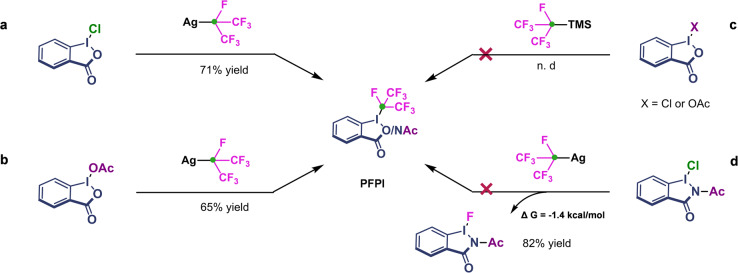
The synthesis development of PFPI reagent. **a** “Ag-Cl” coupling. **b** “Ag-OAc” coupling. **c** “TMS-X” coupling (failed). **d** “Ag-Cl” coupling to synthesize monofluoroiodane(III).

The *i*-C_3_F_7_-iodine(III) reagent (PFPI) was synthesized in 71% yield by means of a two-step procedure (Fig. 2c). First, 2-iodobenzoic acid was treated with trichloroisocyanuric acid (TCICA) to give a hypervalent chloroiodine(III) intermediate. Inspired by the synthesis of Togni-reagent^46,47^, all our initial attempts to obtain the desired *i*-C_3_F_7_-iodane(III) by means of ligand-exchange reactions of the hypervalent iodine(III) intermediate with common TMS-*i*-C_3_F_7_ under various conditions failed (Fig. 3c). However, the treatment of the chloroiodine(III) intermediate with Ag-*i*-C_3_F_7_ based on our proposed Ag-X coupling strategy generated desired PFPI compound in good yield (Fig. 3a), which was characterized by ^1^H, ^13^C, and ^19^F NMR spectroscopy. The product can be purified by washing with diethyl ether to deliver an easy to handle, free-flowing off-white powder that can be stored under anhydrous conditions in the absence of light without significant decomposition for at least a month. In addition, other types of iodine(III) intermediate are also tested, and the *i*-C_3_F_7_-iodine(III) reagent can be obtained in 65% yield by ligand-exchange of the acetoxyiodine(III) intermediate (Fig. 3b). Interestingly, when we changed the substrate to chloroiodane(III) with amide skeleton in this reaction system, the quantitative decomposition of *i*-C_3_F_7_− iodine(III) was occurred with a Gibbs free energy equal to −1.4 kcal mol^−1^ (DFT calculated value, See Supplementary Data 3) and monofluoroiodane(III) was obtained as the main product (Fig. 3d), which revealed that the stability of the reagent is significantly related to the skeleton structure of iodine(III).

### Metal-free electrophilic isoperfluoropropylation of electron-rich heterocycles with PFPI

**Fig. 4:**
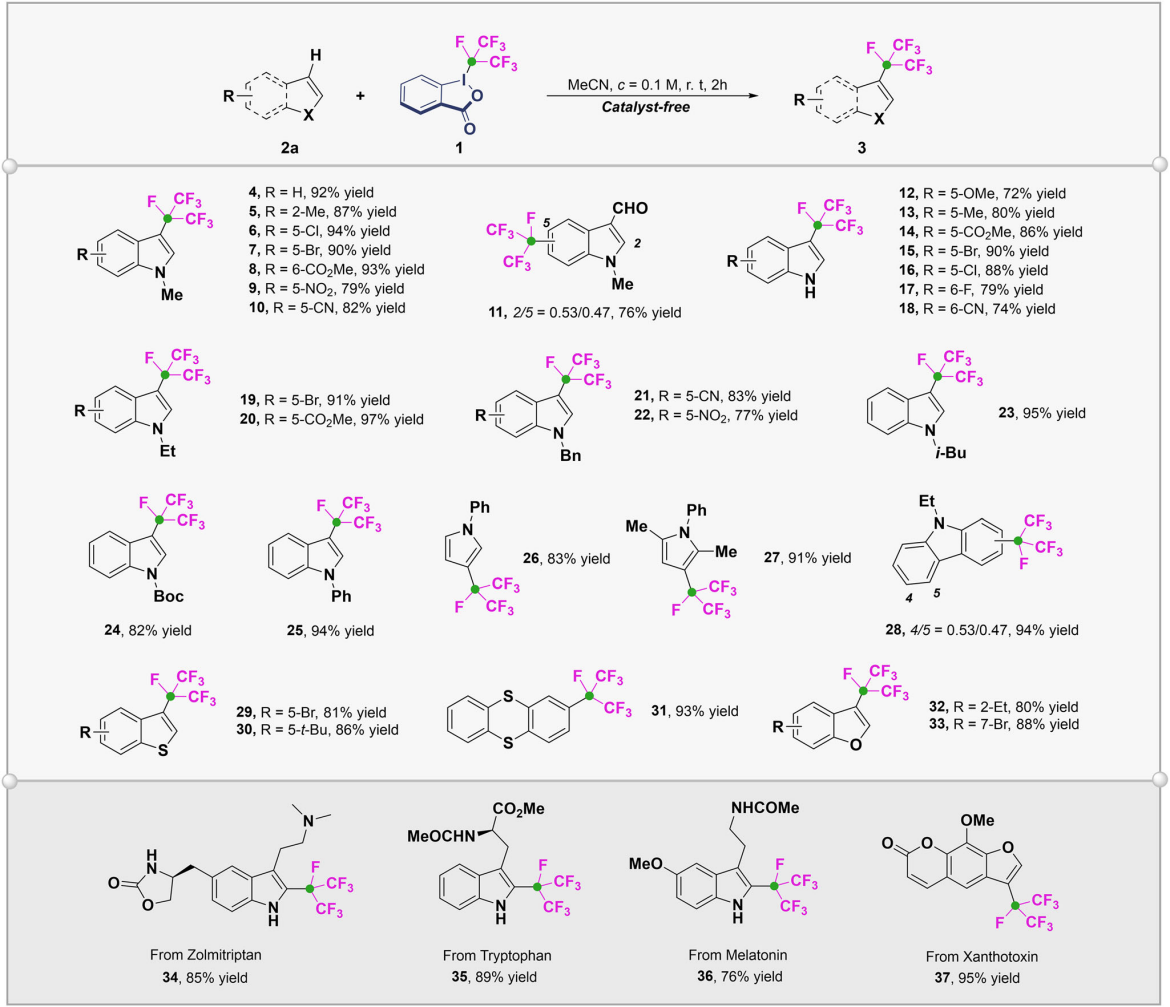
Substrate scope of the metal-free isoperfluoropropylation of electron-rich heterocycles. Reaction conditions: **2a** (0.2 mmol, 1.0 equiv), **1** (0.4 mmol, 2.0 equiv), dry MeCN (2 mL), rt, 2 h, under a N_2_ atmosphere. Isolated yields are reported. The isomer ratios were determined *via* ^19^F NMR analysis and are shown.

It was found that the PFPI regent had unique reactivity compared with other fluoroalkyl-iodine(III) such as Togni-reagent in our early activity exploration. We proposed that the high reactivity of PFPI reagent comes from the strong electron-withdrawing effect of *i*-C_3_F_7_, which makes it easy to be reduced to radical anion through a single electron transfer process. At the same time, the large steric hindrance causes significant elongation of the C(*i*-C_3_F_7_)-I bond (2.282 Å, DFT calculated value), resulting in homolysis and release of highly active *i*-C_3_F_7_ radical. Motivated by these preliminary indications, we developed the isoperfluoropropylation of electron-rich heterocycle compounds and commenced with indoles, which were used as valuable building blocks in the synthesis of natural products and bioactive compounds^48^. We chose the commercially available *N*-methyl indole as a model substrate, and after screening reaction conditions (see SI, Table S1 and S2) we were pleased to observe that the reaction with **2** proceeded readily at room temperature in the absence of any catalyst or exogenous base, delivering the desired product **4** in excellent yield. As comparision, the use of Togni-reagents for similar reactions usually required Lewis acids such as Zn(NTf )_2_ for activation^49,50^. Subsequently, the scope of this direct C-H isoperfluoropropylation was explored (Fig. 4). Most of the electron-rich heterocycles could be transformed to the isoperfluoropropylated compounds **3** in good to excellent yields. In general, the isoperfluoropropylation took place selectively at the C-3 position of indoles and *N*-alkyl (-Me, -Et, -Bn, -*i*-Bu) indoles (**4-11,** **19-23**) showed better reaction efficiency than those *N*-H indoles (**12-18**). With regard to the functional group tolerance, a diverse array of functional groups such as aldehyde (**11**), ether (**12**), ester (**8**, **14**, **20**), cyano (**10**, **18**, **21**), nitro (**9**, **22**), and aryl halides (**6**, **7**, **15-17**, **19**) were compatible with the reaction conditions. For 3-CHO indole, a mixture of 2-and 5-substituted products was obtained. In addition, pyrrole (**26**, **27**), carbazole (**28**), thiophene (**29**, **30**), thianthrene (**31**), and furan (**32**, **33**) could all be smoothly isoperfluoropropylated with *i*-C_3_F_7_-iodine(III) reagent. A plethora of indoles derived from natural products, such as tryptophan (**35**), melatonin (**36**), and xanthotoxin (**37**), as well as the drug molecules zolmitriptan (**34**), successfully underwent the direct isoperfluoropropylation, which proved that complex architectures were well tolerated.

### Screening of reaction conditions for photocatalytic isoperfluoropropylation

**Fig. 5:**
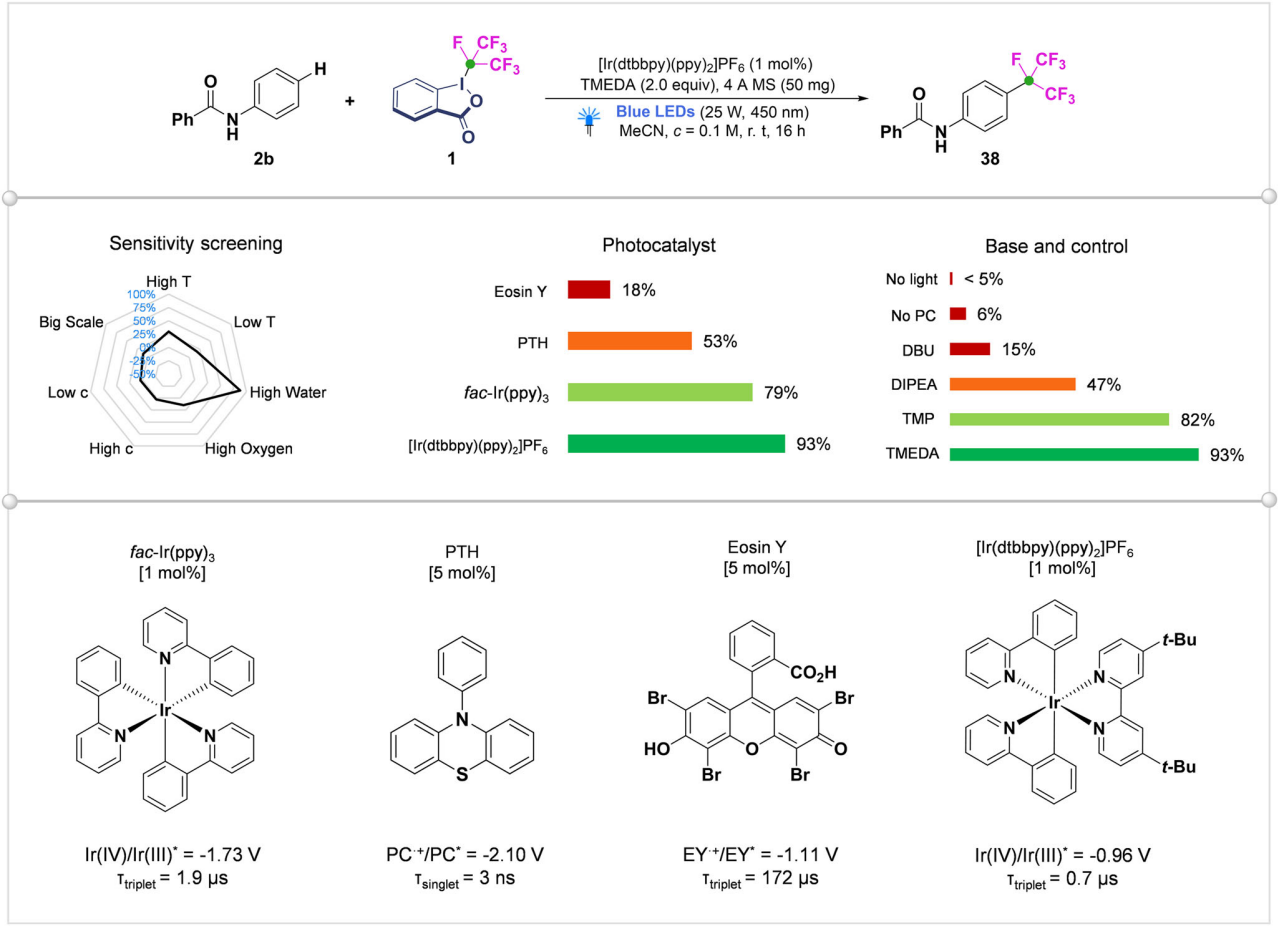
The optimization of a photocatalytic isoperfluoropropylation and sensitivity screening. For experimental details, see SI (Pages S35–39).

Encouraged by the successful metal-free isoperfluoropropylation of electron-rich heterocycles, we next attempted to examine the C-H isoperfluoropropylation of various common arenes. Due to the relatively weak activity of non-activated arenes, their reaction with electrophilic reagents required relatively harsh reaction conditions. In 2019, Bao’ group developed an efficient Mo-catalyzed highly site-selective perfluoroalkylation of anilide derivatives by use of fluoroalkyl iodide, but these reactions need to be carried out at a high temperature and in the atmosphere of CO^26^. In our method, single electron transfer (SET) from an photoredox catalyst to the *i*-C_3_F_7_-iodine(III) is followed by C − I cleavage to deliver *i*-C_3_F_7_ radical that displayed unique reactivity profiles. To find the most suitable catalytic conditions for the isoperfluoropropylation of anilides, a plethora of metal and organic photoredox catalyst, such as *fac*-Ir(ppy)_3_, [Ir(dtbbpy)(ppy)_2_]PF_6_, PTH and Eosin Y, were tested (Fig. 5). Having found the most suitable conditions for the generation of the *i*-C_3_F_7_ radical precursor from *i*-C_3_F_7_-iodine(III) **1** using [Ir(dtbbpy)(ppy)_2_]PF_6_ with appropriate redox potential (Ir^IV^/Ir^III*^ = −0.96 V *vs* SCE) as a photoredox catalyst (1 mol%), we optimized the C-H isoperfluoropropylation of *i*-C_3_F_7_ radical with benzanilide (**2b**) with respect to different reaction parameters (See SI, Table S3–S5). A condition-based sensitivity-screening approach revealed that the process is rather insensitive towards variations in concentration, temperature, oxygen and scale-up (See SI, Table S6–S10). Simple nitrogen sparging afforded comparable yields with a more rigorous 4 Å MS procedure (low water), whereas running the reaction in the presence of stoichiometric water completely suppressed the productive process (high water).

### Experimental and computational mechanistic studies

**Fig. 6:**
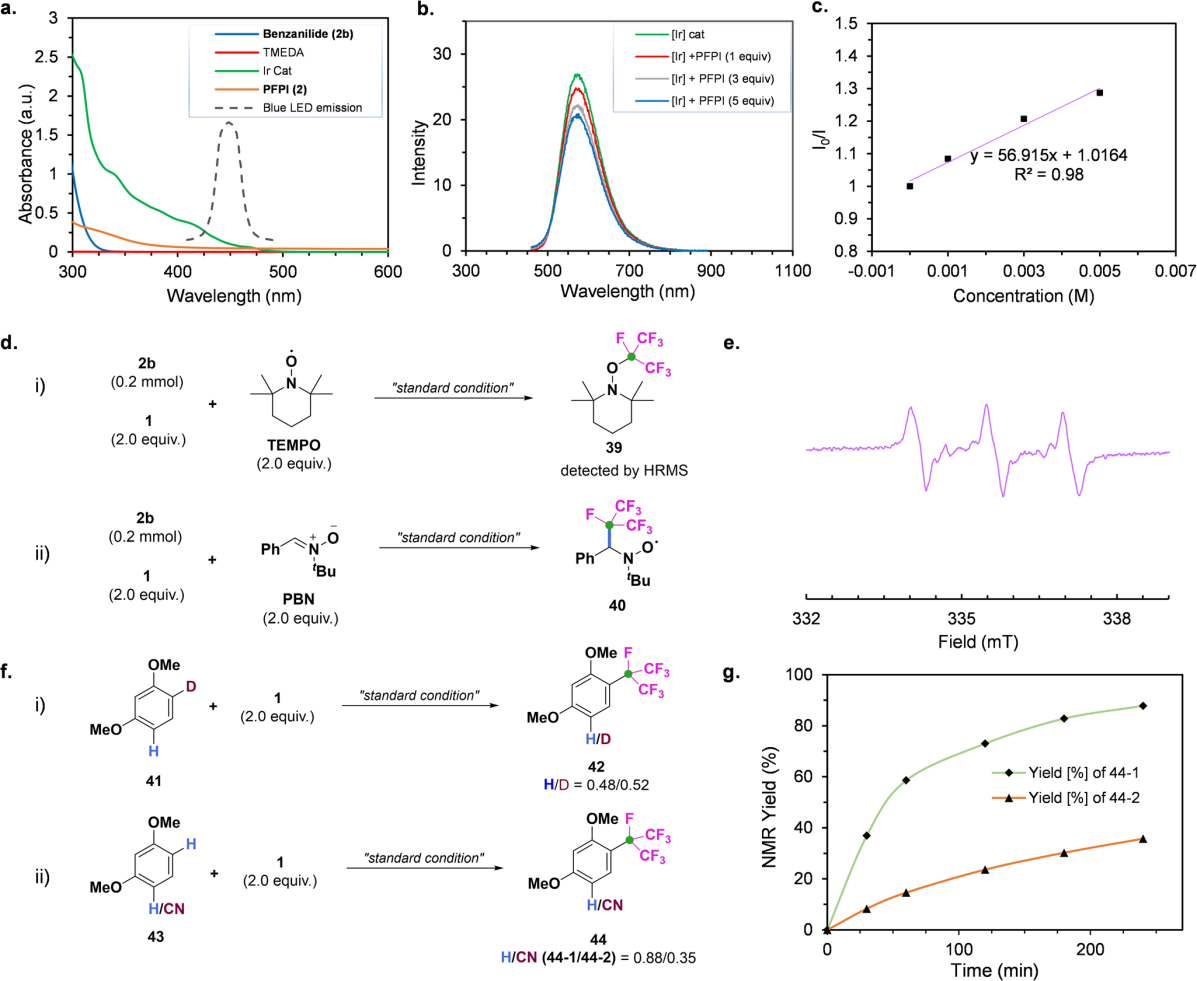
Mechanistic studies. **a** Ultraviolet-visible absorption experiments in MeCN. **b**, **c** Stern-Volmer luminescence quenching of [Ir(dtbbpy)(ppy)_2_]PF_6_ by iodine(III) reagent **2**. **d** Radical trapping experiments. **e** EPR spectra of **40**. **f** Isotopic (H/D) and substrates competition experiment. **g** A reaction rate measurement.

Firstly, UV-visible analysis showed that only the iridium catalyst substantially absorbed at blue light (λ ≈ 450 nm) (Fig. 6a). We also conducted Stern-Volmer luminescence quenching experiments and found that the excited-state Ir(dtbbpy)(ppy)_2_^*^ complex was efficiently quenched by PFPI **1** (Figs. 6b, 6c). A radical trap experiment by using 2, 2, 6, 6-tetramethylpiperidinyl-1-oxide (TEMPO) as a probe and electron paramagnetic resonance (EPR) studies of the reaction of **2b** and **1** with spin-trapping agent phenyl *tert*-butyl nitrone (PBN) demonstrated that an isoperfluoropropyl radical was involved in the reaction (Figs. 6d, 6e). Isotopic (H/D) competition experiments show that the C-H bond cleavage process was not a rate-determining step (Fig. 6f). Finally, subjecting a mixture of arenes, **43-1** and **43-2** bearing an electron-withdrawing group (−CN) to standard conditions produced **44-1** as the major product (88%) along with minor product **44-2** (35%), supporting an electrophilic process to the arene in the product-determining step (Fig. 6g).

**Fig. 7:**
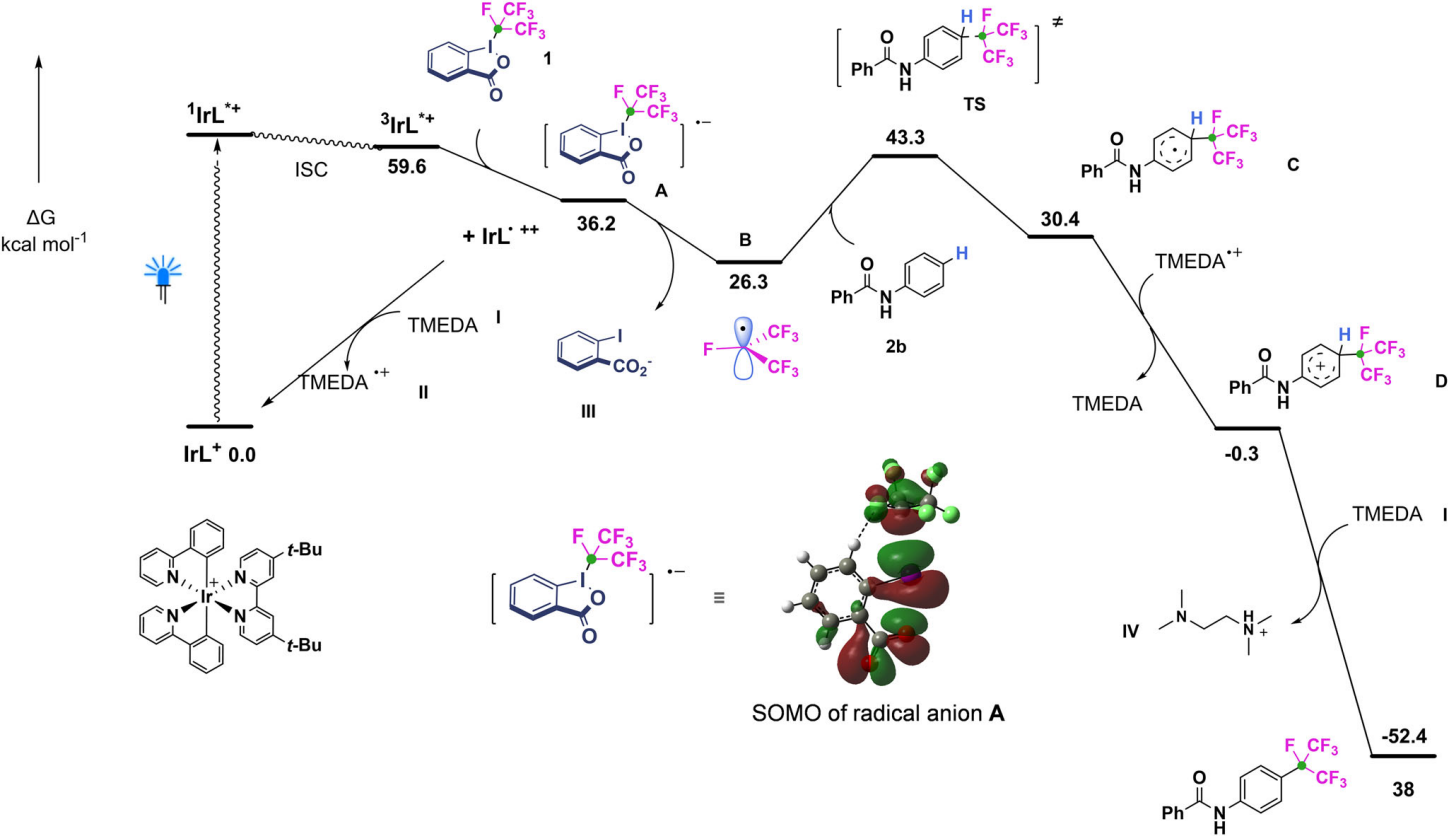
DFT-computed energy profile. DFT-computed Gibbs free energy profile (in kcal mol^−1^, at 298 K) in MeCN solution for photocatalyzed isoperfluoropropylation of benzanilide (**2b**) with PFPI reagent **2** at the TPSS-D3/def2-SVP + IEFPCM//TD-B3LYP/def2-TZVP + SMD level of theory. See Supplementary Data 3.

For a better understanding of how the photocatalyst efficiently mediated the catalytic anilide isoperfluoropropylation, the energy profiles of the reaction were evaluated by density functional theory (DFT) calculations, and the results were summarized in Fig. 7. Consistent with Stern-Volmer fluorescence quenching experiments, our DFT calculations show that after blue light excitation of the [Ir(dtbbpy)(ppy)_2_]^+^ cation (IrL^+^), SET process from IrL^+^ in its lowest triplet excited state (^3^IrL^*+^) to **1** is −23.4 kcal mol^−1^ exergonic to form the oxidized radical cation IrL^·++^ along with the reduced radical anion **A**, thus favoring the oxidative quenching of ^3^IrL^*+^. Further SET oxidation of TMEDA with the oxidized radical cation IrL^·++^ regenerates the photo-catalyst cation IrL^+^ in the ground state along. Subsequently, the cleavage of the C-I bond proceeds almost without barrier and 2-iodobenzoate anion releases from the photo-generated **A** is −9.9 kcal mol^−1^ exergonic to form the *i*-C_3_F_7_ radical **B**. Once formed, the reactive radical **B** can be easily added to the *para*-site C-H bond of **1b** to form a new C-C bond, which is 4.1 kcal mol^−1^ endergonic over a low barrier of 17.0 kcal mol^−1^ (*via* **TS**) to form the transient radical **C**. The final stage of the reaction involves the SET oxidation of intermediate **C** and deprotonation of aryl cation **D** to generate target product **39**, with a Gibbs free energy of the two processes equal to −30.7 kcal mol^−1^ and −52.1 kcal mol^−1^ respectively.

### Substrate scope with respect to photocatalytic isoperfluoropropylation of non-activated arenes

**Fig. 8:**
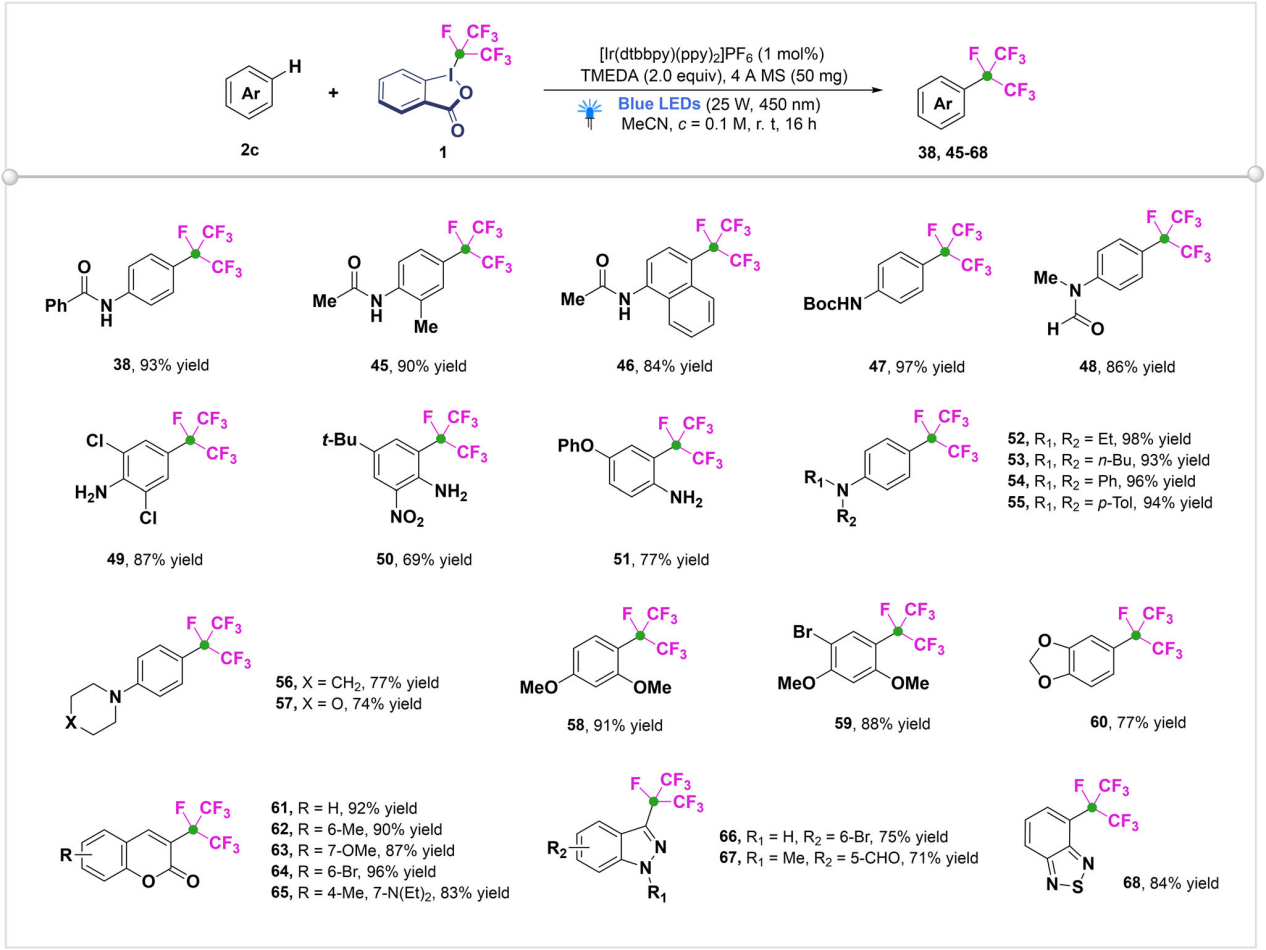
Substrate scope of the photocatalytic isoperfluoropropylation of non-activated arenes. Reaction conditions: **2c** (0.2 mmol, 1.0 equiv), **1** (0.4 mmol, 2.0 equiv), [Ir(dtbbpy)(ppy)_2_]PF_6_ (1.0 mol%), TMEDA (0.4 mmol, 2.0 equiv), 4 Å MS (50 mg), dry MeCN (2 mL), rt, 16 h, under a N_2_ atmosphere and 25 W Blue LEDs. Isolated yields are reported.

With the viable reaction conditions in hand, the substrate scope of this visible-light-induced isoperfluoropropylation was examined (Fig. 8). Gratifyingly, the reaction of **1** with a variety of anilides afforded the corresponding isoperfluoropropylated products (**38**, **45**-**48**) in moderate to excellent yields with highly *para*-site selectivity. Primary aniline derivates bearing different functional groups, such as choro (**49**), nitro (**50**), alkyl (**50**), PhO-(**51**), and *N*-substituted anilines were also applicable to the reaction (**52**-**57**). Polysubstituted aryl ethers and coumarins derivates as substrates worked smoothly to furnish corresponding products **58**-**60** and **61**-**65**, respectively with good yields and functional group compatibility. Coumarins were clinically used as oral anticoagulants and the introduction of isoperfluoropropyl would significantly improve the liposolubility of molecules and thus promote drug metabolism. Noteworthy, indazole (**66**, **67**) and benzo-[2,1,3]-thiadiazole (**68**) functionalities could be compatible under the photoredox condition, which couldn’t be isoperfluoropropylation under the previous catalyst free conditions.

### The synthetic applications and X-ray single-crystal diffraction analysis of products

**Fig. 9:**
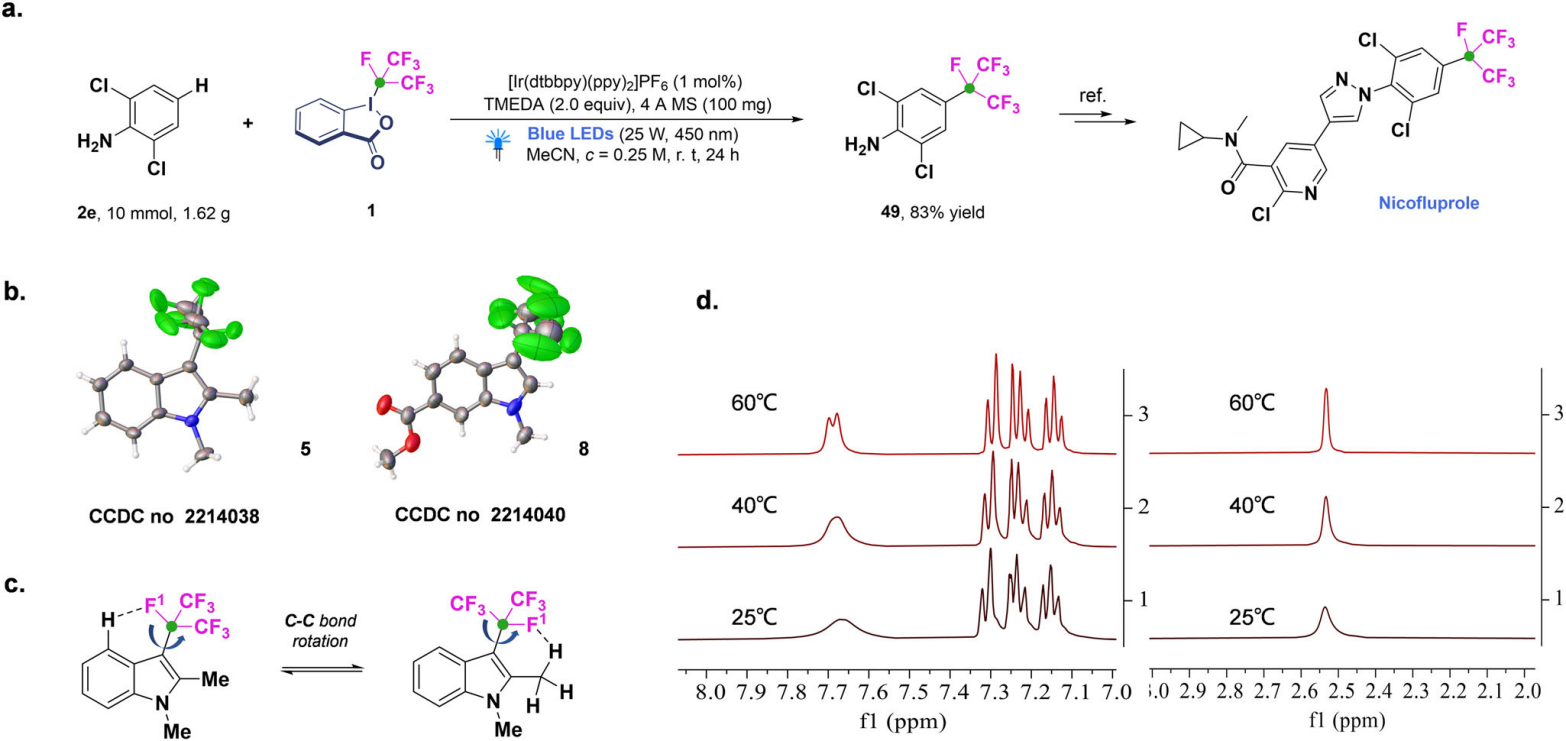
Synthetic applications and conformational analysis. **a** Gram scale synthesis of **49**. **b** X-ray single-crystal diffraction analysis of **5** and **8**. **c**The conformation of **5**. **d** Variable-temperature 1H NMR of **5**.

Our method could offer straightforward access to the 4-perfluoroisopropyl-2,6-dichloroaniline fragment on gram-scale, a key intermediate structure in total synthesis sequences towards broad-spectrum insecticide nicofluprole (Fig. 9a)^51^. The results confirm that the present protocol can serve as an efficient and practical strategy to obtain products. In 2021, Gilmour analyzed the conformation of a heptafluoroisopropylated arene and revealed the preference of the benzylic C(sp^3^)-F bond to be co-planar with the aryl ring^52^. Herein, the X-ray single-crystal diffraction analysis (See SI, Table S11-S20; Supplementary Data 1 and Data 2) of compounds **5** and **8** confirmed the connectivity of the molecules (Fig. 9b), and variable-temperature (VT) ^1^H NMR spectrum of compound **5** clearly depicts that *i*-C_3_F_7_-substituted indoles possess interesting conformation, which might find useful applications in medicinal chemistry (Fig. 9c). The space orientation of the central-site fluorine atom (F^1^) of the isoperfluoropropyl is affected by the hydrogen atom on the adjacent group. In the solution state at room temperature, the F^1^ of **5** showed two orientations and can freely convert between each other, which is reflected in the ^1^H NMR spectrum that the hydrogen atom of 4-site C(sp^2^)-H and 2-site -CH_3_ presented irregular wide peak signals, and the peak pattern becomes sharper after increasing the temperature (Fig. 9d). The result can also be confirmed by two sets of F^1^ signals in the VT ^19^F NMR (See SI, Fig. S11). The striking molecular conformation is probably due to the formation of a six membered ring based on an intramolecular hydrogen bond between the F^1^ on isoperfluoropropyl and the hydrogen on the adjacent group or carbon atom.

## Conclusions

In summary, we have described the development of a hypervalent iodine-based PFPI reagent, which is easily accessible from commercial starting materials in two steps. The reagent can be engaged in metal-free electrophilic and photoredox radical isoperfluoropropylation. The mechanistic experiments and DFT calculations suggested a SET-induced concerted mechanistic pathway in photoredox reactions. Further studies of this air-stable and highly reactive solid reagent are underway in our laboratory.

## Methods

### General procedure for the synthesis of PFPI reagent

In a nitrogen-filled glove box, an oven-dried crimp cap vessel with Teflon-coated stirrer bar was charged with silver fluoride (0.64 g, 5.0 mmol, 1.0 equiv.) and was brought under an atmosphere of dry nitrogen. To this vessel, anhydrous acetonitrile (20 mL) and hexafluoropropylene (1 atm, balloon and adequate) were added, and the mixture was stirred at ice-water bath in the dark until silver fluoride precipitate dissolved completely. Then this solution was added to another oven-dried vessel, which filling with 1-chloro-1,2-benziodoxol-3-(1*H*)-one (1.44 g, 5.1 mmol, 1.02 equiv.) and 4 Å molecular sieves (100 mg). The reaction mixture was stirred at ambient temperature in the dark for 1 h. The reaction mixture was filtered over a sintered-glass funnel with a tightly packed pad of Celite (0.5 cm thick) under dry nitrogen atmosphere, and the filter cake was rinsed with additional anhydrous acetonitrile (5–10 mL). The solvent was evaporated under reduced pressure and crude solid product washed with anhydrous Et_2_O (10–20 mL). Then, the product was obtained by anhydrous DCM leaching in ultrasonic generator under dry nitrogen atmosphere and vacuum evaporated of solvent. The solid was dried for 1 h under high vacuum to give 1-isoperfluoropropyl-1,2-benziodoxol-3-(1*H*)-one as off white solid (1.48 g, 71% yield).

### General procedure for the metal-free isoperfluoropropylation of electron-rich heterocycles

In a 25 mL screw-cap vial equipped with a magnetic stirring bar, electron-rich heterocycles (0.20 mmol, 1.0 equiv.), PFPI reagent (166.4 mg, 0.40 mmol, 2.0 equiv) and additives were dissolved in MeCN (2 mL). The reaction was stirred for 2 h at room temperature under an atmosphere of dry nitrogen. The reaction was quenched with 10 mL 5% NaHCO_3_ aqueous solution, then extracted with DCM (3 × 10 mL). The combined organic phase was dried with anhydrous Na_2_SO_4_ and concentrated under a vacuum. The residue was further purified by column chromatography (PE-EtOAc) to afford corresponding isoperfluoropropylated products (See Supplementary Methods).

### General procedure for the photocatalytic isoperfluoropropylation of non-activated arenes

Under an ambient atmosphere, in a 25 mL screw-cap vial equipped with a magnetic stirring bar, arenes (0.20 mmol, 1.0 equiv.), PFPI reagent (166.4 mg, 0.40 mmol, 2.0 equiv), [Ir(dtbbpy)(ppy)_2_]PF_6_ (1.8 mg, 1 mol%), were dissolved in dry MeCN (2.0 mL). Subsequently, 4 Å molecular sieves (50 mg) and TMEDA (59.8 μL, 0.40 mmol, 2.0 equiv) was added into the vial. The vial was sealed and the reaction was irradiated at 450 nm for 16 h, the temperature of the reaction systems was controlled within 25–35 °C by using the drum fan. After this, the reaction was quenched with 10 mL 5% NaHCO_3_ aqueous solution, then extracted with DCM (3 × 10 mL). The combined organic phase was dried with anhydrous Na_2_SO_4_ and concentrated under a vacuum. The residue was further purified by column chromatography (PE-EtOAc) to afford corresponding isoperfluoropropylated products. (See Supplementary Methods).

### Supplementary information

Supporting Information

Description of Additional Supplementary Files

Supplementary Data 1

Supplementary Data 2

Supplementary Data 3

Supplementary Data 4

## Data Availability

The authors declare that the main data supporting the findings of this study, including experimental procedures and compound characterization, are available within the article and its Supplementary Information files, or from the corresponding author upon request. For supplementary figures, see SI, Figure S1-S16. For X-Ray crystallography of compound 5 and compound 8, see Supplementary Data 1 and Data 2. For Cartesian coordinates of the structures, see Supplementary Data 3. For NMR spectra, see Supplementary Data 4.
