# Supplementary material for: Direct electrophilic and radical isoperfluoropropylation with i-C3F7-Iodine(III) reagent (PFPI reagent)
Source: Commun Chem. 2023 Aug 24;6:177. doi: 10.1038/s42004-023-00986-3 (PMC10449889; doi:10.1038/s42004-023-00986-3)
Supplement: Supplementary file 1 — Supporting Information [file 42004_2023_986_MOESM1_ESM.pdf]

SUPPORTING INFORMATION

Direct Electrophilic and Radical Isoperfluoropropylation with *i*-C_3F_7-Iodine(III) Reagent (PFPI Reagent)

Yaxing Wu[a], Yunchen Jiang[a], Fei Wang[a], Bin Wang[a], Chao Chen* [a, b]

a. Key Laboratory of Bioorganic Phosphorus Chemistry & Chemical Biology (Ministry of Education), Department of Chemistry, Tsinghua University, Beijing 100084, China.

b. State Key Laboratory of Elemento-Organic Chemistry, Nankai University, Tianjin 300071, China

* E-mail: chenchao01@mails.tsinghua.edu.cn. Tel: +86-10-62773684.

TABLE OF CONTENTS

TABLE OF CONTENTS S2

MATERIALS AND METHODS.. S3

EXPERIMENTAL DATA.. S5

The *i*-C_3F_7-iodine(III) reagent synthesis.. S5

Direct isoperfluoropropylation of electron-rich heterocycles.................................. S11

Optimization of reaction conditions.. S11

Substrate scope.. S12

The photocatalytic isoperfluoropropylation of non-activated arenes......................... S35

Optimization of reaction conditions.. S35

Sensitivity screening.. S37

Mechanistic investigations.. S40

DFT calculations... S48

Substrate scope.. S76

Variable-temperature ^{1}H NMR and ^{19}F NMR experiments.................................. S93

X-Ray Crystal Structures... S95

NMR spectra... S112

REFERENCES.. S306

MATERIALS AND METHODS

Supplementary Methods

All reactions were carried out under ambient atmosphere unless otherwise stated. Concentration under reduced pressure was performed by rotary evaporation at 25-45 °C at an appropriate pressure. Purified compounds were further dried under vacuum (10^{-4}-10^{-1} KPa). Isolated yields refer to purified and spectroscopically pure compounds or mixtures of constitutional isomers. All air- and moisture-sensitive manipulations were performed using standard Schlenk- or glove-box techniques under an atmosphere of argon or dinitrogen.

Starting materials

All substrates and materials were used as received from commercial suppliers, or prepared according to published procedures, respectively, unless otherwise stated. AgF was purchased from Energy-Chemical, and stored in an anhydrous brown bottle. Various Ir and Rh photocatalysts was purchased from Sigma-Aldrich, Bidepharm and Energy-Chemical.

Solvents

Dry THF, DCM and DME were purchased from Innochem (water content < 50 ppm). Dry MeCN and DMF were purchased from Energy-Chemical (water content < 30 ppm). Dry ether was obtained by post-treatment with 4 Å molecular sieve, which activated at 300 °C in muffle furnace. All deuterated solvents were purchased from Sigma-Aldrich. EtOAc and petroleum ether (boiling range 60-90 °C) was purchased from Tansoole.

Spectroscopy and instruments

NMR spectra were recorded on a JEOL AL-400MHz spectrometer operating at 400 MHz, 101 MHz, and 376 MHz, for 1H, 13C, and 19F acquisitions. Chemical shifts are reported in ppm with the solvent residual peak as the internal standard. For ^{1}H NMR: chloroform-*d*, δ 7.26; dimethyl sulfoxide-d_6, δ 2.50. For ^{13}C NMR: chloroform-*d*, δ 77.16; dimethyl sulfoxide-d_6, δ 39.52. ^{19}F NMR spectra were referenced using a unified chemical shift scale based on the ^{1}H NMR resonance of tetramethylsilane (1% (v/v) solution in the respective solvent). Data is reported as follows: s = singlet, d = doublet, t = triplet, q = quartet, m = multiplet, br = broad; coupling constants in Hz; integration. HRMS spectra were obtained using Bruker Esquire ion trap mass spectrometer in positive mode. UV-vis absorption spectra were recorded on an UV 2700. Emission spectra were recorded on a Hitachi F-4600. EPR spectra were recorded on a JEOL JES FA-200 spectrometer (sensitivity, 7*109 spins; resolution, ≥ 2.35 μT).

Chromatography

Thin layer chromatography (TLC) was performed using TLC plates pre-coated with 200 μm thickness silica gel F254 plates and visualized by fluorescence quenching under 254 nm UV light, phosphomolybdic Acid, and $KMnO_4$ stain. Column chromatography was performed on silica gel (particle size 10-40 μm, Ocean Chemical Factory of Qingdao, China).

Photochemistry

All reactions with blue light were carried out using a photoreactor equipped with a blue LED module (25 W Power LED blau 450 nm, purchased from GreeThink), consisting out of 50 LED-chips. The power of the LED was adjusted using a linear regulator.

X-ray crystallographic Analysis

A crystal was mounted on a nylon loop using perfluoropolyether, and transferred to a XTALAB SYNERGY CUSTOM diffractometer, Rigaku (Cu K_α radiation, λ=1.54178 Å) equipped with a nitrogen flow apparatus. The sample was held at 100(2) K or room temperature during the experiment.

EXPERIMENTAL DATA

The *i*-C_3F_7-iodine(III) reagent synthesis

Chloroiodine(III) intermediate[1]

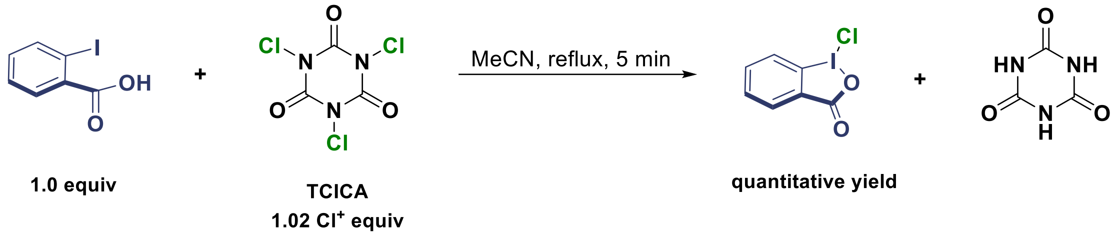

A 500 mL three-necked, round-bottom flask equipped with a teflon-coated magnetic stirring bar, nitrogen inlet and dropping funnel with pressure-equalizing side arm was charged under nitrogen with solid 2-iodobenzoic acid (20 g, 79.0 mmol, 1 equiv.), and anhydrous MeCN (150 mL) was added. The resulting stirred suspension was heated to 75 °C in an oil bath. The dropping funnel was charged with a solution of trichloroisocyanuric acid (6.37 g, 26.6 mol, 1.02 Cl^+ equiv.) in 30 mL of anhydrous MeCN. The solution of trichloroisocyanuric acid was dropped into the vigorously stirred reaction mixture within 5 min. The dropping funnel was rinsed with further anhydrous MeCN (10 mL). After addition was complete, the reaction mixture was refluxed for an additional 5 min. The reaction mixture was vacuum-filtered over an oven-preheated, sintered-glass funnel with a tightly packed pad of Celite (0.5 cm thick), and the filter cake was rinsed with additional hot MeCN (10-20 mL). The combined filtrates were evaporated to near-dryness, and the resulting yellow solid was filtered over a sintered-glass funnel and washed with a small amount of cold MeCN. The solid was dried for 1 h under high vacuum to give 1-chloro-1,2-benziodoxol-3-(1H)-one as free-flowing light yellow crystals in quantitative yield.

NMR Spectroscopy:

^{1}H NMR (400 MHz, Chloroform-*d*, 298 K) δ 8.23 (ddd, *J* = 14.0, 8.0, 1.3 Hz, 2H), 7.98 (ddd, *J* = 8.6, 7.2, 1.6 Hz, 1H), 7.82 - 7.76 (m, 1H).

^{13}C NMR (101 MHz, Chloroform-*d*, 298 K) δ 117.1, 126.8, 128.9, 131.9, 133.4, 136.5, 167.3.

Acetoxyiodine(III) intermediate[2]

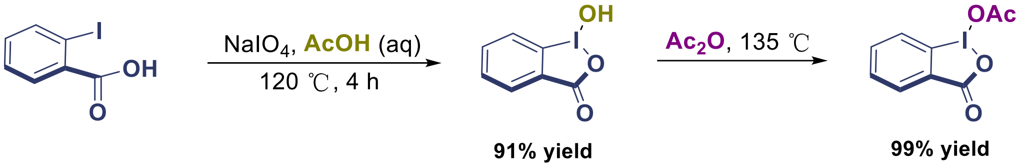

To a suspension of 2-iodobenzoic acid (8.0 g, 32.2 mmol, 1.0 equiv.) in 30% v/v aq. AcOH (48 mL) was added $NaIO_4$ (7.24g, 33.8 mmol, 1.05 equiv.) at room temperature. The mixture was stirred at

120 °C for 4 h and then cooled down to room temperature followed by addition of water (50 mL). While avoiding exposure of ambient light, the precipitate was filtered, further washed with water (20 mL) and cold acetone (20 mL) and dried under vacuum to give 1-hydroxy-1,2-benziodoxol-3-(1*H*)-one as a white crystalline (7.78 g, 93% yield).

NMR Spectroscopy:

^{1}H NMR (400 MHz, Dimethyl sulfoxide-d_6, 298 K) δ 8.02 (dd, *J* = 7.7, 1.4 Hz, 1 H), 7.99 - 7.95 (m, 1 H), 7.85 (dd, *J* = 8.2, 0.7 Hz, 1 H), 7.71 (td, *J* = 7.6, 1.2 Hz, 1 H).

^{13}C NMR (101 MHz, Dimethyl sulfoxide-d_6, 298 K) δ 167.7, 134.5, 131.5, 131.1, 130.4, 126.3, 120.4.

Acetic anhydride (25 mL) was added to 1-hydroxy-1,2-benziodoxol-3-(1*H*)-one (7.78 g, 29.5 mmol) at room temperature. The suspension was allowed to stir at 135 °C for 2 hours until fully dissolved. The resulting solution was slowly cooled down to -20 °C for crystallization. The white crystal was filtered and dried under vacuum to obtain 1-acetoxy-1,2-benziodoxol-3-(1*H*)-one (8.9 g, 99% yield).

NMR Spectroscopy:

^{1}H NMR (400 MHz, Chloroform-*d*, 298 K) δ 8.23 (dd, *J* = 7.6, 1.6 Hz, 1H), 8.00 (dd, *J* = 8.3, 1.0 Hz, 1H), 7.92 (ddd, *J* = 8.4, 7.2, 1.6 Hz, 1H), 7.71 (td, *J* = 7.3, 1.1 Hz, 1H), 2.24 (s, 3H).

^{13}C NMR (101 MHz, Chloroform-*d*, 298 K) δ 176.4, 168.2, 136.2, 133.3, 131.5, 129.4, 129.1, 118.4, 20.3.

Amide-type chloroiodine(III) intermediate[3]

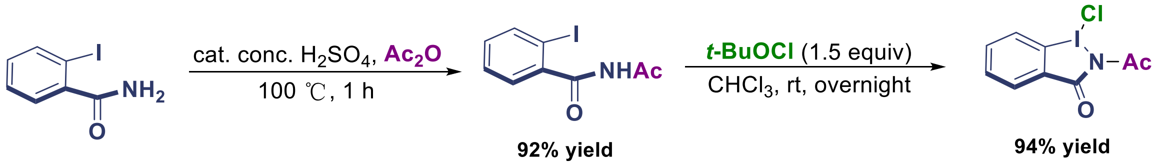

To a suspension of 2-iodobenzamide (4.94 g, 20.0 mmol, 1.0 equiv.) in acetic anhydride (10 mL) was added 10 drops of conc. H_2SO_4 at room temperature. The reaction mixture was stirred at 110 °C and monitored by TLC. After the reaction, the solution was washed with $NaHCO_3$ aq. and extracted with ethyl acetate (3×50 mL), dried over anhydrous Na_2SO_4. The solvent was evaporated under reduced pressure and crude product was purified by flash column chromatography (petroleum ether: ethyl acetate = 4: 1) to give *N*-acetyl-2-iodobenzamide as a white solid (5.32 g, 92% yield).

NMR Spectroscopy:

^{1}H NMR (400 MHz, Chloroform-*d*, 298 K) δ 8.92 (br, 1H), 7.86 (d, *J* = 7.9 Hz, 1H), 7.39 (d, *J* = 4.4 Hz, 2H), 7.14 - 7.11 (m, 1H), 2.48 (s, 3H).

^{13}C NMR (101 MHz, Chloroform-*d*, 298 K) δ 172.7, 167.8, 140.2, 139.9, 132.1, 128.3, 128.1, 92.0, 25.3.

To a solution of *N*-acetyl-2-iodobenzamide (4.34 g, 15 mmol, 1.0 equiv.) in 20 mL $CHCl_3$, *t*-BuOCl (2.44 g, 22.5 mmol, 1.5 equiv.) was added dropwise. The reaction mixture was stirred at room temperature overnight in dark under air. The precipitate was filtered and washed with Et_2O (20 mL). *N*-acetyl-1-chloro-1,2-benziodamine-3-(1*H*)-one was obtained as a colorless solid (4.55 g, 94% yield).

NMR Spectroscopy:

^{1}H NMR (400 MHz, Dimethyl sulfoxide-d_6, 298 K) δ 8.43 (d, *J* = 8.3Hz, 1H), 8.14 - 8.07 (m, 2H), 7.88 (t, *J* = 7.3 Hz, 1H), 2.55 (s, 3H).

^{13}C NMR (101 MHz, Dimethyl sulfoxide-d_6, 298 K) δ 174.4, 161.9, 136.9, 134.4, 131.8, 131.7, 128.2, 115.0, 25.5.

The *i*-C_3F_7-iodine(III)

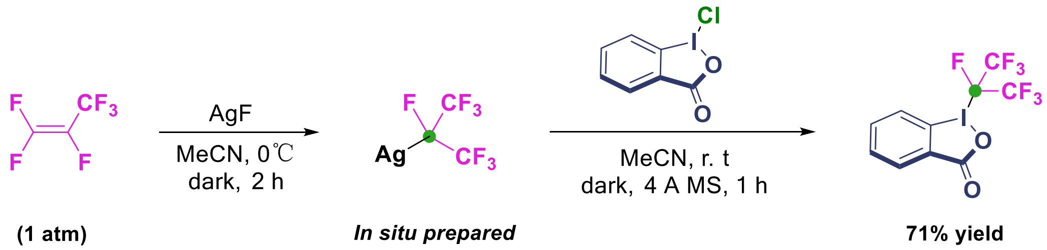

Method A.

In a nitrogen-filled glove box, an oven-dried crimp cap vessel with Teflon-coated stirrer bar was charged with silver fluoride (0.64 g, 5.0 mmol, 1.0 equiv.) and was brought under an atmosphere of dry nitrogen. To this vessel, anhydrous acetonitrile (20 mL) and hexafluoropropylene (1atm, balloon and adequate) were added, and the mixture was stirred at ice-water bath in the dark until silver fluoride precipitate dissolved completely. Then this solution was added to another oven-dried vessel, which filling with 1-chloro-1,2-benziodoxol-3-(1*H*)-one (1.44 g, 5.1 mmol, 1.02 equiv.) and 4 Å molecular sieves (100 mg). The reaction mixture was stirred at ambient temperature in the dark for 1 hours. The reaction mixture was filtered over a sintered-glass funnel with a tightly packed pad of Celite (0.5 cm thick) under dry nitrogen atmosphere, and the filter cake was rinsed with additional anhydrous acetonitrile (5 - 10 mL). The solvent was evaporated under reduced pressure and crude solid product washed with anhydrous Et_2O (10 - 20 mL). Then, the product was obtained by anhydrous DCM leaching in ultrasonic generator under dry nitrogen atmosphere and vacuum evaporated of solvent. The solid was dried for 1 h under high vacuum to give 1-isoperfluoropropyl-1,2-benziodoxol-3-(1*H*)-one as off white solid (1.48 g, 71% yield).

Method B.

Under the Schlenk line, an oven-dried Schlenk flask with Teflon-coated stirrer bar was charged with silver fluoride (0.64 g, 5.0 mmol, 1.0 equiv.) and was brought under an atmosphere of dry nitrogen. To this vessel, anhydrous acetonitrile (20 mL) and hexafluoropropylene (1atm, balloon and adequate) were added, and the mixture was stirred at ice-water bath in the dark until silver fluoride precipitate dissolved completely. Then this solution was added to another oven-dried vessel, which filling with 1-chloro-1,2-benziodoxol-3-(1H)-one (1.44 g, 5.1 mmol, 1.02 equiv.) and 4 Å molecular sieves (100 mg). The reaction mixture was stirred at ambient temperature in the dark for 1 hours. Under nitrogen protection, the filtrate was extracted through catheter tied with filter paper and transferred to another drying bottle, and the filter cake was rinsed with additional anhydrous acetonitrile (5 - 10 mL). The solvent was evaporated under reduced pressure and crude solid product washed with anhydrous Et_2O (10 - 20 mL). Then, the product was obtained by anhydrous DCM leaching in ultrasonic generator under dry nitrogen atmosphere and vacuum evaporated of solvent. The solid was dried for 1 h under high vacuum to give 1-isoperfluoropropyl-1,2-benziodoxol-3-(1*H*)-one as off white solid (1.23 g, 59% yield).

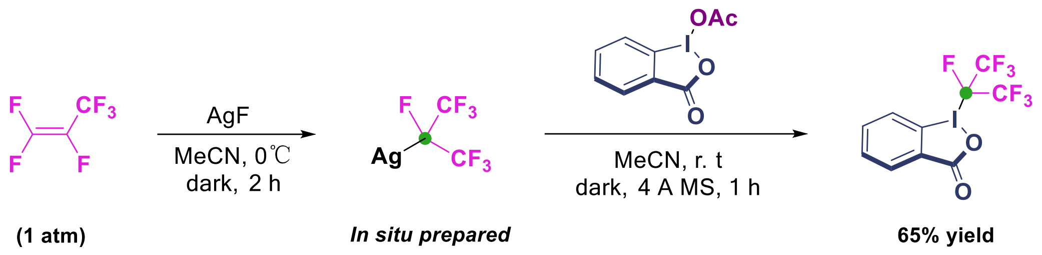

In a nitrogen-filled glove box, an oven-dried crimp cap vessel with Teflon-coated stirrer bar was charged with silver fluoride (0.64 g, 5.0 mmol, 1.0 equiv.) and was brought under an atmosphere of dry nitrogen. To this vessel, anhydrous acetonitrile (20 mL) and hexafluoropropylene (1atm, balloon and adequate) were added, and the mixture was stirred at ice-water bath in the dark until silver fluoride precipitate dissolved completely. Then this solution was added to another oven-dried vessel, which filling with 1-acetoxy-1,2-benziodoxol-3-(1*H*)-one (1.56 g, 5.1 mmol, 1.02 equiv.) and 4 Å molecular sieves (100 mg). The reaction mixture was stirred at ambient temperature in the dark for 1 hours. The reaction mixture was filtered over a sintered-glass funnel with a tightly packed pad of Celite (0.5 cm thick) under dry nitrogen atmosphere, and the filter cake was rinsed with additional anhydrous acetonitrile (5 - 10 mL). The solvent was evaporated under reduced pressure and crude solid product washed with anhydrous Et_2O (10 - 20 mL). Then, the product was obtained by anhydrous DCM leaching in ultrasonic generator under dry nitrogen atmosphere and vacuum evaporated of solvent. The solid was dried for 1 h under high vacuum to give 1-isoperfluoropropyl-1,2-benziodoxol-3-(1*H*)-one as off white solid (1.35 g, 65% yield).

NMR Spectroscopy:

^{1}H NMR (400 MHz, Chloroform-*d*, 298 K) δ 8.25 (dd, *J* = 7.6, 1.5 Hz, 1H), 8.01 (ddd, *J* = 8.5, 7.1, 1.5 Hz, 1H), 7.94 (d, *J* = 8.3 Hz, 1H), 7.78 - 7.70 (m, 1H).

^{13}C NMR (101 MHz, Chloroform-*d*, 298 K) δ 167.9, 142.91 (qd, *J* = 288.9, 29.3 Hz, CF($\underline{C}F_3$)$_2$), 136.7, 133.1, 131.6, 127.99 (d, *J* = 6.7 Hz), 127.8, 120.5 (d, *J* = 10.9 Hz).

^{19}F NMR (376 MHz, Chloroform-*d*, 298 K) δ -71.21 (d, *J* = 11.8 Hz), -171.55 (p, *J* = 12.4 Hz).

Elemental analysis: C%, theoretical value: 28.87%, measured value: 28.99%. H%, theoretical value: 0.97%, measured value: 1.06%. O%, theoretical value: 7.69%, measured value: 7.54%.

UV-Vis Spectrum:

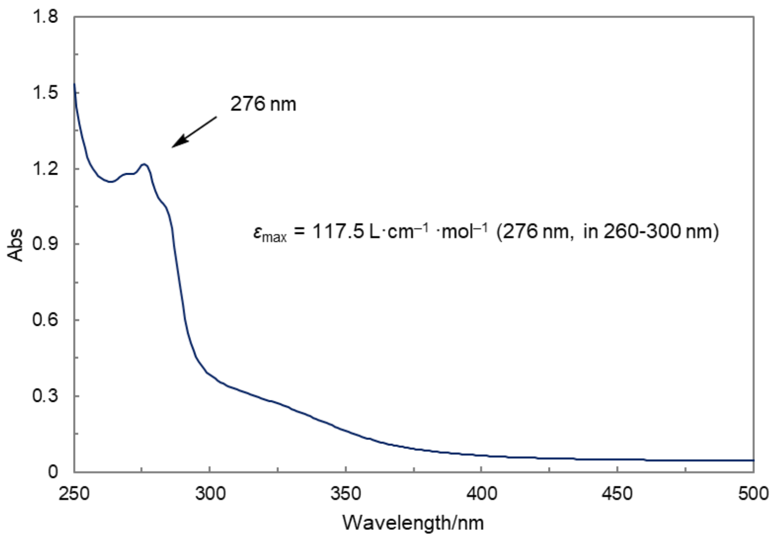

Figure S1. UV-Vis spectrum of *i*-C_3F_7-iodine(III) in MeCN.

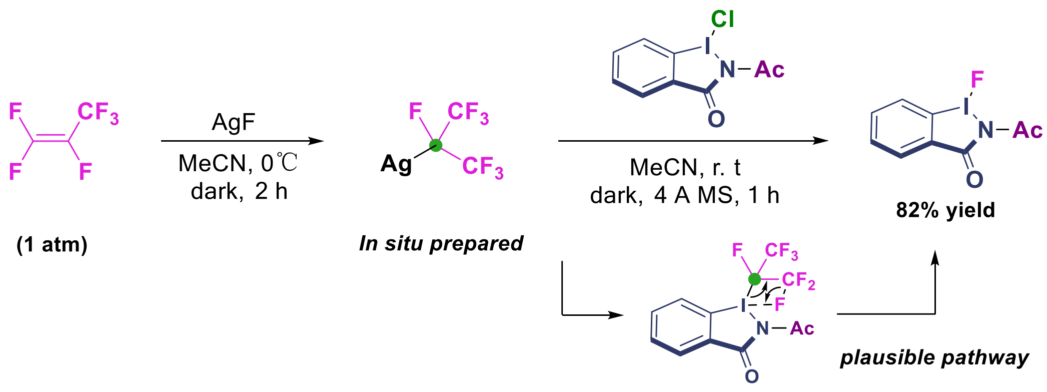

In a nitrogen-filled glove box, an oven-dried crimp cap vessel with Teflon-coated stirrer bar was charged with silver fluoride (0.64 g, 5.0 mmol, 1.0 equiv.) and was brought under an atmosphere of dry nitrogen. To this vessel, anhydrous acetonitrile (20 mL) and hexafluoropropylene (1atm, balloon and adequate) were added, and the mixture was stirred at ice-water bath in the dark until

silver fluoride precipitate dissolved completely. Then this solution was added to another oven-dried vessel, which filling with *N*-acetyl-1-chloro-1,2-benziodamine-3-(1*H*)-one (1.65 g, 5.1 mmol, 1.02 equiv.) and 4 Å molecular sieves (100 mg). The reaction mixture was stirred at ambient temperature in the dark for 1 hours. The reaction mixture was filtered over a sintered-glass funnel with a tightly packed pad of Celite (0.5 cm thick) under dry nitrogen atmosphere, and the filter cake was rinsed with additional anhydrous acetonitrile (5 - 10 mL). The solvent was evaporated under reduced pressure and crude solid product washed with anhydrous Et_2O (10 - 20 mL). Then, the product was obtained by anhydrous DCM leaching in ultrasonic generator under dry nitrogen atmosphere and vacuum evaporated of solvent. The solid was dried for 1 h under high vacuum to give *N*-acetyl-1-fluoro-1,2-benziodamine-3-(1*H*)-one as off solid (1.28 g, 82% yield).

NMR Spectroscopy:

^{1}H NMR (400 MHz, Chloroform-*d*, 298 K) δ 8.21 (d, *J* = 7.6 Hz, 1H), 8.10 (d, *J* = 8.3 Hz, 1H), 7.96 - 7.89 (m, 1H), 7.72 (t, *J* = 7.1 Hz, 1H), 2.68 (s, 3H).

^{13}C NMR (101 MHz, Chloroform-*d*, 298 K) δ 176.5 (d, *J* = 4.0 Hz), 162.3, 136.3, 134.1, 131.9, 130.8, 129.2 (d, *J* = 10.2 Hz), 116.5 (d, *J* = 7.9 Hz), 25.4 (d, *J* = 7.4 Hz).

^{19}F NMR (376 MHz, Chloroform-*d*, 298 K) δ -112.09.

Direct isoperfluoropropylation of electron-rich heterocycles

Optimization of reaction conditions

Solvent screening

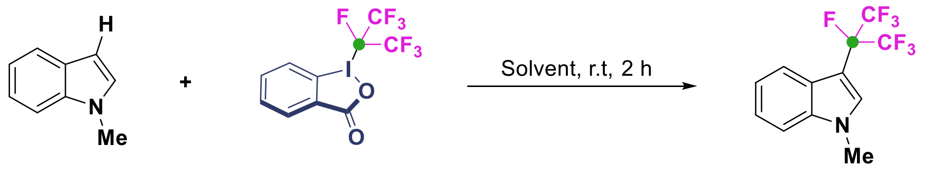

1a-1 2 4

In a 25 mL screw-cap vial equipped with a magnetic stirring bar, *N*-methylindole (**1a-1**, 13.1 mg, 0.10 mmol, 1.0 equiv.) and *i*-C_3F_7-iodine(III) (83.2 mg, 0.20 mmol, 2.0 equiv) were dissolved in solvent (1 mL). The reaction was stirred for 2 hours at room temperature under an atmosphere of dry nitrogen. 10 μL of *n*-dodecane was added as an internal standard and the reaction mixture was diluted with EtOAc. The crude reaction mixture was filtered through a plug of silica then subjected to GC-MS analysis.

Table S1. Solvent screening

Entry	Solvent	GC yield [%]
**1**	MeCN	92
**2**	THF	87
**3**	DCM	19
**4**	DMF	91

Additives screening

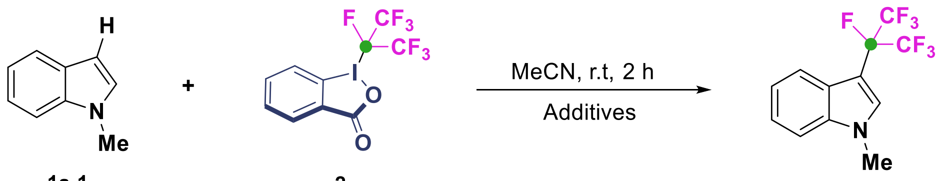

1a-1 2 4

In a 25 mL screw-cap vial equipped with a magnetic stirring bar, *N*-methylindole (**1a-1**, 13.1 mg, 0.10 mmol, 1.0 equiv.), *i*-C_3F_7-iodine(III) (83.2 mg, 0.20 mmol, 2.0 equiv) and additives were dissolved in MeCN (1 mL). The reaction was stirred for 2 hours at room temperature under an atmosphere of dry nitrogen. 10 μL of *n*-dodecane was added as an internal standard and the reaction mixture was diluted with EtOAc. The crude reaction mixture was filtered through a plug of silica then subjected to GC-MS analysis.

Table S2. Additives screening

Entry	Additives, 5 mol%	GC yield [%]
**1**	-	92
**2**	$Zn(OTf)_2$	90
**3**	CuBr	77
**4**	$B(Et)_3$	86

Substrate scope

General procedure to access isoperfluoropropylated products

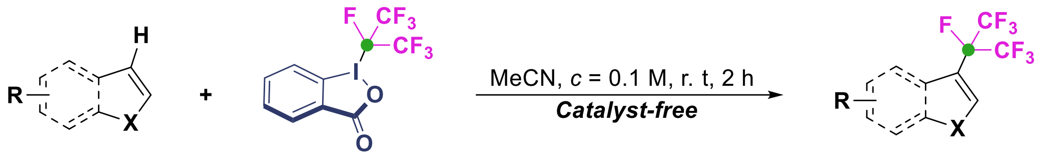

In a 25 mL screw-cap vial equipped with a magnetic stirring bar, electron-rich heterocycles (**1a**, 0.20 mmol, 1.0 equiv.), *i*-C_3F_7-iodine(III) (166.4 mg, 0.40 mmol, 2.0 equiv) and additives were dissolved in MeCN (2 mL). The reaction was stirred for 2 hours at room temperature under an atmosphere of dry nitrogen. The reaction was quenched with 10 mL 5% $NaHCO_3$ aqueous solution, then extracted with DCM (3 x 10 mL). The combined organic phase was dried with anhydrous Na_2SO_4 and concentrated under vacuum. The residue was further purified by column chromatography to afford corresponding isoperfluoropropylated products.

1-Methyl-3-*iso*-perfluoropropyl-1*H*-indole (**4**)

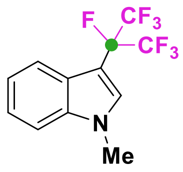

4

1-Methyl-3-*iso*-perfluoropropyl-1*H*-indole (**4**) was prepared according to the general procedure using 1-Methyl-1*H*-indole (26.2 mg, 0.20 mmol). The obtained residue was purified by chromatography on silica gel eluting with EtOAc: petroleum ether (1: 50 - 1: 30 (v/v)) to afford 55.0 mg of **4** as a white solid (92% yield).

R*f* = 0.51 (EtOAc: petroleum ether, 1:20 (v:v)).

NMR Spectroscopy:

^{1}**H NMR** (400 MHz, Chloroform-*d*, 298 K) δ 7.68 (d, *J* = 8.2 Hz, 1H), 7.45 - 7.33 (m, 2H), 7.20 (td, *J* = 7.2, 6.2, 1.8 Hz, 1H), 6.94 (s, 1H), 3.90 (d, *J* = 2.5 Hz, 3H).

^{13}C NMR (101 MHz, Chloroform-*d*, 298 K) δ 139.09, 126.40, 124.37, 122.16 (d, *J* = 3.4 Hz), 121.88, 120.87, 120.64 (qd, *J* = 285.6, 29.0 Hz, CF(<u>C</u>F$_3$)$_2$),109.90, 106.26, 92.96 - 89.85 (m, <u>C</u>F(CF$_3$)$_2$), 32.34 (d, *J* = 11.7 Hz).

^{19}F NMR (376 MHz, Chloroform-*d*, 298 K) δ -74.91 (d, *J* = 8.6 Hz, CF(C<u>F</u>$_3$)$_2$, 6F), -179.12 – -178.48 (m, C<u>F</u>(CF$_3$)$_2$, 1F).

GC-MS: 299

HRMS (ESI) m/z [M+H]$^+$: Calculated for $C_{12}H_9F_7N^+$: 300.0618. Found: 300.0628.

Melt Point: 57.2 - 58.8 °C

1, 2-Dimethyl-3-*iso*-perfluoropropyl-1*H*-indole (**5**)

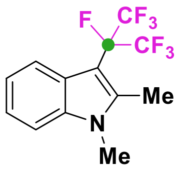

5

1, 2-Dimethyl-3-*iso*-perfluoropropyl-1*H*-indole (**5**) was prepared according to the general procedure using 1, 2-dimethyl-1*H*-indole (29.0 mg, 0.20 mmol). The obtained residue was purified by chromatography on silica gel eluting with EtOAc: petroleum ether (1: 50 - 1: 30 (v/v)) to afford 54.5 mg of **5** as a white solid (87% yield).

R$_f$ = 0.48 (EtOAc: petroleum ether, 1:20 (v:v)).

NMR Spectroscopy:

^{1}H NMR (400 MHz, Chloroform-*d*, 298 K) δ 7.66 (s, 1H), 7.34 - 7.29 (m, 1H), 7.25 (m, 1H), 7.18 - 7.11 (m, 1H), 3.71 (s, 3H), 2.53(s, 3H).

^{13}C NMR (101 MHz, Chloroform-*d*, 298 K) δ 136.82, 121.57 (qd, *J* = 287.0, 27.8 Hz, CF(<u>C</u>F$_3$)$_2$), 121.89, 120.95, 119.77, 109.39, 93.66 - 88.47 (m, <u>C</u>F(CF$_3$)$_2$), 29.64, 11.90.

^{19}F NMR (376 MHz, Chloroform-*d*, 298 K) δ -75.45 (s, CF(C<u>F</u>$_3$)$_2$, 6F), -177.28 (s, C<u>F</u>(CF$_3$)$_2$, 1F).

GC-MS: 313

HRMS (ESI) m/z [M+H]$^+$: Calculated for $C_{13}H_{11}F_7N^+$: 314.0774. Found: 314.0772.

Melt Point: 55.3 - 57.0 °C

5-Chloro-1-methyl-3-*iso*-perfluoropropyl-1*H*-indole (**6**)

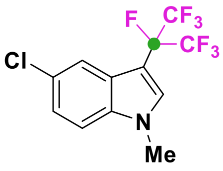

6

5-Chloro-1-methyl-3-*iso*-perfluoropropyl-1*H*-indole (**6**) was prepared according to the general procedure using 5-Chloro-1-methyl-1*H*-indole (33.1 mg, 0.20 mmol). The obtained residue was purified by chromatography on silica gel eluting with EtOAc: petroleum ether (1: 50 - 1: 30 (v/v)) to afford 62.7 mg of **6** as a white solid (94% yield).

R_f = 0.37 (EtOAc: petroleum ether, 1:20 (v:v)).

NMR Spectroscopy:

^{1}H NMR (400 MHz, Chloroform-*d*, 298 K) δ 7.63 - 7.59 (m, 1H), 7.30 (d, *J* = 1.4 Hz, 2H), 6.85 (d, *J* = 2.3 Hz, 1H), 3.88 (d, *J* = 3.1 Hz, 3H).

^{13}C NMR (101 MHz, Chloroform-*d*, 298 K) δ 137.48, 127.22, 126.67, 124.86, 123.39 (d, *J* = 17.9 Hz), 122.66 (qd, *J* = 287.0, 27.8 Hz, CF($\underline{C}F_3)_2$), 121.09, 111.04, 105.66, 92.77 - 90.09 (m, $\underline{C}F(CF_3)_2$), 32.59 (d, *J* = 12.0 Hz).

^{19}F NMR (376 MHz, Chloroform-*d*, 298 K) δ -74.96 (d, *J* = 9.5 Hz, CF(C$\underline{F}_3)_2$, 6F), -179.39 – -179.65 (m, C$\underline{F}(CF_3)_2$, 1F).

GC-MS: 333

HRMS (ESI) m/z $[M+H]^+$: Calculated for $C_{12}H_8ClF_7N^+$: 334.0228. Found: 334.0233.

Melt Point: 64.0 - 65.5 °C

5-Bromo-1-methyl-3-*iso*-perfluoropropyl-1*H*-indole (**7**)

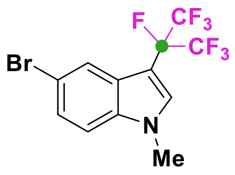

7

5-Bromo-1-methyl-3-*iso*-perfluoropropyl-1*H*-indole (**7**) was prepared according to the general procedure using 5-Bromo-1-methyl-1*H*-indole (41.8 mg, 0.20 mmol). The obtained residue was purified by chromatography on silica gel eluting with EtOAc: petroleum ether (1: 50 - 1: 30 (v/v)) to afford 67.9 mg of **7** as an off-white solid (90% yield).

R_f = 0.6 (EtOAc: petroleum ether, 1:10 (v:v)).

NMR Spectroscopy:

^{1}H NMR (400 MHz, Chloroform-*d*, 298 K) δ 7.78 (d, *J* = 1.9 Hz, 1H), 7.43 (dd, *J* = 8.8, 1.9 Hz, 1H), 7.27 (d, *J* = 8.8 Hz, 1H), 6.84 (d, *J* = 2.4 Hz, 1H), 3.87 (d, *J* = 3.1 Hz, 3H).

^{13}C NMR (101 MHz, Chloroform-*d*, 298 K) δ 137.72, 127.88, 127.37, 124.25, 123.23 (d, *J* = 18.2 Hz), 120.39 (qd, *J* = 287.2, 27.9 Hz, CF($\underline{C}F_3)_2$), 114.08, 111.45, 105.53, 93.66 - 88.47 (m, $\underline{C}F(CF_3)_2$), 32.60 (d, *J* = 12.1 Hz).

^{19}F NMR (376 MHz, Chloroform-*d*, 298 K) δ -74.94 (d, *J* = 8.8 Hz, CF(C$\underline{F}_3)_2$, 6F), -179.50 – -

179.66 (m, C*F*(CF$_3$)$_2$, 1F).

GC-MS: 377

HRMS (ESI) m/z [M+H]$^+$: Calculated for $C_{12}H_8BrF_7N^+$: 377.9723. Found: 377.9729.

Melt Point: 69.8 - 71.9 °C

Methyl 1-methyl-3-*iso*-perfluoropropyl-indole-6-carboxylate (**8**)

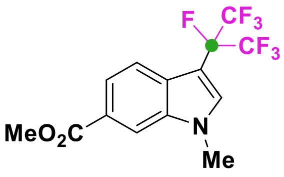

8

Methyl 1-methyl-3-*iso*-perfluoropropyl-indole-6-carboxylate (**8**) was prepared according to the general procedure using methyl 1-methylindole-6-carboxylate (37.8 mg, 0.20 mmol). The obtained residue was purified by chromatography on silica gel eluting with EtOAc: petroleum ether (1: 50 - 1: 30 (v/v)) to afford 66.4 mg of **8** as a white solid (93% yield).

R$_f$ = 0.46 (EtOAc: petroleum ether, 1:10 (v:v)).

NMR Spectroscopy:

^{1}**H NMR** (400 MHz, Chloroform-*d*, 298 K) δ 8.13 (s, 1H), 7.88 (d, *J* = 8.6 Hz, 1H), 7.79 (d, *J* = 8.6 Hz, 1H), 7.44 (s, 1H), 3.95 (s, 3H), 3.89 (s, 3H).

^{13}**C NMR** (101 MHz, Chloroform-*d*, 298 K) δ 167.63, 136.45, 131.47 (d, *J* = 11.1 Hz), 129.08, 124.83, 122.05, 121.09 (qd, *J* = 287.8, 27.6 Hz, CF(*C*F$_3$)$_2$), 120.67, 112.37, 100.93 (d, *J* = 24.9 Hz), 93.35 - 90.67 (m, *C*F(CF$_3$)$_2$), 52.23, 33.63.

^{19}**F NMR** (376 MHz, Chloroform-*d*, 298 K) δ -76.34 (d, *J* = 8.9 Hz, CF(C*F*$_3$)$_2$, 6F), -178.65 (hept, *J* = 8.9 Hz, C*F*(CF$_3$)$_2$, 1F).

GC-MS: 357

HRMS (ESI) m/z [M+H]$^+$: Calculated for $C_{14}H_{11}F_7NO_2^+$: 358.0673. Found: 358.0671.

Melt Point: 81.1 - 82.7 °C

1-Methyl-5-nitro-3-*iso*-perfluoropropyl-1*H*-indole (**9**)

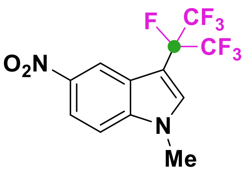

9

1-Methyl-5-nitro-3-*iso*-perfluoropropyl-1*H*-indole (**9**) was prepared according to the general procedure using 1-methyl-5-nitro-1*H*-indole (35.2 mg, 0.20 mmol). The obtained residue was purified by chromatography on silica gel eluting with EtOAc: petroleum ether (1: 50 - 1: 30 (v/v)) to

afford 54.4 mg of **9** as a light-yellow solid (79% yield).

R_f = 0.45 (EtOAc: petroleum ether, 1:20 (v:v)).

NMR Spectroscopy:

^{1}H NMR (400 MHz, Chloroform-*d*, 298 K) δ 8.61 (d, *J* = 2.2 Hz, 1H), 8.24 (dd, *J* = 9.2, 2.2 Hz, 1H), 7.46 (d, *J* = 9.2 Hz, 1H), 7.10 (s, 1H), 3.97 (d, *J* = 3.3 Hz, 3H).

^{13}C NMR (101 MHz, Chloroform-*d*, 298 K) δ 142.66, 141.48, 129.87, 128.95, 120.30 (qd, *J* = 286.4, 28.3 Hz, CF(<u>C</u>F$_3$)$_2$), 119.65, 119.10, 110.30, 108.40, 92.71 – 89.26 (m, <u>C</u>F(CF$_3$)$_2$), 33.13 (d, *J* = 12.5 Hz).

^{19}F NMR (376 MHz, Chloroform-*d*, 298 K) δ -74.92 (d, *J* = 9.1 Hz, CF(C<u>F</u>$_3$)$_2$, 6F), -180.53 – -180.06 (m, C<u>F</u>(CF$_3$)$_2$, 1F).

GC-MS: 344

HRMS (ESI) m/z [M+H]$^+$: Calculated for $C_{12}H_8F_7N_2O_2^+$: 345.0472. Found: 345.0474.

Melt Point: 117.4 - 119.0 °C

5-Cyano-1-methyl-3-*iso*-perfluoropropyl-1*H*-indole (**10**)

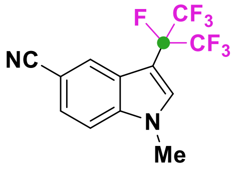

10

5-Cyano-1-methyl-3-*iso*-perfluoropropyl-1*H*-indole (**10**) was prepared according to the general procedure using 5-Cyano-1-methyl-1*H*-indole (31.2 mg, 0.20 mmol). The obtained residue was purified by chromatography on silica gel eluting with EtOAc: petroleum ether (1: 50 - 1: 30 (v/v)) to afford 53.1 mg of **10** as a white solid (82% yield).

R_f = 0.44 (EtOAc: petroleum ether, 1:15 (v:v)).

NMR Spectroscopy:

^{1}H NMR (400 MHz, Chloroform-*d*, 298 K) δ 8.01 (s, 1H), 7.57 (d, *J* = 8.7 Hz, 1H), 7.47 (d, *J* = 8.7 Hz, 1H), 7.00 (s, 1H), 3.94 (d, *J* = 2.8 Hz, 3H).

^{13}C NMR (101 MHz, Chloroform-*d*, 298 K) δ 140.32, 127.65, 126.92, 126.03, 124.81 (d, *J* = 54.1 Hz), 120.32 (qd, *J* = 288.8, 28.1 Hz, CF(<u>C</u>F$_3$)$_2$), 119.91, 111.08, 106.94, 104.46, 91.06 - 88.38 (m, <u>C</u>F(CF$_3$)$_2$), 32.83 (d, *J* = 36.0 Hz).

^{19}F NMR (376 MHz, Chloroform-*d*, 298 K) δ -74.94 (d, *J* = 8.7 Hz, CF(C<u>F</u>$_3$)$_2$, 6F), -180.31 – -179.83 (m, C<u>F</u>(CF$_3$)$_2$, 1F).

GC-MS: 324

HRMS (ESI) m/z [M+H]$^+$: Calculated for $C_{13}H_8F_7N_2^+$: 325.0576. Found: 325.0581.

Melt Point: 109.3 - 111.4 °C

3-Formyl-1-methyl-2-*iso*-perfluoropropyl-1*H*-indole and 3-formyl-1-methyl-5-*iso*-perfluoropropyl-1*H*-indole (**11**)

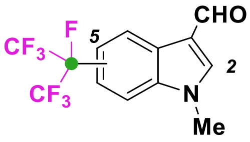

11

3-Formyl-1-methyl-2-*iso*-perfluoropropyl-1*H*-indole and 3-formyl-1-methyl-5-*iso*-perfluoropropyl-1*H*-indole (**11**) was prepared according to the general procedure using 3-formyl-1-methyl-1*H*-indole (31.8 mg, 0.20 mmol). The obtained residue was purified by chromatography on silica gel eluting with EtOAc: petroleum ether (1: 50 - 1: 20 (v/v)) to afford 52.1 mg of **11** as a yellow solid (76% yield).

R*f* = 0.51 (EtOAc: petroleum ether, 1:5 (v:v)).

NMR Spectroscopy:

^{1}**H NMR** (400 MHz, Chloroform-*d*, 298 K) δ 10.43 (d, *J* = 3.0 Hz), 10.22 (s), 8.58 (d, *J* = 8.0 Hz), 7.42 (m), 4.03 (d, *J* = 8.2 Hz), 3.92 (s).

^{13}**C NMR** (101 MHz, Chloroform-*d*, 298 K) δ 187.32 (d, *J* = 27.3 Hz), 186.11, 163.18, 138.97, 138.47, 134.40, 130.66, 130.26, 129.88, 128.96, 128.67, 126.05 (d, *J* = 6.7 Hz), 125.72, 124.40 (d, *J* = 15.8 Hz), 123.76 (d, *J* = 20.5 Hz), 120.96 (qd, *J* = 287.0, 28.6 Hz, CF(<u>C</u>F$_3$)$_2$), 110.15 (d, *J* = 6.4 Hz), 92.78 – 88.33 (m, <u>C</u>F(CF$_3$)$_2$), 34.31 (d, *J* = 20.0 Hz), 32.78.

^{19}**F NMR** (376 MHz, Chloroform-*d*, 298 K) -72.74 (d, *J* = 6.6 Hz, CF(C<u>F</u>$_3$)$_2$), -74.62 (d, *J* = 7.6 Hz, CF(C<u>F</u>$_3$)$_2$), -165.12 – -164.79 (m, C<u>F</u>(CF$_3$)$_2$), -176.98 – -176.67 (m, C<u>F</u>(CF$_3$)$_2$).

GC-MS: 327

HRMS (ESI) m/z [M+H]$^+$: Calculated for $C_{13}H_9F_7NO^+$: 328.0572. Found: 328.0570.

Melt Point: 57.6 - 60.1°C

5-Methoxyl-3-*iso*-perfluoropropyl-1*H*-indole (**12**)

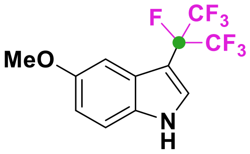

12

5-Methoxyl-3-*iso*-perfluoropropyl-1*H*-indole (**12**) was prepared according to the general procedure using 5-methoxyl-1*H*-indole (29.4 mg, 0.20 mmol). The obtained residue was purified by chromatography on silica gel eluting with EtOAc: petroleum ether (1: 30 - 1: 20 (v/v)) to afford 47.3 mg of **12** as a light-yellow solid (75% yield).

R_f = 0.36 (EtOAc: petroleum ether, 1:5 (v:v)).

NMR Spectroscopy:

^{1}H NMR (400 MHz, Chloroform-*d*, 298 K) δ 8.44 (br, s, 1H), 7.33 (d, *J* = 8.9 Hz, 1H), 7.13 (d, *J* = 2.5 Hz, 1H), 7.01 (dd, *J* = 8.6, 2.8 Hz, 1H), 6.84 (s, 1H), 3.87 (s, 3H).

^{13}C NMR (101 MHz, Chloroform-*d*, 298 K) δ 155.04, 131.63, 127.78, 122.01 (d, *J* = 22.1 Hz), 120.33 (qd, *J* = 288.3, 28.1 Hz, CF($\underline{C}F_3)_2$), 115.63, 112.49, 105.06, 102.56, 91.52 - 88.13 (m, $\underline{C}F(CF_3)_2$), 55.86.

^{19}F NMR (376 MHz, Chloroform-*d*, 298 K) -76.17 (d, *J* = 9.1 Hz, CF(C$\underline{F}_3)_2$), -179.96 (hept, *J* = 9.0 Hz, C$\underline{F}(CF_3)_2$).

GC-MS: 315

HRMS (ESI) m/z $[M+H]^+$: Calculated for $C_{12}H_9F_7NO^+$: 316.0572. Found: 316.0580.

Melt Point: 61.3 - 63.4°C

5-Methyl-3-*iso*-perfluoropropyl-1*H*-indole (**13**)

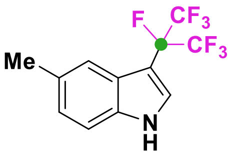

13

5-Methyl-3-*iso*-perfluoropropyl-1*H*-indole (**13**) was prepared according to the general procedure using 5-methyl-1*H*-indole (26.2 mg, 0.20 mmol). The obtained residue was purified by chromatography on silica gel eluting with EtOAc: petroleum ether (1: 30 - 1: 20 (v/v)) to afford 47.8 mg of **13** as a white solid (80% yield).

R_f = 0.39 (EtOAc: petroleum ether, 1:5 (v:v)).

NMR Spectroscopy:

^{1}H NMR (400 MHz, Chloroform-*d*, 298 K) δ 8.35 (br, s, 1H), 7.47 (s, 1H), 7.34 (d, *J* = 8.4 Hz, 1H), 7.16 (d, *J* = 8.4 Hz, 1H), 6.83 (s, 1H), 2.47 (s, 3H).

^{13}C NMR (101 MHz, Chloroform-*d*, 298 K) δ 134.73, 130.63, 127.56, 126.37, 121.53 (d, *J* = 21.5 Hz), 121.18, 120.34 (qd, *J* = 288.3, 28.1 Hz, CF($\underline{C}F_3)_2$), 111.24, 104.97, 91.53 - 88.27 (m, $\underline{C}F(CF_3)_2$), 21.43.

^{19}F NMR (376 MHz, Chloroform-*d*, 298 K) -76.13 (d, *J* = 9.1 Hz, CF(C$\underline{F}_3)_2$), -179.90 (hept, *J* = 9.2 Hz, C$\underline{F}(CF_3)_2$).

GC-MS: 299

HRMS (ESI) m/z $[M+H]^+$: Calculated for $C_{12}H_9F_7N^+$: 300.0621. Found: 300.0624.

Melt Point: 63.4 - 64.9°C

Methyl 3-*iso*-perfluoropropyl-indole-5-carboxylate (**14**)

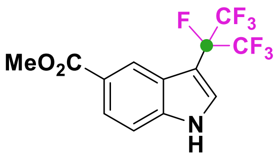

14

Methyl 3-*iso*-perfluoropropyl-indole-5-carboxylate (**14**) was prepared according to the general procedure using Methyl indole-5-carboxylate (35.0 mg, 0.20 mmol). The obtained residue was purified by chromatography on silica gel eluting with EtOAc: petroleum ether (1: 30 - 1: 20 (v/v)) to afford 59.0 mg of **14** as a white solid (86% yield).

R_f = 0.35 (EtOAc: petroleum ether, 1:5 (v:v)).

NMR Spectroscopy:

^{1}**H NMR** (400 MHz, Dimethyl sulfoxide-d_6, 298 K) δ 12.47 (br, s, 1H), 8.38 (s, 1H), 7.85 (d, J = 8.7 Hz, 1H), 7.56 (d, J = 9.3 Hz, 1H), 7.09 (s, 1H), 3.82 (s, 3H).

^{13}**C NMR** (101 MHz, Dimethyl sulfoxide-d_6, 298 K) δ 167.30, 139.94, 126.76, 125.04, 124.73, 122.87, 122.63, 120.43 (qd, *J* = 285.5, 27.3 Hz, CF($\underline{C}F_3)_2$), 112.87, 106.05, 91.53 - 88.36 (m, $\underline{C}F(CF_3)_2$), 52.36.

^{19}**F NMR** (376 MHz, Dimethyl sulfoxide-d_6, 298 K) δ -75.63 (d, *J* = 9.5 Hz, CF(C$\underline{F}_3)_2$, 6F), -178.72 – -178.43 (m, C$\underline{F}(CF_3)_2$, 1F).

GC-MS: 343

HRMS (ESI) m/z $[M+H]^+$: Calculated for $C_{13}H_9F_7NO_2^+$: 344.0521. Found: 344.0527.

Melt Point: 70.3 - 72.4 ℃

5-Bromo-3-*iso*-perfluoropropyl-1*H*-indole (**15**)

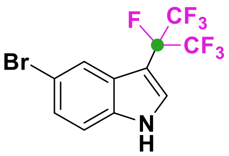

15

5-Bromo-3-*iso*-perfluoropropyl-1*H*-indole (**15**) was prepared according to the general procedure using 5-bromo-1*H*-indole (39.0 mg, 0.20 mmol). The obtained residue was purified by chromatography on silica gel eluting with EtOAc: petroleum ether (1: 30 - 1: 20 (v/v)) to afford 65.3 mg of **15** as a white solid (90% yield).

R_f = 0.42 (EtOAc: petroleum ether, 1:5 (v:v)).

NMR Spectroscopy:

^{1}**H NMR** (400 MHz, Dimethyl sulfoxide-d_6, 298 K) δ 12.29 (br, s, 1H), 7.86 (s, 1H), 7.44 (d, *J* = 8.7 Hz, 1H), 7.36 (d, *J* = 8.8 Hz, 1H), 6.90 (s, 1H).

^{13}**C NMR** (101 MHz, Dimethyl sulfoxide-d_6, 298 K) δ 136.21, 128.88, 127.17, 124.11, 122.34 (d, *J* = 22.1 Hz), 120.43 (qd, *J* = 285.5, 27.3 Hz, CF($\underline{C}F_3)_2$), 114.84, 113.42, 104.04, 91.50 -

88.14 (m, CF(CF_3)$_2$).

^{19}F NMR (376 MHz, Dimethyl sulfoxide-*d$_6$*, 298 K) δ -75.57 (d, *J* = 9.1 Hz, CF(C*F*$_3$)$_2$, 6F), -178.79 – -178.46 (m, C*F*(CF_3)$_2$, 1F).

GC-MS: 363

HRMS (ESI) m/z [M+H]$^+$: Calculated for $C_{11}H_6BrF_7N^+$: 363.9572. Found: 363.9566.

Melt Point: 73.2 - 74.5 ℃

5-Chloro-3-*iso*-perfluoropropyl-1*H*-indole (**16**)

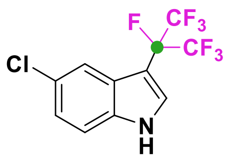

16

5-Chloro-3-*iso*-perfluoropropyl-1*H*-indole (**16**) was prepared according to the general procedure using 5-chloro-1*H*-indole (30.2 mg, 0.20 mmol). The obtained residue was purified by chromatography on silica gel eluting with EtOAc: petroleum ether (1: 30 - 1: 20 (v/v)) to afford 56.1 mg of **16** as a white solid (88% yield).

R$_f$ = 0.41 (EtOAc: petroleum ether, 1:5 (v:v)).

NMR Spectroscopy:

^{1}H NMR (400 MHz, Chloroform-*d*, 298 K) δ 8.47 (br, s, 1H), 7.65 (s, 1H), 7.37 (d, *J* = 8.8 Hz, 1H), 7.28 (dd, *J* = 8.8, 2.8 Hz, 1H), 6.84 (s, 1H).

^{13}C NMR (101 MHz, Chloroform-*d*, 298 K) δ 134.67, 128.27, 127.00, 125.14, 122.99 (d, *J* = 21.9 Hz), 121.12, 120.20 (qd, *J* = 288.3, 27.6 Hz, CF(CF_3)$_2$), 112.69, 104.92, 91.00 - 87.84 (m, CF(CF_3)$_2$).

^{19}F NMR (376 MHz, Chloroform-*d*, 298 K) δ -76.17 (d, *J* = 9.0 Hz, CF(C*F*$_3$)$_2$, 6F), -180.51 (hept, *J* = 9.0 Hz, C*F*(CF_3)$_2$, 1F).

GC-MS: 319

HRMS (ESI) m/z [M+H]$^+$: Calculated for $C_{11}H_6ClF_7N^+$: 320.0074. Found: 320.0085.

Melt Point: 66.7 - 68.5 ℃

6-Fluro-3-*iso*-perfluoropropyl-1*H*-indole (**17**)

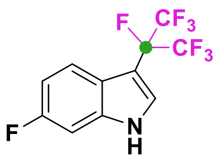

17

6-Fluro-3-*iso*-perfluoropropyl-1*H*-indole (**17**) was prepared according to the general procedure using 5-fluro-1*H*-indole (27.0 mg, 0.20 mmol). The obtained residue was purified by

chromatography on silica gel eluting with EtOAc: petroleum ether (1: 30 - 1: 20 (v/v)) to afford 47.9 mg of **17** as a white solid (79% yield).

R$_f$ = 0.45 (EtOAc: petroleum ether, 1:5 (v:v)).

NMR Spectroscopy:

^{1}H NMR (400 MHz, Dimethyl sulfoxide-d_6, 298 K) δ 12.16 (br s, 1H), 7.63 (dd, J = 8.8, 5.4 Hz, 1H), 7.21 (d, J = 9.9 Hz, 1H), 7.00 - 6.86 (m, 2H).

^{13}C NMR (101 MHz, Dimethyl sulfoxide-d_6, 298 K) δ 160.64 (d, J = 238.3 Hz), 137.52 (d, J = 13.4 Hz), 123.91, 123.30 (d, J = 10.8 Hz), 121.56 (d, J = 22.1 Hz), 120.49 (qd, J = 285.9, 27.3 Hz, CF($\underline{C}F_3)_2$), 110.02 (d, J = 25.1 Hz), 104.73, 98.45 (d, J = 26.0 Hz), 92.44 - 86.90 (m, $\underline{C}F(CF_3)_2$).

^{19}F NMR (376 MHz, Dimethyl sulfoxide-d_6, 298 K) δ -75.80 (d, J = 10.7 Hz, CF(C$\underline{F}_3)_2$, 6F), -118.03 (s, 1F), -178.15 – -177.93 (m, C$\underline{F}(CF_3)_2$, 1F).

GC-MS: 303

HRMS (ESI) m/z [M+H]$^+$: Calculated for $C_{11}H_6F_8N^+$: 304.0370. Found: 304.0366.

Melt Point: 60.4 - 62.4℃

5-Cyano-3-*iso*-perfluoropropyl-1*H*-indole (**18**)

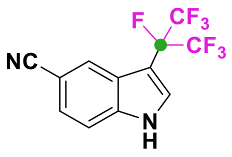

18

5-Cyano-3-*iso*-perfluoropropyl-1*H*-indole (**18**) was prepared according to the general procedure using 5-cyano-1*H*-indole (28.4 mg, 0.20 mmol). The obtained residue was purified by chromatography on silica gel eluting with EtOAc: petroleum ether (1: 30 - 1: 20 (v/v)) to afford 45.9 mg of **18** as a white solid (74% yield).

R$_f$ = 0.40 (EtOAc: petroleum ether, 1:5 (v:v)).

NMR Spectroscopy:

^{1}H NMR (400 MHz, Dimethyl sulfoxide-d_6, 298 K) δ 12.68 (br s, 1H), 8.21 (d, J = 4.6 Hz, 1H), 7.72 – 7.49 (m, 2H), 7.05 (d, J = 10.2 Hz, 1H).

^{13}C NMR (101 MHz, Dimethyl sulfoxide-d_6, 298 K) δ 139.09, 128.00 (d, J = 7.3 Hz), 126.90, 126.83, 123.65 (d, J = 22.1 Hz), 120.48, 120.33 (qd, J = 285.9, 26.9 Hz, CF($\underline{C}F_3)_2$), 114.20, 105.36, 103.49, 91.62 – 88.12 (m, $\underline{C}F(CF_3)_2$).

^{19}F NMR (376 MHz, Dimethyl sulfoxide-d_6, 298 K) δ -76.99 (d, J = 7.5 Hz, CF(C$\underline{F}_3)_2$, 6F), -180.98 – -180.63 (m, C$\underline{F}(CF_3)_2$, 1F).

GC-MS: 310

HRMS (ESI) m/z $[M+H]^+$: Calculated for $C_{12}H_6F_7N_2^+$: 311.0419. Found: 311.0427.

Melt Point: 84.0 - 86.3℃

5-Bromo-1-ethyl-3-*iso*-perfluoropropyl-1*H*-indole (**19**)

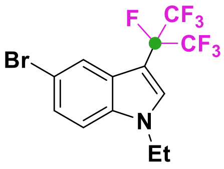

19

5-Bromo-1-ethyl-3-*iso*-perfluoropropyl-1*H*-indole (**19**) was prepared according to the general procedure using 5-bromo-1-ethyl-1*H*-indole (44.6.0 mg, 0.20 mmol). The obtained residue was purified by chromatography on silica gel eluting with EtOAc: petroleum ether (1: 50 - 1: 30 (v/v)) to afford 71.2 mg of **19** as a white solid (91% yield).

R_f = 0.47 (EtOAc: petroleum ether, 1:20 (v:v)).

NMR Spectroscopy:

^{1}**H NMR** (400 MHz, Chloroform-*d*, 298 K) δ 7.91 (s, 1H), 7.41 - 7.32 (m, 2H), 7.25 (d, *J* = 8.8 Hz, 1H), 4.18 (q, *J* = 7.3 Hz, 2H), 1.49 (t, *J* = 7.3 Hz, 3H).

^{13}**C NMR** (101 MHz, Chloroform-*d*, 298 K) δ 134.74, 127.94 (d, *J* = 11.2 Hz), 127.35, 125.95, 123.61 (d, *J* = 5.3 Hz), 121.09 (qd, *J* = 288.3, 29.0 Hz, CF($\underline{C}F_3)_2$), 114.65, 111.45, 100.16 (d, *J* = 24.7 Hz), 93.96 - 90.86 (m, $\underline{C}F(CF_3)_2$), 41.89, 15.22.

^{19}**F NMR** (376 MHz, Chloroform-*d*, 298 K) δ -76.32 (d, *J* = 9.0 Hz, CF(C$\underline{F}_3)_2$, 6F), -178.63 (hept, *J* = 9.2 Hz, C$\underline{F}(CF_3)_2$, 1F).

GC-MS: 391

HRMS (ESI) m/z $[M+H]^+$: Calculated for $C_{13}H_{10}BrF_7N^+$: 391.9883. Found: 391.9894.

Melt Point: 74.6 - 75.9 °C

5-Bromo-1-ethyl-3-*iso*-perfluoropropyl-1*H*-indole (**20**)

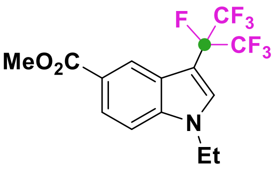

20

Methyl 1-ethyl-3-*iso*-perfluoropropyl-indole-5-carboxylate (**20**) was prepared according to the general procedure using methyl 1-ethylindole-5-carboxylate (40.6 mg, 0.20 mmol). The obtained residue was purified by chromatography on silica gel eluting with EtOAc: petroleum ether (1: 50 - 1: 30 (v/v)) to afford 72.0 mg of **20** as a light-yellow oil (97% yield).

R$_f$ = 0.44 (EtOAc: petroleum ether, 1:20 (v:v)).

NMR Spectroscopy:

^{1}H NMR (400 MHz, Chloroform-*d*, 298 K) δ 8.52 (s, 1H), 8.00 (d, *J* = 8.7 Hz, 1H), 7.40 (d, *J* = 9.0 Hz, 1H), 4.23 (q, *J* = 8.3, 7.8 Hz, 2H), 3.93 (s, 1H), 1.52 (t, *J* = 7.3 Hz, 3H).

^{13}C NMR (101 MHz, Chloroform-*d*, 298 K) δ 167.72, 138.46, 128.37 (d, *J* = 10.8 Hz), 125.36, 124.22, 124.00, 123.39, 121.08 (qd, *J* = 289.3, 29.0 Hz, CF($\underline{C}F_3)_2$), 109.76, 102.13 (d, *J* = 24.8 Hz), 93.55 - 90.54 (m, $\underline{C}F(CF_3)_2$), 52.11, 41.91, 15.25.

^{19}F NMR (376 MHz, Chloroform-*d*, 298 K) δ -76.30 (d, *J* = 8.8 Hz, CF(C$\underline{F}_3)_2$, 6F), -178.67 (hept, *J* = 9.3 Hz, C$\underline{F}(CF_3)_2$, 1F).

GC-MS: 371

HRMS (ESI) m/z $[M+H]^+$: Calculated for $C_{15}H_{13}F_7NO_2^+$: 372.0835. Found: 372.0834.

Melt Point: 77.7 - 79.3 °C

1-Benzyl-5-cyano-3-*iso*-perfluoropropyl-1*H*-indole (**21**)

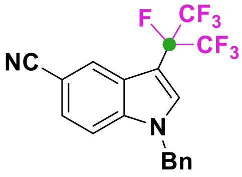

21

1-Benzyl-5-cyano-3-*iso*-perfluoropropyl-1*H*-indole (**21**) was prepared according to the general procedure using 1-benzyl-5-cyano-1*H*-indole (46.4 mg, 0.20 mmol). The obtained residue was purified by chromatography on silica gel eluting with EtOAc: petroleum ether (1: 50 - 1: 30 (v/v)) to afford 66.4 mg of **21** as a yellow oil (83% yield).

R$_f$ = 0.41 (EtOAc: petroleum ether, 1:20 (v:v)).

NMR Spectroscopy:

^{1}H NMR (400 MHz, Chloroform-*d*, 298 K) δ 8.14 (s, 1H), 7.52 (s, 1H), 7.47 (dd, *J* = 8.7, 1.5 Hz, 1H), 7.41 - 7.31 (m, 4H), 7.09 (dd, *J* = 7.6, 2.0 Hz, 2H), 5.39 (s, 2H).

^{13}C NMR (101 MHz, Chloroform-*d*, 298 K) δ 138.07, 135.06, 130.56 (d, *J* = 11.4 Hz), 129.36, 128.67, 126.79, 126.03, 125.56, 120.90 (qd, *J* = 288.8, 28.1 Hz, CF($\underline{C}F_3)_2$), 119.95, 111.67, 105.15, 102.37 (d, *J* = 24.8 Hz), 93.22 - 89.90 (m, $\underline{C}F(CF_3)_2$), 51.11.

^{19}F NMR (376 MHz, Chloroform-*d*, 298 K) δ -76.31 (d, *J* = 9.5 Hz, CF(C$\underline{F}_3)_2$, 6F), -179.15 (hept, *J* = 8.6 Hz, C$\underline{F}(CF_3)_2$, 1F).

GC-MS: 400

HRMS (ESI) m/z $[M+H]^+$: Calculated for $C_{19}H_{12}F_7N_2^+$: 401.0887. Found: 401.0887.

1-Benzyl-5-nitro-3-*iso*-perfluoropropyl-1*H*-indole (**22**)

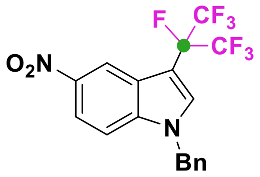

22

1-Benzyl-5-nitro-3-*iso*-perfluoropropyl-1*H*-indole (**22**) was prepared according to the general procedure using 1-benzyl-5-nitro-1*H*-indole (50.4 mg, 0.20 mmol). The obtained residue was purified by chromatography on silica gel eluting with EtOAc: petroleum ether (1: 50 - 1: 30 (v/v)) to afford 64.7 mg of **22** as a yellow solid (77% yield).

R*f* = 0.38 (EtOAc: petroleum ether, 1:20 (v:v)).

NMR Spectroscopy:

^{1}**H NMR** (400 MHz, Chloroform-*d*, 298 K) δ 8.20 (d, *J* = 7.9 Hz, 1H), 7.77 (s, 1H), 7.62 (d, *J* = 8.3 Hz, 1H), 7.37 (t, *J* = 8.1 Hz, 1H), 7.31 - 7.23 (m, 3H), 6.91 (d, *J* = 6.0 Hz, 2H), 5.66 (s, 2H).

^{13}**C NMR** (101 MHz, Chloroform-*d*, 298 K) δ 141.17, 140.49, 136.39, 129.04, 127.92, 126.41 (d, *J* = 17.7 Hz), 125.30, 123.70, 120.64, 120.23 (qd, *J* = 288.8, 28.0 Hz, CF($\underline{C}F_3)_2$), 119.09, 117.73, 106.41, 93.01 - 89.87 (m, $\underline{C}F(CF_3)_2$), 49.93.

^{19}**F NMR** (376 MHz, Chloroform-*d*, 298 K) δ -75.02 (d, *J* = 9.0 Hz, CF(C$\underline{F}_3)_2$, 6F), -181.51 – -181.64 (m, C$\underline{F}(CF_3)_2$, 1F).

GC-MS: 420

HRMS (ESI) m/z $[M+H]^+$: Calculated for $C_{18}H_{12}F_7N_2O_2^+$: 421.0785. Found: 421.0790.

Melt Point: 89.0 - 90.4 °C

1-*iso*-Butyl-3-*iso*-perfluoropropyl-1*H*-indole (**23**)

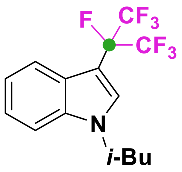

23

1-*iso*-Butyl-3-*iso*-perfluoropropyl-1*H*-indole (**23**) was prepared according to the general procedure using 1-*iso*-butyl-1*H*-indole (34.6 mg, 0.20 mmol). The obtained residue was purified by chromatography on silica gel eluting with EtOAc: petroleum ether (1: 50 - 1: 30 (v/v)) to afford 64.8 mg of **23** as a pale-yellow oil (94% yield).

R*f* = 0.65 (EtOAc: petroleum ether, 1:20 (v:v)).

NMR Spectroscopy:

^{1}**H NMR** (400 MHz, Chloroform-*d*, 298 K) δ 7.87 (d, *J* = 8.1 Hz, 1H), 7.53 - 7.43 (m, 2H), 7.37 - 7.31 (m, 1H), 7.29 - 7.23 (m, 1H), 4.50 (h, *J* = 6.8 Hz, 1H), 1.95 (td, *J* = 14.9, 7.0 Hz, 2H),

1.58 (d, J = 6.8 Hz, 3H), 0.89 (t, J = 7.0 Hz, 3H).

^{13}C NMR (101 MHz, Chloroform-*d*, 298 K) δ 136.43, 125.76, 124.43 (d, J = 11.6 Hz), 122.69, 121.37 (qd, J = 288.4, 28.6 Hz, CF(<u>C</u>F_3)$_2$), 121.15, 121.10, 110.29, 100.73 (d, J = 24.6 Hz), 94.06 - 91.02 (m, <u>C</u>F(CF_3)$_2$), 53.83, 29.91, 20.64, 10.64.

^{19}F NMR (376 MHz, Chloroform-*d*, 298 K) δ -76.20 (d, J = 9.7 Hz, CF(C<u>F</u>$_3$)$_2$, 6F), -178.17 – -177.96 (hept, J = 9.0 Hz, C<u>F</u>(CF_3)$_2$, 1F).

GC-MS: 341

HRMS (ESI) m/z [M+H]$^+$: Calculated for $C_{15}H_{15}F_7N^+$: 342.1093. Found: 342.1104.

1-Boc-3-*iso*-perfluoropropyl-1*H*-indole (**24**)

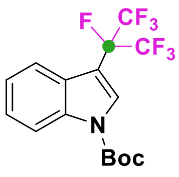

24

1-Boc-3-*iso*-perfluoropropyl-1*H*-indole (**24**) was prepared according to the general procedure using 1-Boc-1*H*-indole (43.4 mg, 0.20 mmol). The obtained residue was purified by chromatography on silica gel eluting with EtOAc: petroleum ether (1: 50 - 1: 30 (v/v)) to afford 63.1 mg of **24** as a colorless oil (82% yield).

R$_f$ = 0.56 (EtOAc: petroleum ether, 1:10 (v:v)).

NMR Spectroscopy:

^{1}H NMR (400 MHz, Chloroform-*d*, 298 K) δ 8.01 (d, J = 8.6 Hz, 1H), 7.64 (d, J = 7.8 Hz, 1H), 7.44 (t, J = 7.8 Hz, 1H), 7.30 (t, J = 7.5 Hz, 1H), 7.12 (s, 1H), 1.66 (s, 9H).

^{13}C NMR (101 MHz, Chloroform-*d*, 298 K) δ 149.43, 138.27, 126.98, 126.67, 123.33, 122.42 (d, J = 19.9 Hz), 121.84, 120.63 (qd, J = 287.0, 28.2 Hz, CF(<u>C</u>F_3)$_2$), 114.51, 113.40, 92.58 - 89.13 (m, <u>C</u>F(CF_3)$_2$), 85.76, 27.71.

^{19}F NMR (376 MHz, Chloroform-*d*, 298 K) δ -74.02 (d, J = 8.0 Hz, CF(C<u>F</u>$_3$)$_2$, 6F), -172.37 (s, C<u>F</u>(CF_3)$_2$, 1F).

GC-MS: 385

HRMS (ESI) m/z [M+H]$^+$: Calculated for $C_{16}H_{15}F_7NO_2^+$: 386.0989. Found: 386.0995.

1-Phenyl-3-*iso*-perfluoropropyl-1*H*-indole (**25**)

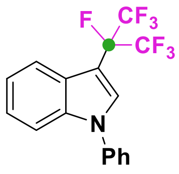

25

1-Phenyl-3-*iso*-perfluoropropyl-1*H*-indole (**25**) was prepared according to the general procedure using 1-Phenyl-1*H*-indole (38.6 mg, 0.20 mmol). The obtained residue was purified by chromatography on silica gel eluting with EtOAc: petroleum ether (1: 50 - 1: 30 (v/v)) to afford 67.8 mg of **25** as a yellow oil (94% yield).

R_f = 0.63 (EtOAc: petroleum ether, 1:10 (v:v)).

NMR Spectroscopy:

^{1}H NMR (400 MHz, Chloroform-*d*, 298 K) δ 7.89 (d, *J* = 7.7 Hz, 1H), 7.61 - 7.43 (m, 7H), 7.35 - 7.25 (m, 2H).

^{13}C NMR (101 MHz, Chloroform-*d*, 298 K) δ 138.52, 136.50, 129.96, 128.05, 127.95, 126.08 (d, *J* = 1.8 Hz), 125.13, 123.73, 121.98, 121.29 (d, *J* = 1.9 Hz), 121.25 (qd, *J* = 285.5, 28.3 Hz, CF($\underline{C}F_3)_2$), 111.17, 102.95 (d, *J* = 24.4 Hz), 94.10 – 90.35 (m, $\underline{C}F(CF_3)_2$)

^{19}F NMR (376 MHz, Chloroform-*d*, 298 K) δ -76.07 (d, *J* = 8.8 Hz, CF(C$\underline{F}_3)_2$, 6F), -178.75 (hept, *J* = 8.9 Hz C$\underline{F}(CF_3)_2$, 1F).

GC-MS: 361

HRMS (ESI) m/z $[M+H]^+$: Calculated for $C_{17}H_{11}F_7N^+$: 362.0780. Found: 362.0788.

1-Phenyl-3-*iso*-perfluoropropyl-pyrrole (**26**)

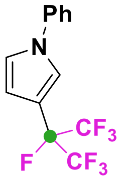

26

1-Phenyl-3-*iso*-perfluoropropyl-pyrrole (**26**) was prepared according to the general procedure using 1-phenylpyrrole (28.6 mg, 0.20 mmol). The obtained residue was purified by chromatography on silica gel eluting with EtOAc: petroleum ether (1: 50 - 1: 30 (v/v)) to afford 51.6 mg of **26** as a colorless oil (83% yield).

R_f = 0.48 (EtOAc: petroleum ether, 1:20 (v:v)).

NMR Spectroscopy:

^{1}H NMR (400 MHz, Chloroform-*d*, 298 K) δ 7.41 (dd, *J* = 5.2, 2.4 Hz, 3H), 7.35 - 7.29 (m, 2H), 6.82 (d, *J* = 4.9 Hz, 1H), 6.64 (s, 1H), 6.33 (d, *J* = 2.5 Hz, 1H).

^{13}C NMR (101 MHz, Chloroform-*d*, 298 K) δ 140.63, 128.91, 128.58, 128.54, 127.50, 120.43 (qd, *J* = 289.3, 29.0 Hz, CF($\underline{C}F_3)_2$), 116.48 (d, *J* = 17.9 Hz), 113.14, 108.96, 92.61 - 89.23 (m, $\underline{C}F(CF_3)_2$).

^{19}F NMR (376 MHz, Chloroform-*d*, 298 K) δ -76.29 (d, *J* = 9.1 Hz, CF(C$\underline{F}_3)_2$, 6F), -179.03 (hept, *J* = 8.9 Hz, C$\underline{F}(CF_3)_2$, 1F).

GC-MS: 311

HRMS (ESI) m/z $[M+H]^+$: Calculated for $C_{13}H_9F_7N^+$: 312.0622. Found: 312.0633.

2,5-Dimethyl-1-phenyl-3-*iso*-perfluoropropyl-pyrrole (**27**)

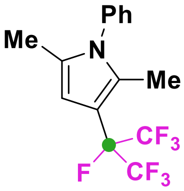

27

2,5-Dimethyl-1-phenyl-3-*iso*-perfluoropropyl-pyrrole (**27**) was prepared according to the general procedure using 2,5-dimethyl-1-phenyl-pyrrole (34.2 mg, 0.20 mmol). The obtained residue was purified by chromatography on silica gel eluting with EtOAc: petroleum ether (1: 50 - 1: 30 (v/v)) to afford 61.7 mg of **27** as a colorless oil (91% yield).

R_f = 0.48 (EtOAc: petroleum ether, 1:20 (v:v)).

NMR Spectroscopy:

^{1}H NMR (400 MHz, Chloroform-*d*, 298 K) δ 7.53 - 7.39 (m, 3H), 7.24 - 7.16 (m, 2H), 6.02 (s, 1H), 2.07 (d, *J* = 2.6 Hz, 3H), 1.97 (s, 3H).

^{13}C NMR (101 MHz, Chloroform-*d*, 298 K) δ 137.82, 130.14, 129.41, 129.27, 128.61, 128.57, 121.36 (qd, *J* = 288.3, 28.5 Hz, CF($\underline{C}F_3$)$_2$), 104.77, 104.24 (d, *J* = 20.3 Hz), 93.86 - 90.79 (m, $\underline{C}$F(CF_3)$_2$), 12.81, 12.03 (d, *J* = 6.9 Hz).

^{19}F NMR (376 MHz, Chloroform-*d*, 298 K) δ -76.41 (d, *J* = 8.4 Hz, CF(C$\underline{F}_3$)$_2$, 6F), -178.90 – -179.06 (m, C$\underline{F}$(CF_3)$_2$, 1F).

GC-MS: 339

HRMS (ESI) m/z $[M+H]^+$: Calculated for $C_{15}H_{13}F_7N^+$: 340.0935. Found: 340.0927.

9-Ethyl-3-*iso*-perfluoropropyl-carbazole (**28-1**) and 9-ethyl-4-*iso*-perfluoropropyl-carbazole (**28-2**)

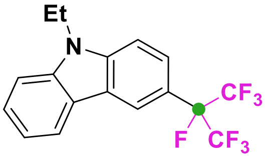

28-1

9-Ethyl-3-*iso*-perfluoropropyl-carbazole (**28-1**) was prepared according to the general procedure using 9-ethylcarbazole (39.0 mg, 0.20 mmol). The obtained residue was purified by chromatography on silica gel eluting with EtOAc: petroleum ether (1: 50 - 1: 30 (v/v)) to afford 36.2 mg of **28-1** as a pale-yellow solid (50% yield).

R_f = 0.44 (EtOAc: petroleum ether, 1:20 (v:v)).

NMR Spectroscopy:

^{1}**H NMR** (400 MHz, Chloroform-*d*, 298 K) δ 8.35 (s, 1H), 8.15 (d, *J* = 7.8 Hz, 1H), 7.68 (d, *J* = 8.7 Hz, 1H), 7.49 (m, 3H), 7.34 - 7.25 (m, 1H), 4.38 (q, *J* = 7.3 Hz, 2H), 1.46 (t, *J* = 7.2 Hz, 3H).

^{13}**C NMR** (101 MHz, Chloroform-*d*, 298 K) δ 140.98, 140.59, 126.74, 123.07, 122.69, 122.58, 121.09 (qd, *J* = 287.8, 28.2 Hz, CF(<u>*C*</u>$F_3)_2$), 120.75, 119.78, 118.41 (d, *J* = 11.8 Hz), 116.68 (d, *J* = 20.5 Hz), 108.95, 108.77, 93.77 - 90.49 (m, <u>*C*</u>F($CF_3)_2$), 37.84, 13.87.

^{19}**F NMR** (376 MHz, Chloroform-*d*, 298 K) δ -75.53 (d, *J* = 7.4 Hz, CF(C<u>*F*</u>$_3)_2$, 6F), -180.12 (hept, J = 7.3 Hz, C<u>*F*</u>($CF_3)_2$, 1F).

GC-MS: 363

HRMS (ESI) m/z $[M+H]^+$: Calculated for $C_{17}H_{13}F_7N^+$: 364.0936. Found: 364.0933.

Melt Point: 103.1 - 104.5 °C

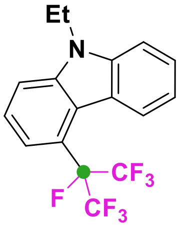

28-2

9-Ethyl-4-*iso*-perfluoropropyl-carbazole (**28-2**) was prepared according to the general procedure using 9-ethylcarbazole (39.0 mg, 0.20 mmol). The obtained residue was purified by chromatography on silica gel eluting with EtOAc: petroleum ether (1: 50 - 1: 30 (v/v)) to afford 32.2 mg of **28-2** as a white solid (44% yield).

R*f* = 0.47 (EtOAc: petroleum ether, 1:20 (v:v)).

NMR Spectroscopy:

^{1}**H NMR** (400 MHz, Chloroform-*d*, 298 K) δ 8.41 (dd, *J* = 8.5, 3.7 Hz, 1H), 7.61 (d, *J* = 8.0 Hz, 1H), 7.56 - 7.50 (m, 2H), 7.49 - 7.43 (m, 2H), 7.36 - 7.23 (m, 1H), 4.41 (q, *J* = 7.3 Hz, 2H), 1.44 (t, *J* = 7.2 Hz, 3H).

^{13}**C NMR** (101 MHz, Chloroform-*d*, 298 K) δ 141.20, 140.58, 126.68, 126.13, 125.84, 124.73, 121.52 (qd, *J* = 289.8, 28.5 Hz, CF(<u>*C*</u>$F_3)_2$), 120.92 (d, *J* = 23.7 Hz), 120.62, 119.75 (d, *J* = 4.6 Hz), 117.56, 111.72, 108.54, 95.89 - 92.77 (m, <u>*C*</u>F($CF_3)_2$), 37.54, 13.55.

^{19}**F NMR** (376 MHz, Chloroform-*d*, 298 K) δ -73.60 (d, *J* = 7.6 Hz, CF(C<u>*F*</u>$_3)_2$, 6F), -172.73 (hept, J = 8.4 Hz, C<u>*F*</u>($CF_3)_2$, 1F).

GC-MS: 363

HRMS (ESI) m/z $[M+H]^+$: Calculated for $C_{17}H_{13}F_7N^+$: 364.0936. Found: 364.0928.

Melt Point: 100.9 - 102.6 °C

5-Bromo-3-*iso*-perfluoropropyl-benzo[b]thiophene (**29**)

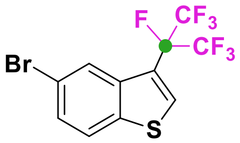

29

5-Bromo-3-*iso*-perfluoropropyl-benzo[b]thiophene (**29**) was prepared according to the general procedure using 5-bromobenzo[b]thiophene (42.6 mg, 0.20 mmol). The obtained residue was purified by chromatography on silica gel eluting with EtOAc: petroleum ether (1: 50 - 1: 30 (v/v)) to afford 61.6 mg of **29** as a white solid (81% yield).

R_f = 0.58 (EtOAc: petroleum ether, 1:20 (v:v)).

NMR Spectroscopy:

^{1}**H NMR** (400 MHz, Chloroform-*d*, 298 K) δ 8.00 (d, *J* = 1.9 Hz, 1H), 7.74 (d, *J* = 8.7 Hz, 1H), 7.58 - 7.51 (m, 2H).

^{13}**C NMR** (101 MHz, Chloroform-*d*, 298 K) δ 140.04, 138.57, 129.83 (d, *J* = 23.6 Hz), 129.50, 127.43, 125.09 (d, *J* = 7.1 Hz), 123.61, 120.15 (qd, *J* = 288.8, 27.6 Hz, CF($\underline{C}F_3)_2$), 119.34, 92.26 - 89.54 (m, $\underline{C}F(CF_3)_2$).

^{19}**F NMR** (376 MHz, Chloroform-*d*, 298 K) δ -76.24 (d, *J* = 8.1 Hz, CF(C$\underline{F}_3)_2$, 6F), -172.38 (hept, J = 8.3 Hz, C$\underline{F}(CF_3)_2$, 1F).

GC-MS: 380

HRMS (ESI) m/z $[M+H]^+$: Calculated for $C_{11}H_5BrF_7S^+$: 380.9182. Found: 380.9178.

Melt Point: 61.7 - 63.6 °C

5-*tert*-Butyl-3-*iso*-perfluoropropyl-benzo[b]thiophene (**30**)

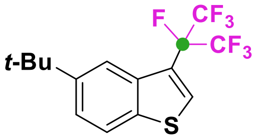

30

5-*tert*-Butyl-3-*iso*-perfluoropropyl-benzo[b]thiophene (**30**) was prepared according to the general procedure using 5-*tert*-butyl-benzo[b]thiophene (38.0 mg, 0.20 mmol). The obtained residue was purified by chromatography on silica gel eluting with EtOAc: petroleum ether (1: 50 - 1: 30 (v/v)) to afford 61.6 mg of **30** as a colorless oil (86% yield).

R_f = 0.55 (EtOAc: petroleum ether, 1:20 (v:v)).

NMR Spectroscopy:

^{1}**H NMR** (400 MHz, Chloroform-*d*, 298 K) δ 7.87 (s, 1H), 7.82 (d, *J* = 8.7 Hz, 1H), 7.64 (s, 1H), 7.55 (d, *J* = 8.7 Hz, 1H), 1.41 (s, 9H).

^{13}**C NMR** (101 MHz, Chloroform-*d*, 298 K) δ 148.74, 138.77, 137.29, 127.85 (d, *J* = 23.6 Hz), 126.22 (d, *J* = 6.9 Hz), 124.87, 121.82, 120.83, 120.33 (qd, *J* = 288.8, 27.6 Hz, CF($\underline{C}F_3)_2$),

92.85 - 89.79 (m, CF(CF_3)$_2$), 34.88, 31.52.

^{19}F NMR (376 MHz, Chloroform-*d*, 298 K) δ -76.32 (d, *J* = 8.1 Hz, CF(C*F*$_3$)$_2$, 6F), -172.20 (hept, J = 7.9 Hz, C*F*(CF_3)$_2$, 1F).

GC-MS: 358

HRMS (ESI) m/z [M+H]$^+$: Calculated for $C_{15}H_{14}F_7S^+$: 359.0704. Found: 359.0699.

2-*iso*-Perfluoropropylthianthrene (**31**)

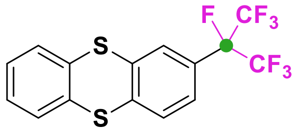

31

2-*iso*-Perfluoropropylthianthrene (**31**) was prepared according to the general procedure using 5-thianthrene (43.2 mg, 0.20 mmol). The obtained residue was purified by chromatography on silica gel eluting with EtOAc: petroleum ether (1: 50 - 1: 30 (v/v)) to afford 71.4 mg of **31** as a white solid (93% yield).

R*f* = 0.55 (EtOAc: petroleum ether, 1:20 (v:v)).

NMR Spectroscopy:

^{1}H NMR (400 MHz, Chloroform-*d*, 298 K) δ 7.72 (s, 1H), 7.59 (d, *J* = 8.3 Hz, 1H), 7.51 - 7.44 (m, 3H), 7.31 - 7.23 (m, 2H).

^{13}C NMR (101 MHz, Chloroform-*d*, 298 K) δ 139.85, 137.11, 134.57, 134.50, 129.01, 128.92, 128.27, 126.45 (d, *J* = 20.9 Hz), 125.86, 125.75, 124.94, 124.84, 120.50 (qd, *J* = 288.8, 27.6 Hz, CF(*C*F$_3$)$_2$), 92.72 - 89.79 (m, *C*F(CF_3)$_2$).

^{19}F NMR (376 MHz, Chloroform-*d*, 298 K) δ -75.49 (d, *J* = 8.1 Hz, CF(C*F*$_3$)$_2$, 6F), -182.05 (hept, J = 7.3 Hz, C*F*(CF_3)$_2$, 1F).

GC-MS: 384

HRMS (ESI) m/z [M+H]$^+$: Calculated for $C_{15}H_8F_7S_2^+$: 384.9955. Found: 384.9958.

Melt Point: 56.8 - 57.9 °C

2-Ethy-3-*iso*-perfluoropropylbenzofuran and 2-ethy-5-*iso*-perfluoropropylbenzofuran (**32**)

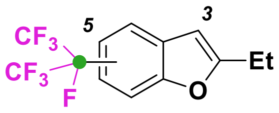

32

2-Ethy-3-*iso*-perfluoropropylbenzofuran and 2-ethy-5-*iso*-perfluoropropylbenzofuran (**32**) was prepared according to the general procedure using 2-ethybenzofuran (29.2 mg, 0.20 mmol). The obtained residue was purified by chromatography on silica gel eluting with EtOAc: petroleum ether (1: 50 - 1: 30 (v/v)) to afford 50.2 mg of **32** as a pale-yellow oil (80% yield).

R$_f$ = 0.57 (EtOAc: petroleum ether, 1:20 (v:v)).

NMR Spectroscopy:

^{1}**H NMR** (400 MHz, Chloroform-*d*, 298 K) δ 7.68 (s, 1H), 7.57 (d, *J* = 4.6 Hz), 7.55 (d, *J* = 3.5 Hz), 7.44 - 7.35 (m), 7.28 (m), 6.61 (d, *J* = 4.9 Hz), 6.43 (s), 2.82 (m), 1.35 (m).

^{13}**C NMR** (101 MHz, Chloroform-*d*, 298 K) δ 163.83, 162.65, 155.32, 154.29, 131.61, 127.72, 123.55, 122.98, 120.79 (qd, *J* = 288.3, 27.6 Hz, CF(<u>C</u>F$_3$)$_2$), 120.64, 120.31, 119.69 (d, *J* = 10.2 Hz), 118.36 (d, *J* = 21.9 Hz), 113.60, 108.86 (d, *J* = 12.7 Hz), 101.24 (d, *J* = 12.0 Hz), 101.08, 94.82 - 91.42 (m, <u>C</u>F(CF$_3$)$_2$), 21.93, 21.82, 11.77, 11.69.

^{19}**F NMR** (376 MHz, Chloroform-*d*, 298 K) δ -75.10 (d, *J* = 7.5 Hz, CF(C<u>F</u>$_3$)$_2$), -75.62 (d, *J* = 7.2 Hz, CF(C<u>F</u>$_3$)$_2$), -179.01 – -179.81 (m, C<u>F</u>(CF$_3$)$_2$), -180.41 – -180.63 (m, C<u>F</u>(CF$_3$)$_2$).

GC-MS: 314

HRMS (ESI) m/z [M+H]$^+$: Calculated for $C_{13}H_{10}F_7O^+$: 315.0620. Found: 315.0620.

7-Bromo-5-*iso*-perfluoropropylbenzofuran (**33**)

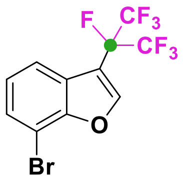

33

7-Bromo-5-*iso*-perfluoropropylbenzofuran (**33**) was prepared according to the general procedure using 7-bromobenzofuran (39.2 mg, 0.20 mmol). The obtained residue was purified by chromatography on silica gel eluting with EtOAc: petroleum ether (1: 50 - 1: 30 (v/v)) to afford 64.1 mg of **33** as a pale-yellow oil (88% yield).

R$_f$ = 0.53 (EtOAc: petroleum ether, 1:20 (v:v)).

NMR Spectroscopy:

^{1}**H NMR** (400 MHz, Chloroform-*d*, 298 K) δ 7.63 - 7.56 (m, 2H), 7.27 (s, 1H), 7.21 (m, 1H).

^{13}**C NMR** (101 MHz, Chloroform-*d*, 298 K) δ 153.02, 142.28 (d, *J* = 27.5 Hz), 129.92, 127.53, 125.35, 121.34, 119.88 (qd, *J* = 289.3, 27.1 Hz, CF(<u>C</u>F$_3$)$_2$), 111.12, 104.78, 90.28 - 86.86 (m, <u>C</u>F(CF$_3$)$_2$).

^{19}**F NMR** (376 MHz, Chloroform-*d*, 298 K) δ -75.24 (d, *J* = 9.0 Hz, CF(C<u>F</u>$_3$)$_2$, 6F), -179.58 (hept, *J* = 8.9 Hz, C<u>F</u>(CF$_3$)$_2$, 1F).

GC-MS: 364

HRMS (ESI) m/z [M+H]$^+$: Calculated for $C_{11}H_5BrF_7O^+$: 364.9411. Found: 364.9401.

2-*iso*-perfluoropropyl-zolmitriptan (**34**)

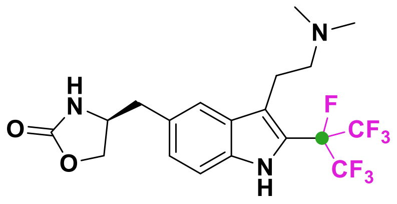

34

2-*iso*-perfluoropropyl-zolmitriptan (**34**) was prepared according to the general procedure using zolmitriptan (57.4 mg, 0.20 mmol). The obtained residue was purified by chromatography on silica gel eluting with MeOH: DCM (1: 40 - 1: 15 (v/v)) to afford 77.4 mg of **34** as an off-white solid (85% yield).

R_f = 0.46 (MeOH: DCM, 1:10 (v:v)).

NMR Spectroscopy:

^{1}H NMR (400 MHz, Acetonitrile-d_3, 298 K) δ 9.94 (br, s, 1H), 7.65 (s, 1H), 7.43 (d, J = 8.5 Hz, 1H), 7.13 (d, J = 8.5 Hz, 1H), 6.71 (m, 1H), 4.29 (t, J = 8.1 Hz, 1H), 4.16 - 4.10 (m, 1H), 4.09 - 4.03 (m, 1H), 3.14 (t, J = 8.5 Hz, 2H), 2.97 (q, J = 7.2 Hz, 2H), 2.87 (dd, J = 10.5, 5.5 Hz, 1H), 2.46 (s, 6H).

^{13}C NMR (101 MHz, Acetonitrile-d_3, 298 K) δ 159.51, 136.13, 129.30, 128.39, 127.62, 126.52, 122.06, 121.01 (qd, J = 288.2, 27.2 Hz, CF($\underline{C}F_3)_2$), 120.26, 112.12, 93.11 - 89.10 (m, $\underline{C}F(CF_3)_2$), 68.68, 58.84, 53.27, 45.95, 40.08, 21.35 (d, J = 6.6 Hz).

^{19}F NMR (376 MHz, Acetonitrile-d_3, 298 K) δ -76.73 (d, J = 8.7 Hz, CF(C$\underline{F}_3)_2$, 6F), -184.39 (hept, J = 7.8 Hz, C$\underline{F}(CF_3)_2$, 1F).

GC-MS: 455

HRMS (ESI) m/z [M+H]$^+$: Calculated for $C_{19}H_{21}F_7N_3O_2^+$: 456.1520. Found: 456.1523.

Melt Point: 127.7 - 129.8 °C

N-Acetyl-2-*iso*-perfluoropropyl-tryptophan methyl ester (**35**)

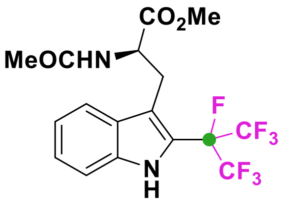

35

N-Acetyl-2-*iso*-perfluoropropyl-tryptophan methyl ester (**35**) was prepared according to the general procedure using *N*-acetyl-tryptophan methyl ester (52.0 mg, 0.20 mmol). The obtained residue was purified by chromatography on silica gel eluting with EtOAc: petroleum ether (1: 5 - 1: 20 (v/v)) to afford 76.2 mg of **35** as a white solid (89% yield).

R_f = 0.25 (EtOAc: petroleum ether, 1:5 (v:v)).

NMR Spectroscopy:

^{1}**H NMR** (400 MHz, Chloroform-*d*, 298 K) δ 9.58 (br, s, 1H), 7.72 (d, *J* = 8.1 Hz, 1H), 7.38 (d, *J* = 7.8 Hz, 1H), 7.29 - 7.21 (m, 1H), 7.14 m, 1H), 6.47 (d, *J* = 7.5 Hz, 1H), 4.96 (q, *J* = 7.1 Hz, 1H), 3.59 (s, 3H), 3.50 - 3.33 (m, 2H), 1.88 (s, 3H).

^{13}**C NMR** (101 MHz, Chloroform-*d*, 298 K) δ 172.74, 170.41, 136.95, 127.60, 124.69, 120.75, 120.41 (qd, *J* = 289.3, 28.1 Hz, CF(<u>*C*</u>$F_3)_2$), 119.38, 117.84 (d, *J* = 27.0 Hz), 114.81, 112.18, 93.22 - 89.70 (m, <u>*C*</u>F$(CF_3)_2$), 60.61, 53.21, 52.37, 27.40 (d, *J* = 7.3 Hz), 22.71.

^{19}**F NMR** (376 MHz, Chloroform-*d*, 298 K) δ -75.85 (d, *J* = 8.0 Hz, CF(C<u>*F*</u>$_3)_2$, 6F), -183.82 (hept, *J* = 9.1 Hz, C<u>*F*</u>$(CF_3)_2$, 1F).

GC-MS: 428

HRMS (ESI) m/z $[M+H]^+$: Calculated for $C_{17}H_{16}F_7N_2O_3^+$: 429.1047. Found: 429.1050.

Melt Point: 114.8 - 117.0 °C

5-Methoxy-2-*iso*-perfluoropropyl-*N*-acetyl-tryptamine (**36**)

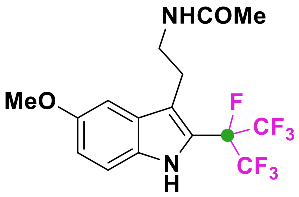

36

5-Methoxy-2-*iso*-perfluoropropyl-*N*-acetyl-tryptamine (**36**) was prepared according to the general procedure using 5-methoxy-*N*-acetyl-tryptamine (46.4 mg, 0.20 mmol). The obtained residue was purified by chromatography on silica gel eluting with EtOAc: petroleum ether (1: 5 - 1: 30 (v/v)) to afford 60.8 mg of **36** as a pale-yellow solid (76% yield).

R_f = 0.21 (EtOAc: petroleum ether, 1:5 (v:v)).

NMR Spectroscopy:

^{1}**H NMR** (400 MHz, Acetonitrile-d_3, 298 K) δ 9.72 (br, s, 1H), 7.36 (d, *J* = 8.9 Hz, 1H), 7.21 (d, *J* = 2.4 Hz, 1H), 6.91 (dd, *J* = 8.9, 2.4 Hz, 1H), 6.65 (s, 1H), 3.79 (s, 3H), 3.35 (q, *J* = 7.3, 6.0 Hz, 2H), 3.01 (td, *J* = 7.4, 2.2 Hz, 2H), 1.79 (s, 3H).

^{13}**C NMR** (101 MHz, Acetonitrile-d_3, 298 K) δ 170.14, 154.66, 132.18, 128.28, 120.54 (qd, *J* = 288.8, 27.6 Hz, CF(<u>*C*</u>$F_3)_2$), 117.12, 116.88 (d, J = 26.4 Hz), 115.32, 112.85, 100.52, 93.25 - 89.52 (m, <u>*C*</u>F$(CF_3)_2$), 55.28, 39.86, 24.38 (d, J = 6.7 Hz), 22.15.

^{19}**F NMR** (376 MHz, Acetonitrile-d_3, 298 K) δ -76.73 (d, *J* = 8.7 Hz, CF(C<u>*F*</u>$_3)_2$, 6F), -184.39 (hept, *J* = 8.0 Hz, C<u>*F*</u>$(CF_3)_2$, 1F).

GC-MS: 400

HRMS (ESI) m/z $[M+H]^+$: Calculated for $C_{16}H_{16}F_7N_2O_2^+$: 401.1100. Found: 401.1095.

Melt Point: 159.5 - 161.4 °C

9-Methoxy-3-*iso*-perfluoropropyl-7H-furo[3,2-g][1]benzopyran-7-one (**37**)

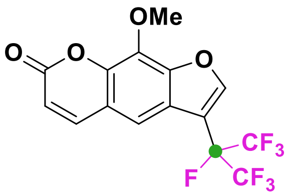

37

9-Methoxy-3-*iso*-perfluoropropyl-7H-furo[3,2-g][1]benzopyran-7-one (**37**) was prepared according to the general procedure using 9-methoxy-7H-furo[3,2-g][1]benzopyran-7-one (43.2 mg, 0.20 mmol). The obtained residue was purified by chromatography on silica gel eluting with EtOAc: petroleum ether (1: 20 - 1: 50 (v/v)) to afford 72.9 mg of **37** as a white solid (95% yield).

R_f = 0.53 (EtOAc: petroleum ether, 1:10 (v:v)).

NMR Spectroscopy:

^{1}H NMR (400 MHz, Chloroform-*d*, 298 K) δ 7.77 (d, *J* = 9.6 Hz, 1H), 7.41 (s, 1H), 7.25 (s, 1H), 6.42 (d, *J* = 9.6 Hz, 1H), 4.30 (s, 3H).

^{13}C NMR (101 MHz, Chloroform-*d*, 298 K) δ 159.98, 147.67, 143.90, 143.23 (d, *J* = 28.2 Hz), 133.12, 124.87, 119.80 (qd, *J* = 289.8, 27.1 Hz, CF($\underline{C}F_3)_2$), 117.67, 115.92, 113.59, 110.66, 90.04 - 86.94 (m, $\underline{C}F(CF_3)_2$), 61.20.

^{19}F NMR (376 MHz, Chloroform-*d*, 298 K) δ -75.22 (d, *J* = 8.9 Hz, CF($C\underline{F}_3)_2$, 6F), -179.38 (hept, *J* = 9.3 Hz, $C\underline{F}(CF_3)_2$, 1F).

GC-MS: 384

HRMS (ESI) m/z $[M+H]^+$: Calculated for $C_{15}H_8F_7O_4^+$: 385.0310. Found: 385.0317.

Melt Point: 120.4 - 121.9 °C

The photocatalytic isoperfluoropropylation of non-activated arenes

Optimization of reaction conditions

Photochemistry parameters screening

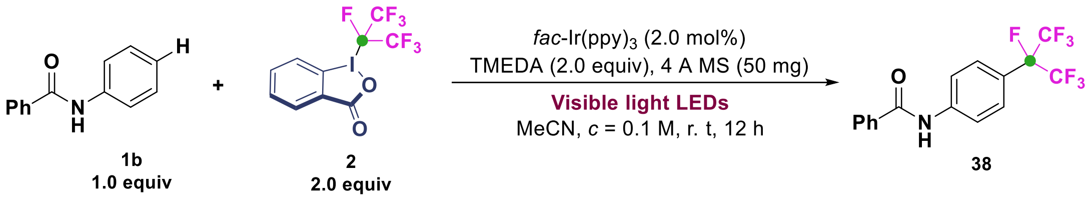

Under an ambient atmosphere, in a 25 mL screw-cap vial equipped with a magnetic stirring bar, benzanilide (**1b**, 19.7 mg, 0.10 mmol, 1.0 equiv.), *i*-C_3F_7-iodine(**III**) (83.2 mg, 0.20 mmol, 2.0 equiv), *fac*-Ir(ppy)$_3$ (1.3 mg, 2.0 mol%), were dissolved in dry MeCN (1 mL). Subsequently, 4 A molecular sieves (50 mg) and TMEDA (29.9 μL, 0.20 mmol, 2.0 equiv) was added into the vial. The vial was sealed and the reaction was irradiated by LED lamps with fixed wavelength and power for 12 h, the

temperature of the reaction systems was controlled within 25 - 35 °C by using the drum fan. After this, 10 μL of *n*-dodecane was added as an internal standard and the reaction mixture was diluted with EtOAc. The crude reaction mixture was filtered through a plug of silica then subjected to GC-MS analysis.

Table S3. Photochemistry parameters screening

Entry	LEDs (Wavelength, Power)	GC yield [%]
**1**	Blue LEDs (450 nm, 25 W)	77
**2**	Blue LEDs (450 nm, 10 W)	54
**3**	Blue LEDs (460 nm, 25 W)	74
**4**	Green LEDs (520 nm, 10 W)	28
**5**	White LEDs (400 - 760 nm, 100W)	63

Photocatalysts screening

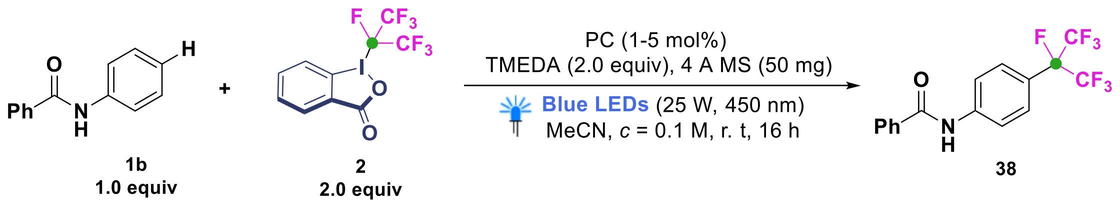

Under an ambient atmosphere, in a 25 mL screw-cap vial equipped with a magnetic stirring bar, benzanilide (**1b**, 19.7 mg, 0.10 mmol, 1.0 equiv.), *i*-C_3F_7-iodine(III) (83.2 mg, 0.20 mmol, 2.0 equiv), photocatalysts (PC), were dissolved in dry MeCN (1 mL). Subsequently, 4 A molecular sieves (50 mg) and TMEDA (29.9 μL, 0.20 mmol, 2.0 equiv) was added into the vial. The vial was sealed and the reaction was irradiated at 450 nm for 16 h, the temperature of the reaction systems was controlled within 25 - 35 °C by using the drum fan. After this, 10 μL of *n*-dodecane was added as an internal standard and the reaction mixture was diluted with EtOAc. The crude reaction mixture was filtered through a plug of silica then subjected to GC-MS analysis.

Table S4. Photocatalysts screening

Entry	PC	GC yield [%]
**1**	[Ir(dtbbpy)(ppy)$_2$]PF$_6$ (1 mol%)	93
**2**	*fac*-Ir(ppy)$_3$ (1 mol%)	79
**3**	PTH (5 mol%)	53
**4**	Eosin Y (5 mol%)	18

**5**	Thiaxanthone (5 mol%)	trace

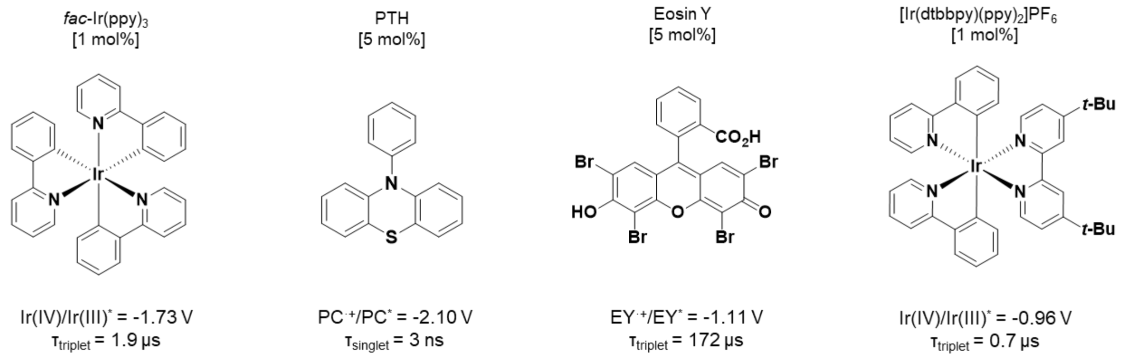

Base screening

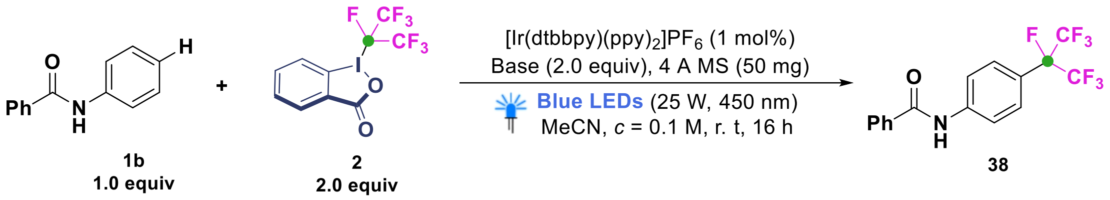

Under an ambient atmosphere, in a 25 mL screw-cap vial equipped with a magnetic stirring bar, benzanilide (**1b**, 19.7 mg, 0.10 mmol, 1.0 equiv.), *i*-C_3F_7-iodine(**III**) (83.2 mg, 0.20 mmol, 2.0 equiv), [Ir(dtbbpy)(ppy)$_2$]PF_6 (0.9 mg, 1 mol%), were dissolved in dry MeCN (1 mL). Subsequently, 4 A molecular sieves (50 mg) and base (0.20 mmol, 2.0 equiv) was added into the vial. The vial was sealed and the reaction was irradiated at 450 nm for 16 h, the temperature of the reaction systems was controlled within 25 - 35 °C by using the drum fan. After this, 10 μL of *n*-dodecane was added as an internal standard and the reaction mixture was diluted with EtOAc. The crude reaction mixture was filtered through a plug of silica then subjected to GC-MS analysis.

Table S5. Base screening

Entry	Base	GC yield [%]
**1**	TMEDA	93
**2**	TMP	82
**3**	DIPEA	47
**4**	DBU	15

Control experiments

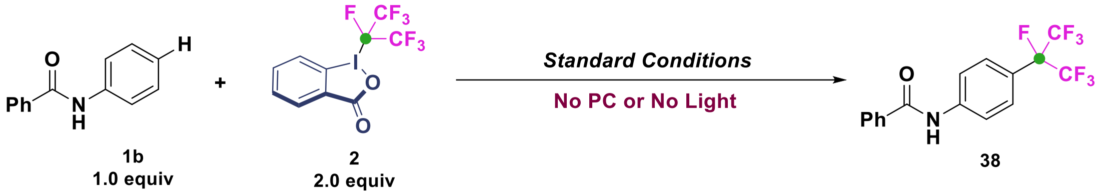

Table S6. Control experiments

Entry	Conditions	GC yield [%]
**1**	No PC	6
**2**	No Light	trace

Sensitivity screening

Temperature sensitivity

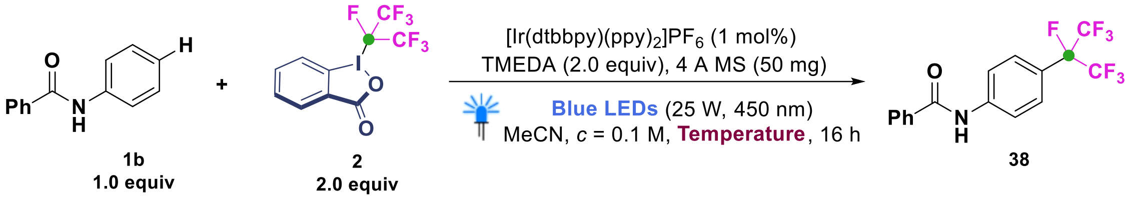

Under an ambient atmosphere, in a 25 mL screw-cap vial equipped with a magnetic stirring bar, benzanilide (**1b**, 19.7 mg, 0.10 mmol, 1.0 equiv.), *i*-C_3F_7-iodine(III) (83.2 mg, 0.20 mmol, 2.0 equiv), $[Ir(dtbbpy)(ppy)_2]PF_6$ (0.9 mg, 1 mol%), were dissolved in dry MeCN (1 mL). Subsequently, 4 A molecular sieves (50 mg) and TMEDA (29.9 µL, 0.20 mmol, 2.0 equiv) was added into the vial. The vial was sealed and the reaction was irradiated at 450 nm for 16 h, the temperature of the reaction systems was controlled by using thermostatic isopropanolbath. After this, 10 µL of *n*-dodecane was added as an internal standard and the reaction mixture was diluted with EtOAc. The crude reaction mixture was filtered through a plug of silica then subjected to GC-MS analysis.

Table S7. Temperature sensitivity

Entry	Temperature [°C]	GC yield [%]	Difference [%]
**1**	-40 °C	76	18
**2**	70 °C	65	30

Concentration sensitivity

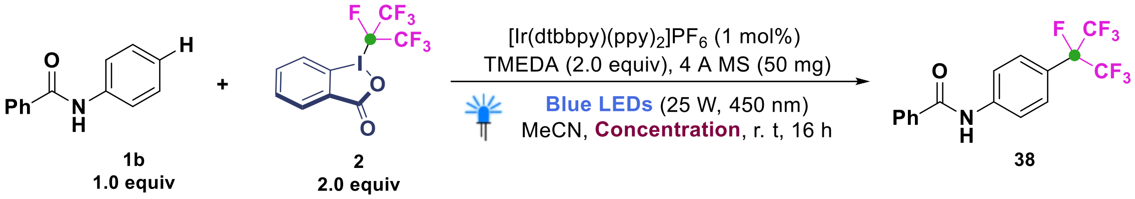

Under an ambient atmosphere, in a 25 mL screw-cap vial equipped with a magnetic stirring bar, benzanilide (**1b**, 19.7 mg, 0.10 mmol, 1.0 equiv.), *i*-C_3F_7-iodine(III) (83.2 mg, 0.20 mmol, 2.0 equiv), [Ir(dtbbpy)(ppy)$_2$]PF_6 (0.9 mg, 1 mol%), were dissolved in dry MeCN (0.5 - 4.0 mL). Subsequently, 4 A molecular sieves (50 mg) and TMEDA (29.9 μL, 0.20 mmol, 2.0 equiv) was added into the vial. The vial was sealed and the reaction was irradiated at 450 nm for 16 h, the temperature of the reaction systems was controlled within 25 - 35 °C by using the drum fan. After this, 10 μL of *n*-dodecane was added as an internal standard and the reaction mixture was diluted with EtOAc. The crude reaction mixture was filtered through a plug of silica then subjected to GC-MS analysis.

Table S8. Concentration sensitivity

Entry	Concentration [M]	GC yield [%]	Difference [%]
**1**	0.025	89	4
**2**	0.2	90	3

Water sensitivity

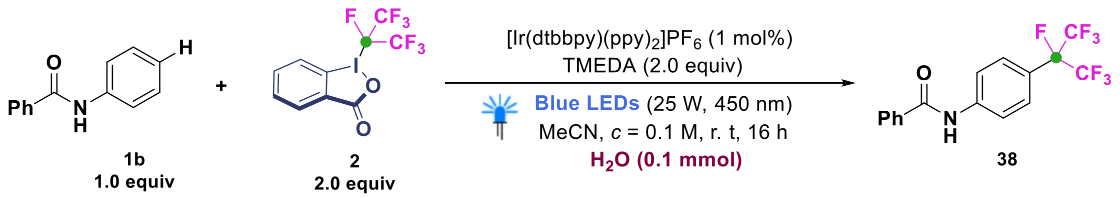

Under an ambient atmosphere, in a 25 mL screw-cap vial equipped with a magnetic stirring bar, benzanilide (**1b**, 19.7 mg, 0.10 mmol, 1.0 equiv.), *i*-C_3F_7-iodine(III) (83.2 mg, 0.20 mmol, 2.0 equiv), [Ir(dtbbpy)(ppy)$_2$]PF_6 (0.9 mg, 1 mol%), were dissolved in dry MeCN (1 mL). Subsequently, TMEDA (29.9 μL, 0.20 mmol, 2.0 equiv) and water (1.8 μL, 0.10 mmol, 1.0 equiv) was added into the vial. The vial was sealed and the reaction was irradiated at 450 nm for 16 h, the temperature of the reaction systems was controlled within 25 - 35 °C by using the drum fan. After this, 10 μL of *n*-dodecane was added as an internal standard and the reaction mixture was diluted with EtOAc. The crude reaction mixture was filtered through a plug of silica then subjected to GC-MS analysis.

Oxygen sensitivity

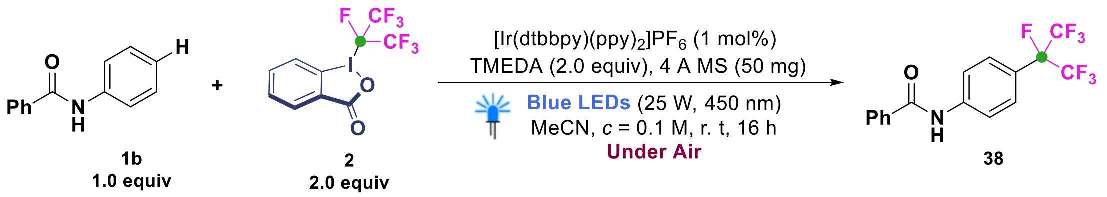

Under an ambient atmosphere, in a 25 mL screw-cap vial equipped with a magnetic stirring bar,

benzanilide (**1b**, 19.7 mg, 0.10 mmol, 1.0 equiv.), *i*-C_3F_7-iodine(III) (83.2 mg, 0.20 mmol, 2.0 equiv), [Ir(dtbbpy)(ppy)$_2$]PF_6 (0.9 mg, 1 mol%), were dissolved in dry MeCN (1 mL). Subsequently, 4 A molecular sieves (50 mg) and TMEDA (29.9 μL, 0.20 mmol, 2.0 equiv) was added into the vial. The vial was exposed to air and the reaction was irradiated at 450 nm for 16 h (avoid excessive solvent volatilization), the temperature of the reaction systems was controlled within 25 - 35 °C by using the drum fan. After this, 10 μL of *n*-dodecane was added as an internal standard and the reaction mixture was diluted with EtOAc. The crude reaction mixture was filtered through a plug of silica then subjected to GC-MS analysis.

Big scale sensitivity

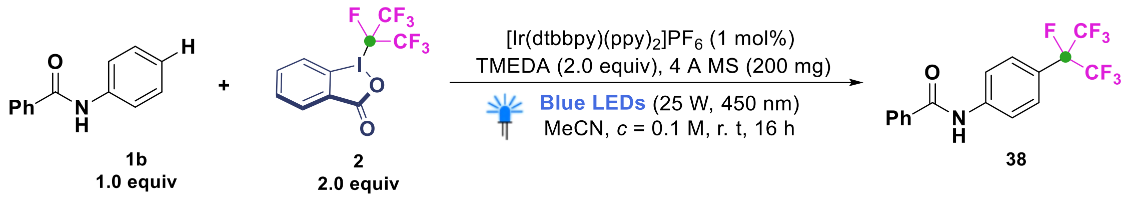

Under an ambient atmosphere, in a 25 mL screw-cap vial equipped with a magnetic stirring bar, benzanilide (**1b**, 98.5 mg, 0.50 mmol, 1.0 equiv.), *i*-C_3F_7-iodine(III) (416.0 mg, 1.0 mmol, 2.0 equiv), [Ir(dtbbpy)(ppy)$_2$]PF_6 (4.6 mg, 1.0 mol%), were dissolved in dry MeCN (5 mL). Subsequently, 4 A molecular sieves (200 mg) and TMEDA (149.5 μL, 1.0 mmol, 2.0 equiv) was added into the vial. The vial was exposed to air and the reaction was irradiated at 450 nm for 16 h (avoid excessive solvent volatilization), the temperature of the reaction systems was controlled within 25 - 35 °C by using the drum fan. After this, 30 μL of *n*-dodecane was added as an internal standard and the reaction mixture was diluted with EtOAc. The crude reaction mixture was filtered through a plug of silica then subjected to GC-MS analysis.

Table S9. Big scale sensitivity

Entry	Conditions	GC yield [%]	Difference [%]
**1**	Add water	11	88
**2**	Under air	79	15
**3**	Big scale	83	11

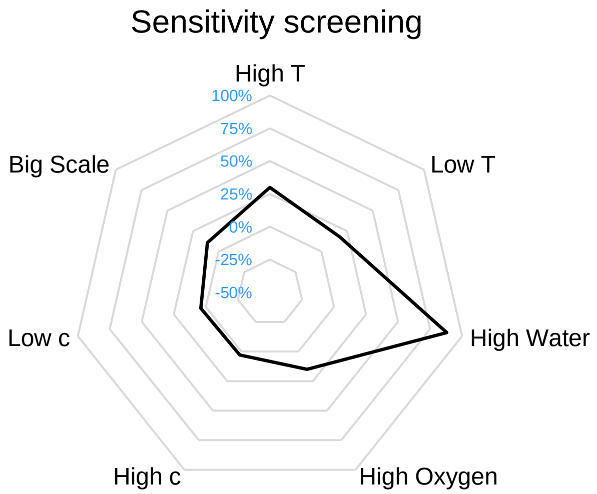

Figure S2. Sensitivity screening.

Mechanistic investigations

UV-vis absorption spectra

UV-visible absorption spectra were recorded on an UV-2700 spectrophotometer, equipped with a temperature control unit at 25 °C. The samples were measured in Starna Fluorometer Microquartz cuvettes (volume: 1.8 ml, path length: 10 mm) equipped with a PTFE-stopper. The spectra were aquired from 300 to 600 nm using 1.0 nm steps. All measurements were performed in MeCN at the following concentrations: benzanilide, TMEDA and *i*-C_3F_7-iodine(III) reagent (1/100 of the reaction concentration); $[Ir(dtbbpy)(ppy)_2]PF_6$ (0.1 mM, 1/10 of the reaction concentration).

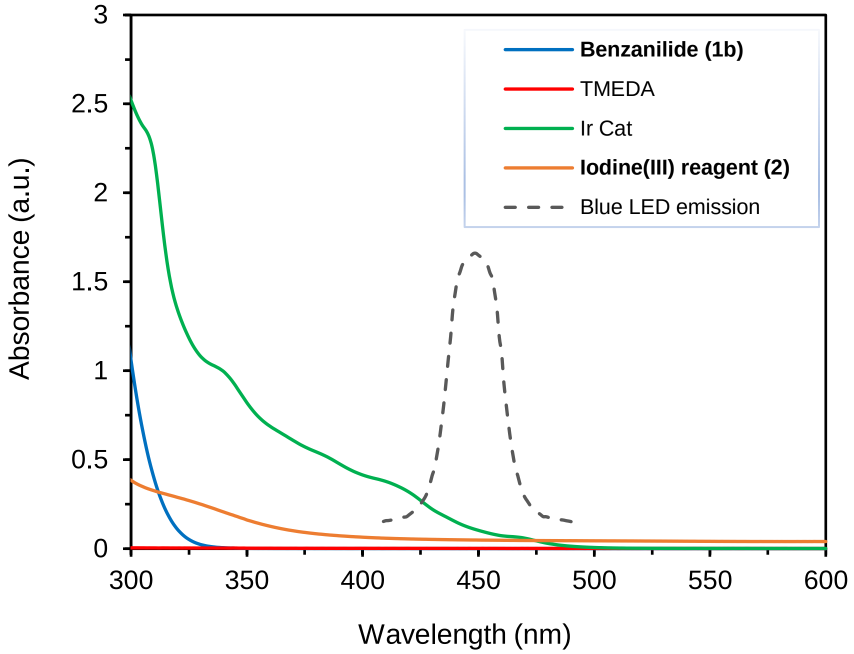

Figure S3. UV-vis absorption spectrum.

Stern-Volmer luminescence quenching experiments

Fluorescence quenching studies were performed using a Hitachi F-4600 spectro fluorometer. In each experiment, $[Ir(dtbbpy)(ppy)_2]PF_6$ and various concentrations of *i*-C_3F_7-iodine(III) reagent or TMEDA were combined in MeCN in Starna Fluorometer Microquartz cuvettes (volume: 1.8 ml, path length: 10 mm) equipped with a PTFE-stopper. The emission quenching of the $[Ir(dtbbpy)(ppy)_2]PF_6$ was achieved using a concentration of 0.1 mM under excitation at 380 nm. The emission intensity was observed at 575 nm. Plots were constructed according to the Stern-Volmer equation $I_0/I = 1 + k_q\tau0[Q]$.

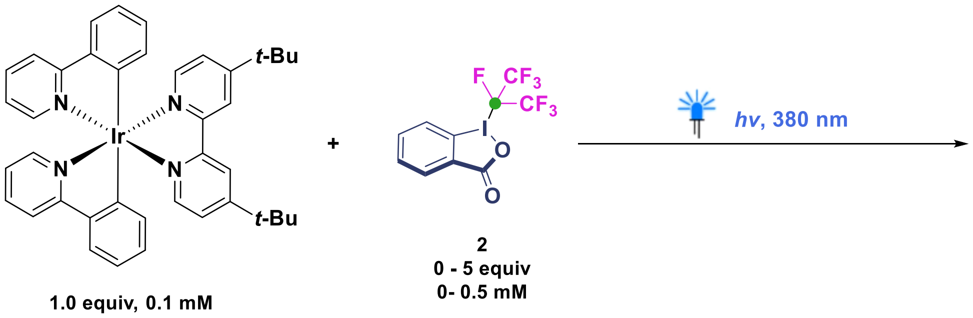

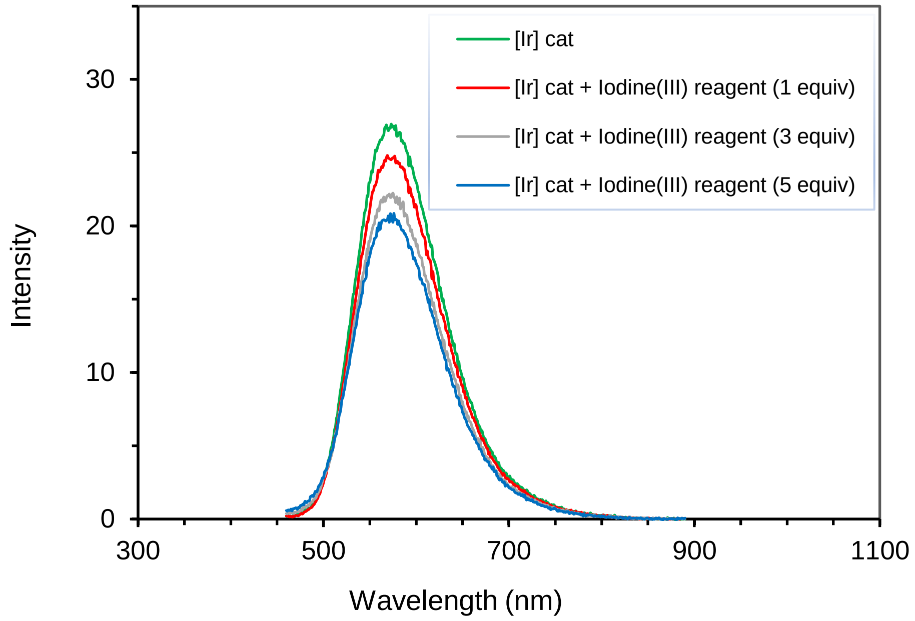

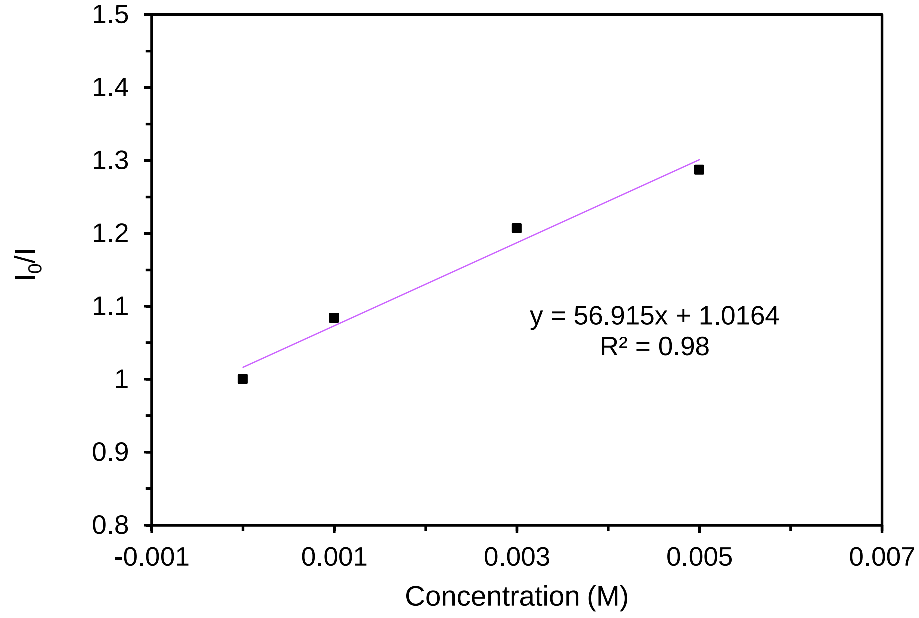

Figure S4. Stern-Volmer luminescence quenching experiments of PFPI reagent.

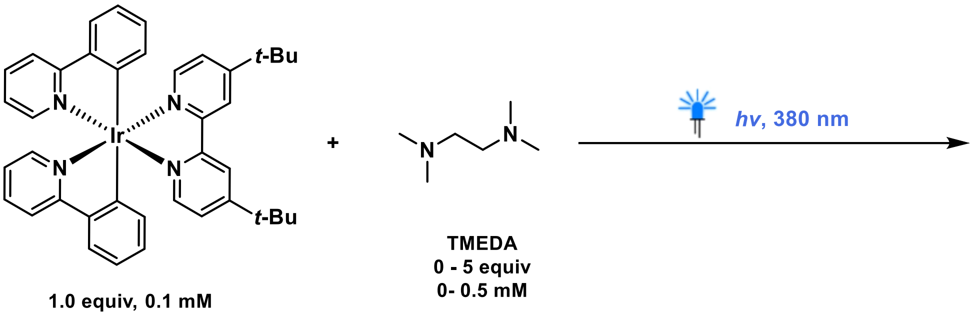

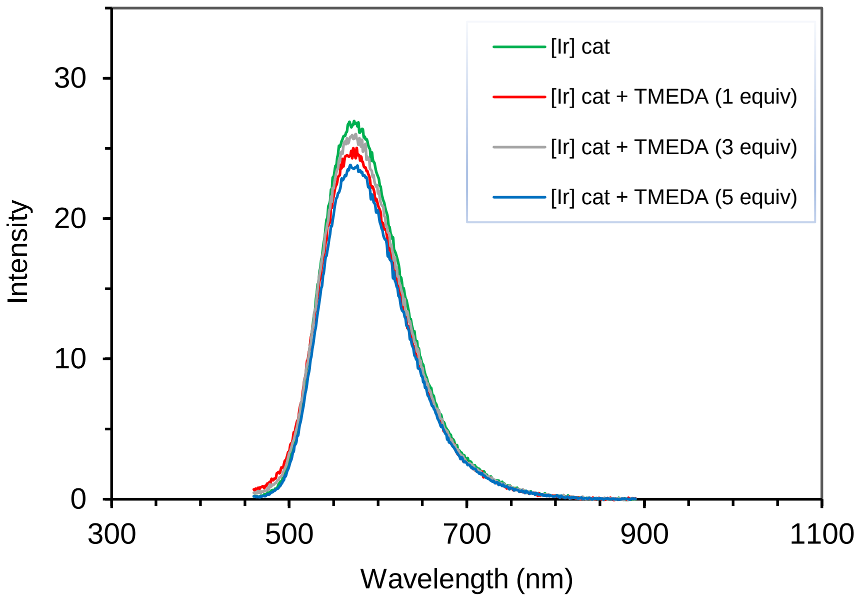

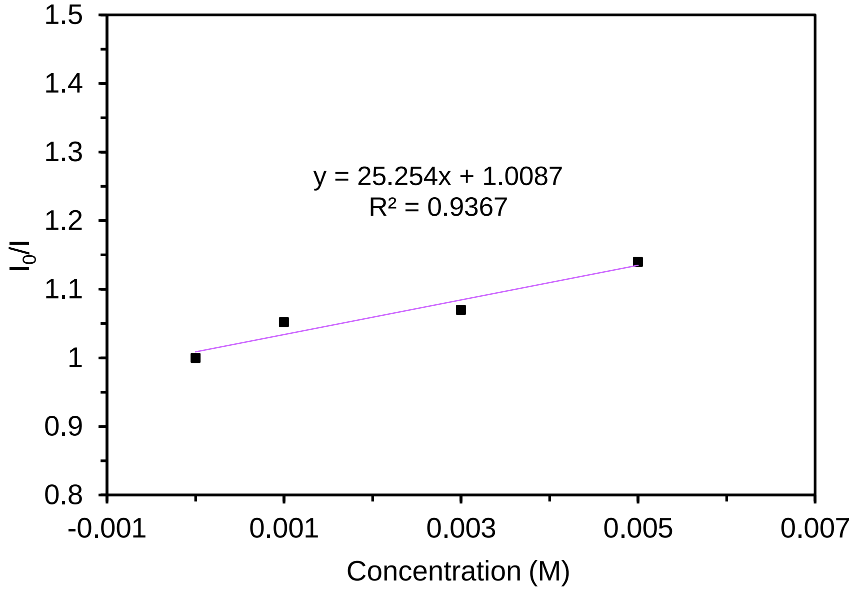

Figure S5. Stern-Volmer luminescence quenching experiments of TMEDA.

Radical trap experiment

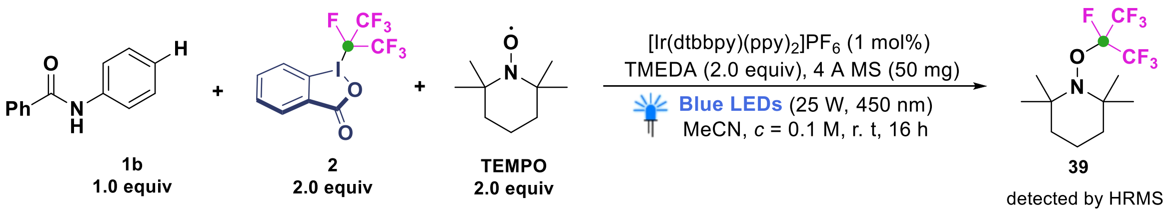

Under an ambient atmosphere, in a 25 mL screw-cap vial equipped with a magnetic stirring bar, benzanilide (**1b**, 39.4 mg, 0.20 mmol, 1.0 equiv.), *i*-C_3F_7-iodine(III) (166.4 mg, 0.40 mmol, 2.0 equiv), $[Ir(dtbbpy)(ppy)_2]PF_6$ (1.8 mg, 1 mol%) and 2, 2, 6, 6-tetramethylpiperidinyl-1-oxide (TEMPO, 62.4 mg, 0.40 mmol, 2.0 equiv) were dissolved in dry MeCN (2 mL). Subsequently, 4 A molecular sieves (50 mg) and TMEDA (59.8 μL, 0.40 mmol, 2.0 equiv) was added into the vial. The vial was sealed and the reaction was irradiated at 450 nm for 16 h, the temperature of the reaction systems was controlled within 25 - 35 °C by using the drum fan. After this, 10 μL of *n*-dodecane was added as an internal standard and the reaction mixture was diluted with EtOAc. The crude reaction mixture was diluted with EtOAc and filtered through a plug of silica then subjected to LC-HRMS analysis. **HRMS (ESI) m/z** $[M+H]^+$: Calculated for $C_{12}H_{19}F_7NO^+$: 326.1349. Found: 326.1357.

Electron paramagnetic resonance (EPR) experiment

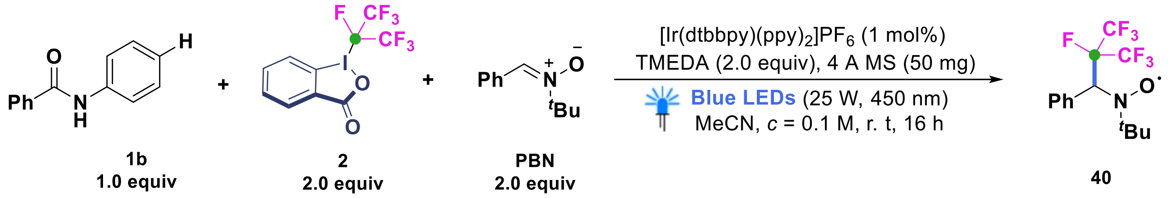

Under an ambient atmosphere, in a 25 mL screw-cap vial equipped with a magnetic stirring bar, benzanilide (**1b**, 39.4 mg, 0.20 mmol, 1.0 equiv.), *i*-C_3F_7-iodine(III) (166.4 mg, 0.40 mmol, 2.0 equiv), $[Ir(dtbbpy)(ppy)_2]PF_6$ (1.8 mg, 1 mol%) and phenyl *tert*-butyl nitrone (PBN, 70.8 mg, 0.40 mmol, 2.0 equiv) were dissolved in dry MeCN (2 mL). Subsequently, 4 A molecular sieves (50 mg) and TMEDA (59.8 μL, 0.40 mmol, 2.0 equiv) was added into the vial. The vial was sealed and the reaction was irradiated at 450 nm for 16 h, the temperature of the reaction systems was controlled within 25 - 35 °C by using the drum fan. After this, 10 μL of *n*-dodecane was added as an internal standard and the reaction mixture was diluted with EtOAc. The crude reaction mixture was filtered through a plug of silica then analyzed by EPR. The EPR showed a EPR signal of nitroxide (g = 2.0063, a^N= 14.76, a^H= 2.08) which indicated that the isoperfluoropropyl group must be substituted at the β-carbon atom of nitroxide.

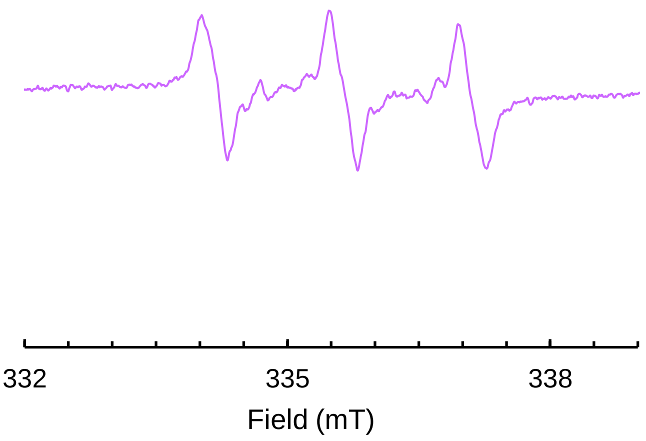

Figure S6. Electron paramagnetic resonance (EPR) experiment.

Kinetic experiment

Synthesis of 1-deutero-2,4-dimethoxybenzene (**41**)

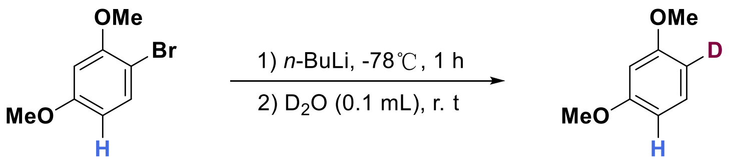

41

Under an ambient atmosphere, in a 25 mL screw-cap vial equipped with a magnetic stirring bar, 1-bromo-2,4-dimethoxybenzene (217.1 mg, 144.1 μL, 1.0 mmol) and dry THF (0.5 mL) were added and the resulting mixture was placed into a cooling bath at -78 ºC. Then, a solution of the *n*-butyllithium (0.44 mL, 1.1 equiv.) in dry THF (2.5 M) was added dropwise during 10 min. The reaction mixture was stirred for additional 30 min at -78 ºC. After this, D_2O (0.1 mL) was added dropwise and the resulting mixture was slowly warmed to room temperature. On completion, the reaction was quenched with sat. aqueous NH_4Cl solution (5 mL) and extracted with DCM (3 x 10 mL). The combined organic phases were washed with brine and dried over anhydrous Na_2SO_4. The mixture was concentrated under reduced pressure to afford 1-deutero-2,4-dimethoxybenzene (**41**) as colorless oil (yield: 136.2 mg, 98%).

R_f = 0.74 (EtOAc: petroleum ether, 1:20 (v:v)).

NMR Spectroscopy:

^{1}H NMR (400 MHz, Chloroform-*d*, 298 K) δ 7.21 (t, *J* = 8.2 Hz, 1 H), 6.48 - 6.54 (m, 2 H), 3.81 (s, 6 H).

^{13}C NMR (101 MHz, Chloroform-*d*, 298 K) δ 160.8, 129.9, 106.1, 100.4, 55.2.

GC-MS: 139

Isotopic (H/D) competition experiment

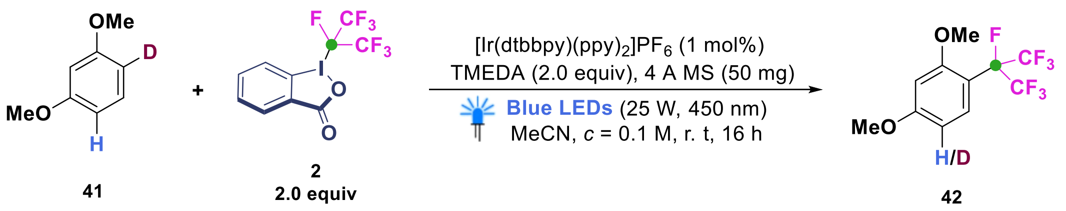

Under an ambient atmosphere, in a 25 mL screw-cap vial equipped with a magnetic stirring bar, 1-deutero-2,4-dimethoxybenzene (**41**, 27.8 mg, 0.20 mmol, 1.0 equiv.), *i*-C_3F_7-iodine(III) (166.4 mg, 0.40 mmol, 2.0 equiv), [Ir(dtbbpy)(ppy)$_2$]PF_6 (1.8 mg, 1 mol%) were dissolved in dry MeCN (2 mL). Subsequently, 4 A molecular sieves (50 mg) and TMEDA (59.8 μL, 0.40 mmol, 2.0 equiv) was added into the vial. The vial was sealed and the reaction was irradiated at 450 nm for 16 h, the temperature of the reaction systems was controlled within 25 - 35 °C by using the drum fan. After this, the reaction was quenched with 10 mL 5% $NaHCO_3$ aqueous solution, then extracted with DCM (3 x 10 mL). The combined organic phase was dried with anhydrous Na_2SO_4 and concentrated under vacuum. The residue was further purified by column chromatography to afford corresponding isoperfluoropropylated products and analyzed by ^{1}H NMR and ^{13}C NMR.

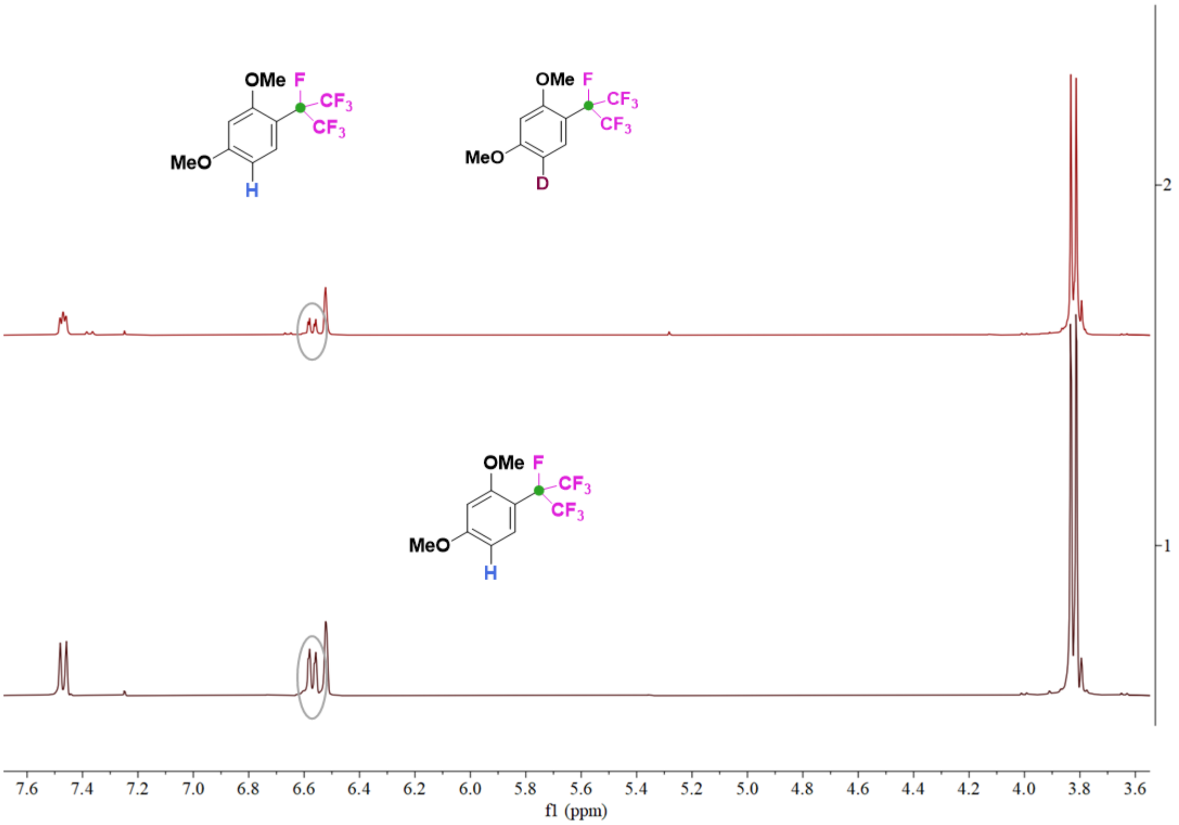

Figure S7. Isotopic (H/D) competition experiment (^{1}H NMR)

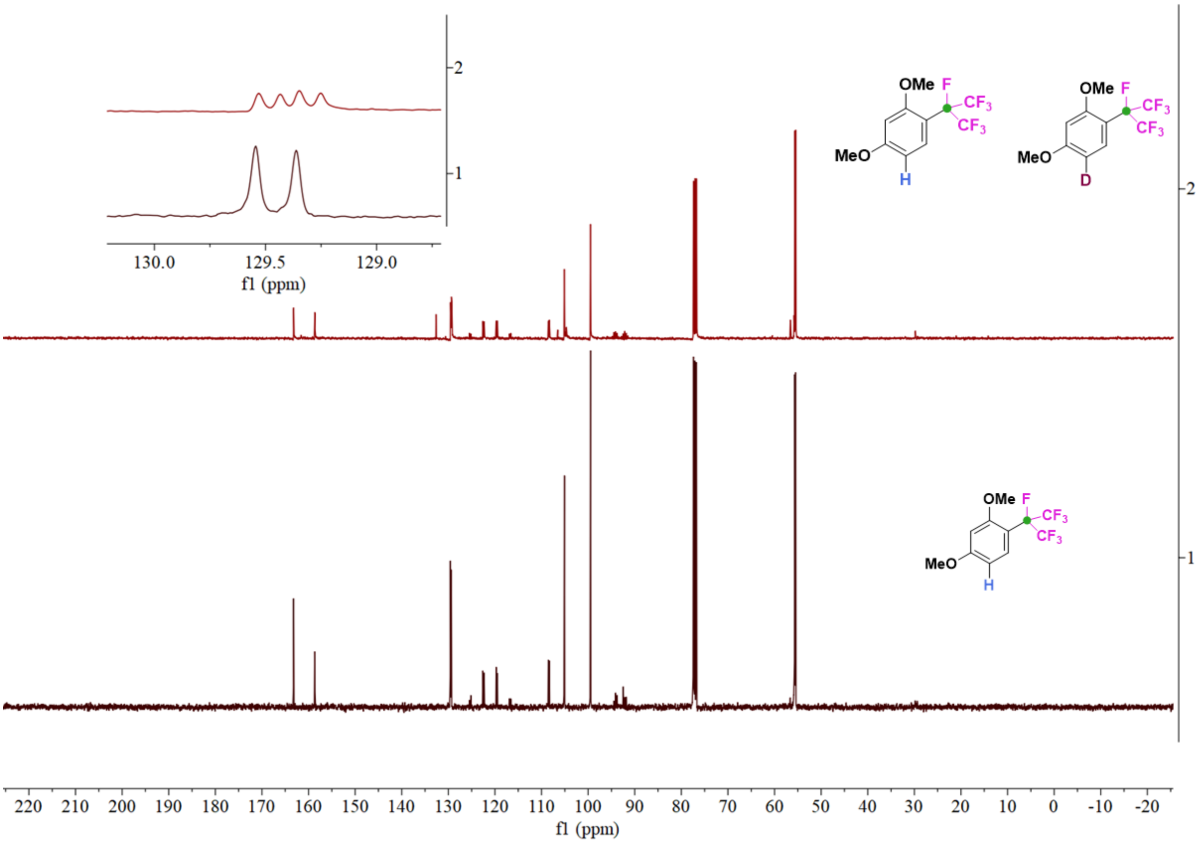

Figure S8. Isotopic (H/D) competition experiment (^{13}C NMR)

NMR Spectroscopy:

^{1}H NMR (400 MHz, Chloroform-*d*, 298 K) δ δ 7.50 - 7.44 (m, 1H), 6.57 (dd, *J* = 8.9, 2.4 Hz, **0.48 H**), 6.52 (s, 1H), 3.83 (s, 3H), 3.81 (s, 3H).

GC-MS: 306 and 307.

Reaction rate measurement

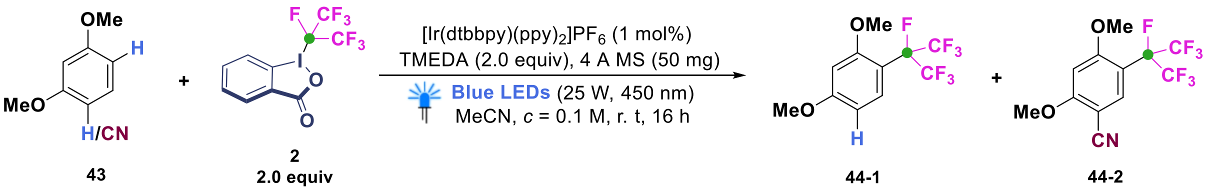

Under an ambient atmosphere, in a 25 mL screw-cap vial equipped with a magnetic stirring bar, 2,4-dimethoxybenzene (**43-1**, 13.8 mg, 0.10 mmol, 0.5 equiv.), 2,4-dimethoxybenzonitrile (**43-2**, 16.3 mg, 0.10 mmol, 0.5 equiv.), *i*-C_3F_7-iodine(III) (166.4 mg, 0.40 mmol, 2.0 equiv) and $[Ir(dtbbpy)(ppy)_2]PF_6$ (1.8 mg, 1 mol%) were dissolved in dry MeCN (2 mL). Subsequently, 4 A molecular sieves (50 mg) and TMEDA (59.8 μL, 0.40 mmol, 2.0 equiv) was added into the vial. The

vial was sealed and the reaction was irradiated at 450 nm, the temperature of the reaction systems was controlled within 25 - 35 °C by using the drum fan. Periodic aliquots (60 μL) (from 30 min to 240 min) were removed by a syringe, filtered by a short of silicon, and then analyzed by GC using *n*-dodecane as internal standard. The yield was determined by GC analysis using *n*-dodecane as an internal standard and average yields was obtained by measuring three times.

Table S10. Reaction rate measurement

Entry	Time [min]	GC yield of 44-1[%]	GC yield of 44-2[%]
**1**	30	37	8
**2**	60	59	15
**3**	120	73	24
**4**	180	83	30
**5**	240	88	36

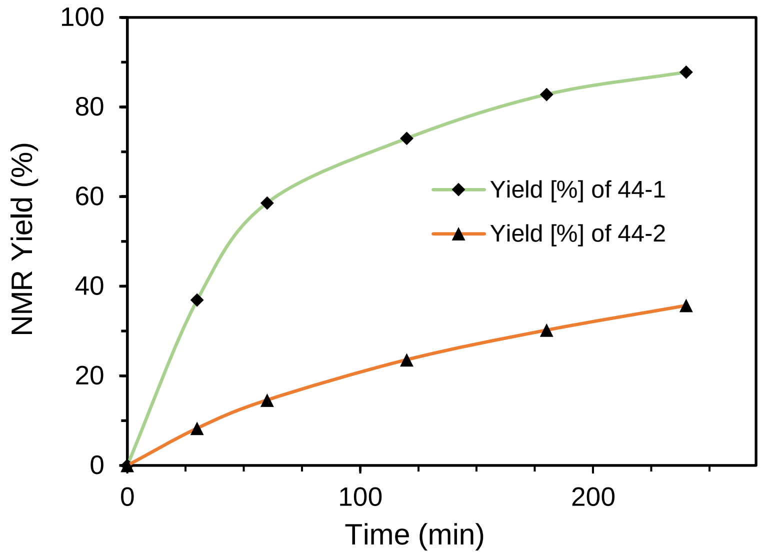

Figure S9. Reaction rate measurement

Substrate scope

General procedure to access isoperfluoropropylated products

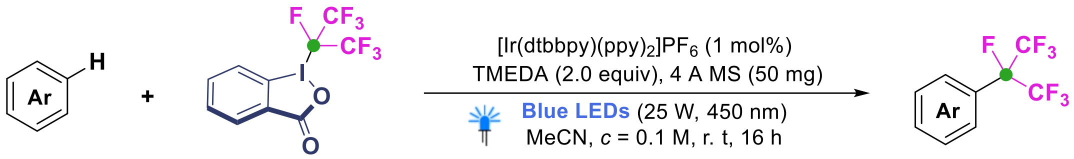

1c 2 38, 45-69

Under an ambient atmosphere, in a 25 mL screw-cap vial equipped with a magnetic stirring bar, arenes (**1c**, 0.20 mmol, 1.0 equiv.), *i*-C_3F_7-iodine(III) (166.4 mg, 0.40 mmol, 2.0 equiv), $[Ir(dtbbpy)(ppy)_2]PF_6$ (1.8 mg, 1 mol%), were dissolved in dry MeCN (2.0 mL). Subsequently, 4 Å molecular sieves (50 mg) and TMEDA (59.8 μL, 0.40 mmol, 2.0 equiv) was added into the vial. The vial was sealed and the reaction was irradiated at 450 nm for 16 h, the temperature of the reaction systems was controlled within 25 - 35 °C by using the drum fan. After this, the reaction was quenched with 10 mL 5% $NaHCO_3$ aqueous solution, then extracted with DCM (3 x 10 mL). The combined organic phase was dried with anhydrous Na_2SO_4 and concentrated under vacuum. The residue was further purified by column chromatography to afford corresponding isoperfluoropropylated products.

N-(4-(*iso*-Perfluoropropyl)phenyl)benzamide (**38**)

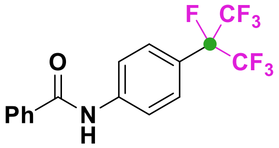

38

N-(4-(*iso*-Perfluoropropyl)phenyl)benzamide (**38**) was prepared according to the general procedure using benzanilide (39.4 mg, 0.20 mmol). The obtained residue was purified by chromatography on silica gel eluting with EtOAc: petroleum ether (1: 20 - 1: 5 (v/v)) to afford 67.9 mg of **38** as a white solid (93% yield).

R_f = 0.27 (EtOAc: petroleum ether, 1:5 (v:v)).

NMR Spectroscopy:

^{1}H NMR (400 MHz, Acetonitrile-*d*$_3$, 298 K) δ 9.01 (br, s, 1H), 7.96 - 7.90 (m, 4H), 7.63 (d, *J* = 8.6 Hz, 2H), 7.59 - 7.54 (m, 1H), 7.53 - 7.46 (m, 2H).

^{13}C NMR (101 MHz, Acetonitrile-*d*$_3$, 298 K) δ 166.26, 142.02, 134.70, 132.06, 128.64, 127.62, 126.39 (d, *J* = 10.8 Hz), 120.78 (qd, *J* = 287.8, 28.0 Hz, CF($\underline{C}F_3)_2$), 120.76 (d, *J* = 20.6 Hz), 120.49 (d, *J* = 2.1 Hz), 93.26 - 90.07 (m, $\underline{C}F(CF_3)_2$).

^{19}F NMR (376 MHz, Acetonitrile-*d*$_3$, 298 K) δ -76.50 (d, *J* = 6.5 Hz, CF(C$\underline{F}_3)_2$, 6F), -182.35 (hept, *J* = 7.1 Hz, C$\underline{F}(CF_3)_2$, 1F).

GC-MS: 365

HRMS (ESI) m/z $[M+H]^+$: Calculated for $C_{16}H_{11}F_7NO^+$: 366.0727. Found: 366.0735.

Melt Point: 158.4 - 160.0 °C

N-(2-Methyl-4-(*iso*-perfluoropropyl)phenyl)acetamide (**45**)

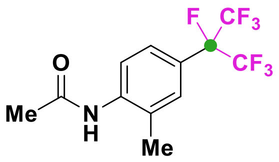

45

N-(2-Methyl-4-(*iso*-perfluoropropyl)phenyl)acetamide (**45**) was prepared according to the general procedure using 2-(acetylamino)toluene (29.8 mg, 0.20 mmol). The obtained residue was purified by chromatography on silica gel eluting with EtOAc: petroleum ether (1: 20 - 1: 5 (v/v)) to afford 57.1 mg of **45** as a pale-yellow solid (90% yield).

$\mathbf{R}_f$ = 0.29 (EtOAc: petroleum ether, 1:5 (v:v)).

NMR Spectroscopy:

^{1}H NMR (400 MHz, Acetonitrile-d_3, 298 K) δ 8.07 (br, s, 1H), 7.90 (d, J = 8.3 Hz, 1H), 7.47 (s, 1H), 7.42 (d, J = 8.1 Hz, 1H), 2.29 (s, 3H), 2.11 (s, 3H).

^{13}C NMR (101 MHz, Acetonitrile-d_3, 298 K) δ 169.30, 139.66, 131.28 (d, *J* = 15.6 Hz), 129.46, 127.48 (d, *J* = 10.8 Hz), 123.76, 123.57 (d, *J* = 10.2 Hz), 121.53, 120.76 (qd, *J* = 287.8, 28.1 Hz, CF($\underline{C}F_3)_2$), 93.40 - 90.11 (m, $\underline{C}F(CF_3)_2$), 23.20, 17.37.

^{19}F NMR (376 MHz, Acetonitrile-d_3, 298 K) δ -76.47 (d, *J* = 7.2 Hz, CF(C$\underline{F}_3)_2$, 6F), -182.49 (dh, *J* = 13.8, 7.2 Hz, C$\underline{F}(CF_3)_2$, 1F).

GC-MS: 317

HRMS (ESI) m/z $[M+H]^+$: Calculated for $C_{12}H_{11}F_7NO^+$: 318.0729. Found: 318.0728.

Melt Point: 129.6 - 131.1 °C

N-(4-(*iso*-Perfluoropropyl)naphthalen-1-yl)acetamide (**46**)

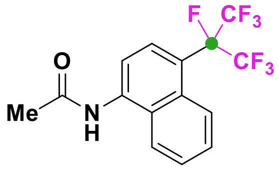

46

N-(4-(*iso*-Perfluoropropyl)naphthalen-1-yl)acetamide (**46**) was prepared according to the general procedure using 1-acetamidonaphthalene (37.0 mg, 0.20 mmol). The obtained residue was purified by chromatography on silica gel eluting with EtOAc: petroleum ether (1: 20 - 1: 5 (v/v)) to afford 59.3 mg of **45** as a white solid (84% yield).

R$_f$ = 0.25 (EtOAc: petroleum ether, 1:5 (v:v)).

NMR Spectroscopy:

^{1}H NMR (400 MHz, Dimethyl sulfoxide-*d$_6$*, 298 K) δ 10.18 (br, s, 1H), 8.31 (t, *J* = 8.4 Hz, 2H), 7.94 (d, *J* = 8.4 Hz, 1H), 7.81 (d, *J* = 8.3 Hz, 1H), 7.69 - 7.62 (m, 2H), 2.21 (s, 3H).

^{13}C NMR (101 MHz, Dimethyl sulfoxide-*d$_6$*, 298 K) δ 169.90, 138.56, 131.73, 128.61, 128.53, 127.41, 126.80, 125.10 (d, *J* = 21.4 Hz), 124.39, 121.44 (qd, *J* = 290.7, 29.5 Hz, CF($\underline{C}F_3)_2$), 119.81, 116.53 (d, *J* = 18.7 Hz), 97.03 - 94.13 (m, $\underline{C}F(CF_3)_2$), 24.19.

^{19}F NMR (376 MHz, Dimethyl sulfoxide-*d$_6$*, 298 K) δ -72.82 (d, *J* = 6.4 Hz, $CF(C\underline{F}_3)_2$, 6F), -172.96 – -173.37 (m, $C\underline{F}(CF_3)_2$, 1F).

GC-MS: 353

HRMS (ESI) m/z [M+H]$^+$: Calculated for $C_{15}H_{11}F_7NO^+$: 354.0727. Found: 354.0729.

Melt Point: 121.7 - 123.0 °C

tert-Butyl (4-*iso*-perfluoropropyl)phenyl)carbamate (**47**)

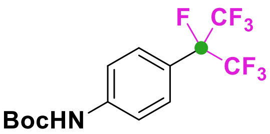

47

tert-Butyl (4-*iso*-perfluoropropyl)phenyl)carbamate (**47**) was prepared according to the general procedure using *tert*-butyl-phenylcarbamate (38.6 mg, 0.20 mmol). The obtained residue was purified by chromatography on silica gel eluting with EtOAc: petroleum ether (1: 20 - 1: 5 (v/v)) to afford 70.0 mg of **47** as a yellow oil (97% yield).

R$_f$ = 0.48 (EtOAc: petroleum ether, 1:5 (v:v)).

NMR Spectroscopy:

^{1}H NMR (400 MHz, Chloroform-*d*, 298 K) δ 7.49 (s, 4H), 6.88 (br, s, 1H), 1.51 (s, 9H).

^{13}C NMR (101 MHz, Chloroform-*d*, 298 K) δ 152.63, 141.13, 126.64 (d, *J* = 10.7 Hz), 120.71 (qd, *J* = 288.3, 28.1 Hz, CF($\underline{C}F_3)_2$), 120.66 (d, *J* = 21.0 Hz), 118.31, 93.36 - 89.63 (m, $\underline{C}F(CF_3)_2$), 81.33, 28.23.

^{19}F NMR (376 MHz, Chloroform-*d*, 298 K) δ -75.91 (d, *J* = 6.8 Hz, $CF(C\underline{F}_3)_2$, 6F), -182.14 (hept, J = 7.7 Hz, 7.1 Hz, $C\underline{F}(CF_3)_2$, 1F).

GC-MS: 361

HRMS (ESI) m/z [M+H]$^+$: Calculated for $C_{14}H_{15}F_7NO_2^+$: 362.0990. Found: 362.0990.

N-Methyl-*N*-(4-(*iso*-perfluoropropyl)-formamide (**48**)

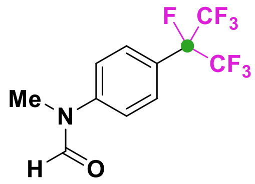

48

N-Methyl-*N*-(4-(*iso*-perfluoropropyl)-formamide (**48**) was prepared according to the general procedure using *N*-methylformanilide (27.0 mg, 0.20 mmol). The obtained residue was purified by chromatography on silica gel eluting with EtOAc: petroleum ether (1: 20 - 1: 5 (v/v)) to afford 52.1 mg of **48** as a colorless oil (86% yield).

$\mathbf{R_f}$ = 0.41 (EtOAc: petroleum ether, 1:5 (v:v)).

NMR Spectroscopy:

^{1}H NMR (400 MHz, Chloroform-*d*, 298 K) δ 8.59 (s, 1H), 7.64 (d, *J* = 8.7 Hz, 2H), 7.30 (d, *J* = 8.3 Hz, 2H), 3.34 (s, 3H).

^{13}C NMR (101 MHz, Chloroform-*d*, 298 K) δ 161.94, 144.64, 127.39 (d, *J* = 10.9 Hz), 124.29 (d, *J* = 21.1 Hz), 121.59, 120.55 (qd, *J* = 288.8, 27.6 Hz, CF(<u>C</u>F$_3$)$_2$), 93.02 - 89.63 (m, <u>C</u>F(CF$_3$)$_2$), 31.59.

^{19}F NMR (376 MHz, Chloroform-*d*, 298 K) δ -75.65 (d, *J* = 7.0 Hz, CF(C<u>*F*</u>$_3$)$_2$, 6F), -182.09 (hept, *J* = 7.2 Hz, C<u>*F*</u>(CF$_3$)$_2$, 1F).

GC-MS: 303

HRMS (ESI) m/z [M+H]$^+$: Calculated for $C_{11}H_9F_7NO^+$: 304.0570. Found: 304.0575.

2,6-Dichloro-4-*iso*-perfluoropropyl-aniline (**49**)

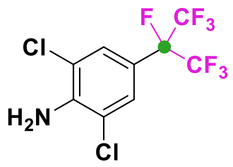

49

2,6-Dichloro-4-*iso*-perfluoropropyl-aniline (**49**) was prepared according to the general procedure using 2,6-dichloroaniline (32.4 mg, 0.20 mmol). The obtained residue was purified by chromatography on silica gel eluting with EtOAc: petroleum ether (1: 20 - 1: 5 (v/v)) to afford 57.4 mg of **49** as a colorless oil (87% yield). The experimental data are in agreement with the literature report[12].

$\mathbf{R_f}$ = 0.27 (EtOAc: petroleum ether, 1:5 (v:v)).

NMR Spectroscopy:

^{1}H NMR (400 MHz, Chloroform-*d*, 298 K) δ 7.40 (s, 2H), 4.75 (br, s, 2H).

^{13}C NMR (101 MHz, Chloroform-*d*, 298 K) δ 142.53, 125.44 (d, *J* = 11.3 Hz), 120.48 (qd, *J* =

288.3, 27.6 Hz, CF($\underline{C}F_3$)$_2$), 119.58 (d, *J* = 2.4 Hz), 115.80 (d, *J* = 21.7 Hz), 91.95 - 88.79 (m, $\underline{C}$F(CF_3)$_2$).

^{19}F NMR (376 MHz, Chloroform-*d*, 298 K) δ -75.84 (d, *J* = 7.5 Hz, CF(C$\underline{F}_3$)$_2$, 6F), -181.43 (hept, *J* = 7.3 Hz, C$\underline{F}$(CF_3)$_2$, 1F).

GC-MS: 329

HRMS (ESI) m/z $[M+H]^+$: Calculated for $C_9H_5Cl_2F_7N^+$: 329.9687. Found: 329.9690.

4-*tert*-Butyl-2-*iso*-perfluoropropyl-6-nitro-aniline (**50**)

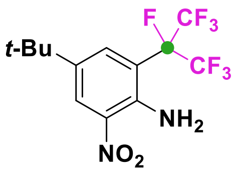

50

4-*tert*-Butyl-2-*iso*-perfluoropropyl-6-nitro-aniline (**50**) was prepared according to the general procedure using 4-*tert*-butyl-2-nitroaniline (38.8 mg, 0.20 mmol). The obtained residue was purified by chromatography on silica gel eluting with EtOAc: petroleum ether (1: 20 - 1: 5 (v/v)) to afford 45.0 mg of **50** as a light-yellow solid (69% yield).

R_f = 0.27 (EtOAc: petroleum ether, 1:5 (v:v)).

NMR Spectroscopy:

^{1}H NMR (400 MHz, Chloroform-*d*, 298 K) δ 8.32 (d, *J* = 2.3 Hz, 1H), 7.61 (s, 1H), 6.85 (br, s, 2H), 1.27 (s, 9H).

^{13}C NMR (101 MHz, Chloroform-*d*, 298 K) δ 142.77, 138.50, 134.42, 132.54, 126.55, 120.86 (qd, *J* = 290.2, 28.5 Hz, CF($\underline{C}F_3$)$_2$), 110.96 (d, *J* = 17.5 Hz), 97.17 - 93.97 (m, $\underline{C}$F(CF_3)$_2$), 34.16, 30.86.

^{19}F NMR (376 MHz, Chloroform-*d*, 298 K) δ -74.17 (d, *J* = 6.4 Hz, CF(C$\underline{F}_3$)$_2$, 6F), -181.74 – -181.51 (m, C$\underline{F}$(CF_3)$_2$, 1F).

GC-MS: 362

HRMS (ESI) m/z $[M+H]^+$: Calculated for $C_{13}H_{14}F_7N_2O_2^+$: 363.0944. Found: 363.0940.

Melt Point: 76.0 - 78.3 °C

4-Phenoxy-2-*iso*-perfluoropropyl-aniline (**51**)

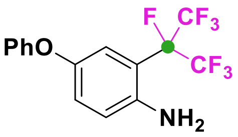

51

4-Phenoxy-2-*iso*-perfluoropropyl-aniline (**51**) was prepared according to the general procedure

using 4-phenoxyanilin (37.0 mg, 0.20 mmol). The obtained residue was purified by chromatography on silica gel eluting with EtOAc: petroleum ether (1: 20 - 1: 5 (v/v)) to afford 54.4 mg of **51** as a yellow oil (77% yield).

R*f* = 0.27 (EtOAc: petroleum ether, 1:5 (v:v)).

NMR Spectroscopy:

^{1}H NMR (400 MHz, Chloroform-*d*, 298 K) δ 7.30 (td, *J* = 7.1, 2.2 Hz, 2H), 7.08 - 7.00 (m, 2H), 6.97 (d, *J* = 8.7 Hz, 1H), 6.91 (d, *J* = 9.4 Hz, 2H), 6.71 (d, *J* = 9.1 Hz, 1H), 4.16 (br, s, 2H).

^{13}C NMR (101 MHz, Chloroform-*d*, 298 K) δ 158.20, 147.67, 143.04, 129.82, 124.17, 122.80, 121.00 (qd, *J* = 289.3, 28.1 Hz, CF(<u>C</u>F$_3$)$_2$), 120.35, 118.87, 117.35, 94.85 - 92.17 (m, <u>C</u>F(CF$_3$)$_2$).

^{19}F NMR (376 MHz, Chloroform-*d*, 298 K) δ -74.56 (s, CF(C<u>F</u>$_3$)$_2$, 6F), -182.14 (s, C<u>F</u>(CF$_3$)$_2$, 1F).

GC-MS: 353

HRMS (ESI) m/z [M+H]$^+$: Calculated for $C_{15}H_{11}F_7NO^+$: 354.0728. Found: 354.0735.

N, *N*-Diethyl-4-*iso*-perfluoropropyl-aniline (**52**)

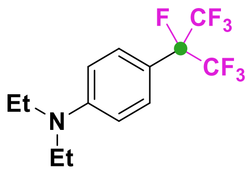

52

N, *N*-Diethyl-4-*iso*-perfluoropropyl-aniline (**52**) was prepared according to the general procedure using *N,N*-diethyl-aniline (24.2 mg, 0.20 mmol). The obtained residue was purified by chromatography on silica gel eluting with EtOAc: petroleum ether (1: 50 - 1: 30 (v/v)) to afford 62.1 mg of **52** as a colorless oil (98% yield).

R*f* = 0.69 (EtOAc: petroleum ether, 1:20 (v:v)).

NMR Spectroscopy:

^{1}H NMR (400 MHz, Chloroform-*d*, 298 K) δ 7.37 (d, *J* = 8.7 Hz, 2H), 6.68 (d, *J* = 7.5 Hz, 2H), 3.38 (q, *J* = 7.1 Hz, 4H), 1.18 (t, *J* = 7.9 Hz, 6H).

^{13}C NMR (101 MHz, Chloroform-*d*, 298 K) δ 149.25, 126.95 (d, *J* = 10.2 Hz), 121.03 (qd, *J* = 285.5, 28.4 Hz, CF(<u>C</u>F$_3$)$_2$), 111.89 (d, *J* = 21.1 Hz), 110.88, 93.57 - 90.28 (m, <u>C</u>F(CF$_3$)$_2$), 44.35, 12.49.

^{19}F NMR (376 MHz, Chloroform-*d*, 298 K) δ -75.96 (d, *J* = 7.7 Hz, CF(C<u>F</u>$_3$)$_2$, 6F), -181.26 (hept, *J* = 7.5 Hz, C<u>F</u>(CF$_3$)$_2$, 1F).

GC-MS: 317

HRMS (ESI) m/z [M+H]$^+$: Calculated for $C_{13}H_{15}F_7N^+$: 318.1093. Found: 318.1095.

N, *N*-Dibutyl-4-*iso*-perfluoropropyl-aniline (**53**)

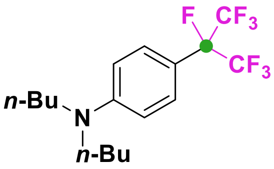

53

N, *N*-Dibutyl-4-*iso*-perfluoropropyl-aniline (**53**) was prepared according to the general procedure using *N*, *N*-Dibutyl-aniline (41.0 mg, 0.20 mmol). The obtained residue was purified by chromatography on silica gel eluting with EtOAc: petroleum ether (1: 50 - 1: 30 (v/v)) to afford 69.4 mg of **53** as a colorless oil (93% yield).

R_f = 0.70 (EtOAc: petroleum ether, 1:20 (v:v)).

NMR Spectroscopy:

^{1}**H NMR** (400 MHz, Chloroform-*d*, 298 K) δ 7.37 (d, *J* = 8.8 Hz, 2H), 6.66 (d, *J* = 9.0 Hz, 2H), 3.29 (t, *J* = 7.4 Hz, 4H), 1.59 (p, *J* = 8.4, 7.7 Hz, 4H), 1.37 (h, *J* = 8.9, 8.2 Hz, 4H), 0.98 (t, *J* = 7.3 Hz, 6H).

^{13}**C NMR** (101 MHz, Chloroform-*d*, 298 K) δ 149.65, 126.87 (d, *J* = 9.9 Hz), 121.05 (qd, *J* = 287.8 Hz, 29.0 Hz, CF($\underline{C}F_3)_2$), 111.76 (d, *J* = 21.2 Hz), 110.94, 93.49 - 90.36 (m, $\underline{C}F(CF_3)_2$), 50.73, 29.30, 20.37, 13.99.

^{19}**F NMR** (376 MHz, Chloroform-*d*, 298 K) δ -75.96 (d, *J* = 7.8 Hz, CF($C\underline{F}_3)_2$, 6F), -181.17 (hept, *J* = 7.5 Hz, $C\underline{F}(CF_3)_2$, 1F).

GC-MS: 373.

HRMS (ESI) m/z $[M+H]^+$: Calculated for $C_{17}H_{23}F_7N^+$: 374.1716. Found: 374.1720.

N, *N*-Diphenyl-4-*iso*-perfluoropropyl-aniline (**54**)

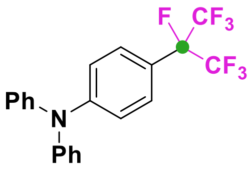

54

N, *N*-Diphenyl-4-*iso*-perfluoropropyl-aniline (**54**) was prepared according to the general procedure using triphenylamine (49.0 mg, 0.20 mmol). The obtained residue was purified by chromatography on silica gel eluting with EtOAc: petroleum ether (1: 50 - 1: 30 (v/v)) to afford 79.3 mg of **54** as a white solid (96% yield).

R_f = 0.57 (EtOAc: petroleum ether, 1:20 (v:v)).

NMR Spectroscopy:

^{1}**H NMR** (400 MHz, Chloroform-*d*, 298 K) δ 7.41 (d, *J* = 8.5 Hz, 2H), 7.37 - 7.23 (m, 4H), 7.17

(d, J = 8.2 Hz, 4H), 7.15 - 7.06 (m, 4H).

^{13}C NMR (101 MHz, Chloroform-*d*, 298 K) δ 150.30, 146.87, 129.71, 126.68 (d, J = 10.3 Hz), 125.77, 124.41, 120.87, 120.86 (qd, J = 288.3 Hz, 28.1 Hz, CF($\underline{C}F_3)_2$), 118.41 (d, J = 20.8 Hz), 93.37 - 89.97 (m, $\underline{C}F(CF_3)_2$).

^{19}F NMR (376 MHz, Chloroform-*d*, 298 K) δ -75.72 (d, J = 7.8 Hz, CF(C$\underline{F}_3)_2$, 6F), -181.57 (hept, J = 7.3 Hz, C$\underline{F}(CF_3)_2$, 1F).

GC-MS: 413.

HRMS (ESI) m/z $[M+H]^+$: Calculated for $C_{21}H_{15}F_7N^+$: 414.1093. Found: 414.1099.

Melt Point: 84.6 - 85.9 °C

N, *N*-Di(*p*-tolyl)-4-*iso*-perfluoropropyl-aniline (**55**)

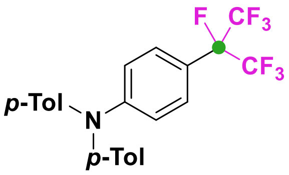

55

N, *N*-Di(*p*-Tolyl)-4-*iso*-perfluoropropyl-aniline (**55**) was prepared according to the general procedure using *N*-phenyl-di-*p*-tolylamine (54.6 mg, 0.20 mmol). The obtained residue was purified by chromatography on silica gel eluting with EtOAc: petroleum ether (1: 50 - 1: 30 (v/v)) to afford 82.9 mg of **55** as a white solid (94% yield).

R_f = 0.57 (EtOAc: petroleum ether, 1:20 (v:v)).

NMR Spectroscopy:

^{1}H NMR (400 MHz, Chloroform-*d*, 298 K) δ 7.35 (d, J = 8.6 Hz, 2H), 7.13 (d, J = 8.0 Hz, 4H), 7.06 (d, J = 8.4 Hz, 4H), 7.01 (d, J = 9.0 Hz, 2H), 2.35 (s, 6H).

^{13}C NMR (101 MHz, Chloroform-*d*, 298 K) δ 150.58, 144.26, 134.22, 130.32, 126.53 (d, J = 10.4 Hz), 125.95, 120.88 (qd, J = 288.3 Hz, 28.1 Hz, CF($\underline{C}F_3)_2$), 119.59 (d, J = 2.0 Hz), 117.32 (d, J = 20.8 Hz), 93.31 - 90.11 (m, $\underline{C}F(CF_3)_2$), 20.96.

^{19}F NMR (376 MHz, Chloroform-*d*, 298 K) δ -75.75 (d, J = 7.6 Hz, CF(C$\underline{F}_3)_2$, 6F), -181.49 (hept, J = 7.3 Hz, C$\underline{F}(CF_3)_2$, 1F).

GC-MS: 441.

HRMS (ESI) m/z $[M+H]^+$: Calculated for $C_{23}H_{19}F_7N^+$: 442.1403. Found: 442.1403.

Melt Point: 89.5 - 91.1 °C

N-((4-*iso*-Perfluoropropyl)-phenyl)-piperidine (**56**)

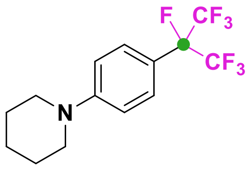

56

N-((4-*iso*-Perfluoropropyl)-phenyl)-piperidine (**56**) was prepared according to the general procedure using *N*-phenyl piperidine (32.2 mg, 0.20 mmol). The obtained residue was purified by chromatography on silica gel eluting with EtOAc: petroleum ether (1: 50 - 1: 30 (v/v)) to afford 50.6 mg of **56** as a pale-yellow oil (77% yield).

R_f = 0.67 (EtOAc: petroleum ether, 1:20 (v:v)).

NMR Spectroscopy:

^{1}H NMR (400 MHz, Chloroform-*d*, 298 K) δ 7.40 (d, *J* = 9.0 Hz, 2H), 6.93 (d, *J* = 9.1 Hz, 2H), 3.25 (t, *J* = 5.3 Hz, 4H), 1.76 - 1.52 (m, 6H).

^{13}C NMR (101 MHz, Chloroform-*d*, 298 K) δ 153.19, 126.74 (d, J = 10.2 Hz), 120.91 (qd, *J* = 288.6 Hz, 28.8 Hz, CF($\underline{C}F_3$)$_2$), 115.24, 114.90, 92.10 - 87.97 (m, $\underline{C}$F(CF_3)$_2$), 49.18, 25.58, 24.32.

^{19}F NMR (376 MHz, Chloroform-*d*, 298 K) δ -75.88 (d, *J* = 7.7 Hz, CF(C$\underline{F}_3$)$_2$, 6F), -181.63 (hept, *J* = 7.4 Hz, C$\underline{F}$(CF_3)$_2$, 1F).

GC-MS: 329.

HRMS (ESI) m/z $[M+H]^+$: Calculated for $C_{14}H_{15}F_7N^+$: 330.1093. Found: 330.1094.

N-((4-*iso*-Perfluoropropyl)-phenyl)-morpholine (**57**)

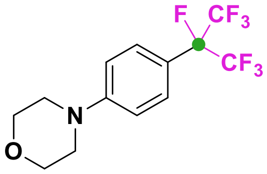

57

N-((4-*iso*-Perfluoropropyl)-phenyl)-morpholine (**57**) was prepared according to the general procedure using *N*-phenyl-morpholine (32.6 mg, 0.20 mmol). The obtained residue was purified by chromatography on silica gel eluting with EtOAc: petroleum ether (1: 50 - 1: 30 (v/v)) to afford 49.0 mg of **57** as a white solid (74% yield).

R_f = 0.54 (EtOAc: petroleum ether, 1:20 (v:v)).

NMR Spectroscopy:

^{1}H NMR (400 MHz, Chloroform-*d*, 298 K) δ 7.46 (d, *J* = 8.6 Hz, 2H), 6.94 (d, *J* = 7.8 Hz, 2H), 3.85 (t, *J* = 4.9 Hz, 4H), 3.23 (t, *J* = 4.9 Hz, 4H).

^{13}C NMR (101 MHz, Chloroform-*d*, 298 K) δ 152.72, 126.88 (d, *J* = 10.4 Hz), 120.86 (qd, *J* = 287.8 Hz, 27.6 Hz, CF($\underline{C}F_3$)$_2$), 116.66 (d, *J* = 21.2 Hz), 114.65, 93.48 - 90.33 (m, $\underline{C}$F(CF_3)$_2$),

66.76, 48.08.

^{19}F NMR (376 MHz, Chloroform-*d*, 298 K) δ -75.86 (d, *J* = 7.6 Hz, CF(C*F*$_3$)$_2$, 6F), -181.82 (hept, *J* = 7.3 Hz, C*F*(CF$_3$)$_2$, 1F).

GC-MS: 331.

HRMS (ESI) m/z [M+H]$^+$: Calculated for $C_{13}H_{13}F_7NO^+$: 332.0881. Found: 332.0886.

Melt Point: 77.0 - 78.8 °C

1,3-Dimethoxy-4-*iso*-perfluoropropyl-benzene (**58**)

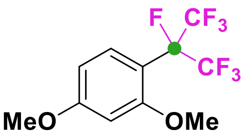

58

1,3-Dimethoxy-4-*iso*-perfluoropropyl-benzene (**58**) was prepared according to the general procedure using 1,3-dimethoxy-benzene (27.6 mg, 0.20 mmol). The obtained residue was purified by chromatography on silica gel eluting with EtOAc: petroleum ether (1: 50 - 1: 30 (v/v)) to afford 55.7 mg of **58** as a colorless oil (91% yield).

R$_f$ = 0.75 (EtOAc: petroleum ether, 1:20 (v:v)).

NMR Spectroscopy:

^{1}H NMR (400 MHz, Chloroform-*d*, 298 K) δ 7.47 (d, *J* = 8.8 Hz, 1H), 6.57 (dd, *J* = 8.9, 2.2 Hz, 1H), 6.52 (d, *J* = 2.4 Hz, 1H), 3.83 (s, 3H), 3.81 (s, 3H).

^{13}C NMR (101 MHz, Chloroform-*d*, 298 K) δ 163.20, 158.66 (d, *J* = 4.9 Hz), 129.45 (d, *J* = 18.5 Hz), 121.03 (qd, *J* = 286.1, 28.2 Hz, CF(*C*F$_3$)$_2$), 108.43 (d, *J* = 22.3 Hz), 105.10 (d, *J* = 3.2 Hz), 99.49, 94.95 – 91.34 (m, *C*F(CF$_3$)$_2$), 55.66, 55.48.

^{19}F NMR (376 MHz, Chloroform-*d*, 298 K) δ -74.69 (d, *J* = 4.7 Hz, CF(C*F*$_3$)$_2$, 6F), -173.10 (s, C*F*(CF$_3$)$_2$, 1F).

GC-MS: 306

HRMS (ESI) m/z [M+H]$^+$: Calculated for $C_{11}H_{10}F_7O_2^+$: 307.0568. Found: 307.0577.

1-Bromo-2,4-dimethoxy-5-*iso*-perfluoropropyl-benzene (**59**)

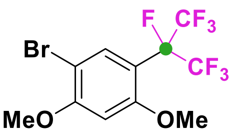

59

1-Bromo-2,4-dimethoxy-5-*iso*-perfluoropropyl-benzene (**59**) was prepared according to the general procedure using 1-bromo-2,4-dimethoxy-benzene (27.6 mg, 0.20 mmol). The obtained residue was purified by chromatography on silica gel eluting with EtOAc: petroleum ether (1: 50 - 1: 30 (v/v)) to afford 67.8 mg of **59** as a white solid (88% yield).

R_f = 0.68 (EtOAc: petroleum ether, 1:20 (v:v)).

NMR Spectroscopy:

^{1}H NMR (400 MHz, Chloroform-*d*, 298 K) δ 7.67 (s, 1H), 6.51 (s, 1H), 3.93 (s, 3H), 3.85 (s, 3H).

^{13}C NMR (101 MHz, Chloroform-*d*, 298 K) δ 159.07, 158.09 (d, *J* = 5.1 Hz), 132.34 (d, *J* = 19.7 Hz), 120.82 (qd, *J* = 287.5, 27.9 Hz, CF($\underline{C}F_3$)$_2$), 109.25 (d, *J* = 22.6 Hz), 102.51 (d, *J* = 3.3 Hz), 97.01, 94.22 - 90.62 (m, $\underline{C}$F(CF_3)$_2$), 56.42, 56.12.

^{19}F NMR (376 MHz, Chloroform-*d*, 298 K) δ -74.57 (d, *J* = 4.8 Hz, CF(C$\underline{F}_3$)$_2$, 6F), -172.82 (s, C$\underline{F}$(CF_3)$_2$, 1F).

GC-MS: 384

HRMS (ESI) m/z $[M+H]^+$: Calculated for $C_{11}H_9BrF_7O_2^+$: 384.9674. Found: 384.9677.

Melt Point: 66.2 - 68.0 °C

5-*iso*-Perfluoropropyl-benzo[d][1,3]-dioxole (**60**)

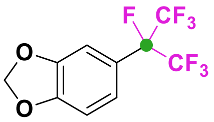

60

5-*iso*-Perfluoropropyl-benzo[d][1,3]-dioxole (**60**) was prepared according to the general procedure using methylenedioxybenzene (24.4 mg, 0.20 mmol). The obtained residue was purified by chromatography on silica gel eluting with EtOAc: petroleum ether (1: 50 - 1: 30 (v/v)) to afford 44.7 mg of **60** as a colorless oil (77% yield). The experimental data are in agreement with the literature report[13].

R_f = 0.65 (EtOAc: petroleum ether, 1:20 (v:v)).

NMR Spectroscopy:

^{1}H NMR (400 MHz, Chloroform-*d*, 298 K) δ 7.10 (d, *J* = 8.4 Hz, 1H), 7.03 (s, 1H), 6.89 (dd, *J* = 8.3, 1.9 Hz, 1H), 6.04 (s, 2H).

^{13}C NMR (101 MHz, Chloroform-*d*, 298 K) δ 149.90, 148.41, 120.66 (qd, *J* = 288.3, 28.5 Hz, CF($\underline{C}F_3$)$_2$), 120.22 (d, *J* = 11.7 Hz), 119.89, 108.63, 106.34 (d, *J* = 11.7 Hz), 101.98, 93.28 - 89.73 (m, $\underline{C}$F(CF_3)$_2$).

^{19}F NMR (376 MHz, Chloroform-*d*, 298 K) δ -75.78 (d, *J* = 7.1 Hz, CF(C$\underline{F}_3$)$_2$, 6F), -180.30 (hept, *J* = 7.0 Hz, C$\underline{F}$(CF_3)$_2$, 1F).

GC-MS: 290

HRMS (ESI) m/z $[M+H]^+$: Calculated for $C_{10}H_6F_7O_2^+$: 291.0253. Found: 291.0248.

3-*iso*-Perfluoropropyl-coumarin (**61**)

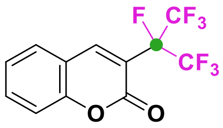

61

3-*iso*-Perfluoropropyl-coumarin (**61**) was prepared according to the general procedure using coumarin (29.2 mg, 0.20 mmol). The obtained residue was purified by chromatography on silica gel eluting with EtOAc: petroleum ether (1: 50 - 1: 20 (v/v)) to afford 57.8 mg of **61** as a white solid (92% yield). The experimental data are in agreement with the literature report[14].

R_f = 0.44 (EtOAc: petroleum ether, 1:20 (v:v)).

NMR Spectroscopy:

^{1}H NMR (400 MHz, Chloroform-*d*, 298 K) δ 8.19 (s, 1H), 7.73 - 7.56 (m, 2H), 7.44 - 7.33 (m, 2H).

^{13}C NMR (101 MHz, Chloroform-*d*, 298 K) δ 155.98 (d, *J* = 6.2 Hz), 154.46, 145.82 (d, *J* = 17.0 Hz), 134.47, 129.45, 125.31, 120.22 (qd, *J* = 287.9, 27.2 Hz, CF($\underline{C}F_3)_2$), 117.28 (d, *J* = 2.6 Hz), 116.77, 115.83 (d, *J* = 22.4 Hz), 93.58 - 89.48 (m, $\underline{C}F(CF_3)_2$).

^{19}F NMR (376 MHz, Chloroform-*d*, 298 K) δ -73.86 (d, *J* = 5.3 Hz, CF($C\underline{F}_3)_2$, 6F), -175.20 (s, $C\underline{F}(CF_3)_2$, 1F).

GC-MS: 314

HRMS (ESI) m/z $[M+H]^+$: Calculated for $C_{12}H_6F_7O_2^+$: 315.0254. Found: 315.0252.

6-Methyl-3-*iso*-perfluoropropyl-coumarin (**62**)

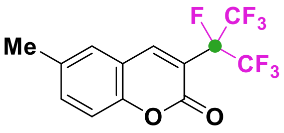

62

6-Methyl-3-*iso*-perfluoropropyl-coumarin (**62**) was prepared according to the general procedure using 6-methylcoumarin (32.0 mg, 0.20 mmol). The obtained residue was purified by chromatography on silica gel eluting with EtOAc: petroleum ether (1: 50 - 1: 20 (v/v)) to afford 59.0 mg of **62** as a white solid (90% yield).

R_f = 0.46 (EtOAc: petroleum ether, 1:20 (v:v)).

NMR Spectroscopy:

^{1}H NMR (400 MHz, Chloroform-*d*, 298 K) δ 8.13 (s, 1H), 7.47 (d, *J* = 10.2 Hz, 1H), 7.40 (s, 1H), 7.27 (dd, *J* = 8.5, 2.9 Hz, 1H), 2.43 (s, 3H).

^{13}C NMR (101 MHz, Chloroform-*d*, 298 K) δ 156.15 (d, *J* = 6.1 Hz), 152.66, 145.66 (d, *J* = 17.0 Hz), 135.53, 135.21, 129.05, 120.24 (qd, *J* = 288.0, 27.1 Hz, CF($\underline{C}F_3)_2$), 117.05, 116.54, 115.71 (d, *J* = 22.3 Hz), 93.30 - 89.80 (m, $\underline{C}F(CF_3)_2$), 20.73.

^{19}**F NMR** (376 MHz, Chloroform-*d*, 298 K) δ -73.80 (d, *J* = 3.9 Hz, $CF(C\underline{F}_3)_2$, 6F), -175.14 (s, $C\underline{F}(CF_3)_2$, 1F).

GC-MS: 328

HRMS (ESI) m/z $[M+H]^+$: Calculated for $C_{13}H_8F_7O_2^+$: 329.0414. Found: 329.0422.

Melt Point: 95.2 - 96.4 °C

7-Methoxy-3-*iso*-perfluoropropyl-coumarin (**63**)

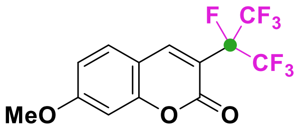

63

7-Methoxy-3-*iso*-perfluoropropyl-coumarin (**63**) was prepared according to the general procedure using 7-methoxycoumarin (35.2 mg, 0.20 mmol). The obtained residue was purified by chromatography on silica gel eluting with EtOAc: petroleum ether (1: 50 - 1: 20 (v/v)) to afford 59.9 mg of **63** as a white solid (87% yield).

$\mathbf{R_f}$ = 0.46 (EtOAc: petroleum ether, 1:20 (v:v)).

NMR Spectroscopy:

^{1}**H NMR** (400 MHz, Chloroform-*d*, 298 K) δ 8.09 (s, 1H), 7.50 (d, *J* = 8.7 Hz, 1H), 6.91 (dd, *J* = 8.7, 2.4 Hz, 1H), 6.85 - 6.80 (m, 1H), 3.89 (s, 3H).

^{13}**C NMR** (101 MHz, Chloroform-*d*, 298 K) δ 165.09, 156.70, 156.36 (d, *J* = 6.2 Hz), 145.50 (d, *J* = 16.6 Hz), 130.47, 120.34 (qd, *J* = 287.9, 27.3 Hz, $CF(\underline{C}F_3)_2$), 113.80 (d, *J* = 3.2 Hz), 111.51 (d, *J* = 22.7 Hz), 111.04 (d, *J* = 2.6 Hz), 100.46, 93.25 - 89.75 (m, $\underline{C}F(CF_3)_2$), 56.07.

^{19}**F NMR** (376 MHz, Chloroform-*d*, 298 K) δ -74.05 (d, *J* = 5.4 Hz, $CF(C\underline{F}_3)_2$, 6F), -175.32 (s, $C\underline{F}(CF_3)_2$, 1F).

GC-MS: 344

HRMS (ESI) m/z $[M+H]^+$: Calculated for $C_{13}H_8F_7O_3^+$: 345.0363. Found: 345.0362.

Melt Point: 121.4 - 123.0 °C

6-Bromo-3-*iso*-perfluoropropyl-coumarin (**64**)

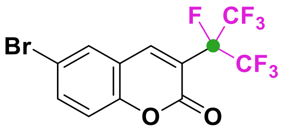

64

6-Bromo-3-*iso*-perfluoropropyl-coumarin (**64**) was prepared according to the general procedure using 7-bromocoumarin (45.0 mg, 0.20 mmol). The obtained residue was purified by chromatography on silica gel eluting with EtOAc: petroleum ether (1: 50 - 1: 20 (v/v)) to afford 75.5 mg of **64** as a white solid (96% yield).

R_f = 0.43 (EtOAc: petroleum ether, 1:20 (v:v)).

NMR Spectroscopy:

^{1}H NMR (400 MHz, Chloroform-*d*, 298 K) δ 8.13 (s, 1H), 7.78 (d, *J* = 2.3 Hz, 1H), 7.74 (dd, *J* = 8.8, 2.3 Hz, 1H), 7.27 (d, *J* = 8.8 Hz, 1H).

^{13}C NMR (101 MHz, Chloroform-*d*, 298 K) δ 155.24 (d, *J* = 5.9 Hz), 153.31, 144.54 (d, *J* = 17.2 Hz), 137.13, 131.62, 120.09 (qd, *J* = 289.3, 27.1 Hz, CF($\underline{C}F_3)_2$), 118.74 (d, *J* = 2.7 Hz), 118.53, 117.82, 117.17 (d, *J* = 22.5 Hz), 93.22 - 89.63 (m, $\underline{C}F(CF_3)_2$).

^{19}F NMR (376 MHz, Chloroform-*d*, 298 K) δ -73.78 (d, *J* = 5.4 Hz, CF(C$\underline{F}_3)_2$, 6F), -175.16 (s, C$\underline{F}(CF_3)_2$, 1F).

GC-MS: 392

HRMS (ESI) m/z $[M+H]^+$: Calculated for $C_{12}H_5BrF_7O_2^+$: 392.9361. Found: 392.9361.

Melt Point: 119.4 - 121.9 °C

7-Diethylamino-4-methyl-3-*iso*-perfluoropropyl-coumarin (**65**)

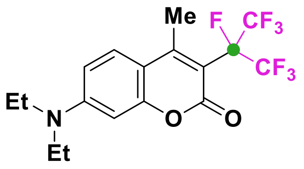

65

7-Diethylamino-4-methyl-3-*iso*-perfluoropropyl-coumarin (**65**) was prepared according to the general procedure using 7-diethylamino-4-methyl-coumarin (46.2 mg, 0.20 mmol). The obtained residue was purified by chromatography on silica gel eluting with EtOAc: petroleum ether (1: 50 - 1: 20 (v/v)) to afford 66.2 mg of **65** as an orange solid (83% yield).

R_f = 0.36 (EtOAc: petroleum ether, 1:20 (v:v)).

NMR Spectroscopy:

^{1}H NMR (400 MHz, Chloroform-*d*, 298 K) δ 7.58 (d, *J* = 9.4 Hz, 1H), 6.62 (dd, *J* = 9.3, 2.7 Hz, 1H), 6.40 (d, *J* = 2.7 Hz, 1H), 3.41 (q, *J* = 7.1 Hz, 4H), 2.57 (d, *J* = 6.4 Hz, 3H), 1.19 (t, *J* = 7.2 Hz, 6H).

^{13}C NMR (101 MHz, Chloroform-*d*, 298 K) δ 158.41 (d, *J* = 8.5 Hz), 157.85, 155.65, 151.91, 127.14, 121.23 (qd, *J* = 288.8, 28.2 Hz, CF($\underline{C}F_3)_2$), 109.39, 108.96, 105.62 (d, *J* = 21.7 Hz), 96.63, 96.48 - 93.41 (m, $\underline{C}F(CF_3)_2$), 44.92, 16.00 (d, *J* = 20.1 Hz), 12.39.

^{19}F NMR (376 MHz, Chloroform-*d*, 298 K) δ -72.93 (d, *J* = 4.2 Hz, CF(C$\underline{F}_3)_2$, 6F), -167.21 (hept, *J* = 6.5 Hz, C$\underline{F}(CF_3)_2$, 1F).

GC-MS: 399

HRMS (ESI) m/z $[M+H]^+$: Calculated for $C_{17}H_{17}F_7NO_2^+$: 400.1146. Found: 400.1157.

Melt Point: 83.2 - 84.4 °C

6-Bromo-3-*iso*-perfluoropropyl-1H-indazole (**66**)

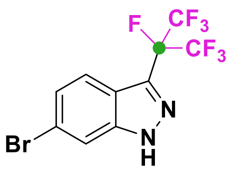

66

6-Bromo-3-*iso*-perfluoropropyl-1H-indazole (**66**) was prepared according to the general procedure using 6-bromo-1H-indazole (39.4 mg, 0.20 mmol). The obtained residue was purified by chromatography on silica gel eluting with EtOAc: petroleum ether (1: 20 - 1: 5 (v/v)) to afford 54.6 mg of **66** as a white solid (75% yield).

R_f = 0.22 (EtOAc: petroleum ether, 1:5 (v:v)).

NMR Spectroscopy:

^{1}H NMR (400 MHz, Chloroform-*d*, 298 K) δ 10.96 (br, s, 1H), 7.79 - 7.69 (m, 2H), 7.38 (dd, *J* = 8.7, 1.6 Hz, 1H).

^{13}C NMR (101 MHz, Chloroform-*d*, 298 K) δ 141.62, 126.80, 122.54, 122.14, 122.09, 120.56, 120.43 (qd, *J* = 287.8, 26.6 Hz, CF($\underline{C}F_3)_2$), 113.31, 95.14 - 92.02 (m, $\underline{C}F(CF_3)_2$).

^{19}F NMR (376 MHz, Chloroform-*d*, 298 K) δ -75.39 (d, *J* = 7.9 Hz, CF(C$\underline{F}_3)_2$, 6F), -182.81 (hept, *J* = 8.2 Hz, C$\underline{F}(CF_3)_2$, 1F).

GC-MS: 364

HRMS (ESI) m/z $[M+H]^+$: Calculated for $C_{10}H_5BrF_7N_2^+$: 364.9527. Found: 364.9524.

Melt Point: 141.6 - 143.6 °C

1-Methyl-3-*iso*-perfluoropropyl-1H-indazole-5-carbaldehyde (**67**)

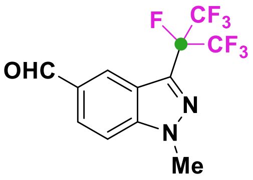

67

1-Methyl-3-*iso*-perfluoropropyl-1H-indazole-5-carbaldehyde (**67**) was prepared according to the general procedure using 1-methyl-1H-indazole-5-carbaldehyde (32.0 mg, 0.20 mmol). The obtained residue was purified by chromatography on silica gel eluting with EtOAc: petroleum ether (1: 20 - 1: 5 (v/v)) to afford 46.6 mg of **67** as a pale-pink solid (71% yield).

R_f = 0.41 (EtOAc: petroleum ether, 1:5 (v:v)).

NMR Spectroscopy:

^{1}H NMR (400 MHz, Chloroform-*d*, 298 K) δ 10.06 (s, 1H), 8.34 (s, 1H), 8.03 (d, *J* = 8.9 Hz, 1H), 7.56 (d, *J* = 8.3 Hz, 1H), 4.18 (s, 3H).

^{13}**C NMR** (101 MHz, Chloroform-*d*, 298 K) δ 191.37, 143.10, 133.05 (d, *J* = 29.2 Hz), 131.99, 127.66 (d, *J* = 5.2 Hz), 125.87, 122.26, 120.35 (qd, *J* = 287.4, 27.1 Hz, CF(<u>C</u>F$_3$)$_2$), 110.70, 92.95 - 89.63 (m, <u>C</u>F(CF$_3$)$_2$), 36.64.

^{19}**F NMR** (376 MHz, Chloroform-*d*, 298 K) δ -75.37 (d, *J* = 8.6 Hz, CF(C<u>F</u>$_3$)$_2$, 6F), -182.59 (hept, *J* = 8.1 Hz, C<u>F</u>(CF$_3$)$_2$, 1F).

GC-MS: 328

HRMS (ESI) m/z [M+H]$^+$: Calculated for $C_{12}H_8F_7N_2O^+$: 329.0527. Found: 329.0525.

Melt Point: 61.9 - 63.3 °C

4-*iso*-Perfluoropropyl-2,1,3-benzothiadiazole (**68**)

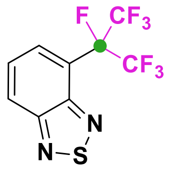

68

4-*iso*-Perfluoropropyl-2,1,3-benzothiadiazole (**68**) was prepared according to the general procedure using 2,1,3-benzothiadiazole (40.8 mg, 0.20 mmol). The obtained residue was purified by chromatography on silica gel eluting with EtOAc: petroleum ether (1: 40 - 1: 20 (v/v)) to afford 51.1 mg of **68** as a pale-yellow solid (84% yield).

R$_f$ = 0.44 (EtOAc: petroleum ether, 1:10 (v:v)).

NMR Spectroscopy:

^{1}**H NMR** (400 MHz, Chloroform-*d*, 298 K) δ 8.22 (d, *J* = 8.8 Hz, 1H), 7.96 (d, *J* = 7.3 Hz, 1H), 7.75 - 7.67 (m, 1H).

^{13}**C NMR** (101 MHz, Chloroform-*d*, 298 K) δ 155.47, 150.83, 129.29 (d, *J* = 14.4 Hz), 128.31 (d, *J* = 2.1 Hz), 125.29, 120.73 (qd, *J* = 289.3, 28.0 Hz, CF(<u>C</u>F$_3$)$_2$), 120.14 (d, *J* = 21.1 Hz), 94.55 - 90.40 (m, <u>C</u>F(CF$_3$)$_2$).

^{19}**F NMR** (376 MHz, Chloroform-*d*, 298 K) δ -73.86 (d, *J* = 6.5 Hz, CF(C<u>F</u>$_3$)$_2$, 6F), -176.58 (s, C<u>F</u>(CF$_3$)$_2$, 1F).

GC-MS: 304

HRMS (ESI) m/z [M+H]$^+$: Calculated for $C_9H_4F_7N_2S^+$: 304.9983. Found: 304.9979.

Melt Point: 64.4 - 67.1 °C

Gram scale experiments

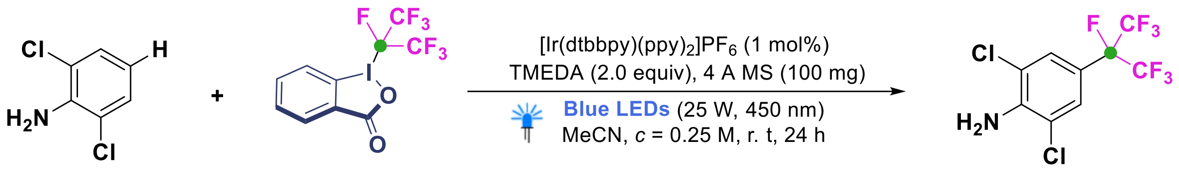

1e, 10 mmol, 1.62 g **2** **49**, 83% yield

Under an ambient atmosphere, in a 250 mL screw-cap vial equipped with a magnetic stirring bar, 2,6-dichloroaniline (**1e**, 10.0 mmol, 1.62 g, 1.0 equiv.), *i*-C_3F_7-iodine(III) (6.24 g, 15.0 mmol, 1.5 equiv), [Ir(dtbbpy)(ppy)$_2$]PF_6 (0.09 g, 1.0 mol%), were dissolved in dry MeCN (40 mL). Subsequently, 4 A molecular sieves (100 mg) and TMEDA (2.99 mL, 20.0 mmol, 2.0 equiv) was added into the vial. The vial was sealed and the reaction was irradiated at 450 nm for 16 h, the temperature of the reaction systems was controlled within 25 - 35 °C by using the drum fan. After this, the reaction was quenched with 50 mL 5% $NaHCO_3$ aqueous solution and the MeCN was removed by rotary evaporation, then extracted with DCM (3 x 50 mL). The combined organic phase was dried with anhydrous Na_2SO_4 and concentrated under vacuum. The residue was further purified by column chromatography to afford 2,6-dichloro-4-*iso*-perfluoropropyl-aniline (**49**, 2.74 g, 83% yield).

Variable-temperature ^{1}H NMR and ^{19}F NMR experiments

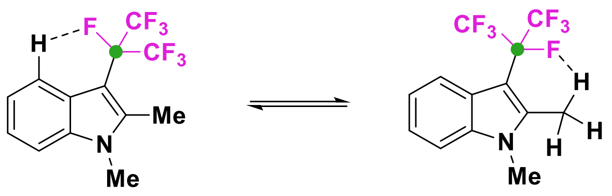

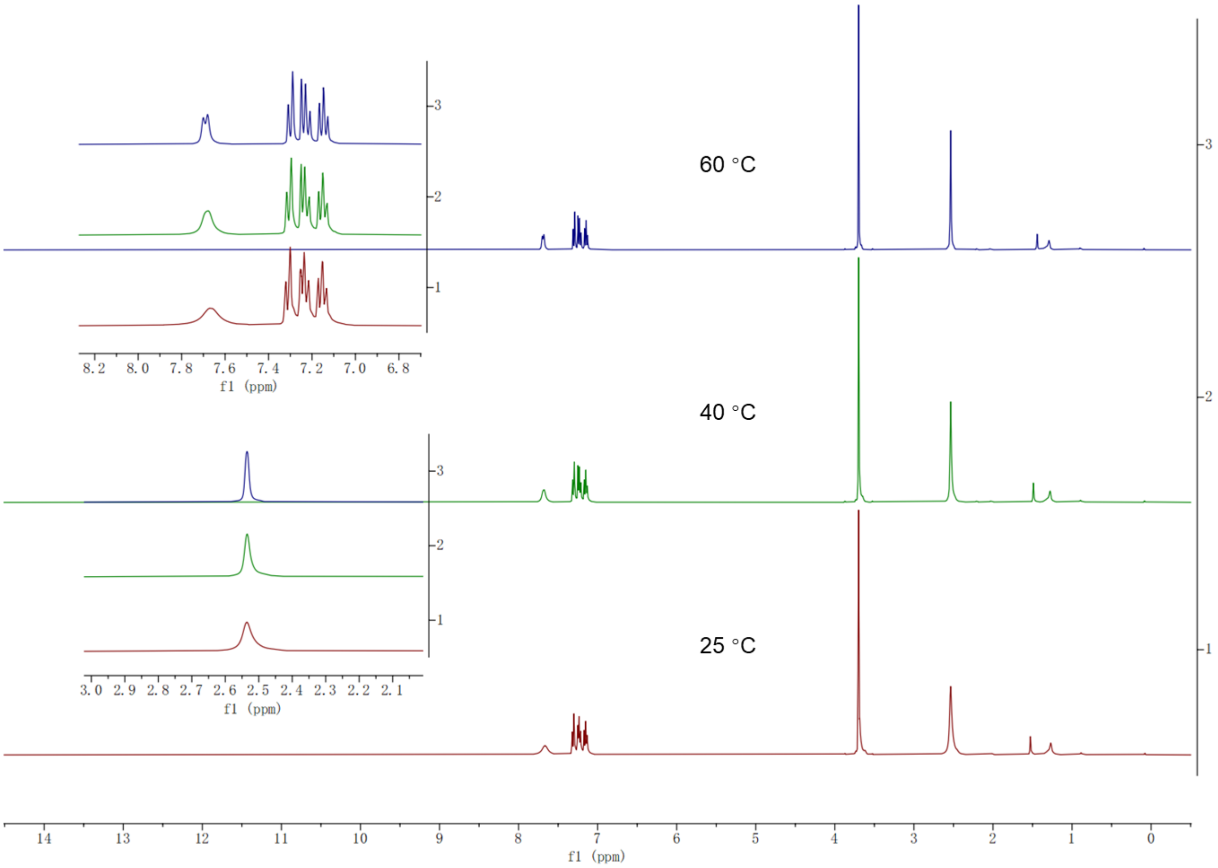

Figure S10. Variable-temperature ^{1}H NMR spectrum.

Variable-temperature ^{19}F NMR spectrum

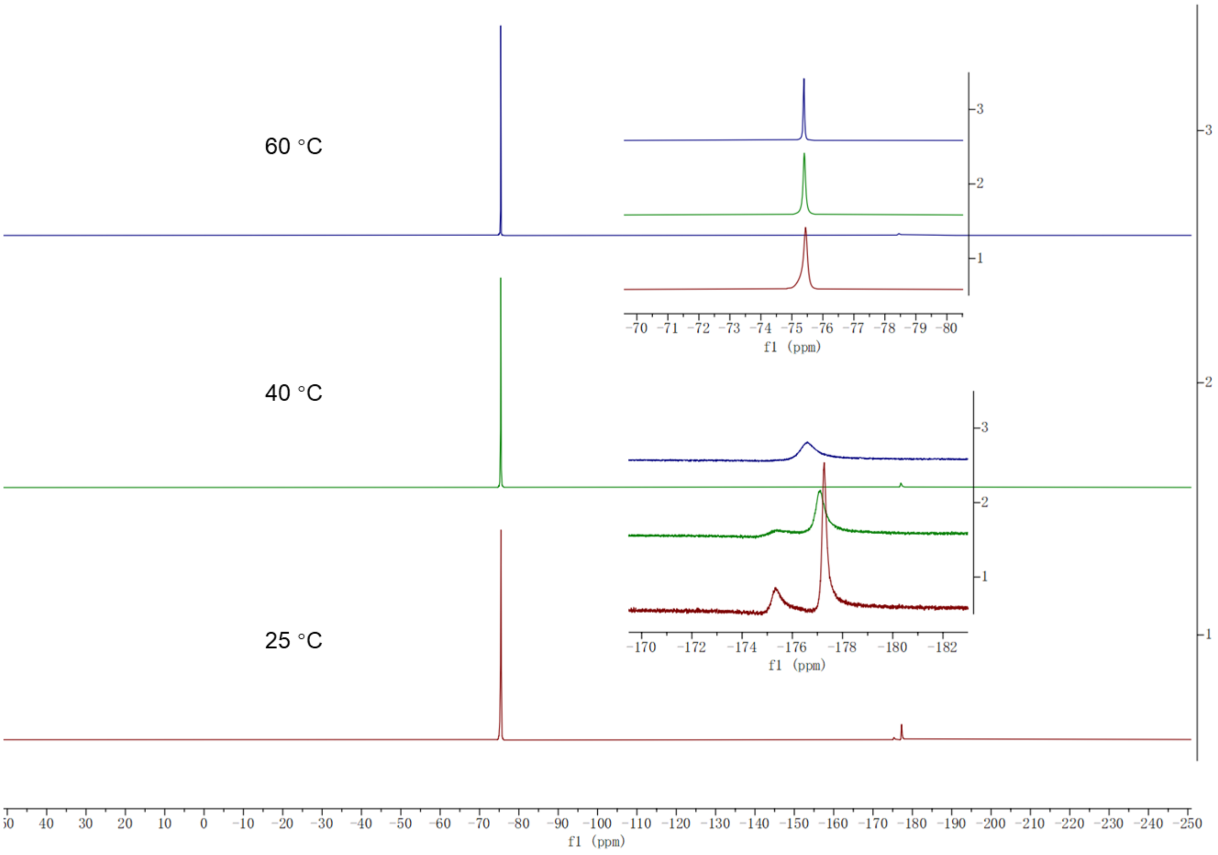

Figure S11. Variable-temperature ^{19}F NMR spectrum.

X-Ray Crystal Structures

Crystal Structure of $C_{13}H_{10}F_7N$

The low temperature [100(2)°K] single-crystal X-ray experiments were performed on a Rigaku diffractometer with Cu K_{α} radiation. Unit cell was obtained and refined by 7179 reflections with 4,2° < θ < 62.5°. No decay was observed in data collection. Raw intensities were corrected for Lorentz and polarization effects, and for absorption by empirical method. Direct phase determination yielded the positions of all non-hydrogen atoms. All non-hydrogen atoms were subjected to anisotropic refinement. The hydrogen atoms were generated geometrically with C-H bonds of 0.93-0.96 Å according to criteria described in the SHELXTL manual (Bruker, 1997). They were included in the refinement with $U_{iso}(H) = 1.2U_{eq}$ or $1.5U_{eq}$ (for methyl C) of their parent atoms. The final full-matric least-square refinement on F^2 converged with $R1 = 0.1224$ and $wR2 = 0.2305$ for 1732 observed reflections [$I \geq 2\sigma(I)$]. The final difference electron density map shows no features. Details of crystal parameters, data collection and structure refinement are given in Table 1.

Data collection was controlled by CrysAlis^{Pro} (Rigaku, 2016). Computations were performed using the SHELXTL NT ver. 5.10 program package (Bruker, 1997) on an IBM PC 586 computer. Analytic expressions of atomic scattering factors were employed, and anomalous dispersion corrections were incorporated (*International Tables for X-ray Crystallography*, 1989). Crystal drawings were produced with XP (Bruker, 1997).

The sample was refined to a low resolution and still has a large R factor and residual electron density which is high. There is a poor data/parameter ratio and large SHELXL weighting. This could be due to the sample being a small needle.

References

Bruker. (1997) SHELXTL. Structure Determination Programs, Version 5.10, Bruker AXS Inc.,6300 Enterprise Lane, Madison, WI 53719-1173, USA.

International Tables for X-ray Crystallography: (1989) Vol. C (Kluwer Academic Publishers, Dordrecht) Tables 4.2.6.8 and 6.1.1.4.

Rigaku. (2016) CrysAlis^{Pro}, Data Collection and Process Software for Rigaku Oxford Diffraction X-ray Diffractometer, Version 5.4, February, 2016. Rigaku Corporation, 9009, New Trails Drive, The Woodlands, TX77381, USA.

Table S11. Details of Data Collection, Processing and Structure Refinement

Sample code	**5**		
CCDC no	2214038		
Molecular formula	$C_{13}H_{10}F_7N$		
Molecular weight	313.22		
Color and habit	colorless needle		
Crystal size	0.05 × 0.05 × 0.20 mm		
Crystal system	monoclinic		
Space group	$P2_1$ (No. 4)		
Unit cell parameters	a = 8.6928(3) Å	α = 90.00°	
	b = 6.9961(3) Å	β = 90.258(3)°	
	c = 10.3356(4) Å	γ = 90.00°	
	V = 628.56(4) Å^3	Z = 2	F(000) = 316
Density (calcd)	1.655 g/cm^3		
Diffractometer	XtaLAB AFC11 (RINC): quarter-chi single		
Radiation	Cu K$_\alpha$, λ = 1.54178 Å		
Temperature	100(2)°K		
Scan type	ω-scan		
Data collection range	$-9 < h < 9$, $-8 < k < 8$, $-11 < l < 11$; θ_{max} = 63.0°		
Reflections measured	Total: 13695	Unique (n): 1996	Observed [I ≥ 2σ(I)]: 1732
Absorption coefficient	1.538 mm^{-1}		
Minimum and maximum transmission	0.612, 1.000		
No. of variables, p	192		
Weighting scheme	$w = \dfrac{1}{\sigma^2(F_o^2) + (0.001P)^2 + 3.5P}$	$P = (F_o^2 + 2F_c^2)/3$	
$R1 = \dfrac{\Sigma\|\|F_o\|-\|F_c\|\|}{\Sigma\|F_o\|}$ (for all reflections)	0.1370	0.1224 (for observed data)	
$wR2 = \sqrt{\dfrac{\Sigma[w(F_o^2 - F_c^2)^2]}{\Sigma w(F_o^2)^2}}$ (for all reflections)	0.2385	0.2305 (for observed data)	
Goof = S = $\sqrt{\dfrac{\Sigma[w(F_o^2 - F_c^2)^2]}{n - p}}$	1.252		
Largest and mean Δ/σ	0.018, 0.001		
Residual extrema in final difference map	-0.669 to 1.551 e Å^{-3}		

Table S12. Atomic coordinates and equivalent isotropic temperature factors* (Å^2)

Atoms	x	y	z	$U_{eq.}$
F(1)	0.4025(9)	0.444(4)	0.9442(8)	0.139(5)
F(2)	0.1461(15)	0.1637(17)	0.8006(9)	0.115(5)
F(3)	0.1598(12)	0.2338(17)	0.9996(9)	0.090(4)
F(4)	0.3636(14)	0.1193(18)	0.8875(10)	0.110(4)
F(5)	0.1657(12)	0.6267(18)	0.9966(7)	0.085(4)
F(6)	0.0422(14)	0.516(3)	0.8176(10)	0.169(8)
F(7)	0.198(2)	0.7431(18)	0.8190(13)	0.171(7)
N(1)	0.5436(9)	0.432(2)	0.5604(8)	0.043(2)
C(1)	0.5210(12)	0.431(3)	0.6917(10)	0.046(3)
C(2)	0.3636(11)	0.432(2)	0.7149(9)	0.043(2)
C(3)	0.2869(11)	0.435(2)	0.5887(9)	0.039(2)
C(4)	0.1342(11)	0.435(3)	0.5449(10)	0.045(3)
C(5)	0.1053(12)	0.430(3)	0.4164(10)	0.048(3)
C(6)	0.2236(13)	0.431(3)	0.3244(10)	0.052(3)
C(7)	0.3755(12)	0.435(2)	0.3649(9)	0.044(2)
C(8)	0.4050(11)	0.429(2)	0.4955(9)	0.040(2)
C(9)	0.6934(12)	0.427(3)	0.4974(11)	0.051(3)
C(10)	0.6596(12)	0.441(3)	0.7795(11)	0.058(3)
C(11)	0.2968(12)	0.431(2)	0.8464(10)	0.057(3)
C(12)	0.2402(16)	0.2363(19)	0.8870(12)	0.079(5)
C(13)	0.1771(18)	0.5761(18)	0.8729(12)	0.090(5)

*$U_{eq.}$ defined as one third of the trace of the orthogonalized **U** tensor.

Table S13. Bond lengths (Å) and bond angles (º)

F(1)-C(11)	1.365(10)	C(1)-C(10)	1.507(14)
F(2)-C(12)	1.311(12)	C(2)-C(3)	1.462(14)
F(3)-C(12)	1.360(11)	C(2)-C(11)	1.481(14)
F(4)-C(12)	1.350(12)	C(3)-C(4)	1.401(14)
F(5)-C(13)	1.330(11)	C(3)-C(8)	1.411(12)
F(6)-C(13)	1.368(13)	C(4)-C(5)	1.351(14)
F(7)-C(13)	1.308(12)	C(5)-C(6)	1.404(14)
N(1)-C(1)	1.372(13)	C(6)-C(7)	1.383(15)
N(1)-C(8)	1.377(13)	C(7)-C(8)	1.373(14)
N(1)-C(9)	1.458(12)	C(11)-C(13)	1.481(12)
C(1)-C(2)	1.391(14)	C(11)-C(12)	1.508(12)
C(1)-N(1)-C(8)	110.7(8)	F(1)-C(11)-C(13)	106.8(14)
C(1)-N(1)-C(9)	125.0(9)	F(1)-C(11)-C(2)	114.5(9)
C(8)-N(1)-C(9)	124.3(9)	C(13)-C(11)-C(2)	116.4(11)
N(1)-C(1)-C(2)	108.4(9)	F(1)-C(11)-C(12)	94.2(13)
N(1)-C(1)-C(10)	118.6(9)	C(13)-C(11)-C(12)	109.7(10)
C(2)-C(1)-C(10)	132.9(10)	C(2)-C(11)-C(12)	113.0(12)
C(1)-C(2)-C(3)	106.9(8)	F(2)-C(12)-F(4)	105.1(13)
C(1)-C(2)-C(11)	123.2(9)	F(2)-C(12)-F(3)	104.9(11)
C(3)-C(2)-C(11)	129.8(9)	F(4)-C(12)-F(3)	113.6(12)
C(4)-C(3)-C(8)	118.0(9)	F(2)-C(12)-C(11)	111.3(11)
C(4)-C(3)-C(2)	135.7(9)	F(4)-C(12)-C(11)	106.8(11)
C(8)-C(3)-C(2)	106.2(8)	F(3)-C(12)-C(11)	114.8(11)
C(5)-C(4)-C(3)	119.4(9)	F(7)-C(13)-F(5)	100.5(12)
C(4)-C(5)-C(6)	122.1(10)	F(7)-C(13)-F(6)	102.5(15)
C(7)-C(6)-C(5)	119.8(10)	F(5)-C(13)-F(6)	114.6(12)
C(8)-C(7)-C(6)	118.1(9)	F(7)-C(13)-C(11)	115.7(14)
C(7)-C(8)-N(1)	129.6(9)	F(5)-C(13)-C(11)	114.5(12)
C(7)-C(8)-C(3)	122.5(9)	F(6)-C(13)-C(11)	108.4(11)
N(1)-C(8)-C(3)	107.7(8)		

Table S14. Anisotropic thermal parameters* (Å^2)

Atoms	U_{11}	U_{22}	U_{33}	U_{23}	U_{13}	U_{12}
F(1)	0.051(5)	0.301(17)	0.064(5)	0.001(12)	0.000(4)	-0.031(11)
F(2)	0.163(12)	0.136(10)	0.047(5)	0.011(6)	0.010(6)	-0.109(9)
F(3)	0.099(8)	0.125(10)	0.045(6)	0.015(6)	0.024(5)	-0.027(7)
F(4)	0.115(9)	0.131(10)	0.083(7)	0.036(7)	0.038(6)	0.015(8)
F(5)	0.086(7)	0.142(10)	0.028(5)	-0.012(5)	0.015(4)	0.036(6)
F(6)	0.117(8)	0.32(2)	0.068(6)	-0.056(10)	-0.040(6)	0.102(11)
F(7)	0.267(17)	0.121(11)	0.127(12)	0.033(9)	0.130(11)	0.050(12)
N(1)	0.030(4)	0.060(6)	0.039(5)	-0.008(7)	0.006(3)	0.002(6)
C(1)	0.040(6)	0.059(7)	0.038(6)	0.016(8)	0.003(4)	0.008(8)
C(2)	0.040(5)	0.055(6)	0.033(5)	-0.003(8)	0.008(4)	-0.003(8)
C(3)	0.031(5)	0.054(6)	0.034(5)	0.006(7)	0.007(4)	-0.004(7)
C(4)	0.031(5)	0.066(7)	0.039(5)	0.003(8)	0.006(4)	0.010(8)
C(5)	0.035(5)	0.062(7)	0.048(6)	0.007(9)	0.004(5)	0.010(8)
C(6)	0.057(7)	0.061(7)	0.039(6)	-0.012(8)	0.003(5)	-0.009(9)
C(7)	0.042(6)	0.056(7)	0.034(5)	0.000(8)	0.012(4)	-0.012(8)
C(8)	0.032(5)	0.052(6)	0.036(5)	0.013(8)	0.011(4)	0.007(8)
C(9)	0.036(6)	0.054(7)	0.063(7)	0.019(9)	0.020(5)	-0.001(8)
C(10)	0.045(6)	0.075(9)	0.053(7)	-0.010(9)	-0.007(5)	0.007(9)
C(11)	0.049(6)	0.082(9)	0.041(6)	0.028(9)	0.006(5)	-0.003(9)
C(12)	0.110(11)	0.076(9)	0.052(8)	0.003(7)	0.034(7)	-0.012(9)
C(13)	0.132(14)	0.080(12)	0.059(11)	0.009(9)	0.035(9)	0.021(12)

*The exponent takes the form: $-2\pi^2\Sigma\Sigma U_{ij}h_ih_j\mathbf{a}_i^*\mathbf{a}_j^*$

Table S15. Coordinates and isotropic temperature factors* (Å^2) for H atoms

Atoms	*x*	*y*	*z*	$U_{eq.}$
H(4)	0.0536	0.4373	0.6037	0.054
H(5)	0.0036	0.4263	0.3879	0.058
H(6)	0.2000	0.4290	0.2366	0.063
H(7)	0.4553	0.4403	0.3054	0.053
H(9A)	0.7537	0.3245	0.5331	0.076
H(9B)	0.7455	0.5461	0.5114	0.076
H(9C)	0.6795	0.4065	0.4062	0.076
H(10A)	0.6877	0.5720	0.7933	0.086
H(10B)	0.7439	0.3742	0.7402	0.086
H(10C)	0.6356	0.3823	0.8610	0.086

*The exponent takes the form: $-8\pi^2 U\sin^2\theta/\lambda^2$

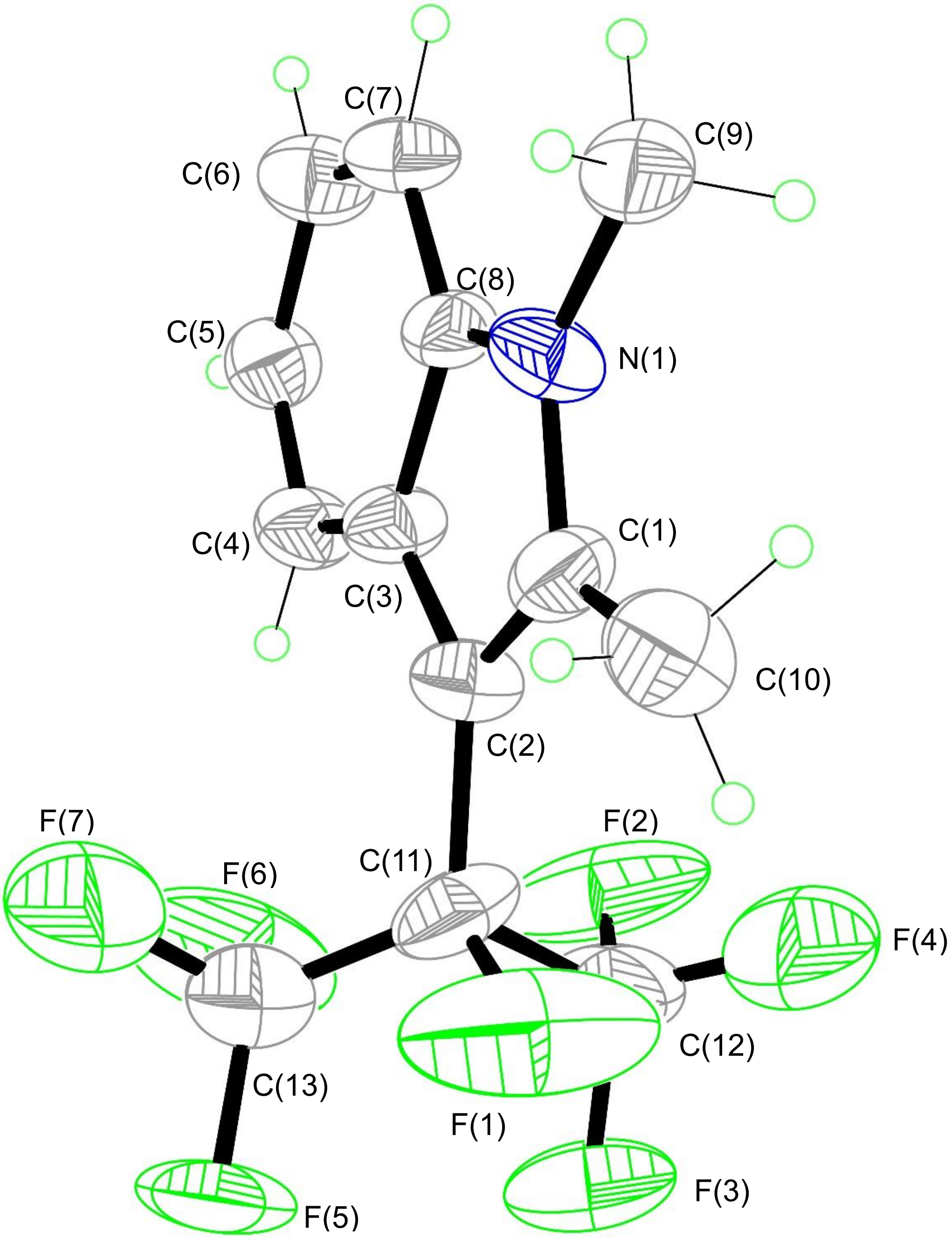

Figure S12. ORTEP drawing of $C_{13}H_{10}F_7N$ with 50% probability ellipsoids, showing the atomic numbering scheme.

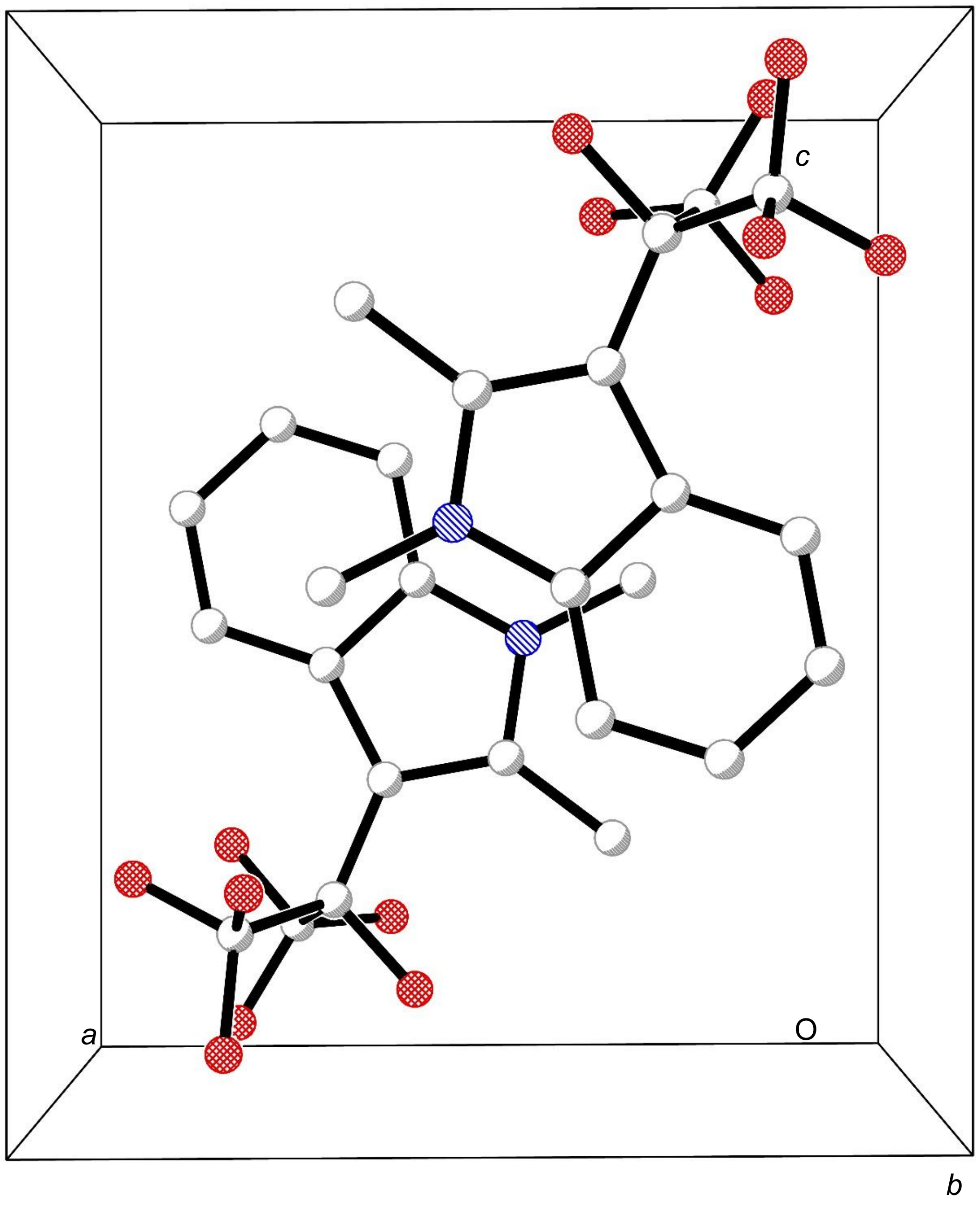

Figure S13. A packing view along the *b* direction

Crystal Structure of $C_{14}H_{10}F_7NO_2$

The room temperature (292±2°K) single-crystal X-ray experiments were performed on a SuperNova diffractometer with Cu K_α radiation. Unit cell was obtained and refined by 934 reflections with 4.8° < θ < 71.4°. No decay was observed in data collection. Raw intensities were corrected for Lorentz and polarization effects, and for absorption by empirical method. Direct phase determination yielded the positions of all non-hydrogen atoms. All non-hydrogen atoms were subjected to anisotropic refinement. All hydrogen atoms were generated geometrically with C-H bonds of 0.93-0.96 Å according to criteria described in the SHELXTL manual (Bruker, 1997). They were included in the refinement with $U_{iso}(H) = 1.2U_{eq}$ of their parent atoms. The final full-matric least-square refinement on F^2 converged with $R1 = 0.1089$ and $wR2 = 0.1678$ for 1343 observed reflections [$I \geq 2\sigma(I)$]. The final difference electron density map shows no features. Details of crystal parameters, data collection and structure refinement are given in Table 1.

Data collection was controlled by CrysAlisPro, Agilent Technologies, Version 1.171.36.32 (Oxford, 2013). Computations were performed using the SHELXTL NT ver. 5.10 program package (Bruker, 1997) on an IBM PC 586 computer. Analytic expressions of atomic scattering factors were employed, and anomalous dispersion corrections were incorporated (*International Tables for X-ray Crystallography*, 1989). Crystal drawings were produced with XP (Bruker, 1997).

References

Bruker. (1997) SHELXTL. Structure Determination Programs, Version 5.10, Bruker AXS Inc.,6300 Enterprise Lane, Madison, WI 53719-1173, USA.

International Tables for X-ray Crystallography: (1989) Vol. C (Kluwer Academic Publishers, Dordrecht) Tables 4.2.6.8 and 6.1.1.4.

Oxford. (2013) CrysAlisPro, Agilent Technologies, Version 1.171.36.32, Oxford Diffraction Ltd., 68 Milton Park, Abingdon, Oxfordshire, OX14 4RX, UK.

Table S16. Details of Data Collection, Processing and Structure Refinement

Sample code	**8**
CCDC no	2214040
Molecular formula	$C_{14}H_{10}F_7NO_2$
Molecular weight	357.23
Color and habit	colorless plate
Crystal size	0.1 × 0.2 × 0.4 mm
Crystal system	orthorhombic
Space group	*Pna*2$_1$/*n* (No. 33)
Unit cell parameters	a = 21.2544(14) Å α = 90.00°<br>b = 9.9719(7) Å β = 90.00°<br>c = 7.0300(5) Å γ = 90.00°<br>V = 1489.99(18) Å^3 Z = 4 F(000) = 720
Density (calcd)	1.592 g/cm^3
Diffractometer	SuperNova, Dual, Cu at home/near, AtlasS2
Radiation	Cu K_{α}, λ = 1.54178 Å
Temperature	292±2K
Scan type	ω-scan
Data collection range	$-26 < h < 24$, $-8 < k < 11$, $-8 < l < 4$; θ_{max} = 74.1°
Reflections measured	Total: 3130 Unique (n): 1908 Observed [I ≥ 2σ(I)]: 1343
Absorption coefficient	1.485 mm^{-1}
Minimum and maximum transmission	0.581, 1.000
No. of variables, p	220
Weighting scheme	$w = \dfrac{1}{\sigma^2(F_o^2) + (0.001P)^2 + 4.5P}$ $P = (F_o^2 + 2F_c^2)/3$
$R1 = \dfrac{\Sigma\|\|F_o\|-\|F_c\|\|}{\Sigma\|F_o\|}$ (for all reflections)	0.1416 0.1089 (for observed data)
$wR2 = \sqrt{\dfrac{\Sigma[w(F_o^2 - F_c^2)^2]}{\Sigma w(F_o^2)^2}}$ (for all reflections)	0.1842 0.1678 (for observed data)
Goof = S = $\sqrt{\dfrac{\Sigma[w(F_o^2 - F_c^2)^2]}{n - p}}$	1.178
Largest and mean Δ/σ	0.004, 0.001
Residual extrema in final difference map	-0.549 to 0.808 e Å^{-3}

Table S17. Atomic coordinates and equivalent isotropic temperature factors* (Å^2)

Atoms	*x*	*y*	*z*	$U_{eq.}$
F(1)	0.2750(4)	0.5321(10)	0.467(2)	0.203(6)
F(2)	0.3207(6)	0.7337(15)	0.0889(18)	0.177(6)
F(3)	0.2311(5)	0.6872(19)	0.181(3)	0.278(12)
F(4)	0.2955(6)	0.5228(13)	0.107(2)	0.225(8)
F(5)	0.3177(7)	0.715(3)	0.6816(15)	0.277(12)
F(6)	0.3310(4)	0.8527(8)	0.397(3)	0.208(7)
F(7)	0.2381(7)	0.758(2)	0.526(3)	0.307(10)
O(1)	0.5717(3)	0.1086(5)	0.385(2)	0.096(3)
O(2)	0.4863(3)	-0.0219(6)	0.384(2)	0.100(3)
N(1)	0.4864(3)	0.5858(6)	0.383(2)	0.0625(19)
C(1)	0.4338(4)	0.6634(8)	0.388(3)	0.067(2)
C(2)	0.3813(4)	0.5849(8)	0.386(2)	0.062(2)
C(3)	0.4018(4)	0.4487(8)	0.390(2)	0.058(2)
C(4)	0.3725(4)	0.3237(8)	0.391(2)	0.065(2)
C(5)	0.4084(4)	0.2088(8)	0.390(3)	0.068(2)
C(6)	0.4742(4)	0.2159(8)	0.391(2)	0.062(2)
C(7)	0.5052(4)	0.3380(7)	0.390(2)	0.059(2)
C(8)	0.4681(4)	0.4517(7)	0.385(2)	0.0554(19)
C(9)	0.5512(4)	0.6329(8)	0.383(3)	0.070(2)
C(10)	0.3136(4)	0.6306(10)	0.3962(19)	0.086(3)
C(11)	0.2903(5)	0.6430(13)	0.1923(18)	0.110(5)
C(12)	0.3019(8)	0.7589(16)	0.506(2)	0.181(9)
C(13)	0.5099(4)	0.0887(8)	0.383(3)	0.070(2)
C(14)	0.6105(5)	-0.0086(9)	0.388(5)	0.125(6)

*$U_{eq.}$ defined as one third of the trace of the orthogonalized **U** tensor.

Table S18. Bond lengths (Å) and bond angles (º)

F(1)-C(10)	1.374(9)	C(1)-C(2)	1.364(11)
F(2)-C(11)	1.329(10)	C(2)-C(3)	1.427(11)
F(3)-C(11)	1.336(10)	C(2)-C(10)	1.511(12)
F(4)-C(11)	1.346(10)	C(3)-C(4)	1.394(10)
F(5)-C(12)	1.353(11)	C(3)-C(8)	1.411(10)
F(6)-C(12)	1.356(10)	C(4)-C(5)	1.376(11)
F(7)-C(12)	1.366(11)	C(5)-C(6)	1.401(10)
O(1)-C(13)	1.329(10)	C(6)-C(7)	1.384(10)
O(1)-C(14)	1.431(8)	C(6)-C(13)	1.479(11)
O(2)-C(13)	1.211(9)	C(7)-C(8)	1.380(11)
N(1)-C(1)	1.359(10)	C(10)-C(12)	1.513(11)
N(1)-C(8)	1.392(9)	C(10)-C(11)	1.521(10)
N(1)-C(9)	1.457(9)		
C(13)-O(1)-C(14)	116.7(7)	F(1)-C(10)-C(12)	108.8(12)
C(1)-N(1)-C(8)	108.6(6)	C(2)-C(10)-C(12)	115.7(11)
C(1)-N(1)-C(9)	126.4(6)	F(1)-C(10)-C(11)	101.9(12)
C(8)-N(1)-C(9)	125.0(7)	C(2)-C(10)-C(11)	106.9(11)
N(1)-C(1)-C(2)	110.2(7)	C(12)-C(10)-C(11)	111.0(11)
C(1)-C(2)-C(3)	107.2(7)	F(2)-C(11)-F(3)	101.6(12)
C(1)-C(2)-C(10)	127.4(8)	F(2)-C(11)-F(4)	108.7(15)
C(3)-C(2)-C(10)	125.3(7)	F(3)-C(11)-F(4)	110.1(15)
C(4)-C(3)-C(8)	117.7(7)	F(2)-C(11)-C(10)	114.3(11)
C(4)-C(3)-C(2)	135.7(8)	F(3)-C(11)-C(10)	113.0(12)
C(8)-C(3)-C(2)	106.6(7)	F(4)-C(11)-C(10)	108.8(12)
C(5)-C(4)-C(3)	119.8(7)	F(5)-C(12)-F(6)	128.7(19)
C(4)-C(5)-C(6)	120.8(7)	F(5)-C(12)-F(7)	98.5(15)
C(7)-C(6)-C(5)	121.3(8)	F(6)-C(12)-F(7)	121.0(17)
C(7)-C(6)-C(13)	120.7(7)	F(5)-C(12)-C(10)	98.7(13)
C(5)-C(6)-C(13)	117.9(7)	F(6)-C(12)-C(10)	102.9(10)
C(8)-C(7)-C(6)	116.8(7)	F(7)-C(12)-C(10)	102.3(13)
C(7)-C(8)-N(1)	129.1(7)	O(2)-C(13)-O(1)	123.0(8)
C(7)-C(8)-C(3)	123.6(7)	O(2)-C(13)-C(6)	124.7(8)
N(1)-C(8)-C(3)	107.3(7)	O(1)-C(13)-C(6)	112.2(7)
F(1)-C(10)-C(2)	111.8(8)		

Table S19. Anisotropic thermal parameters* (Å^2)

Atoms	U_{11}	U_{22}	U_{33}	U_{23}	U_{13}	U_{12}
F(1)	0.069(4)	0.158(7)	0.381(19)	0.055(11)	0.034(8)	-0.016(5)
F(2)	0.137(9)	0.237(13)	0.156(10)	0.121(11)	-0.014(8)	-0.030(9)
F(3)	0.092(7)	0.44(3)	0.30(2)	0.24(2)	-0.012(10)	0.053(11)
F(4)	0.216(13)	0.183(11)	0.277(17)	-0.043(12)	-0.148(14)	0.027(9)
F(5)	0.187(15)	0.56(4)	0.080(7)	-0.052(16)	0.009(10)	0.106(18)
F(6)	0.118(6)	0.112(6)	0.39(2)	-0.019(16)	0.037(15)	0.005(5)
F(7)	0.117(9)	0.423(18)	0.38(2)	-0.147(16)	0.028(11)	0.083(11)
O(1)	0.070(4)	0.051(3)	0.166(8)	-0.028(7)	0.021(8)	-0.001(3)
O(2)	0.096(5)	0.054(3)	0.151(8)	-0.010(7)	-0.002(9)	-0.020(3)
N(1)	0.065(4)	0.050(3)	0.072(5)	-0.025(6)	0.012(6)	-0.018(3)
C(1)	0.080(5)	0.053(4)	0.069(6)	0.002(9)	-0.013(8)	0.001(4)
C(2)	0.063(4)	0.060(4)	0.064(5)	0.006(8)	0.001(8)	0.001(4)
C(3)	0.057(4)	0.065(4)	0.053(5)	-0.008(7)	-0.008(7)	-0.004(3)
C(4)	0.055(4)	0.067(5)	0.072(6)	-0.001(8)	-0.014(7)	-0.012(4)
C(5)	0.069(5)	0.064(5)	0.071(6)	-0.002(8)	0.001(9)	-0.018(4)
C(6)	0.065(5)	0.053(4)	0.069(6)	-0.016(7)	-0.014(8)	-0.012(3)
C(7)	0.055(4)	0.056(4)	0.067(6)	0.017(7)	0.008(7)	-0.008(3)
C(8)	0.066(4)	0.047(4)	0.053(5)	0.014(6)	-0.003(7)	-0.012(3)
C(9)	0.075(5)	0.062(5)	0.072(6)	-0.011(8)	0.005(9)	-0.020(4)
C(10)	0.075(5)	0.088(6)	0.096(7)	0.006(8)	-0.002(8)	0.006(5)
C(11)	0.093(7)	0.122(9)	0.115(9)	0.008(8)	-0.010(8)	0.007(8)
C(12)	0.169(13)	0.179(13)	0.196(14)	-0.003(10)	0.014(10)	0.008(10)
C(13)	0.076(5)	0.051(4)	0.082(7)	-0.009(8)	0.008(9)	-0.006(4)
C(14)	0.091(7)	0.062(6)	0.223(18)	0.031(15)	-0.004(16)	0.011(5)

*The exponent takes the form: $-2\pi^2\Sigma\Sigma U_{ij}h_ih_j\mathbf{a}_i^*\mathbf{a}_j^*$

Table S20. Coordinates and isotropic temperature factors* (Å^2) for H atoms

Atoms	*x*	*y*	*z*	$U_{eq.}$
H(1)	0.4338	0.7566	0.3917	0.081
H(4)	0.3288	0.3178	0.3927	0.078
H(5)	0.3887	0.1255	0.3890	0.081
H(7)	0.5489	0.3432	0.3921	0.071
H(9A)	0.5520	0.7275	0.4086	0.105
H(9B)	0.5700	0.6159	0.2612	0.105
H(9C)	0.5745	0.5864	0.4798	0.105
H(14A)	0.6529	0.0156	0.3556	0.188
H(14B)	0.5949	-0.0725	0.2977	0.188
H(14C)	0.6099	-0.0474	0.5131	0.188

*The exponent takes the form: $-8\pi^2 U\sin^2\theta/\lambda^2$

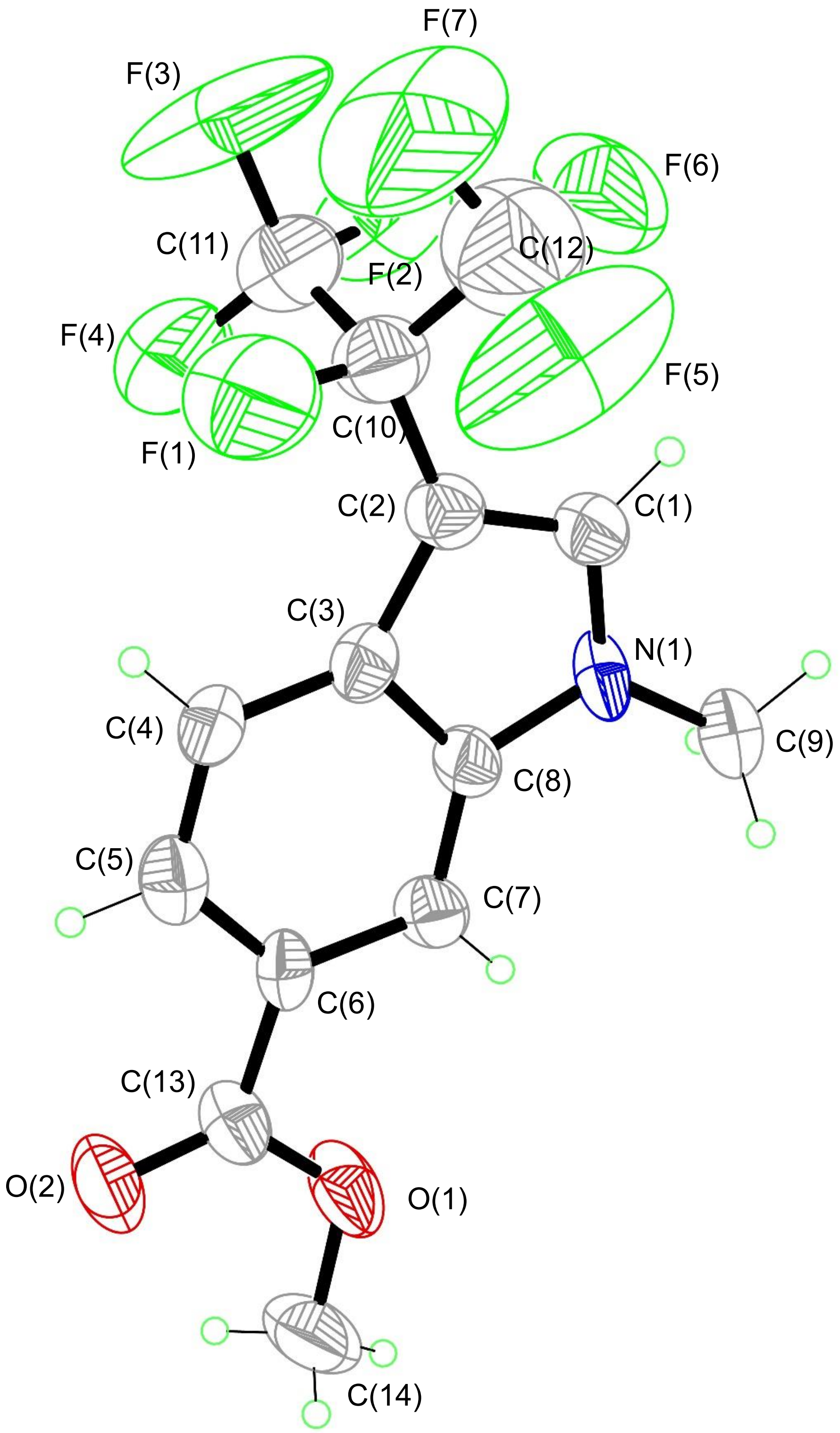

Figure S14. ORTEP drawing of $C_{14}H_{10}F_7NO_2$ with 50% probability ellipsoids, showing the atomic numbering scheme.

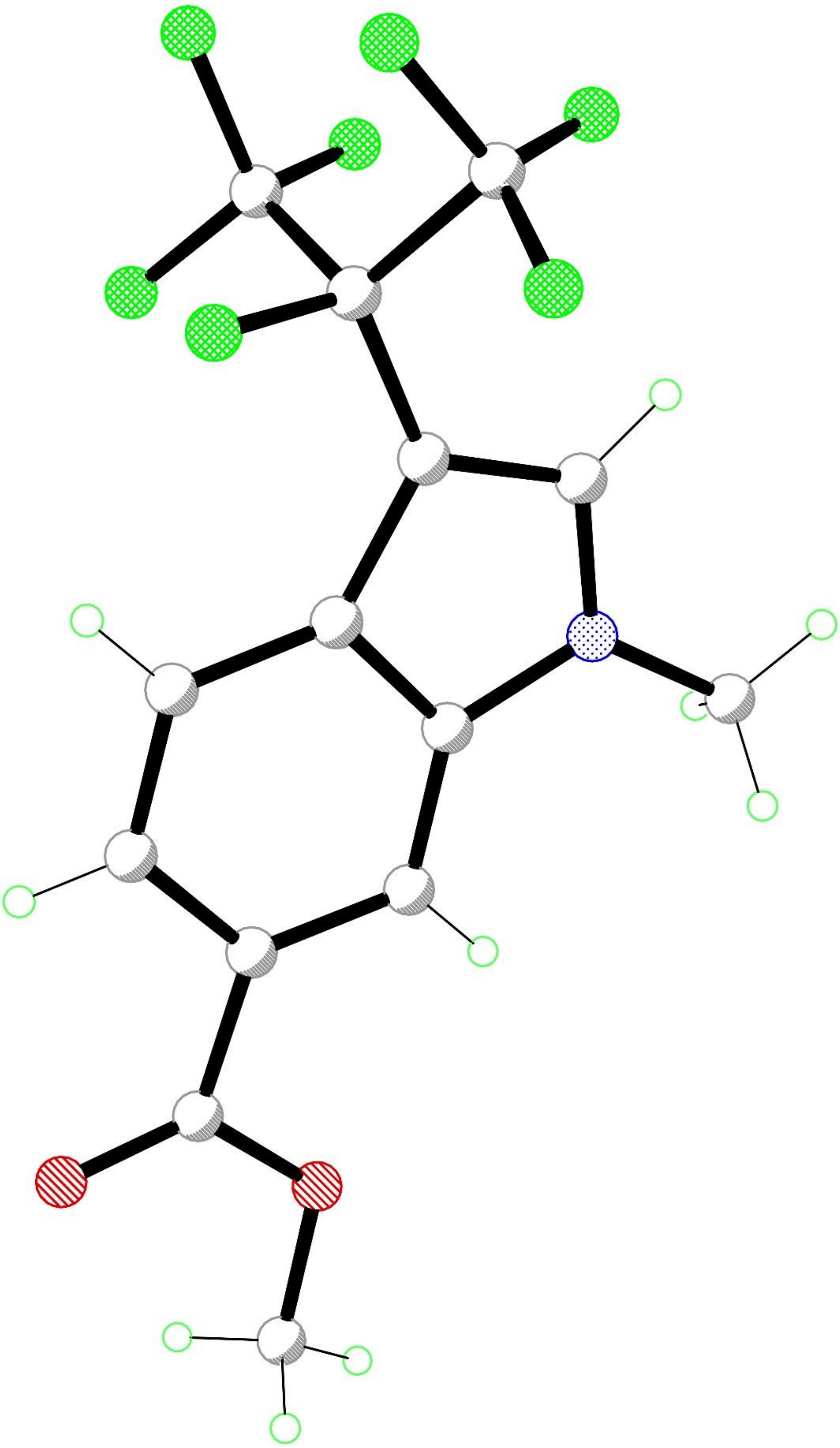

Figure S15. Drawing of ball and stick

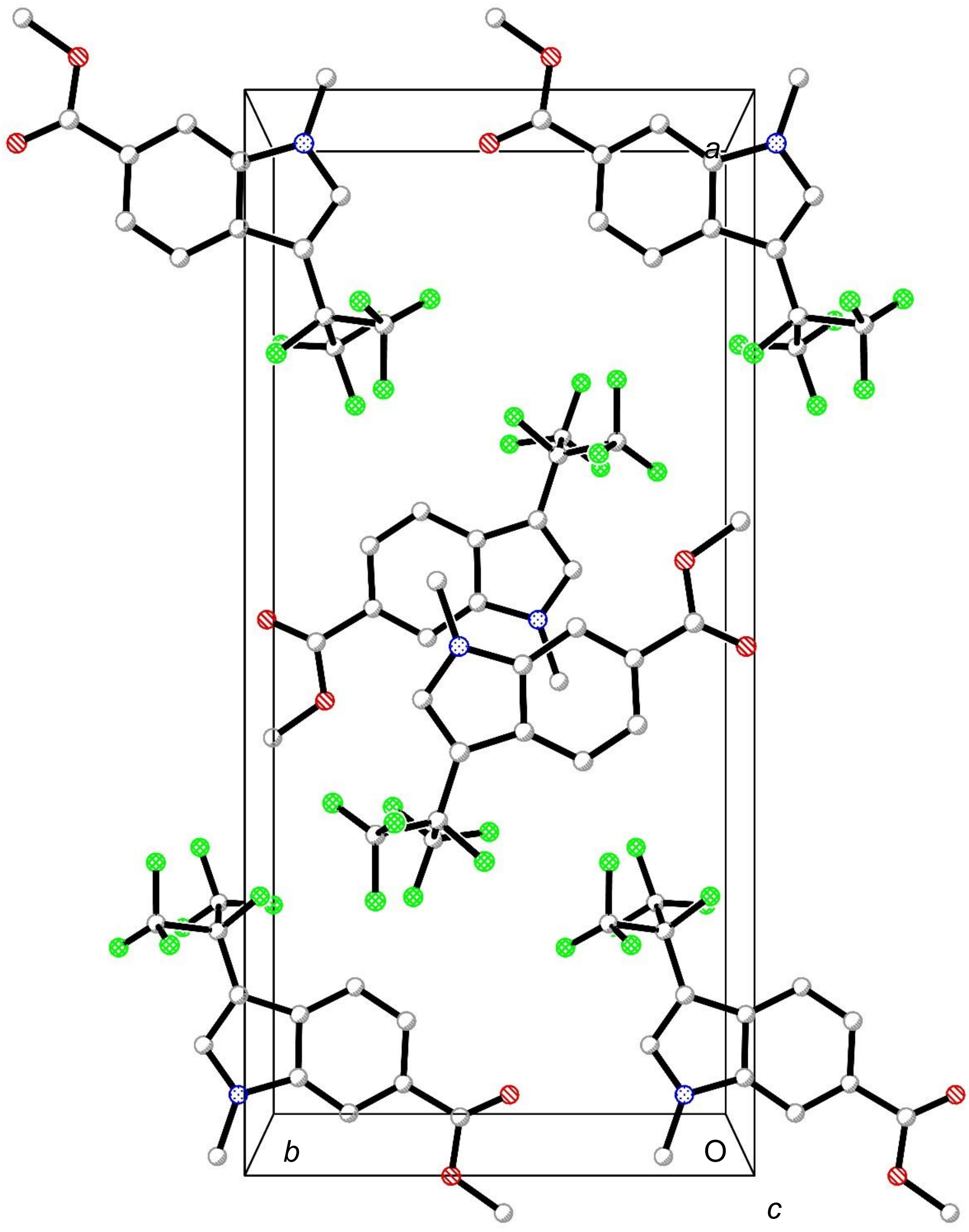

Figure S16. A packing view along the *c* direction

SUPPLEMENTARY REFERENCRS

1. Matoušek, V.; Pietrasiak, E.; Schwenk, R.; Togni, A. *J. Org. Chem.* 2013, **78**, 6763.
2. Borrel, J.; Waser. J. *Org. Lett.* 2022, **24**, 142.
3. Ren, J.; Du, F. H.; Jia, M. C.; Hu, Z. N.; Chen, Z.; Zhang, C. *Angew. Chem., Int. Ed.* 2021, **60**, 24171.
4. Frisch, M.; Trucks, J. G. W. Schlegel, H. B.; Scuseria, G. E.; Robb, M. A.; Cheeseman, J. R.; Scalmani, G.; Barone, V.; Mennucci, B.; Petersson, G. A.; Nakatsuji, H.; Caricato, M.; Li, X.; Hratchian, H. P.; Izmaylov, A. F.; Bloino, J.; Zheng, G.; Sonnenberg, J. L.; Had, M.; D. J. F. Gaussian 09. (2016).
5. Tao, J.; Perdew, J. P.; Staroverov, V. N.; Scuseria, G. E. *Phys. Rev. Lett.* 2003, **91**, 146401.
6. (a) Grimme, S.; Antony, J.; Ehrlich, S.; Krieg, H. *J. Chem. Phys.* 2010, **132**, 154104. (b) Grimme, S.; Ehrlich, S.; Goerigk, L. *J. Comput. Chem.* 2011, **32**, 1456.
7. (a) Weigend, F.; Häser, M.; Patzelt, H.; Ahlrichs, R. *Chem. Phys. Lett.* 1998, **294**, 143. (b) Weigend, F.; Ahlrichs, R. *Phys. Chem. Chem. Phys.* 2005, **7**, 3297.
8. Skyner, R. E.; McDonagh, J. L.; Groom, C. R.; Mourik, T. van.; Mitchell, J. B. O. *Phys. Chem. Chem. Phys.* 2015, **17**, 6174.
9. Jesser, A.; Rohrmuller, M.; Schmidt, W.; Herres-Pawlis, S. *J. Comput. Chem.* 2014, **35**, 1.
10. Grimme, S. *Chem. Eur. J.* 2012, **18**, 9955.
11. Qu, Z. W.; Hansen, A.; Grimme, S. *J. Chem. Theory Comput.* 2015, **11**, 1037.
12. ELANCO ANIMAL HEALTH INC - US2022/48847, 2022, A1.
13. Li, Y.; Wang, X.; Guo, Y.; Zhu, Z.; Wu, Y.; Gong, Y. *Chem. Commun.* 2016, **52**, 796.
14. Song, D.; Wang, C. M.; Ye, Z. P.; Xia, P. J.; Deng, Z. X.; Xiao, J. A.; Xiang, H. Y.; Yang, H. *J. Org. Chem.* 2019, **84**, 7480.
15. Hirata, G.; Shimada, T.; Nishikata, T. *Org. Lett.* 2020, **22**, 8952.
