# Supplementary material for: Direct electrophilic and radical isoperfluoropropylation with i-C3F7-Iodine(III) reagent (PFPI reagent)
Source: Commun Chem. 2023 Aug 24;6:177. doi: 10.1038/s42004-023-00986-3 (PMC10449889; doi:10.1038/s42004-023-00986-3)
Supplement: Supplementary file 2 — Description of Additional Supplementary Files [file 42004_2023_986_MOESM2_ESM.pdf]

Description of Additional Supplementary Files

File name: Supplementary Data 1

Description: X-Ray crystallography of compound 5

File name: Supplementary Data 2

Description: X-Ray crystallography of compound 8

File name: Supplementary Data 3

Description: Cartesian coordinates of the structures

File name: Supplementary Data 4

Description: NMR spectra
