# Supplementary material for: Direct electrophilic and radical isoperfluoropropylation with i-C3F7-Iodine(III) reagent (PFPI reagent)
Source: Commun Chem. 2023 Aug 24;6:177. doi: 10.1038/s42004-023-00986-3 (PMC10449889; doi:10.1038/s42004-023-00986-3)
Supplement: Supplementary file 5 — Supplementary Data 3 [file 42004_2023_986_MOESM5_ESM.docx]

**DFT calculations**

Computational Details

The quantum chemical DFT calculations have been performed with the Gaussian 16 program (for geometry optimization and numerical harmonic frequency calculations)^4^ and ORCA 5.0.3 program (for single point calculations). The structures are fully optimized at the TPSS-D3/def2-SVP + IEFPCM(MeCN) level of theory, which combines the TPSS meta-GGA density functional^5^ with the BJ-damped DFT-D3 dispersion correction^6^ and the def2-SVP basis set^7^, using the IEFPCM solvation model for MeCN solvent^8^. The triplet state T1 computed by TD-B3LYP/def2-TZVP + SMD level^9^. The optimized structures are characterized by frequency analysis to identify the nature of located stationary points (no imaginary frequency for true minima and only one imaginary frequency for transition state) and to provide thermal corrections (at 298.15 K and 1 atm) according to the modified ideal gas rigid rotor harmonic oscillator model^10^. Single-point calculations are performed at hybrid[1]meta-GGA PW6B95-D3/def2-TZVP + SMD(MeCN) level of theory^11^. The final reaction Gibbs free energies (ΔG) are determined from the electronic single-point energies plus TPSS-D3 thermal corrections. The density-fitting RI-J approach is used to accelerate the single point calculations in solution.

Cartesian Coordinates, Structures and Computed Gibbs Free Energies for the Optimized Structures and Transition States


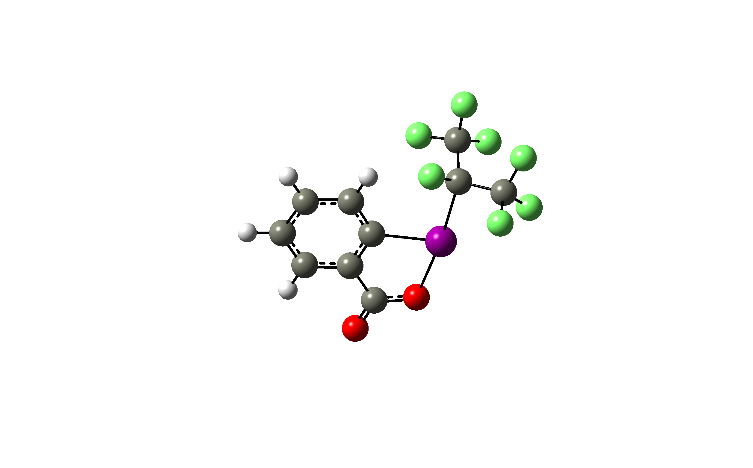


Charge: 0, Multiplicity: 1

# of imaginary frequency = 0

G_sol_: -1530.122687 kcal/mol

C 1.67891100 0.58712700 -0.21408500

C 1.38026700 1.90999100 -0.53605200

C 2.46527800 2.78941900 -0.69722000

C 3.78711000 2.34434700 -0.54065500

C 4.04144200 1.00703300 -0.22125600

C 2.97870100 0.10063700 -0.05316000

H 0.36212700 2.27045400 -0.66648800

H 2.25941700 3.83407100 -0.95021800

H 4.61754200 3.04468600 -0.66986100

H 5.05754100 0.62137000 -0.09174500

I 0.21425000 -0.97871800 0.09932000

C 3.24824700 -1.34533100 0.28688200

O 2.16711200 -2.08132200 0.41303300

O 4.39233500 -1.77498100 0.42755200

C -1.67669200 0.37331800 -0.27900300

F -1.49064100 1.29075300 -1.28055400

C -2.04208100 1.12900500 1.00869100

C -2.76137000 -0.61481700 -0.75111600

F -0.98530600 1.83834000 1.45849200

F -3.05306200 1.99327900 0.80680200

F -2.40439000 0.25572900 1.96813800

F -3.99447200 -0.07582100 -0.73524400

F -2.76975700 -1.70553600 0.05098100

F -2.49418300 -1.01146500 -2.00511100


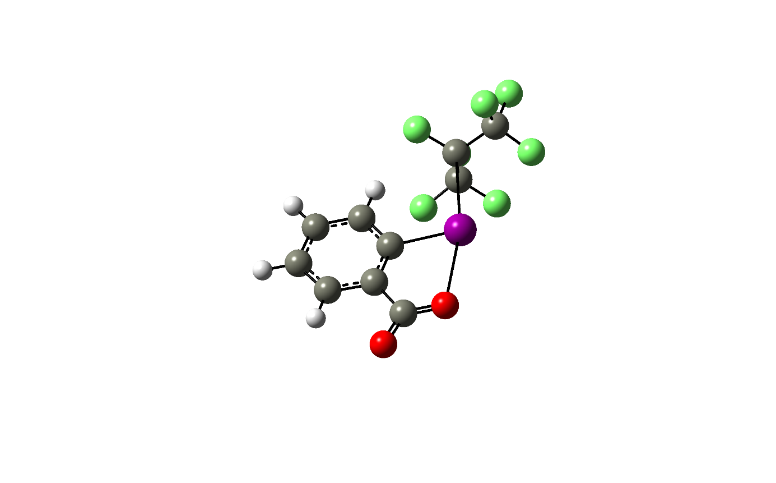


Charge: 1, Multiplicity: 2

# of imaginary frequency = 0

G_sol_: -1530.264805 kcal/mol

C 1.84722400 0.48342900 -0.23146200

C 1.28625800 1.72031500 -0.58663400

C 2.13330500 2.82078000 -0.78279000

C 3.52068900 2.67615400 -0.62432600

C 4.04962700 1.43019700 -0.27268000

C 3.22897900 0.30077700 -0.06714500

H 0.20694600 1.82747600 -0.71149500

H 1.70073100 3.78804900 -1.05996800

H 4.18482900 3.53365400 -0.77633700

H 5.12504900 1.27248800 -0.14049700

I 0.47081900 -1.13738000 0.06148200

C 3.91643900 -1.02580700 0.31180300

O 3.16356400 -2.03345600 0.47835500

O 5.16768200 -1.00250100 0.42361500

C -2.22199600 0.48555400 -0.26545400

F -2.07613600 1.51049300 -1.15605000

C -2.42030200 0.99254200 1.12510500

C -3.06914200 -0.61704100 -0.81609300

F -1.39025800 1.78703500 1.50696300

F -3.55002400 1.75394900 1.26759600

F -2.52131000 -0.02763600 2.00458100

F -4.40975300 -0.32802500 -0.83068100

F -2.93446400 -1.75193500 -0.09024900

F -2.72332100 -0.88324900 -2.09415400


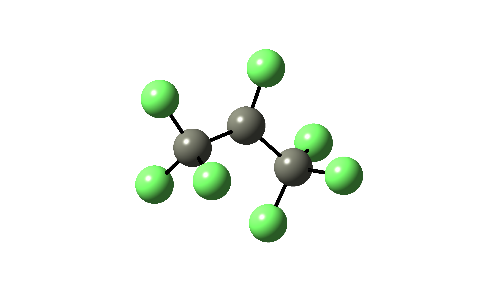


Charge: 0, Multiplicity: 2

# of imaginary frequency = 0

G_sol_: -813.1229855 kcal/mol

C 0.00358700 0.54669900 -0.20877500

F 0.02518500 1.86563800 -0.04491700

C 1.32948700 -0.13676200 -0.02449700

C -1.33581200 -0.11980800 -0.02560700

F 2.25163400 0.37700500 -0.86376200

F 1.80599600 0.02051400 1.23488500

F 1.20932600 -1.45668700 -0.26384000

F -1.48558100 -0.61698300 1.22998000

F -1.48389900 -1.15102100 -0.88090300

F -2.32083500 0.76811400 -0.23885800


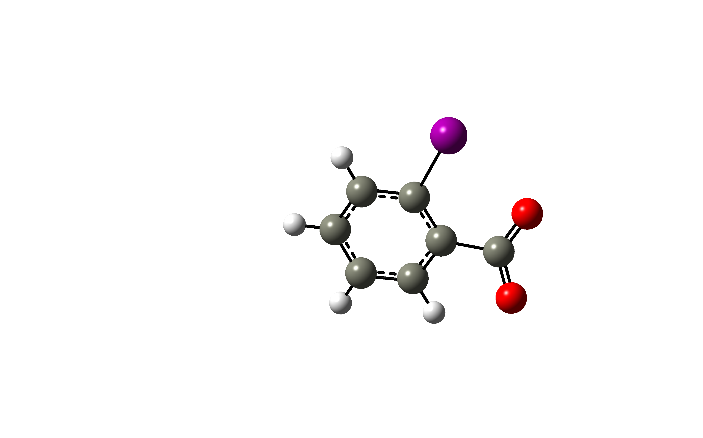


Charge: 1, Multiplicity: 1

# of imaginary frequency = 0

G_sol_: -717.1575949 kcal/mol

C 0.34637700 -0.59554200 -0.01582300

C 0.66067800 -1.96655000 -0.00805800

C 2.00204900 -2.37182500 -0.03763400

C 3.01812000 -1.40347000 -0.05456300

C 2.67763100 -0.04738600 -0.03309100

C 1.33697600 0.40173400 -0.02349000

H -0.13852300 -2.71377000 0.01953400

H 2.24395700 -3.44019500 -0.04202600

H 4.07054400 -1.70738400 -0.07454800

H 3.44273400 0.73544900 -0.01166800

I -1.76357400 -0.15995800 0.03642500

C 1.11810600 1.94297400 -0.02862300

O 0.00805400 2.35956700 -0.45065300

O 2.10333500 2.62094400 0.37388900


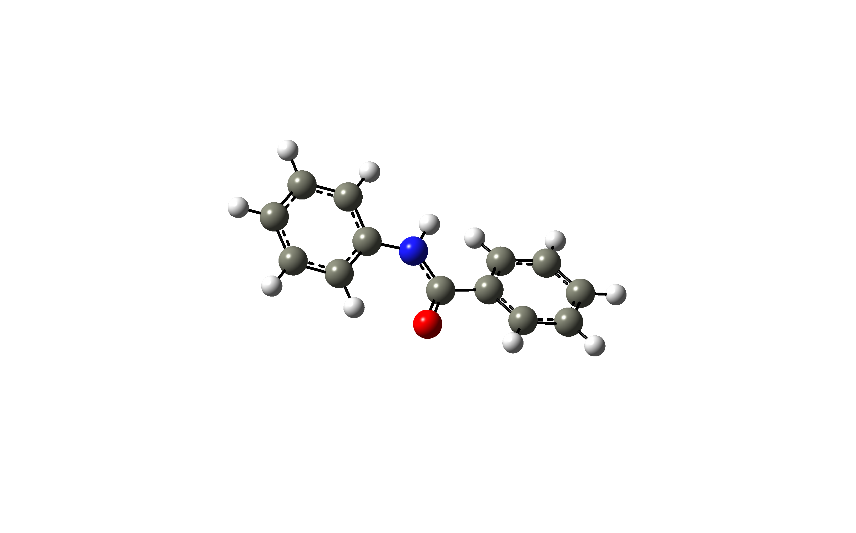


Charge: 0, Multiplicity: 1

# of imaginary frequency = 0

G_sol_: -631.6334359 kcal/mol

C -4.70286100 0.12597200 0.01242600

C -3.95578500 -1.04548100 -0.18103000

C -2.55403500 -1.02448800 -0.16767400

C -1.88037400 0.19871000 0.04295800

C -2.63143200 1.37975600 0.23792900

C -4.02923000 1.34015100 0.22264300

H -5.79697900 0.09480200 0.00037800

H -4.46832000 -1.99957500 -0.34540800

H -1.97117100 -1.93366700 -0.31237100

H -2.10983700 2.32984700 0.40056200

H -4.59392700 2.26581000 0.37570300

N -0.47711100 0.31620400 0.06495900

H -0.13295100 1.25904400 0.24136200

C 0.48163500 -0.66588000 -0.05850400

O 0.21904700 -1.86852300 -0.17178200

C 1.90407000 -0.17563800 -0.02877000

C 2.90107300 -1.12399400 0.27637100

C 2.28437800 1.15053300 -0.32123300

C 4.24877800 -0.75007000 0.31596900

H 2.59220300 -2.15299200 0.48259000

C 3.63525500 1.52218700 -0.28906100

H 1.53845000 1.90019200 -0.60688400

C 4.61897000 0.57535300 0.03506100

H 5.01403900 -1.49253500 0.56476000

H 3.91971800 2.55239700 -0.52584100

H 5.67350700 0.86875800 0.06218200


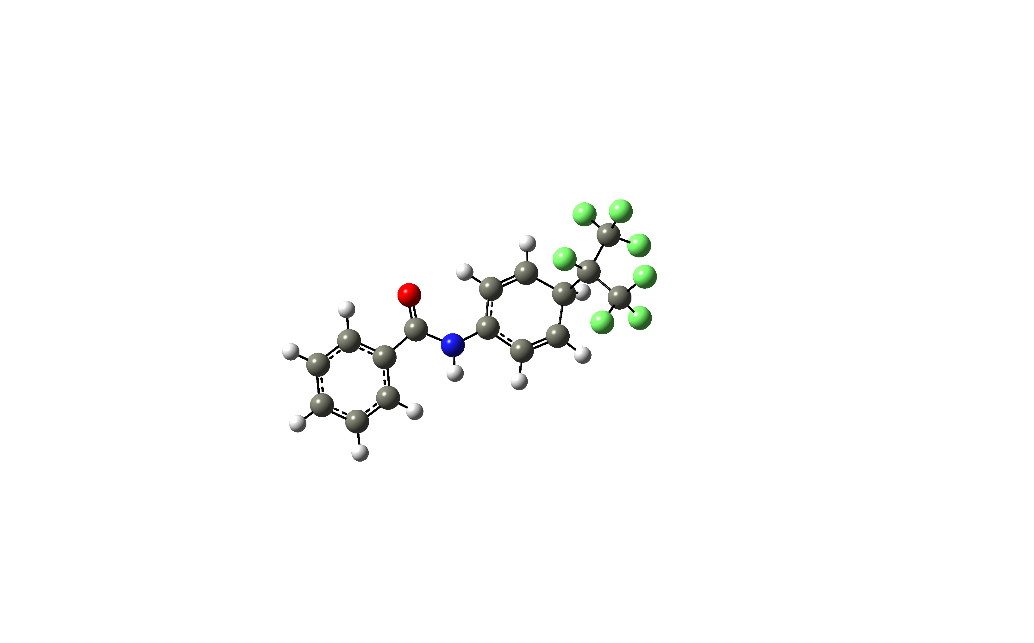


Charge: 0, Multiplicity: 2

# of imaginary frequency = 0

G_sol_: -1444.749909 kcal/mol

C 0.95806700 1.27955400 -0.88081100

C -0.39300100 1.19118500 -0.67270900

C -1.05456900 -0.07595600 -0.62762200

C -0.28681900 -1.25341400 -0.90015800

C 1.06270100 -1.20500000 -1.10882100

H 1.41751900 2.26765200 -0.94289000

H -0.99232300 2.09452600 -0.55561700

H -0.80505700 -2.21658900 -0.97600800

H 1.59658500 -2.12066800 -1.37457100

N -2.41744100 -0.23210700 -0.39675600

H -2.74455000 -1.19801000 -0.41503200

C -3.37161300 0.72222100 -0.08327700

O -3.09989000 1.91901400 0.07687700

C -4.76906200 0.20031100 0.07056700

C -5.69235500 1.03668000 0.73267900

C -5.21019600 -1.04333600 -0.43096300

C -7.01860300 0.63031900 0.91267300

H -5.34125800 2.00479200 1.10151900

C -6.54022900 -1.44635300 -0.25457700

H -4.53455400 -1.70012100 -0.98993700

C -7.44624700 -0.61408900 0.42119500

H -7.72294600 1.28473300 1.43657200

H -6.87119500 -2.41013400 -0.65441700

H -8.48479900 -0.93230900 0.55871900

C 1.86147800 0.07299500 -1.00288500

H 2.50733300 0.16471600 -1.90186700

C 2.89287100 0.06487200 0.20428400

C 3.48398400 -1.34703600 0.49630100

C 4.05456800 1.07783600 -0.01768000

F 2.55565500 -2.12312700 1.08165100

F 4.54236700 -1.28990600 1.33153500

F 3.89338500 -1.94763000 -0.64148100

F 4.92141400 0.58452900 -0.93009600

F 4.72060700 1.31704600 1.12414400

F 3.61580900 2.26705600 -0.47725800

F 2.25194300 0.44640000 1.37001900


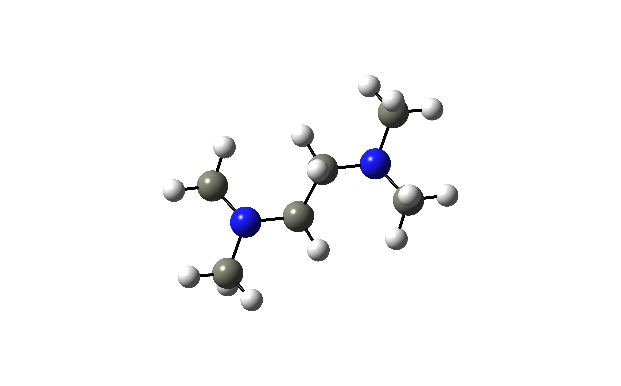


Charge: 0, Multiplicity: 1

# of imaginary frequency = 0

G_sol_: -347.4154339 kcal/mol

C -0.59423783 -0.37227633 -0.31243671

H -0.58486179 -0.19989844 -1.41635507

H -0.46494335 -1.45779423 -0.16053477

C 0.59423783 0.37227633 0.31243671

H 0.58486179 0.19989844 1.41635507

H 0.46494335 1.45779423 0.16053477

N -1.87168842 -0.00092673 0.29990057

N 1.87168842 0.00092673 -0.29990057

C 2.31223354 -1.34103594 0.07079039

H 3.24732166 -1.58310456 -0.46314498

H 2.50415616 -1.44716642 1.16666008

H 1.55633956 -2.09085455 -0.21476615

C 2.91125625 0.98714217 -0.02280633

H 3.16080495 1.07098952 1.06321789

H 3.83635952 0.71275365 -0.55952649

H 2.58695082 1.98183433 -0.37450298

C -2.91125625 -0.98714217 0.02280633

H -3.83635952 -0.71275365 0.55952649

H -3.16080495 -1.07098952 -1.06321789

H -2.58695082 -1.98183433 0.37450298

C -2.31223354 1.34103594 -0.07079039

H -2.50415616 1.44716642 -1.16666008

H -3.24732166 1.58310456 0.46314498

H -1.55633956 2.09085455 0.21476615


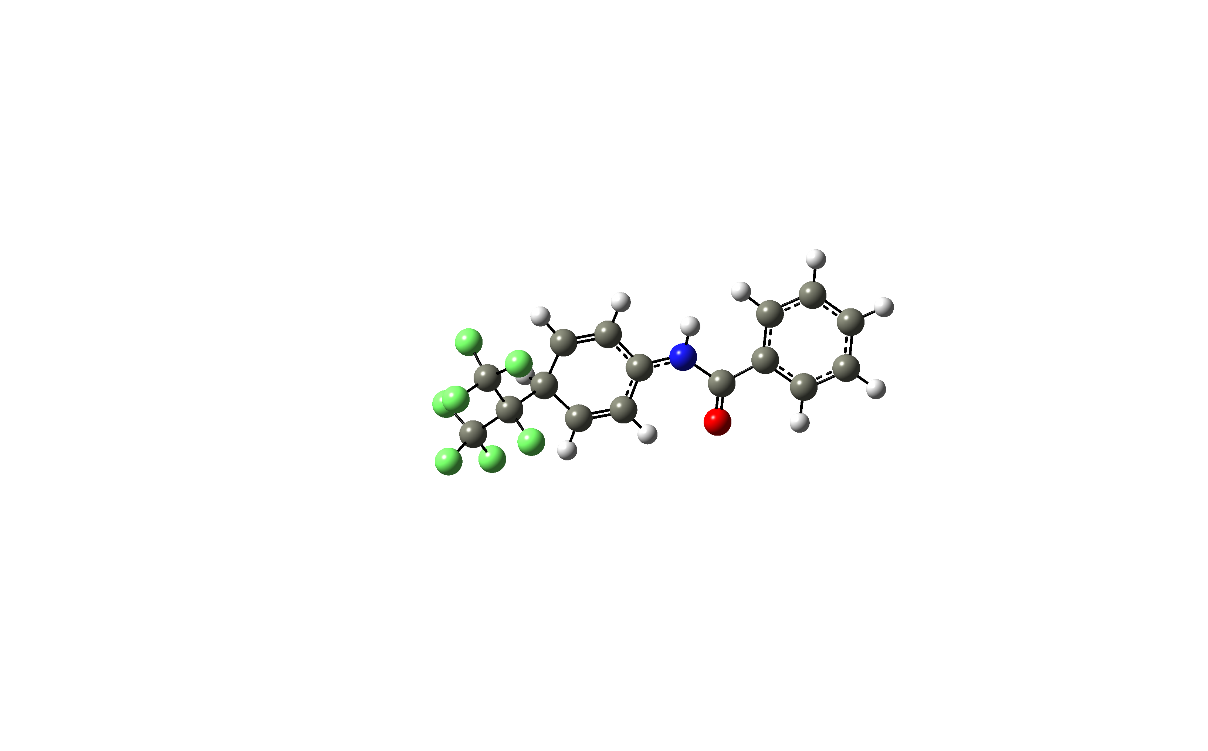


Charge: 1, Multiplicity: 1

# of imaginary frequency = 0

G_sol_: -1444.59575 kcal/mol

C -0.88431800 -1.28107800 -0.39724000

C 0.46506500 -1.19489300 -0.26467100

C 1.12061300 0.08240800 -0.40654100

C 0.34001400 1.25470900 -0.72550100

C -1.00829500 1.18091200 -0.85270000

H -1.36646900 -2.25322400 -0.27615300

H 1.06619300 -2.07380400 -0.03877000

H 0.85354900 2.21019400 -0.86557400

H -1.56844200 2.07763600 -1.12666900

N 2.44678500 0.24973700 -0.26871500

H 2.78342800 1.20849800 -0.38727200

C 3.46048200 -0.71960800 0.05828500

O 3.16272300 -1.87267200 0.32038700

C 4.84738200 -0.19293500 0.06317800

C 5.83989100 -1.05326400 0.58592000

C 5.22006900 1.07793600 -0.43101900

C 7.17411800 -0.64526600 0.62757900

H 5.53734900 -2.03547300 0.95886400

C 6.55946600 1.47925500 -0.39142800

H 4.49141700 1.76425700 -0.87463900

C 7.53572200 0.62241600 0.14020300

H 7.93612400 -1.31319100 1.04015600

H 6.84133300 2.46187100 -0.78050400

H 8.58185200 0.94261900 0.17233800

C -1.75812500 -0.10944900 -0.72592200

H -2.17300900 -0.29463500 -1.74418100

C -3.00311900 -0.09250500 0.23871100

C -3.57644400 1.33641000 0.49255400

C -4.11831600 -1.05450100 -0.27913100

F -2.66591400 2.06793700 1.16089300

F -4.69129500 1.28728000 1.23364000

F -3.86106400 1.95749300 -0.66903800

F -4.67823100 -0.54325300 -1.39210700

F -5.06574900 -1.23949700 0.64563900

F -3.60388500 -2.26221700 -0.58947100

F -2.62251100 -0.55716800 1.48229400


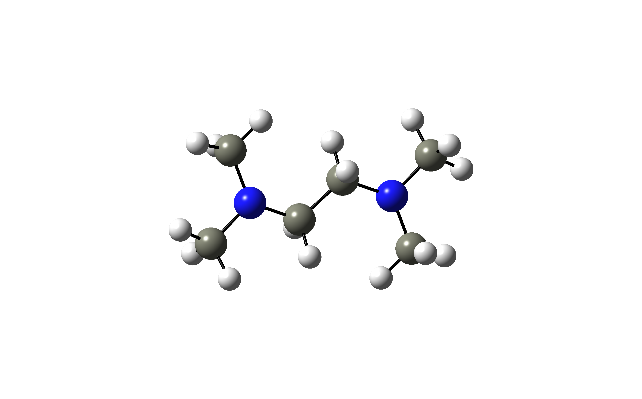


Charge: 1, Multiplicity: 2

# of imaginary frequency = 0

G_sol_: -347.212352 kcal/mol

C -0.54137400 -0.46087400 -0.27179700

H -0.46171600 -0.53113400 -1.38210000

H -0.41254600 -1.48067100 0.12440900

C 0.54137400 0.46087400 0.27179700

H 0.46171600 0.53113400 1.38210000

H 0.41254600 1.48067100 -0.12440900

N -1.87616100 -0.02621400 0.12527800

N 1.87616100 0.02621400 -0.12527800

C 2.27197300 -1.36452000 0.01979300

H 3.22332300 -1.52190800 -0.50996600

H 2.41878300 -1.62250800 1.09087100

H 1.51049400 -2.03887400 -0.39806300

C 2.92923000 1.01390900 0.02382500

H 3.21806800 1.12896700 1.09132300

H 3.81789200 0.69183800 -0.54095300

H 2.57993700 1.98899300 -0.34940000

C -2.92923000 -1.01390900 -0.02382500

H -3.81789200 -0.69183800 0.54095300

H -3.21806800 -1.12896700 -1.09132300

H -2.57993700 -1.98899300 0.34940000

C -2.27197300 1.36452000 -0.01979300

H -2.41878300 1.62250800 -1.09087100

H -3.22332300 1.52190800 0.50996600

H -1.51049400 2.03887400 0.39806300


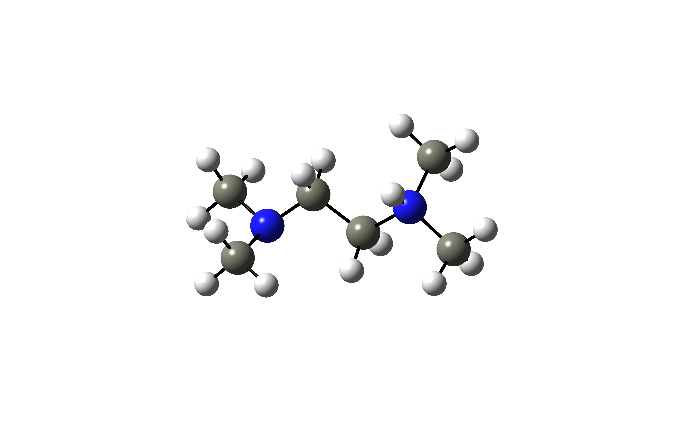


Charge: 1, Multiplicity: 1

# of imaginary frequency = 0

G_sol_: -347.8721546 kcal/mol

C 0.63250000 -0.43096100 0.26436400

H 0.56763600 -0.32680600 1.37433600

H 0.56028400 -1.50589500 0.03483900

C -0.52497800 0.31991600 -0.40357100

H -0.63564200 0.02606200 -1.45770500

H -0.36393100 1.40639700 -0.35452300

N 1.89301500 0.06632000 -0.27696300

C -2.29858200 -1.36765700 0.18645600

H -3.26149900 -1.46592900 0.70458700

H -2.40164800 -1.63227700 -0.87474900

H -1.54367100 -1.99915700 0.67081600

C -2.91972700 0.99760700 -0.26803800

H -3.04876700 0.78663700 -1.33792000

H -3.85454100 0.81188200 0.27678500

H -2.58365900 2.03184200 -0.11805800

C 2.94761500 -0.94617400 -0.27037000

H 3.84138800 -0.54200700 -0.77524000

H 3.24728000 -1.26107800 0.75653800

H 2.61072500 -1.83950700 -0.82265700

C 2.33366500 1.31049500 0.35210000

H 2.59188300 1.17993400 1.42917400

H 3.22537700 1.69090600 -0.17347000

H 1.54783400 2.08106900 0.28143600

H -1.74129100 0.29704000 1.27073100

N -1.86168900 0.07104100 0.26974000


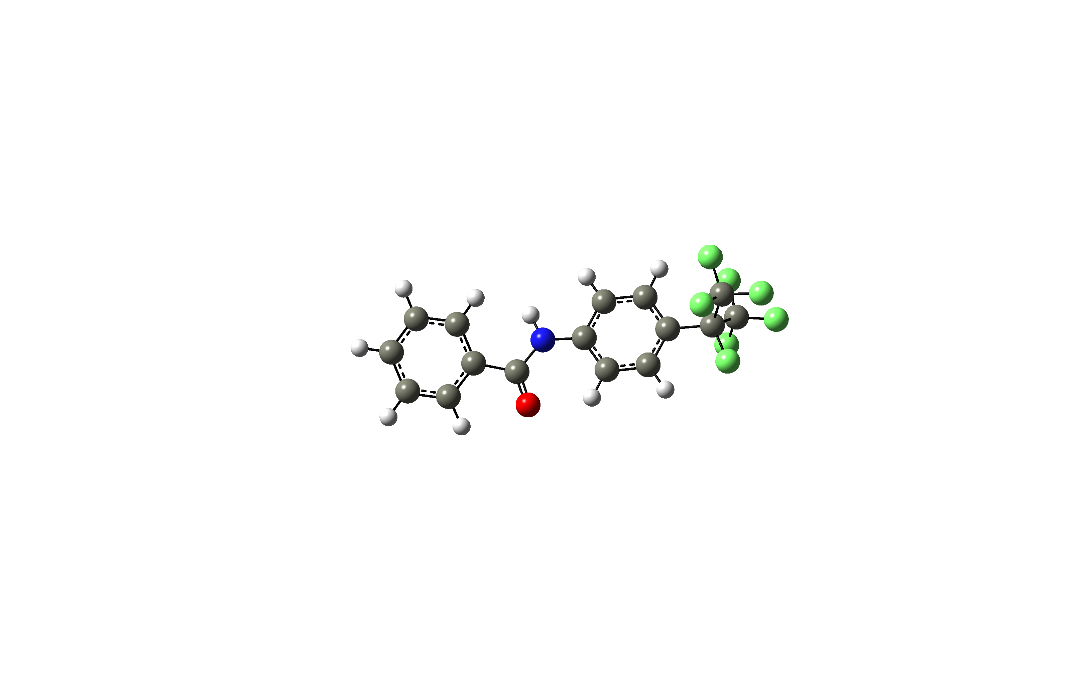


Charge: 0, Multiplicity: 1

# of imaginary frequency = 0

G_sol_: -1444.222553 kcal/mol

C -1.69131300 -0.17199200 -0.06765800

C -0.87701800 -1.12934400 -0.69433900

C 0.51727800 -1.04597500 -0.63175600

C 1.12478800 0.01843500 0.06893000

C 0.30463800 0.98258900 0.69722300

C -1.08507200 0.89077700 0.63081400

H -1.33763800 -1.95636300 -1.23938100

H 1.14431900 -1.79464600 -1.11415900

H 0.76440500 1.81393400 1.24173000

H -1.69006800 1.65280800 1.12622300

N 2.51353800 0.18264600 0.17473900

H 2.80898400 0.98475200 0.73000700

C 3.52355100 -0.62036700 -0.32115300

O 3.31688100 -1.68258600 -0.91510300

C 4.91731600 -0.11627500 -0.07252300

C 5.96303900 -1.05762500 -0.15716800

C 5.22574800 1.23308500 0.19749900

C 7.28929800 -0.66457900 0.05190800

H 5.70937300 -2.09689200 -0.38584100

C 6.55547200 1.62671200 0.39868300

H 4.44012300 1.99605300 0.22298900

C 7.58817800 0.67886900 0.33241800

H 8.09342600 -1.40535400 -0.00497300

H 6.78511200 2.67794400 0.59946900

H 8.62606500 0.98842100 0.49292900

C -3.20015400 -0.30356000 -0.17398900

C -3.88068200 -0.45617000 1.21734400

C -3.82046200 0.84123700 -1.02626400

F -3.53711000 -1.46775100 -0.85548800

F -3.29007300 -1.45542900 1.89776800

F -5.18653600 -0.75407700 1.09540700

F -3.77588300 0.67410300 1.94764100

F -5.14304500 0.66752300 -1.20418900

F -3.62991800 2.04558800 -0.45001200

F -3.23760700 0.85522200 -2.23903200


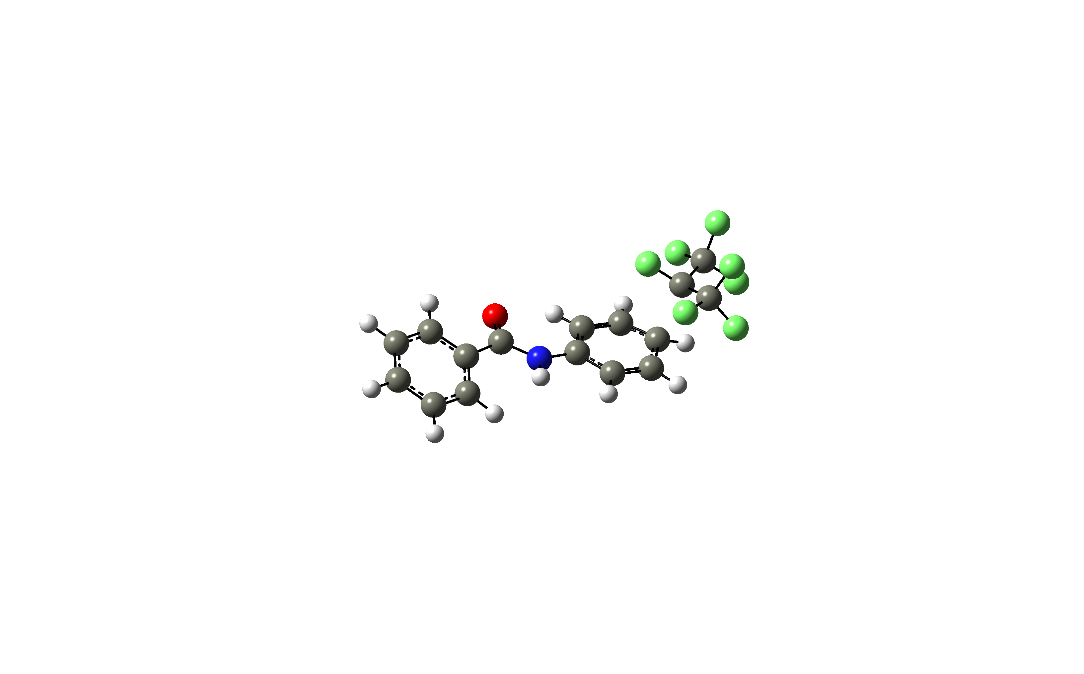


Charge: 0, Multiplicity: 2

# of imaginary frequency = 1

G_sol_: -1444.729379 kcal/mol

C -1.01668800 -1.30997600 -1.34595300

C 0.32233500 -1.23212600 -1.00071000

C 0.96127600 0.03609300 -0.92027700

C 0.22538700 1.20516300 -1.26028400

C -1.11099800 1.12406400 -1.60356400

H -1.48790800 -2.29220800 -1.43194300

H 0.90758900 -2.13042600 -0.80660500

H 0.73639800 2.17415300 -1.27359900

H -1.65320900 2.02922800 -1.88982600

N 2.29986200 0.20191800 -0.56659100

H 2.62248400 1.16927500 -0.55783300

C 3.22583900 -0.74764800 -0.16184400

O 2.94809900 -1.94124500 -0.01483900

C 4.60343400 -0.21641100 0.10803300

C 5.45543700 -1.02106800 0.89221500

C 5.08974300 1.00525900 -0.40359300

C 6.75795000 -0.60204300 1.18255900

H 5.06975300 -1.97340800 1.26756500

C 6.39726600 1.41968300 -0.11787100

H 4.47034800 1.63200200 -1.05461800

C 7.23139400 0.62097800 0.67951300

H 7.40769100 -1.22935000 1.80133600

H 6.76733500 2.36528300 -0.52648600

H 8.25180500 0.94867400 0.90337400

C -1.81746300 -0.12923700 -1.53073600

H -2.76219700 -0.21493200 -2.07842200

C -2.86081100 -0.04391900 0.34220800

C -3.27663100 1.39283900 0.59794800

C -3.98596000 -1.05313400 0.24559100

F -2.20297400 2.14535400 0.90523300

F -4.15484500 1.51690000 1.63267700

F -3.88022400 1.92483100 -0.48965500

F -4.87354000 -0.67312700 -0.70195000

F -4.67038300 -1.19695300 1.41105200

F -3.52116000 -2.27969400 -0.07711500

F -1.94387000 -0.44318200 1.26559000


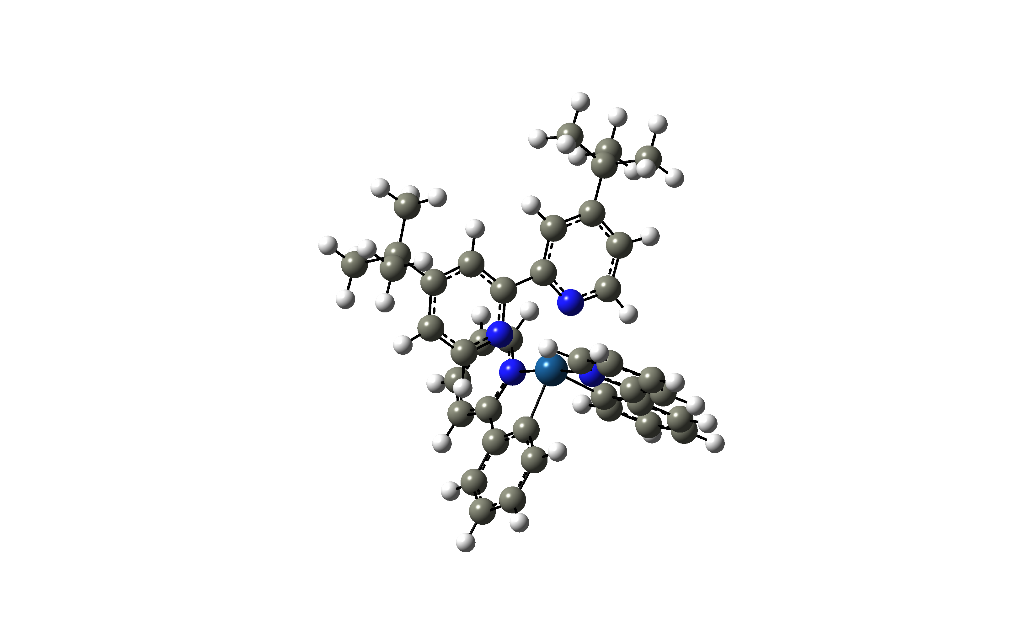


Charge: 1, Multiplicity: 1

# of imaginary frequency = 0

G_sol_: -1870.390319 kcal/mol

Ir -1.00544500 0.00000100 0.00000000

C -2.44180600 1.39143900 0.27313600

N 0.70214800 1.28912800 0.33162200

N 0.70213900 -1.28914600 -0.33158800

N -1.10522800 0.76954500 -1.91140700

C -2.44182100 -1.39141500 -0.27317200

N -1.10529400 -0.76953800 1.91140600

C -3.13458100 1.69727600 1.46300300

C -2.74187400 2.17001300 -0.88708400

C 1.92972500 0.72079100 0.16671300

C 0.62579500 2.60426200 0.62848600

C 0.62577700 -2.60428100 -0.62844600

C 1.92972000 -0.72081600 -0.16668100

C -1.99515500 1.80372000 -2.09180500

C -0.35689400 0.32672700 -2.94812300

C -3.13456300 -1.69724700 -1.46305900

C -2.74194100 -2.16997600 0.88704400

C -1.99525100 -1.80369100 2.09178500

C -0.35697600 -0.32673200 2.94814000

C -4.07730300 2.73611600 1.50556500

H -2.93725000 1.11949500 2.37250100

C -3.69085900 3.21407400 -0.84019400

C 3.10224600 1.47916300 0.31052300

C 1.75656500 3.40152000 0.78316000

H -0.38458800 3.00703300 0.74402200

C 1.75654100 -3.40154700 -0.78311900

H -0.38461000 -3.00704500 -0.74397700

C 3.10223600 -1.47919700 -0.31049200

C -2.11918300 2.40019100 -3.36037400

C -0.44886400 0.88435400 -4.22160100

H 0.32327700 -0.49934200 -2.72929800

C -4.07730300 -2.73607000 -1.50564500

H -2.93719100 -1.11947600 -2.37255500

C -3.69094400 -3.21401900 0.84013000

C -2.11933100 -2.40014900 3.36035500

C -0.44899700 -0.88434800 4.22161900

H 0.32322300 0.49931700 2.72932700

C -4.35782400 3.49744900 0.35550300

H -4.60143200 2.95530600 2.44293600

H -3.90982900 3.80693300 -1.73455800

C 3.04444800 2.84550500 0.62618600

H 4.06650800 0.99180400 0.16815300

H 1.62007700 4.45845200 1.02621000

C 3.04442700 -2.84553900 -0.62615100

H 1.62004600 -4.45847900 -1.02616500

H 4.06650100 -0.99184500 -0.16812400

C -1.34763000 1.94282200 -4.42961200

H -2.82588100 3.22147500 -3.49903500

H 0.17418800 0.49200200 -5.02890300

C -4.35787500 -3.49738900 -0.35558600

H -4.60140500 -2.95525700 -2.44303100

H -3.90995400 -3.80686800 1.73449100

C -1.34779800 -1.94279100 4.42961200

H -2.82605400 -3.22141300 3.49900200

H 0.17404200 -0.49200700 5.02893600

H -5.09391700 4.30674400 0.39446300

C 4.29652600 3.71207000 0.79754300

C 4.29650000 -3.71211300 -0.79751100

H -1.44520100 2.40481500 -5.41642800

H -5.09398300 -4.30667100 -0.39456500

H -1.44541000 -2.40477400 5.41642900

C 5.59108000 2.90744900 0.57354800

C 4.30578100 4.28758800 2.23658300

C 4.24312200 4.87254500 -0.22806700

C 5.59106000 -2.90749900 -0.57351900

C 4.30574700 -4.28763000 -2.23655200

C 4.24309200 -4.87258700 0.22809800

H 6.46139000 3.57195200 0.70558000

H 5.63928000 2.48874700 -0.44670200

H 5.68736600 2.07964900 1.29748500

H 5.19773400 4.92275000 2.37635700

H 4.33665800 3.47529100 2.98318700

H 3.41342400 4.90554000 2.43367200

H 5.13479300 5.51252700 -0.11037600

H 3.35010000 5.50375400 -0.08320500

H 4.22841100 4.48271900 -1.26050200

H 6.46136500 -3.57200800 -0.70555300

H 5.63926400 -2.48879700 0.44673000

H 5.68734900 -2.07970000 -1.29745700

H 5.19769500 -4.92279700 -2.37632800

H 4.33662700 -3.47533300 -2.98315500

H 3.41338600 -4.90557700 -2.43363700

H 5.13475900 -5.51257500 0.11040500

H 3.35006500 -5.50379100 0.08324000

H 4.22838700 -4.48276100 1.26053400


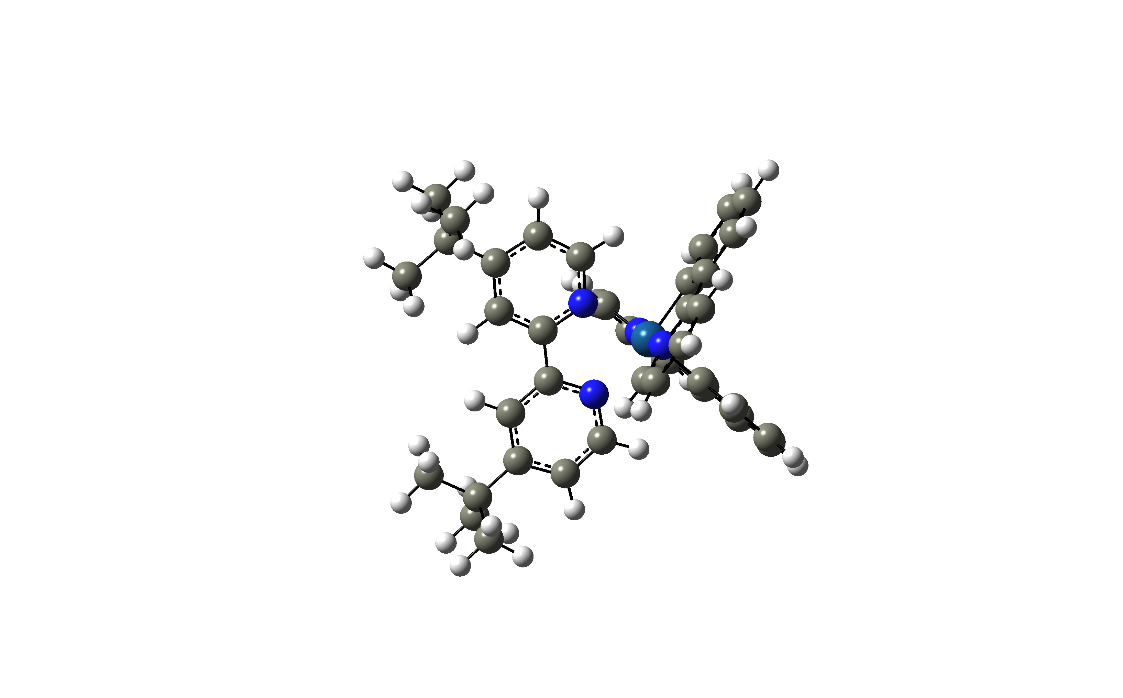


Charge: 2, Multiplicity: 2

# of imaginary frequency = 0

G_sol_: -1870.190529 kcal/mol

Ir -1.06937700 -0.00000400 -0.00000100

C -2.40626800 -1.43722200 -0.32182100

N 0.66852200 -1.28902500 -0.33200100

N 0.66850900 1.28903900 0.33196900

N -1.12732800 -0.80878800 1.90473900

C -2.40628500 1.43719100 0.32184800

N -1.12738500 0.80877800 -1.90473900

C -3.05712900 -1.73938300 -1.54261800

C -2.67109200 -2.26464500 0.82550500

C 1.89412300 -0.71946900 -0.16722100

C 0.59344200 -2.60589800 -0.62912200

C 0.59341400 2.60591300 0.62908400

C 1.89411600 0.71949400 0.16719400

C -1.95138600 -1.89880800 2.04678200

C -0.39474400 -0.36288100 2.94775000

C -3.05712000 1.73934200 1.54266000

C -2.67115000 2.26460800 -0.82547300

C -1.95146500 1.89878300 -2.04676600

C -0.39481500 0.36288300 -2.94776500

C -3.95189600 -2.80888300 -1.62118900

H -2.86456600 -1.13046000 -2.43031600

C -3.57438900 -3.33595300 0.73442600

C 3.06661000 -1.47512800 -0.31089300

C 1.72570200 -3.39944500 -0.78338100

H -0.41111500 -3.01764700 -0.75159000

C 1.72566600 3.39947100 0.78334500

H -0.41114700 3.01765200 0.75154500

C 3.06659500 1.47516600 0.31087000

C -2.03771300 -2.55281000 3.28667900

C -0.44871300 -0.97897600 4.19850800

H 0.23626500 0.50851700 2.76075800

C -3.95190500 2.80882600 1.62125200

H -2.86452500 1.13042300 2.43035400

C -3.57446500 3.33590000 -0.73437400

C -2.03783000 2.55278300 -3.28666100

C -0.44882200 0.97897600 -4.19852200

H 0.23621400 -0.50850300 -2.76078600

C -4.21023900 -3.60610500 -0.48556000

H -4.45723000 -3.03110200 -2.56624300

H -3.78821400 -3.96416600 1.60370000

C 3.01215300 -2.84189600 -0.62636800

H 4.02935300 -0.98577200 -0.16825300

H 1.58831000 -4.45548500 -1.02831200

C 3.01212300 2.84193400 0.62634000

H 1.58826300 4.45551100 1.02827000

H 4.02934300 0.98581800 0.16823400

C -1.28440800 -2.09170300 4.36918500

H -2.69389200 -3.41855400 3.39597300

H 0.15736200 -0.58514100 5.01746700

C -4.21029000 3.60604200 0.48562800

H -4.45721900 3.03103800 2.56631800

H -3.78832200 3.96410800 -1.60364300

C -1.28454000 2.09168800 -4.36918300

H -2.69402800 3.41851500 -3.39594100

H 0.15724300 0.58515200 -5.01749500

H -4.90956300 -4.44448800 -0.55615500

C 4.26561900 -3.70359000 -0.79857400

C 4.26558100 3.70363900 0.79855200

H -1.34837100 -2.59594900 5.33730500

H -4.90962800 4.44441200 0.55623900

H -1.34853300 2.59593200 -5.33730200

C 5.55741600 -2.89562300 -0.57136800

C 4.27465700 -4.27432800 -2.24003800

C 4.21206400 -4.86700000 0.22420500

C 5.55738600 2.89568600 0.57134900

C 4.27460700 4.27437400 2.24001800

C 4.21201800 4.86705100 -0.22422400

H 6.42894800 -3.55819100 -0.70308200

H 5.60326600 -2.47888600 0.44972600

H 5.65332100 -2.06707100 -1.29441500

H 5.16887900 -4.90574400 -2.38029100

H 4.30333100 -3.45983500 -2.98410200

H 3.38471800 -4.89516600 -2.43848600

H 5.10544000 -5.50371500 0.10418400

H 3.32103800 -5.50012700 0.07621300

H 4.19625200 -4.48036200 1.25764600

H 6.42891100 3.55826100 0.70306700

H 5.60324300 2.47895100 -0.44974600

H 5.65329600 2.06713200 1.29439400

H 5.16882300 4.90579700 2.38027600

H 4.30328600 3.45987800 2.98408000

H 3.38466200 4.89520200 2.43846400

H 5.10538800 5.50377400 -0.10419800

H 3.32098600 5.50017000 -0.07623400

H 4.19621400 4.48041600 -1.25766600


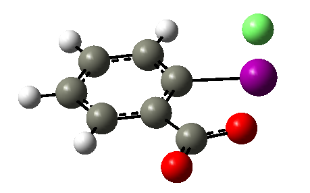


Charge: 0, Multiplicity: 1

# of imaginary frequency = 0

G_sol_: -816.821851 kcal/mol

C 0.56896500 -0.58497700 -0.00000100

C 0.92514900 -1.92943600 -0.00000100

C 2.30257800 -2.21083600 0.00000000

C 3.25275900 -1.17316100 0.00000100

C 2.84607100 0.16679500 0.00000100

C 1.47422900 0.47709000 0.00000000

H 0.16295600 -2.71193400 -0.00000200

H 2.63206100 -3.25460900 0.00000000

H 4.31919300 -1.41726200 0.00000100

H 3.56710000 0.99009200 0.00000200

I -1.44365900 0.08604200 0.00000000

C 0.95293200 1.88275000 -0.00000100

O -0.38183800 1.96796300 0.00000200

O 1.66903300 2.87555500 -0.00000300

F -2.04456100 -1.85378200 -0.00000100


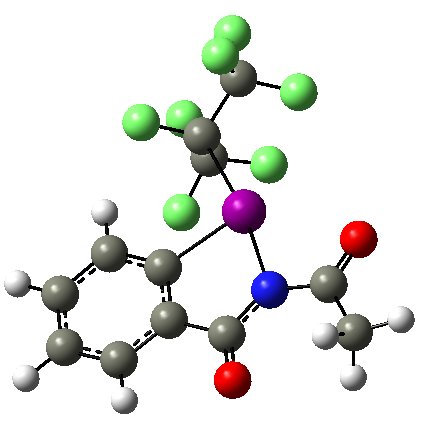


Charge: 0, Multiplicity: 1

# of imaginary frequency = 0

G_sol_: -1662.812180 kcal/mol

C 1.11665100 1.35584400 -0.21119800

C 0.40584700 2.54314800 -0.38375000

C 1.13725100 3.74121600 -0.46118400

C 2.53715400 3.74002300 -0.37031900

C 3.22208700 2.53305000 -0.20389000

C 2.51921900 1.31693600 -0.12302000

H -0.67876200 2.56464800 -0.46406300

H 0.59236000 4.68062000 -0.59627500

H 3.09098200 4.68137900 -0.43168100

H 4.31331200 2.48917800 -0.13183400

I 0.17913700 -0.58738300 -0.05658500

C 3.27428700 0.03150700 0.04645600

O 4.50434500 -0.01475000 0.12344800

C -2.08766700 0.24433800 -0.24061100

F -2.25493600 1.28930400 -1.12539500

C -2.57630300 0.70191600 1.13452700

C -2.85568000 -0.95570300 -0.80734900

F -1.75996000 1.65542600 1.63846400

F -3.81849200 1.22671100 1.09095500

F -2.58460900 -0.33847500 1.99387900

F -4.19601100 -0.82682100 -0.71698400

F -2.50489900 -2.08456000 -0.14157000

F -2.54625300 -1.12025200 -2.10562400

N 2.40681900 -1.03159700 0.09580600

C 2.65313100 -2.38427700 0.24284000

C 4.07893200 -2.87133300 0.36729000

H 4.66456700 -2.56696400 -0.51563500

H 4.56466100 -2.40713300 1.24132900

H 4.06587300 -3.96624800 0.46684300

O 1.68090300 -3.14962500 0.26622300


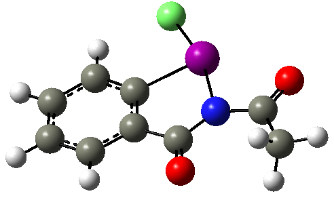


Charge: 0, Multiplicity: 1

# of imaginary frequency = 0

G_sol_: -949.519502 kcal/mol

C 1.34208400 0.00749900 -0.00000300

C 2.64585700 -0.47984400 0.00000400

C 3.68491300 0.46671700 0.00001000

C 3.40509100 1.84499600 0.00000900

C 2.08144500 2.29995400 0.00000200

C 1.02265900 1.37357200 -0.00000500

H 2.82758600 -1.55734400 0.00000600

H 4.72276800 0.11884900 0.00001500

H 4.22817700 2.56574200 0.00001500

H 1.83761700 3.36680500 0.00000100

I -0.33649400 -1.29846800 -0.00000600

C -0.40750500 1.80509200 -0.00001100

O -0.77262400 2.97988900 -0.00000300

N -1.24751700 0.69862100 -0.00003000

C -2.63112500 0.62006900 0.00000700

C -3.45001600 1.88650400 0.00001500

H -3.20512100 2.49798800 0.88383100

H -3.20519300 2.49794300 -0.88385400

H -4.51429800 1.61170100 0.00005900

O -3.13128400 -0.51159000 0.00002500

F 0.99401200 -2.87410700 0.00001600


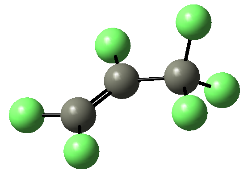


Charge: 0, Multiplicity: 1

# of imaginary frequency = 0

G_sol_: -713.294865 kcal/mol

C -1.42530500 -0.16088600 -0.00000100

C -0.24199700 0.47870900 -0.00000500

C 1.12686800 -0.13627900 0.00000000

F 1.07499700 -1.48105300 -0.00000300

F 1.83242500 0.25976200 1.08628300

F -1.57977500 -1.46706300 -0.00000300

F -2.57479000 0.48155500 0.00000400

F -0.22500500 1.82600500 -0.00000500

F 1.83243800 0.25976500 -1.08627100
