# Supplementary material for: Direct electrophilic and radical isoperfluoropropylation with i-C3F7-Iodine(III) reagent (PFPI reagent)
Source: Commun Chem. 2023 Aug 24;6:177. doi: 10.1038/s42004-023-00986-3 (PMC10449889; doi:10.1038/s42004-023-00986-3)
Supplement: Supplementary file 6 — Supplementary Data 4 [file 42004_2023_986_MOESM6_ESM.docx]

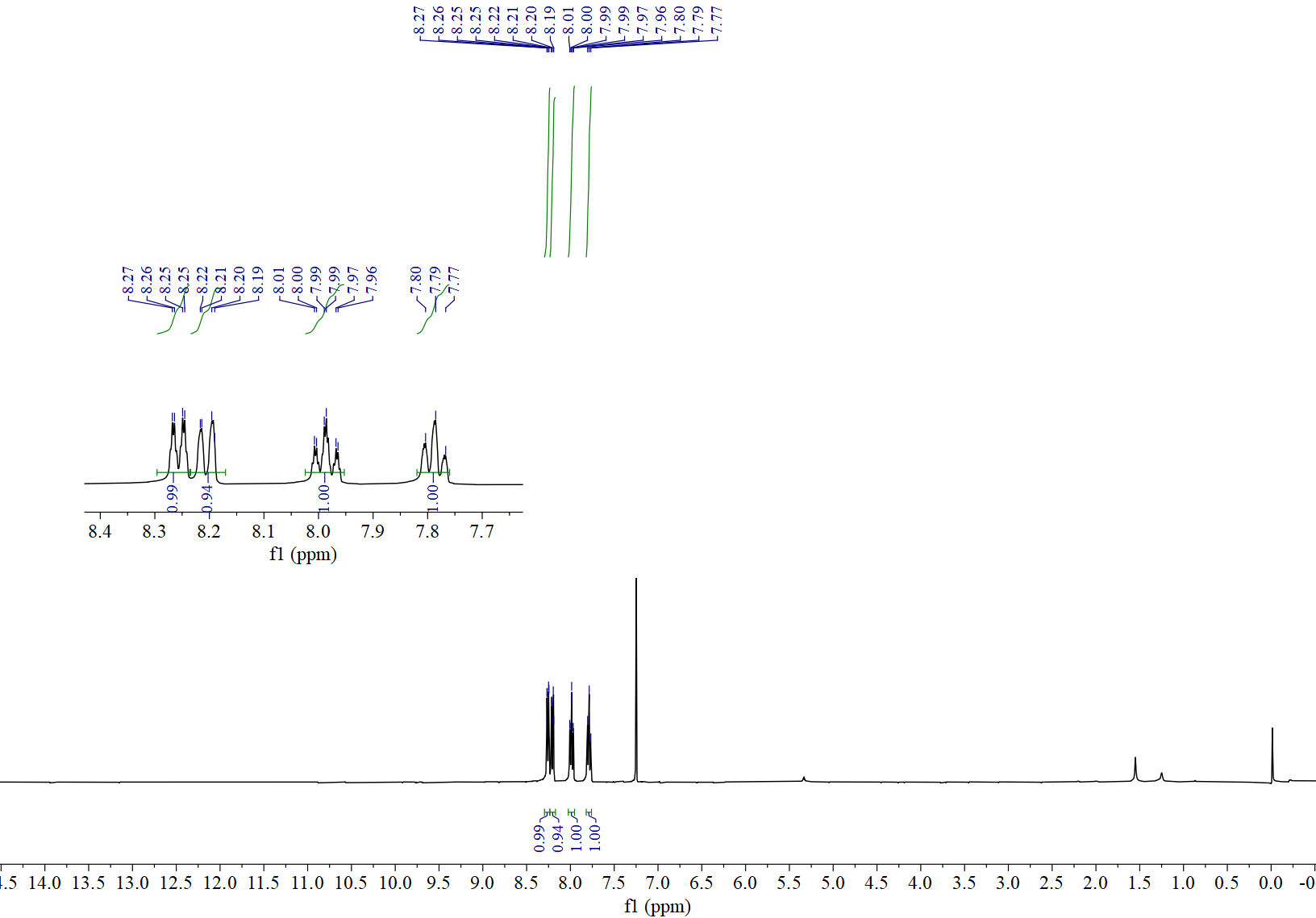
**NMR Spectra**

**^1^H NMR** of **Chloroiodine(III)** (400 MHz, Chloroform-*d*, 298 K)


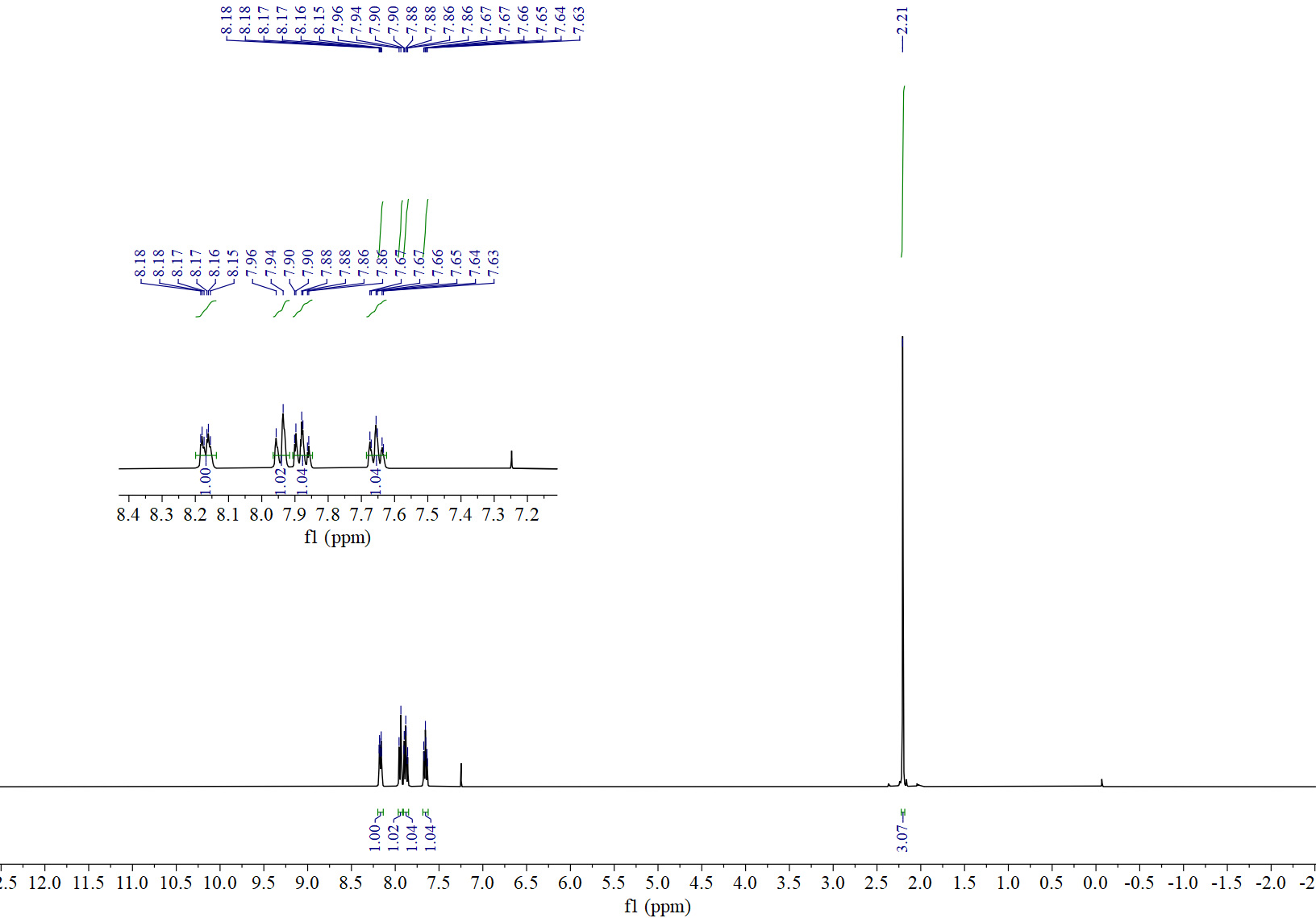

**^1^H NMR** of **Acetoxyiodine(III)** (400 MHz, Chloroform-*d*, 298 K)


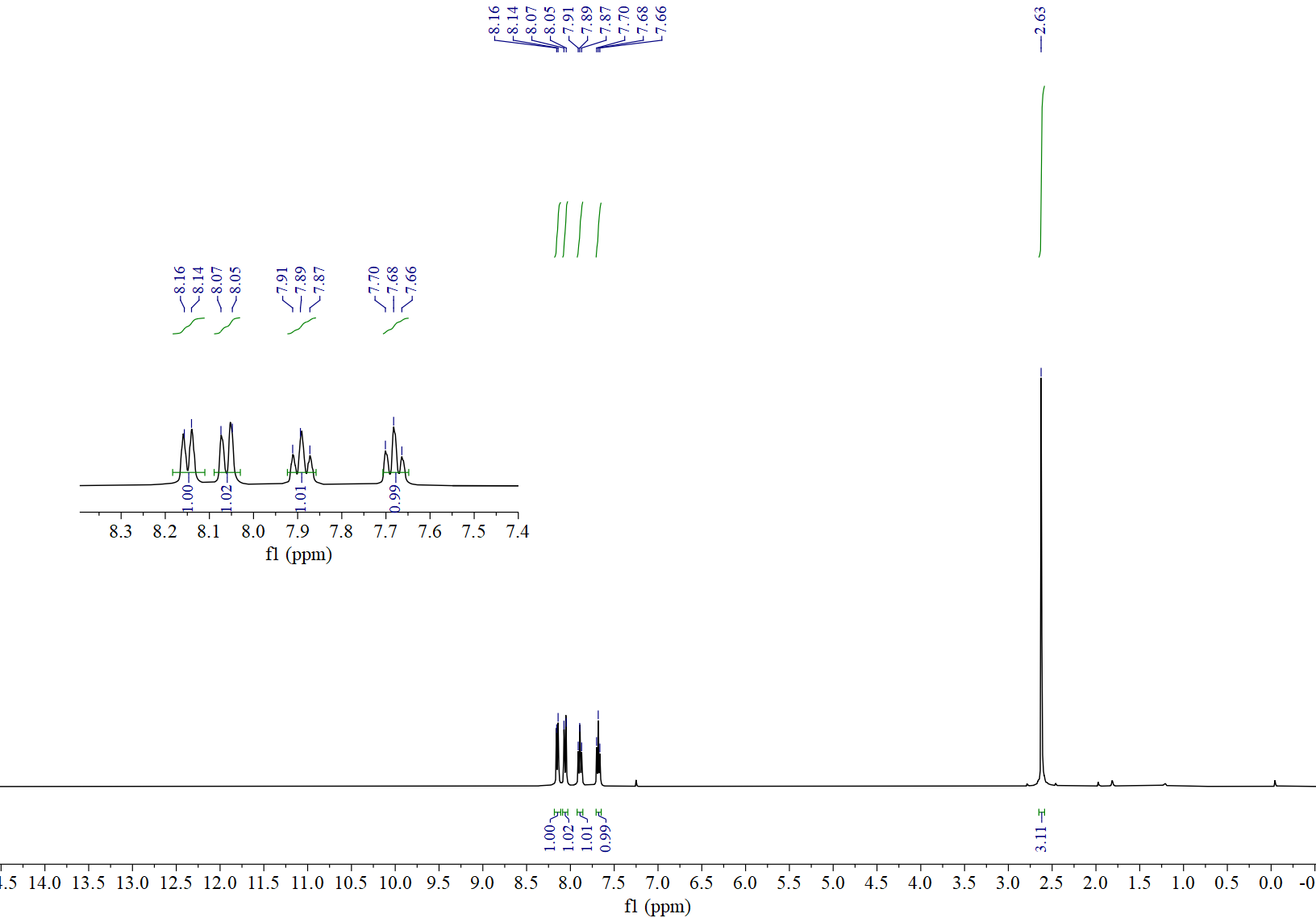

**^1^H NMR** of **Fluoroiodine(III)** (400 MHz, Chloroform-*d*, 298 K)


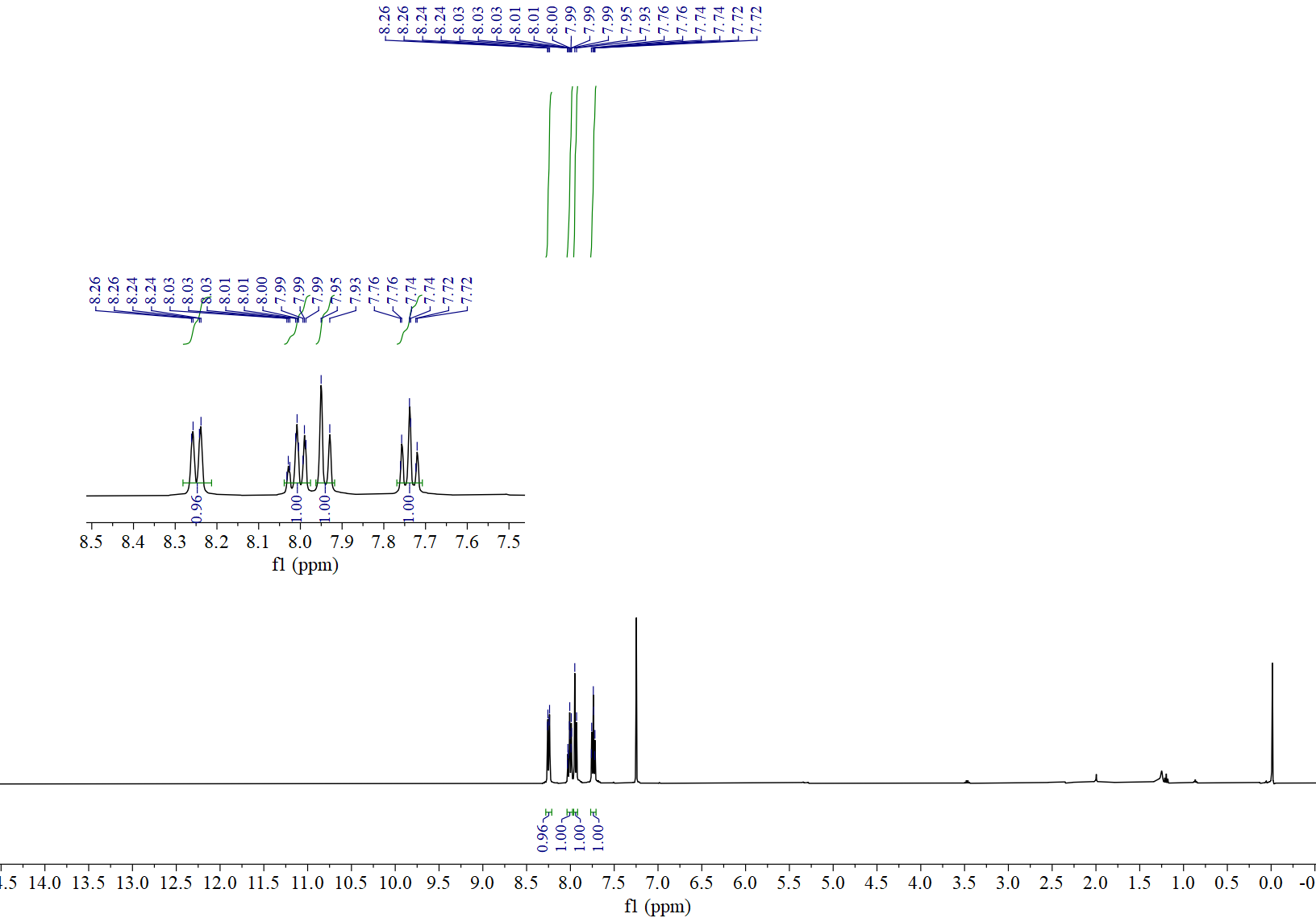

**^1^H NMR** of **2** (400 MHz, Chloroform-*d*, 298 K)


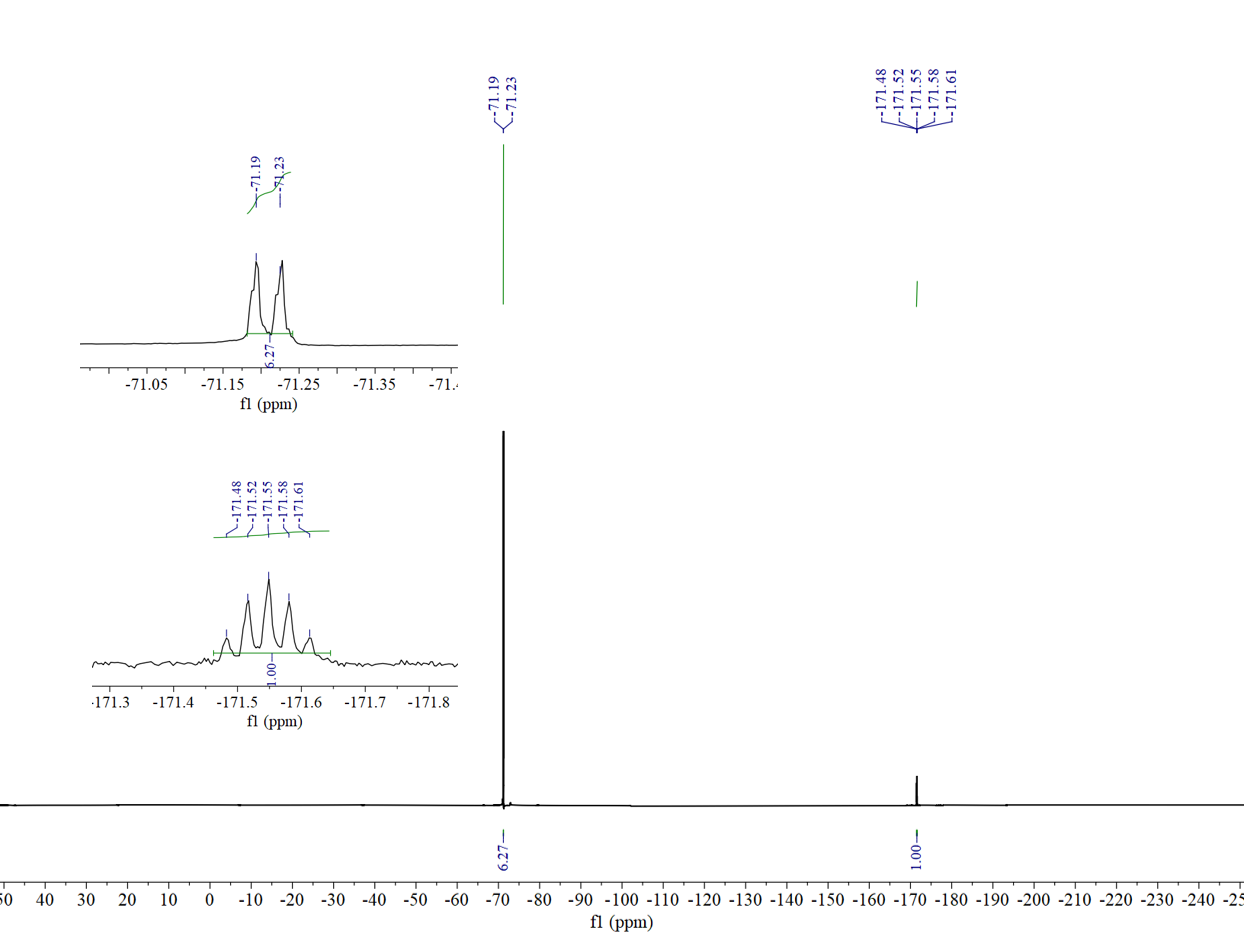

**^19^F NMR** of **2** (376 MHz, Chloroform-*d*, 298 K)


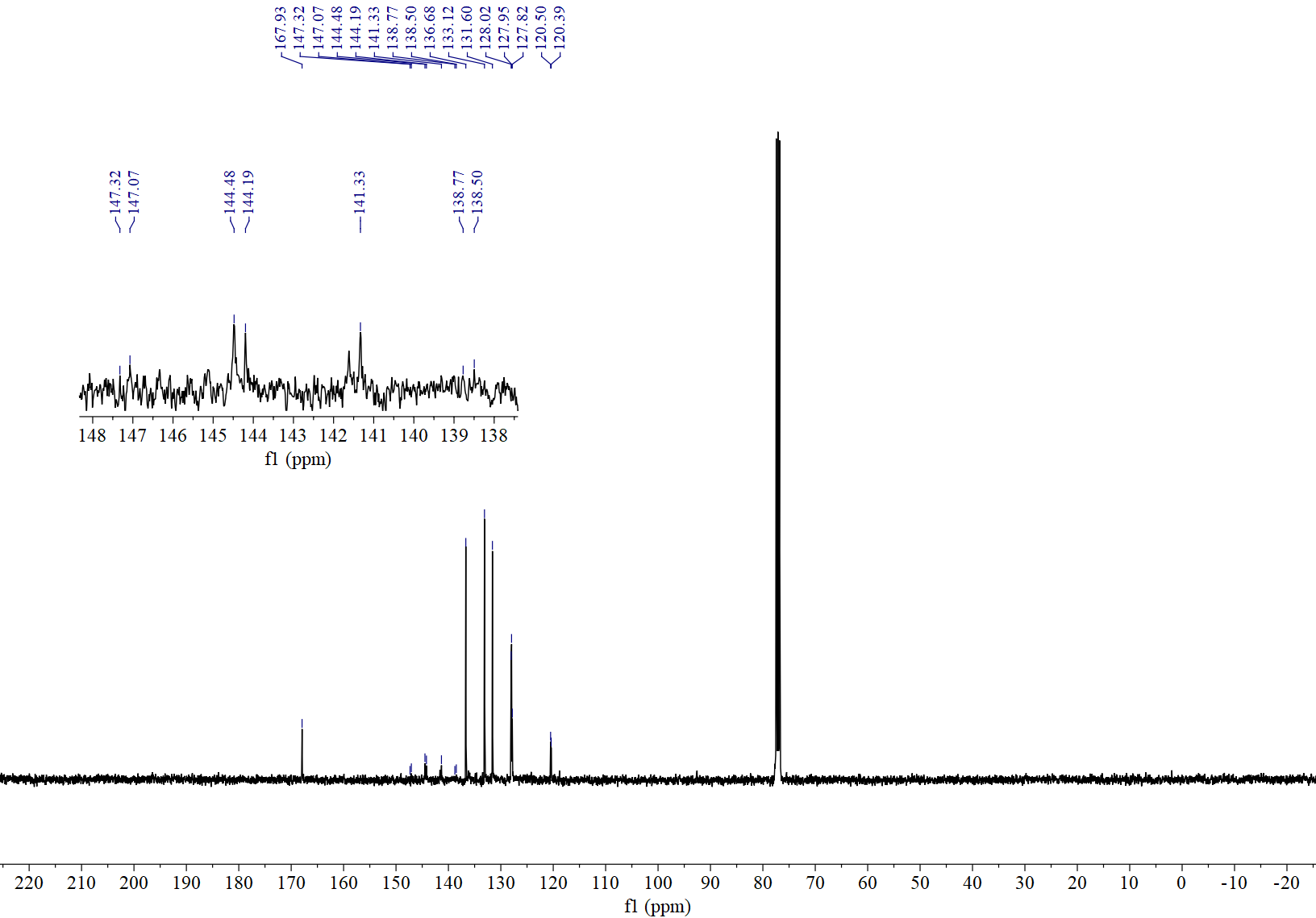

**^13^C NMR** of **2** (101 MHz, Chloroform-*d*, 298 K)


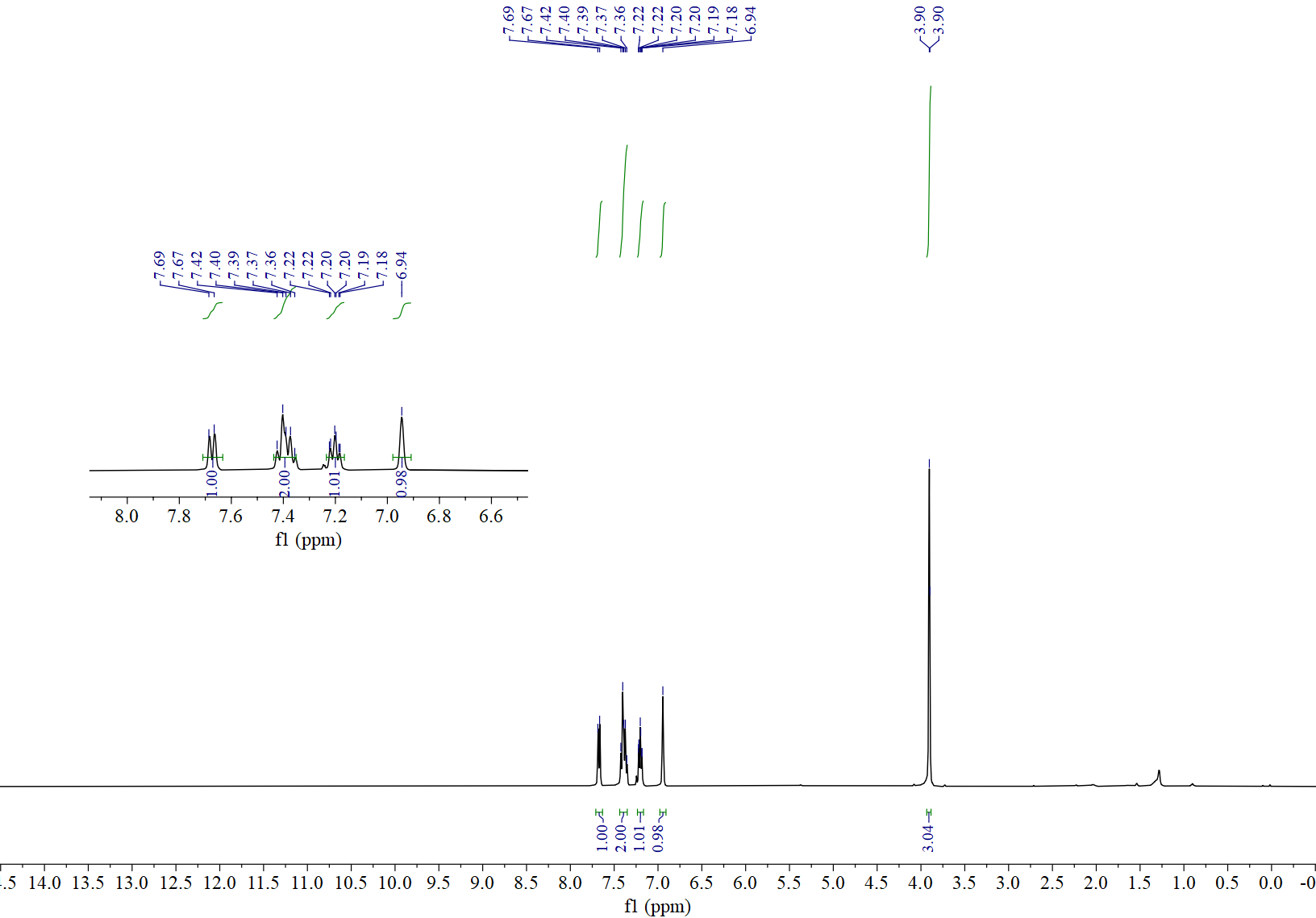

**^1^H NMR** of **4** (400 MHz, Chloroform-*d*, 298 K)


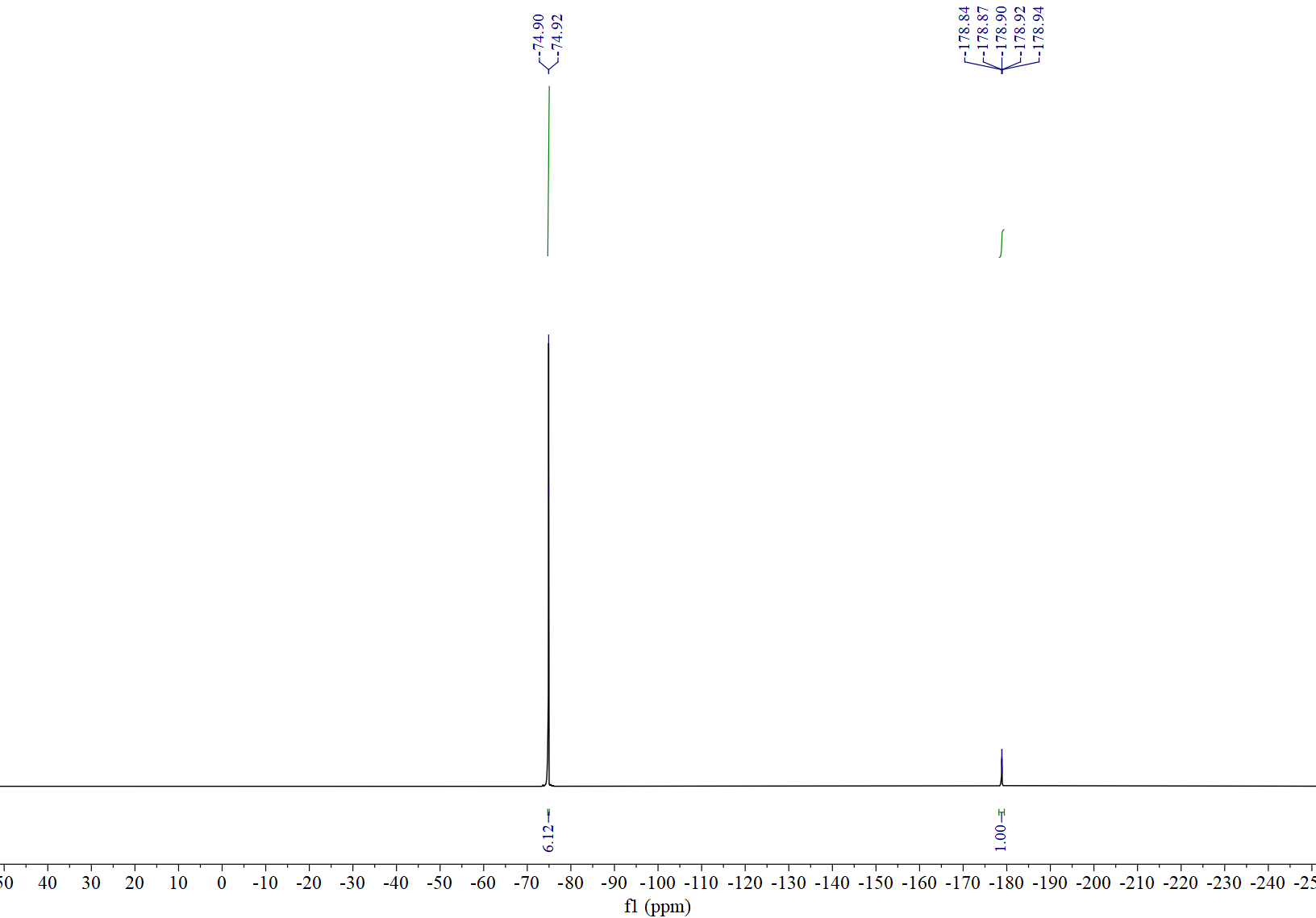

**^19^F NMR** of **4** (376 MHz, Chloroform-*d*, 298 K)


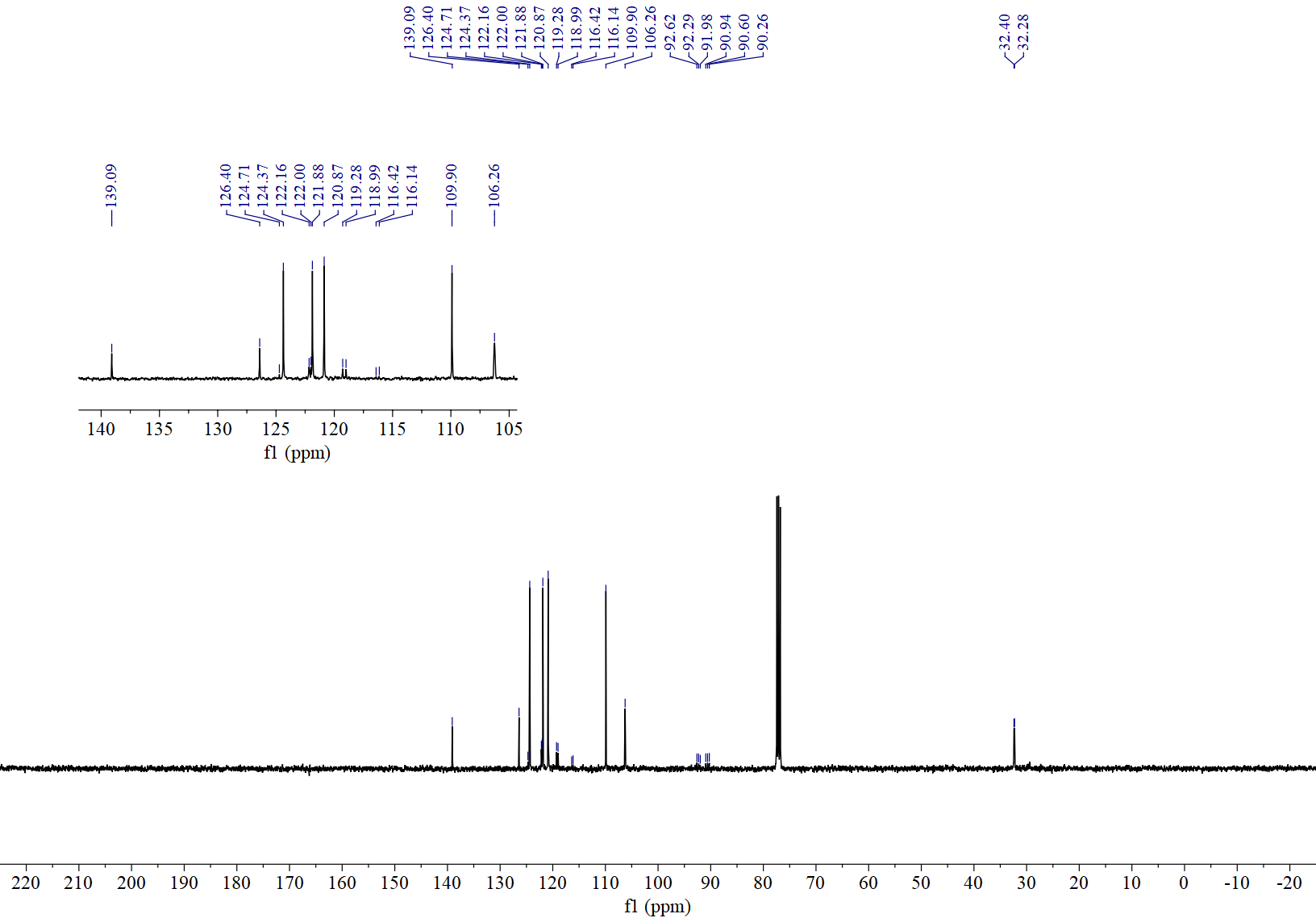

**^13^C NMR** of **4** (101 MHz, Chloroform-*d*, 298 K)


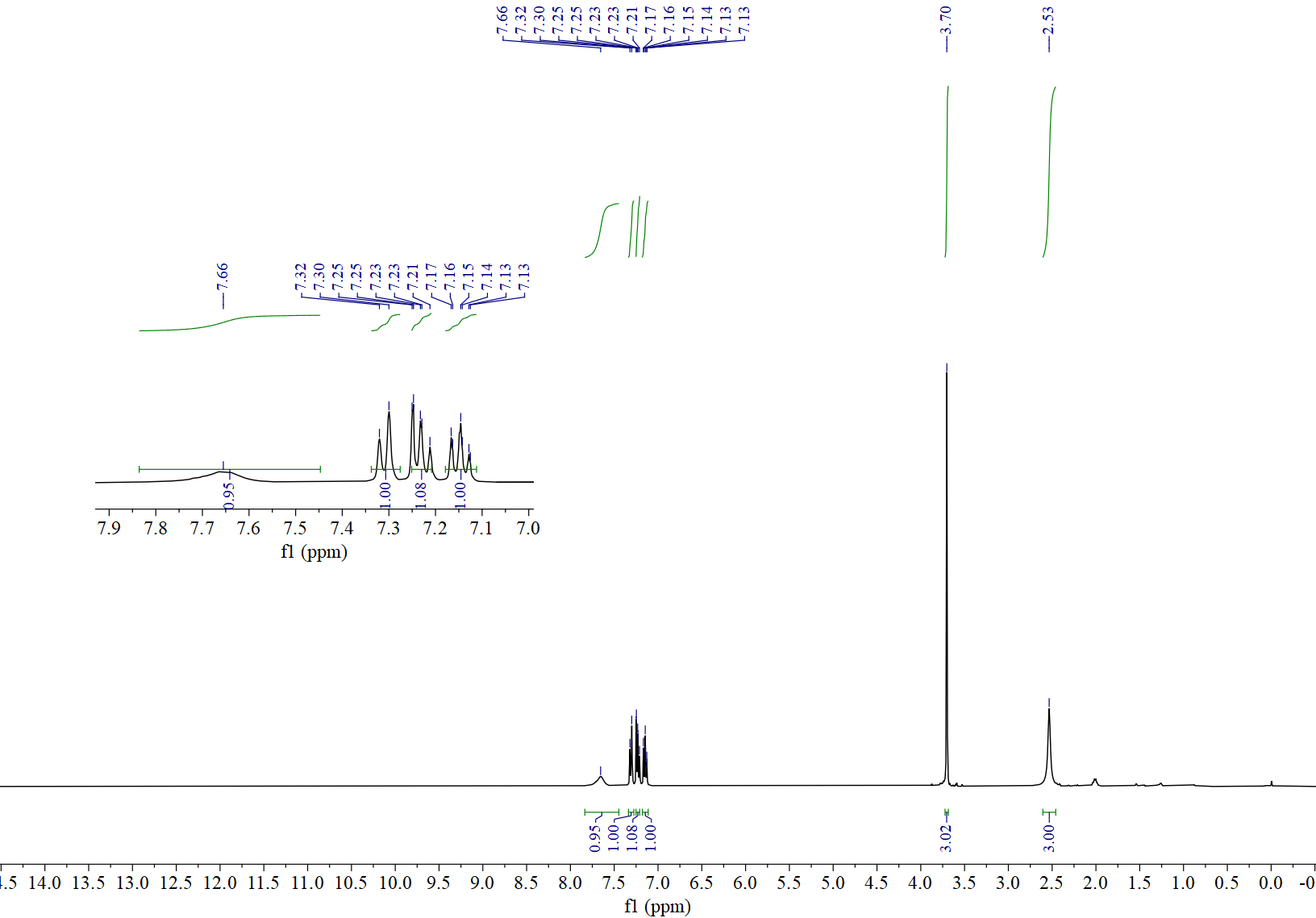

**^1^H NMR** of **5** (400 MHz, Chloroform-*d*, 298 K)


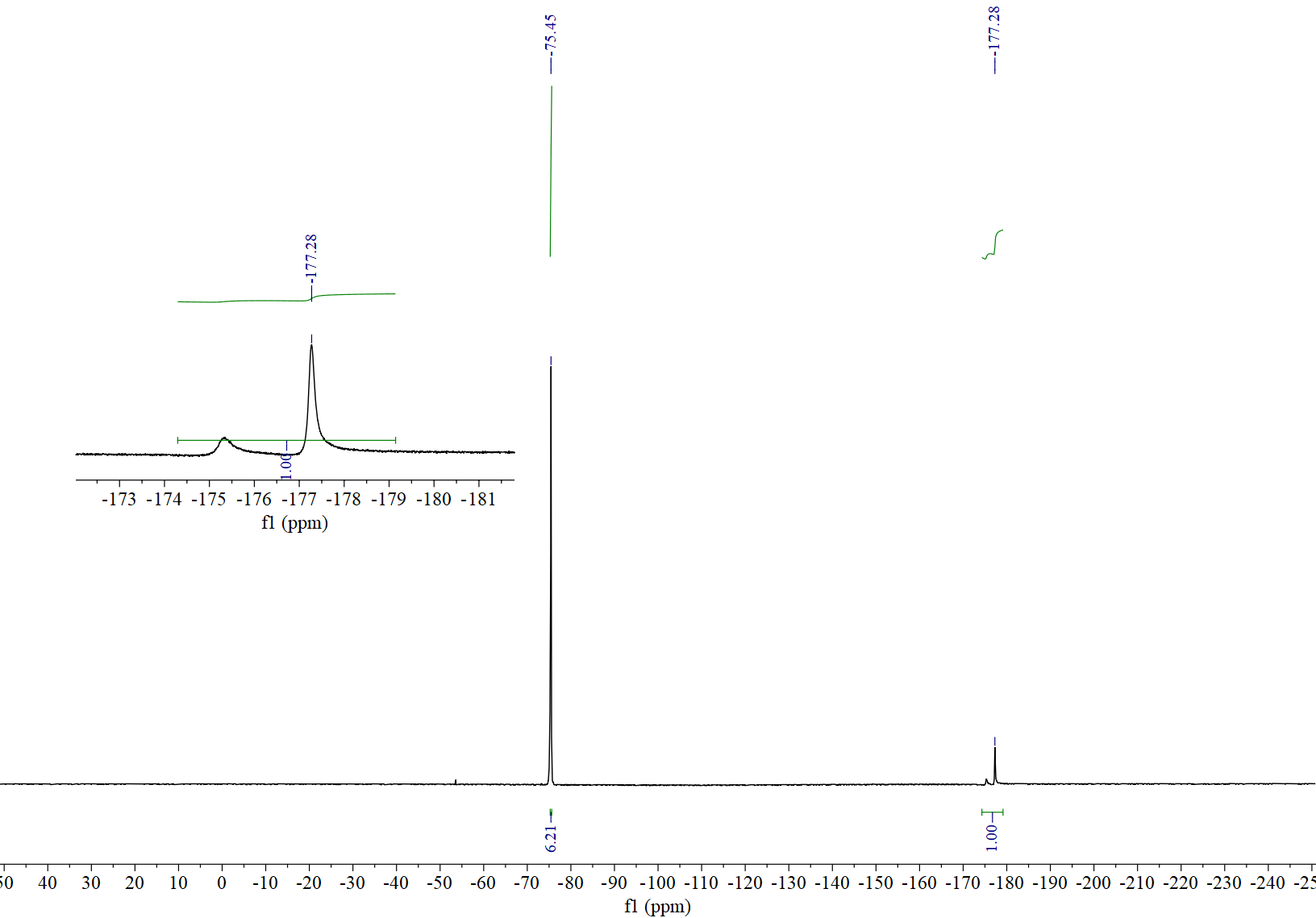

**^19^F NMR** of **5** (376 MHz, Chloroform-*d*, 298 K)


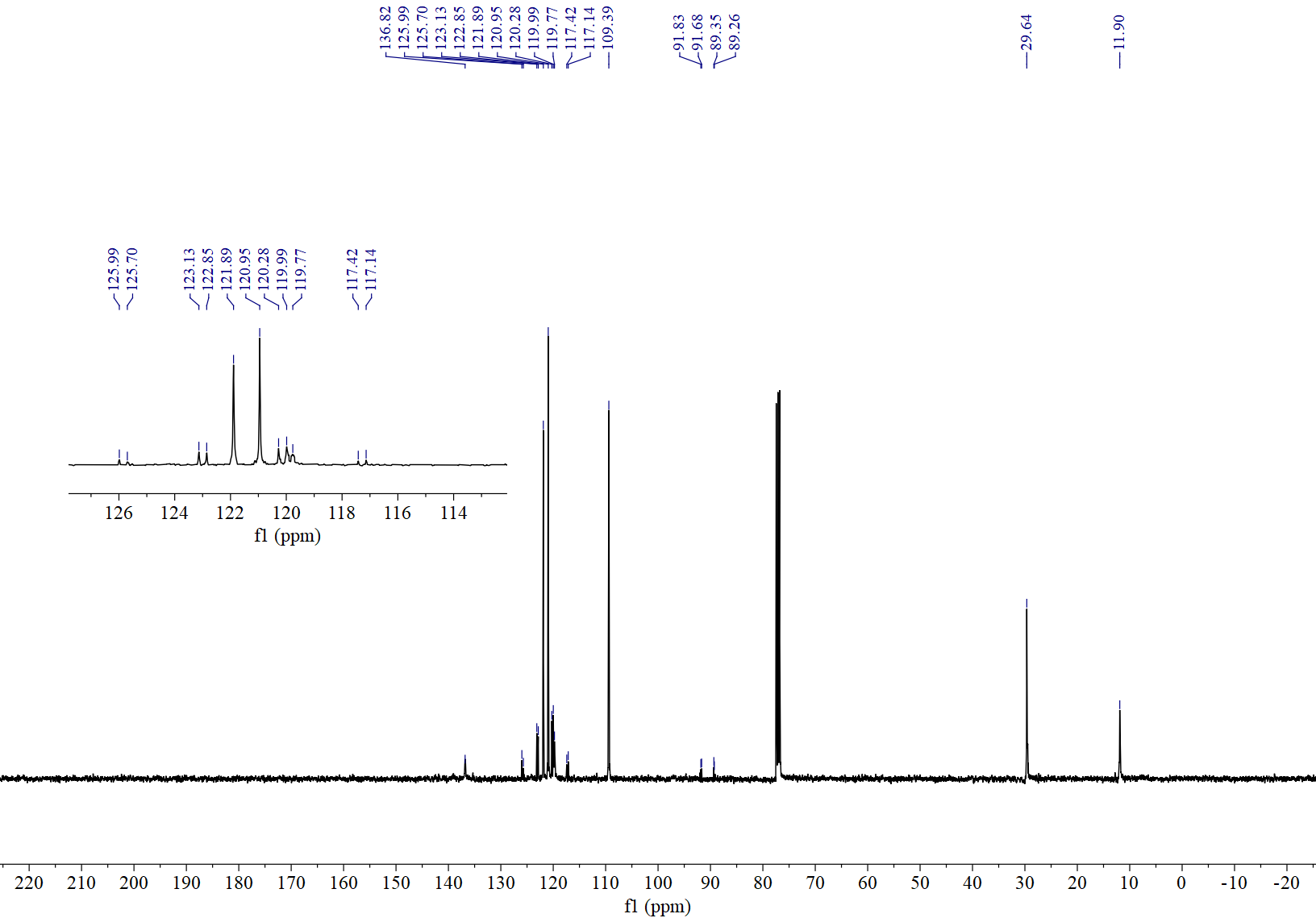

**^13^C NMR** of **5** (101 MHz, Chloroform-*d*, 298 K)


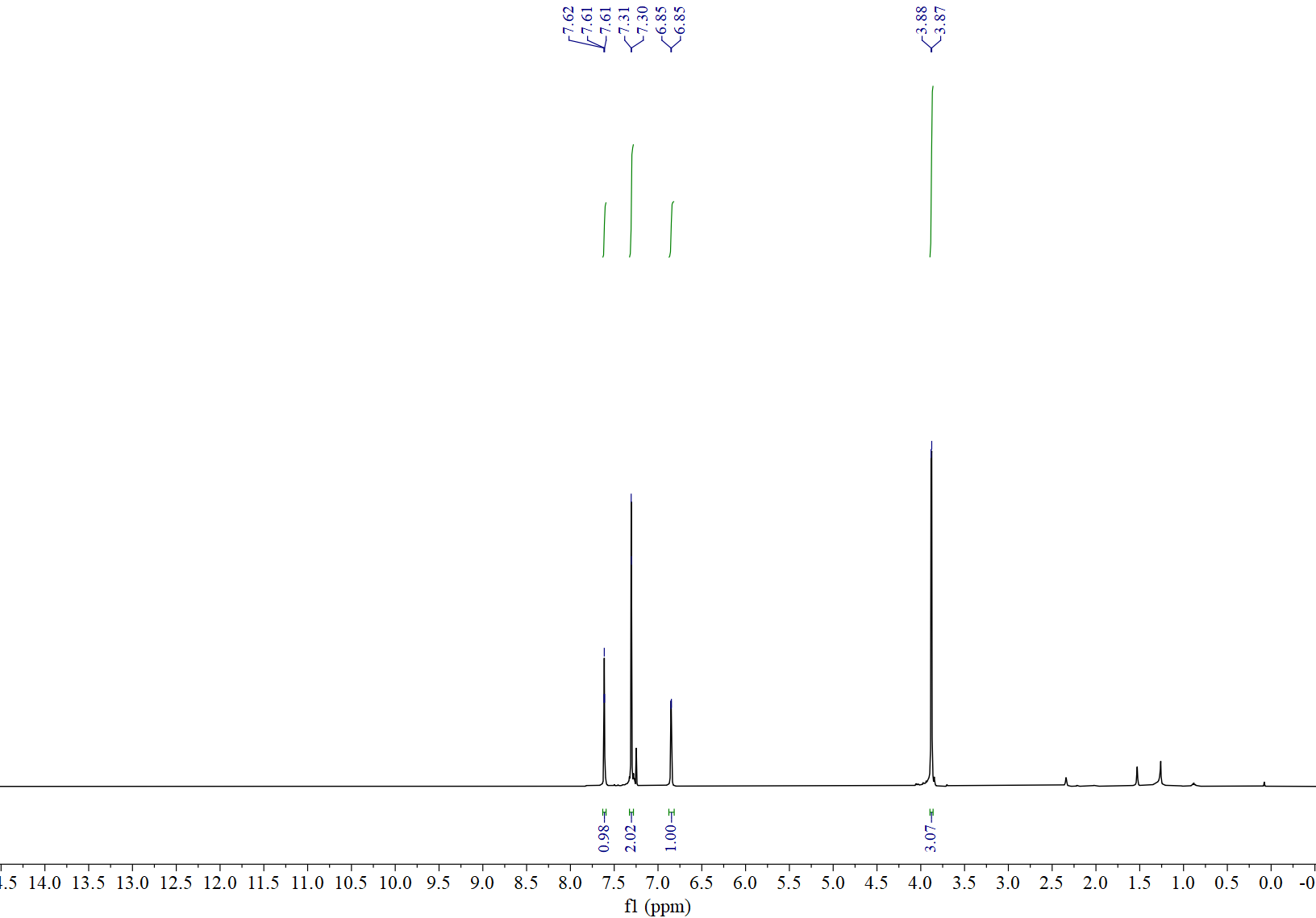

**^1^H NMR** of **6** (400 MHz, Chloroform-*d*, 298 K)


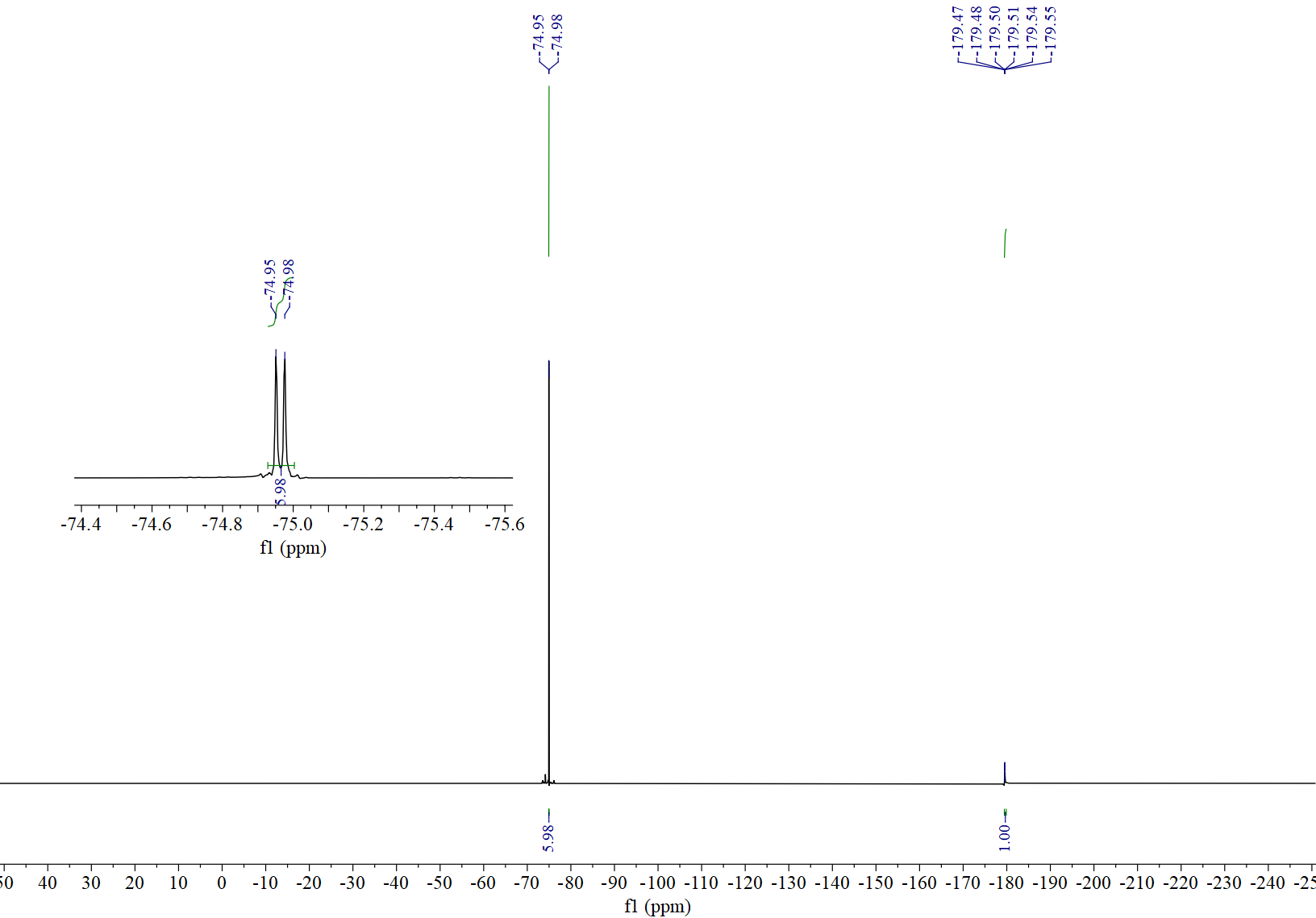

**^19^F NMR** of **6** (376 MHz, Chloroform-*d*, 298 K)


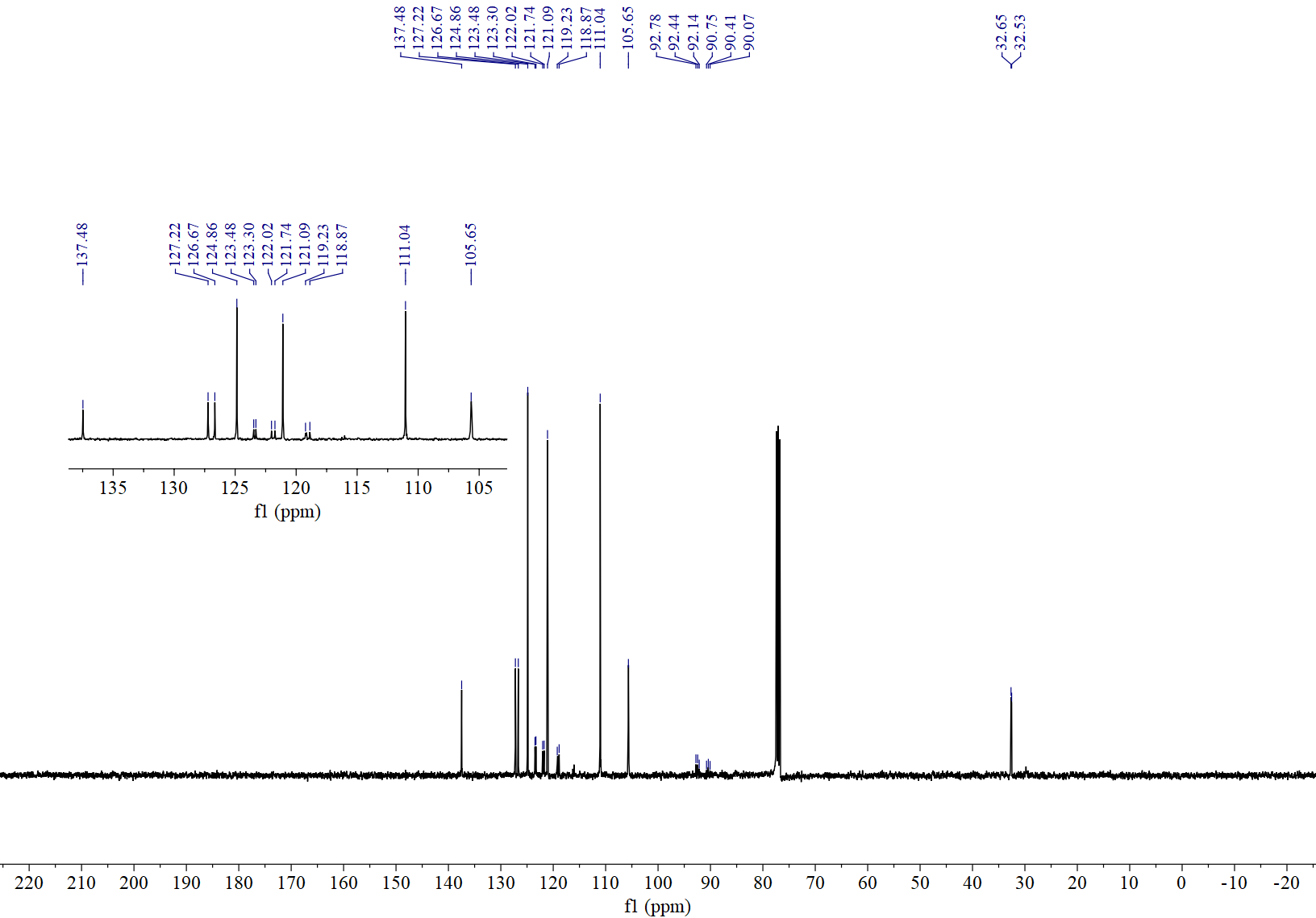

**^13^C NMR** of **6** (101 MHz, Chloroform-*d*, 298 K)


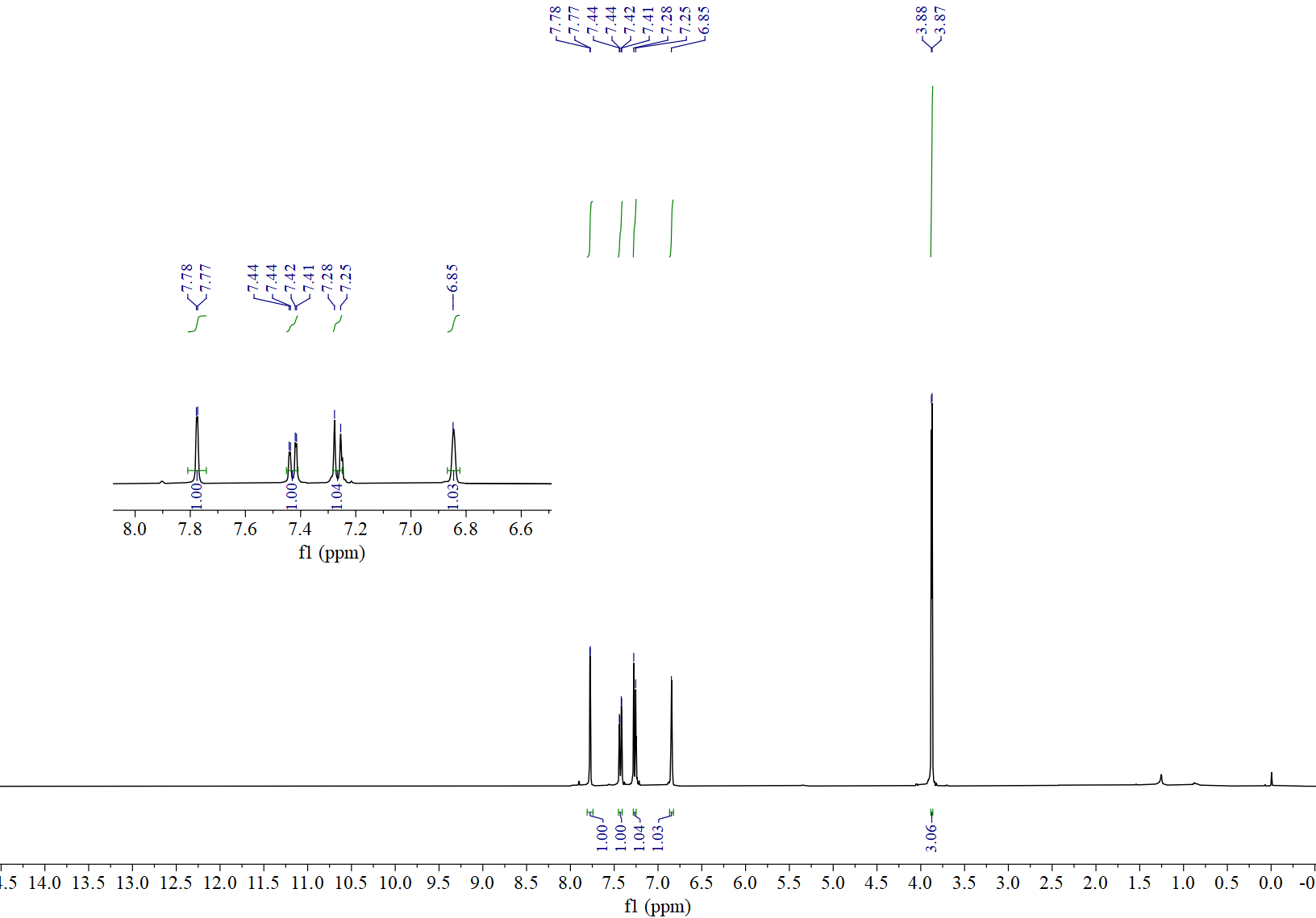

**^1^H NMR** of **7** (400 MHz, Chloroform-*d*, 298 K)


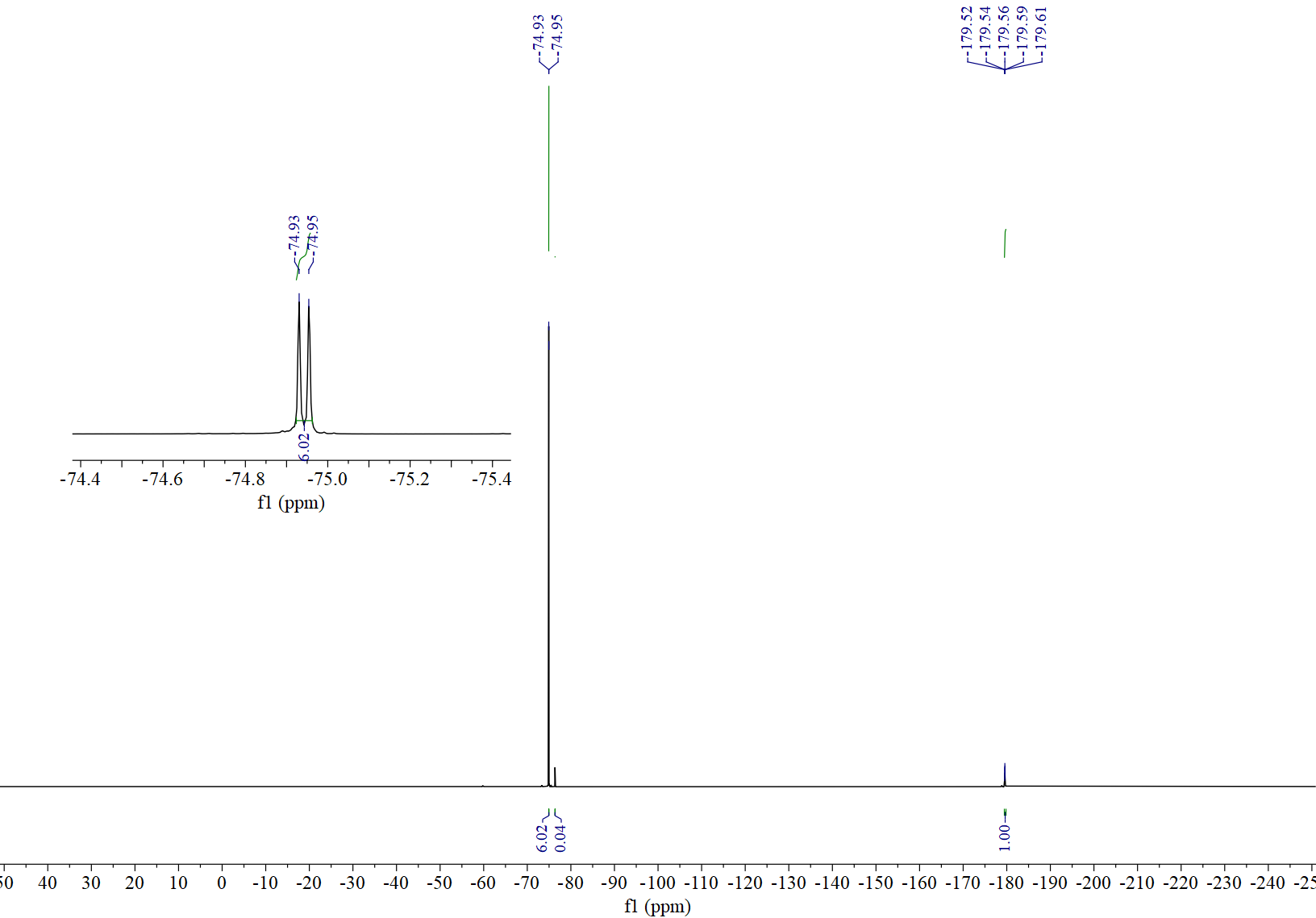

**^19^F NMR** of **7** (376 MHz, Chloroform-*d*, 298 K)


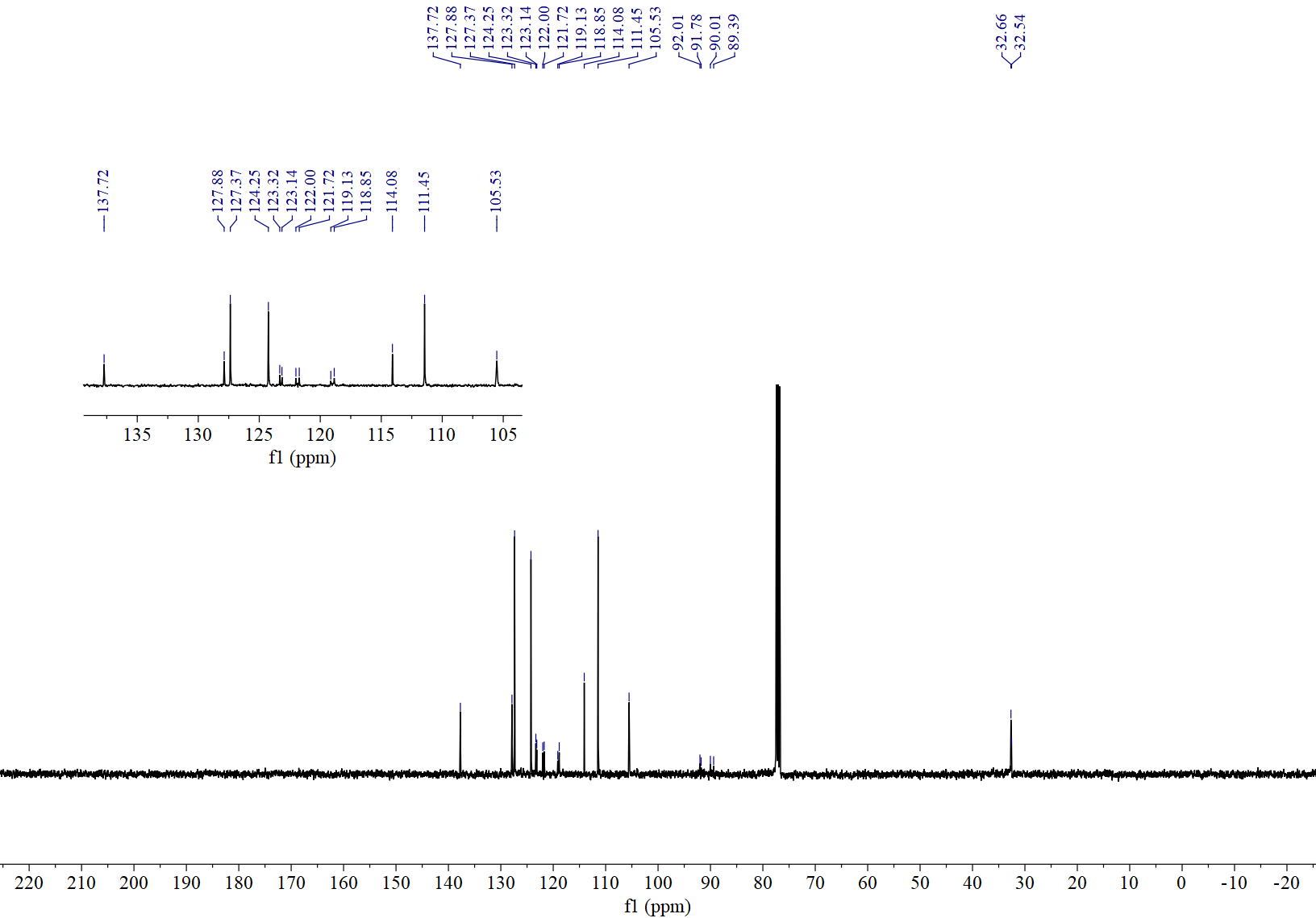

**^13^C NMR** of **7** (101 MHz, Chloroform-*d*, 298 K)


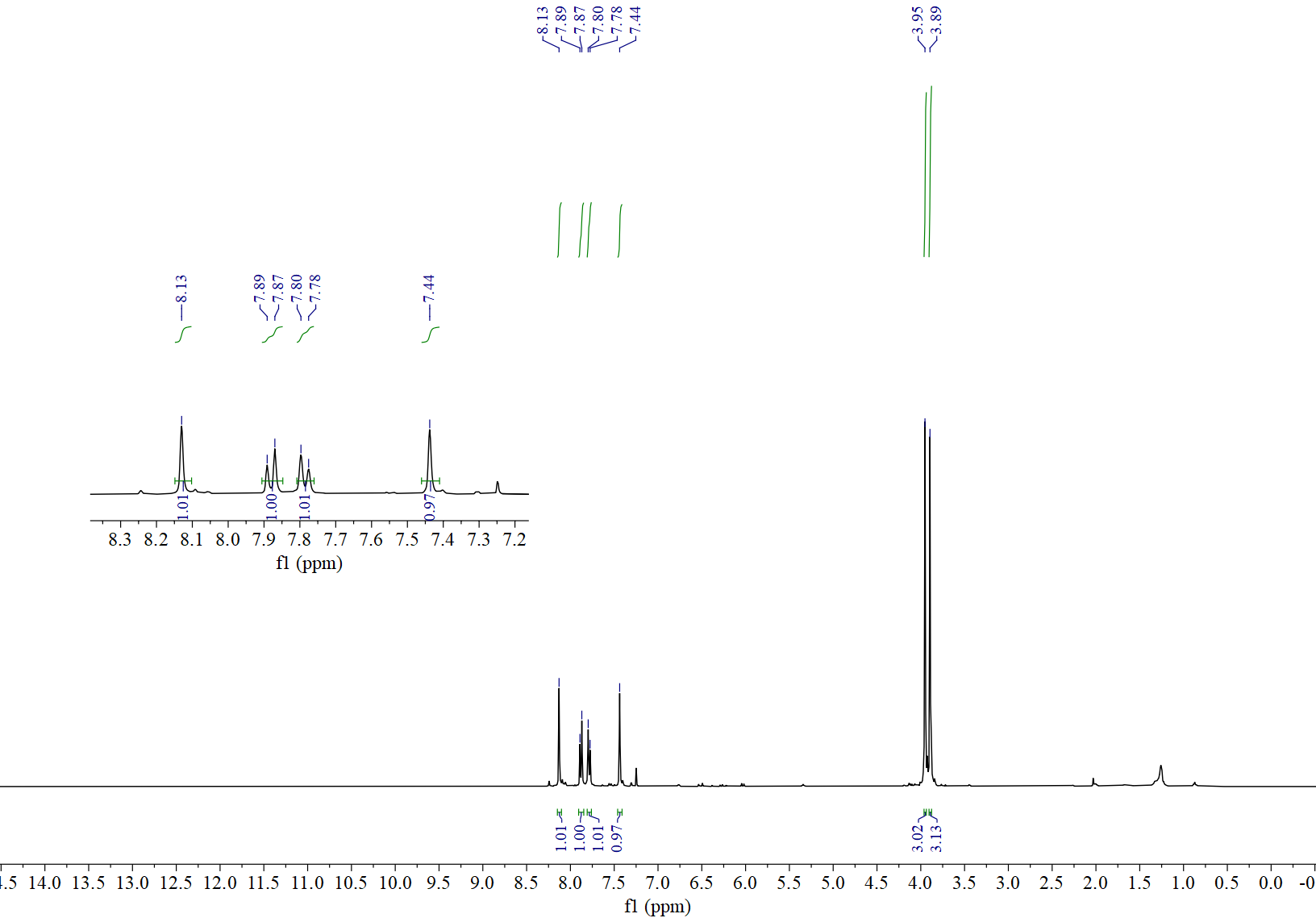

**^1^H NMR** of **8** (400 MHz, Chloroform-*d*, 298 K)


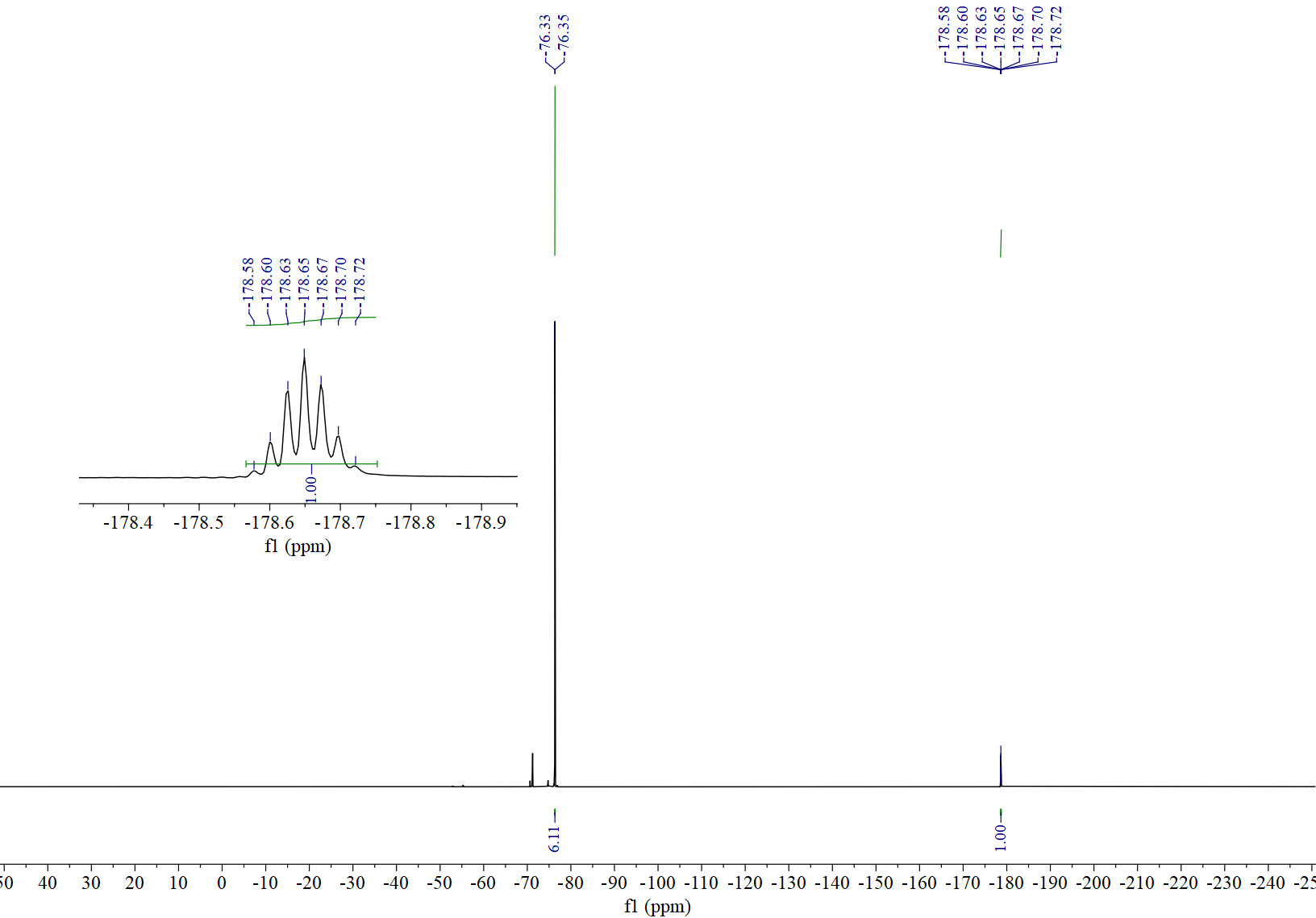

**^19^F NMR** of **8** (376 MHz, Chloroform-*d*, 298 K)


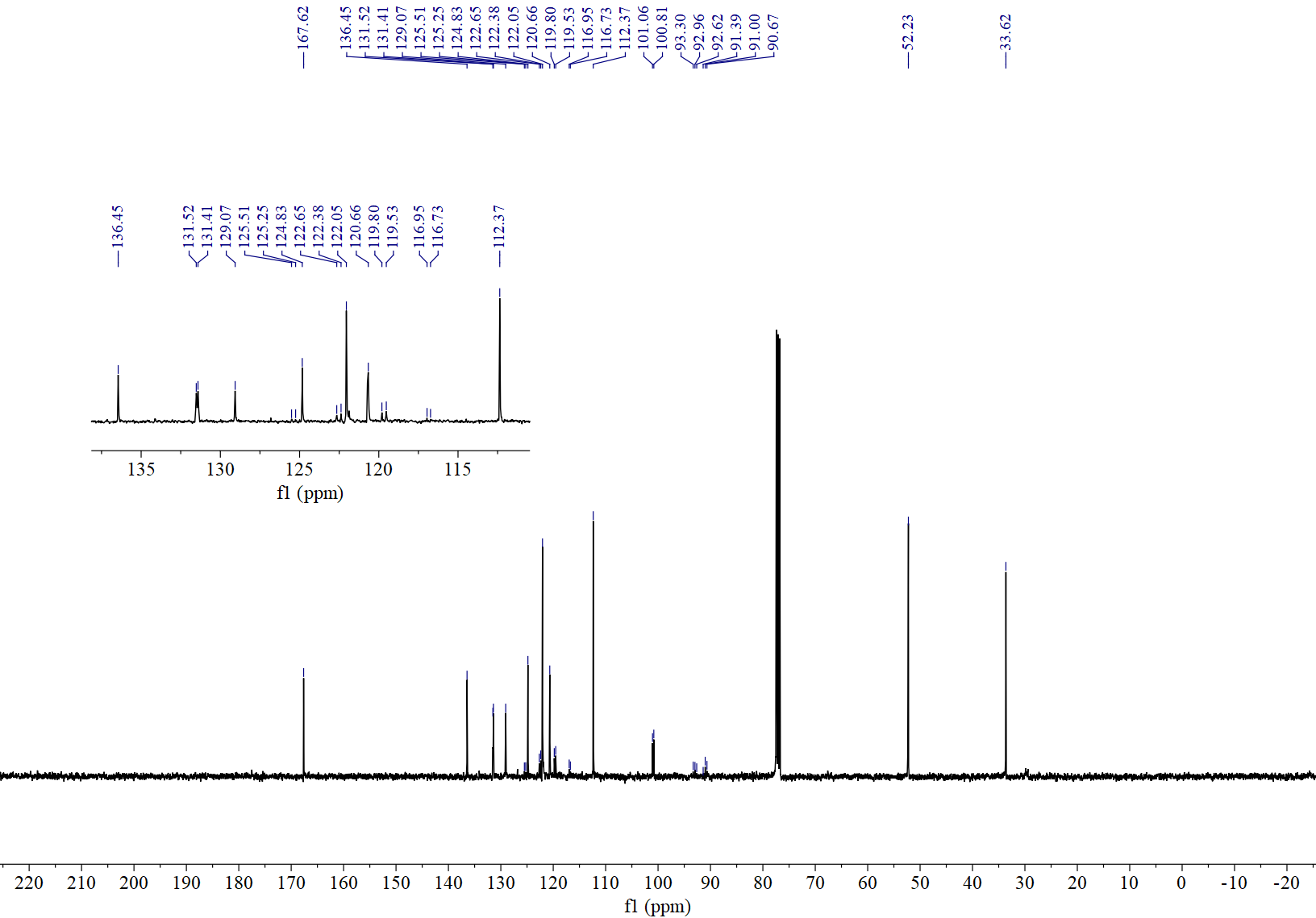

**^13^C NMR** of **8** (101 MHz, Chloroform-*d*, 298 K)


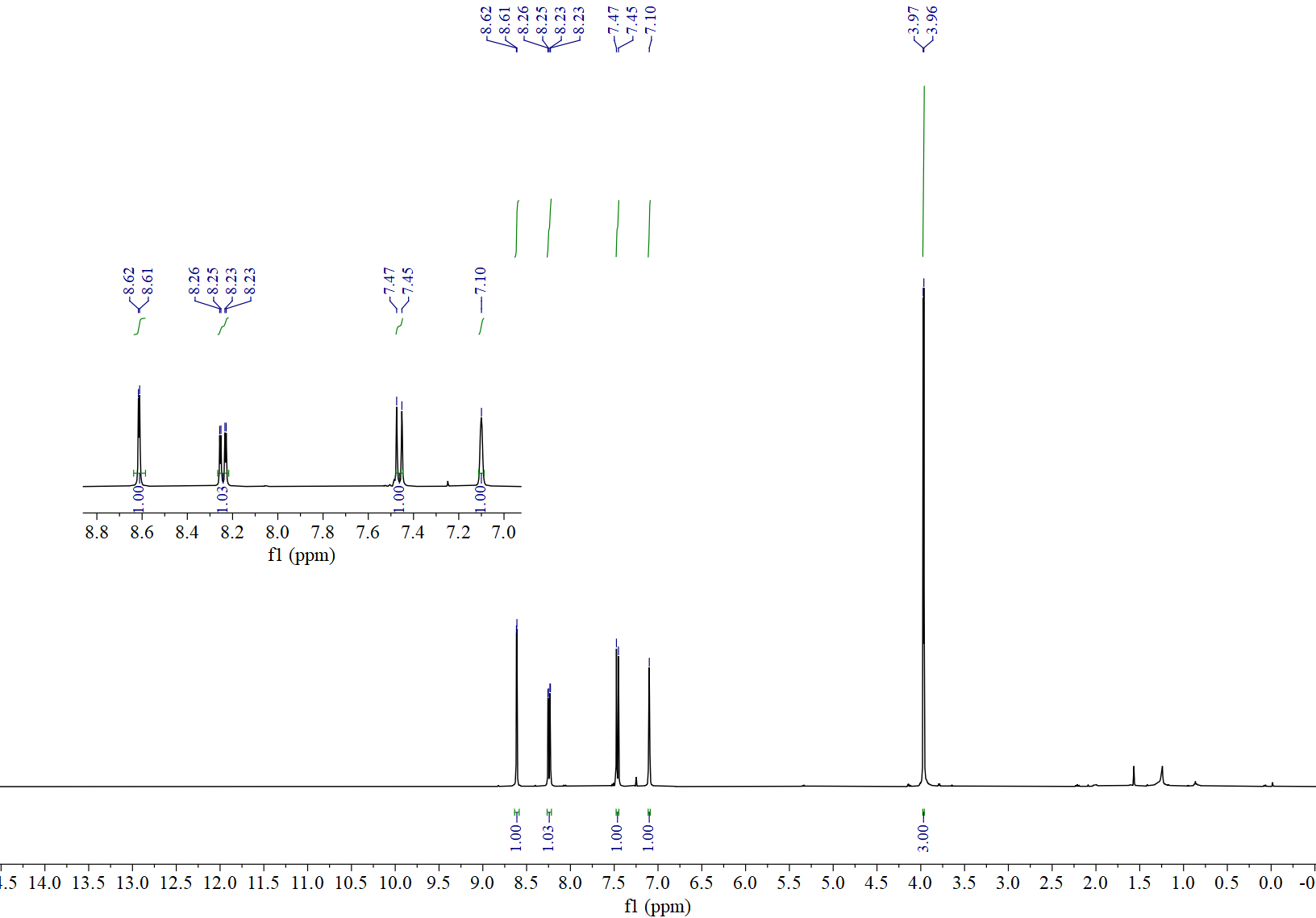

**^1^H NMR** of **9** (400 MHz, Chloroform-*d*, 298 K)


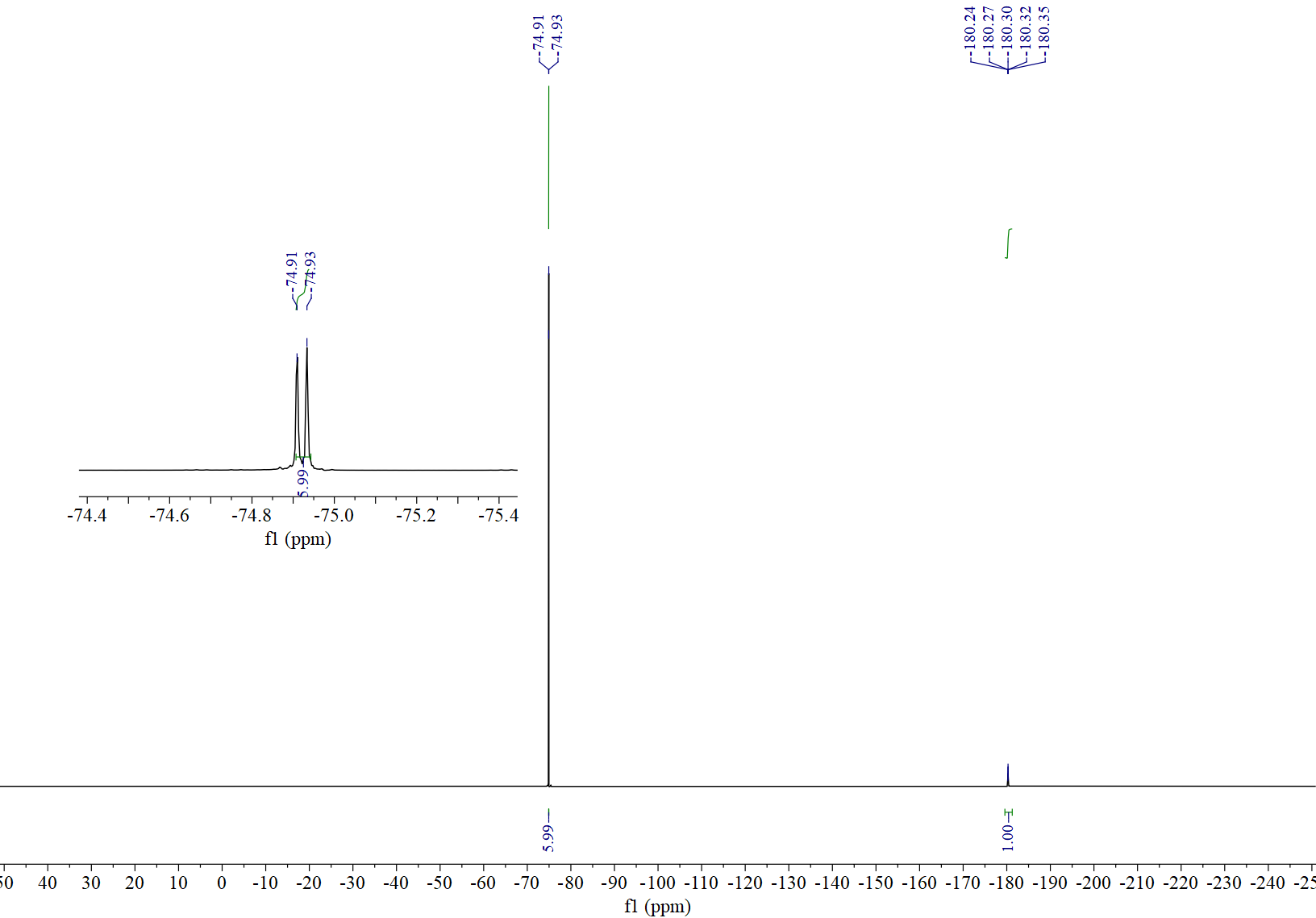

**^19^F NMR** of **9** (376 MHz, Chloroform-*d*, 298 K)


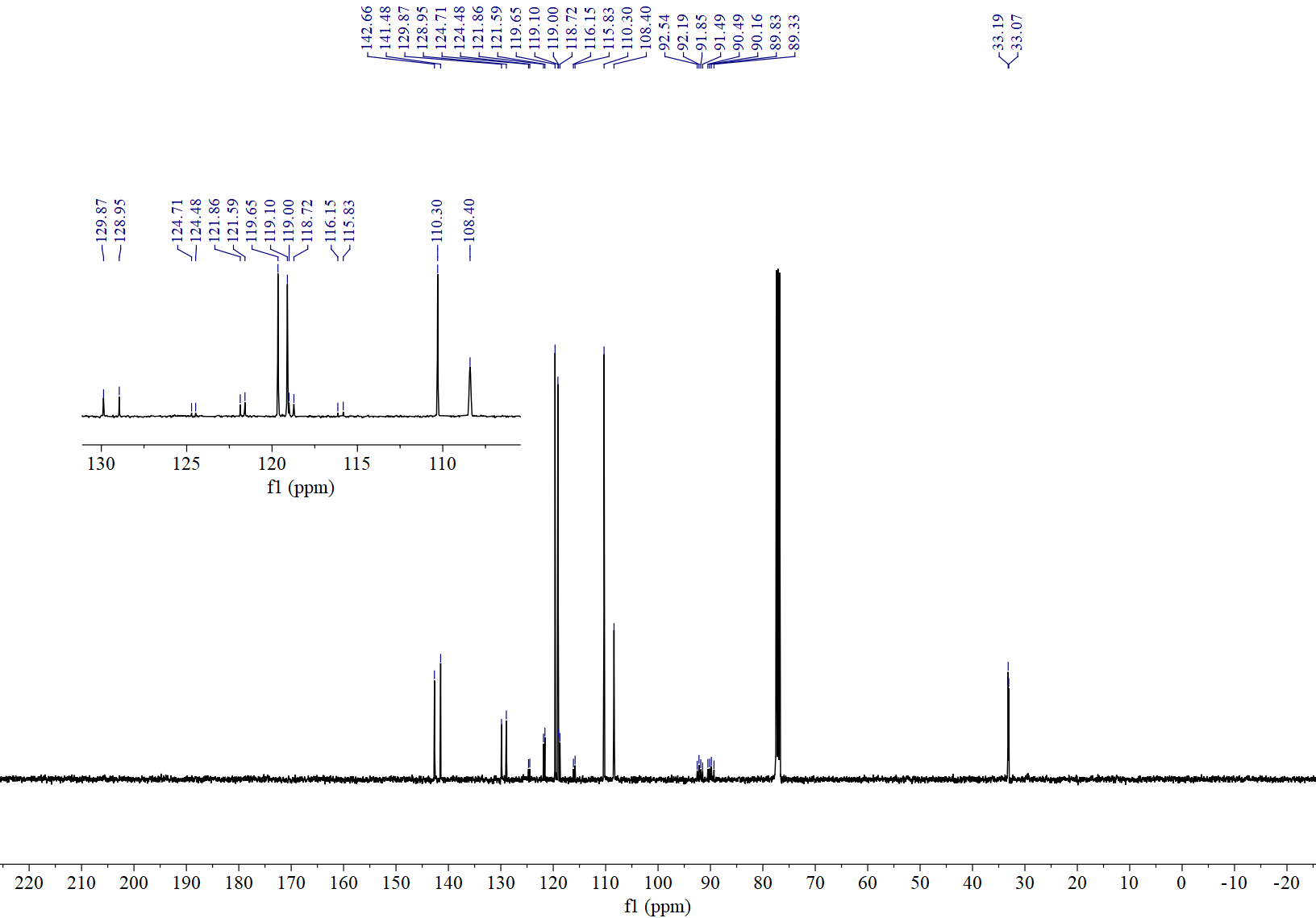

**^13^C NMR** of **9** (101 MHz, Chloroform-*d*, 298 K)


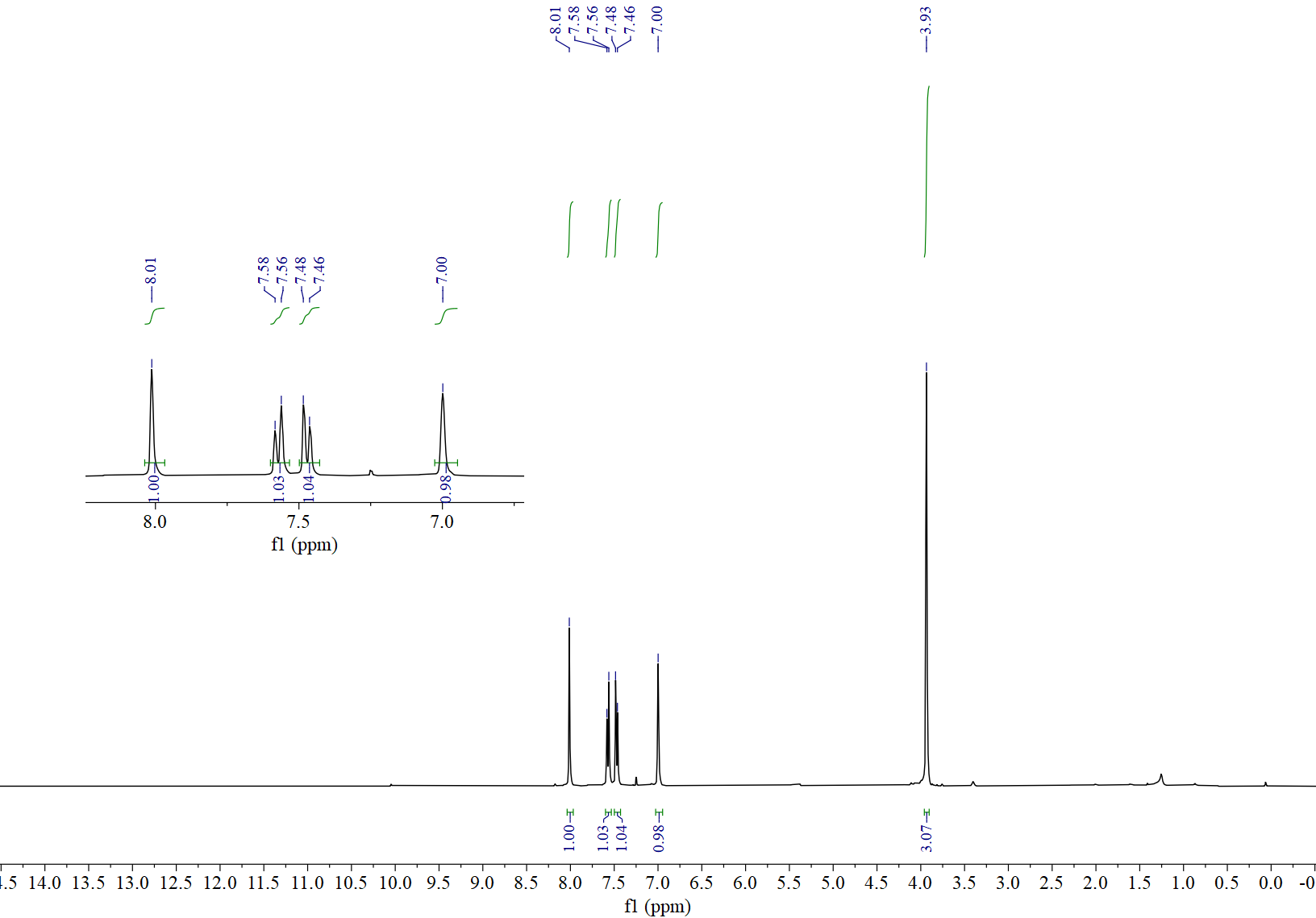

**^1^H NMR** of **10** (400 MHz, Chloroform-*d*, 298 K)


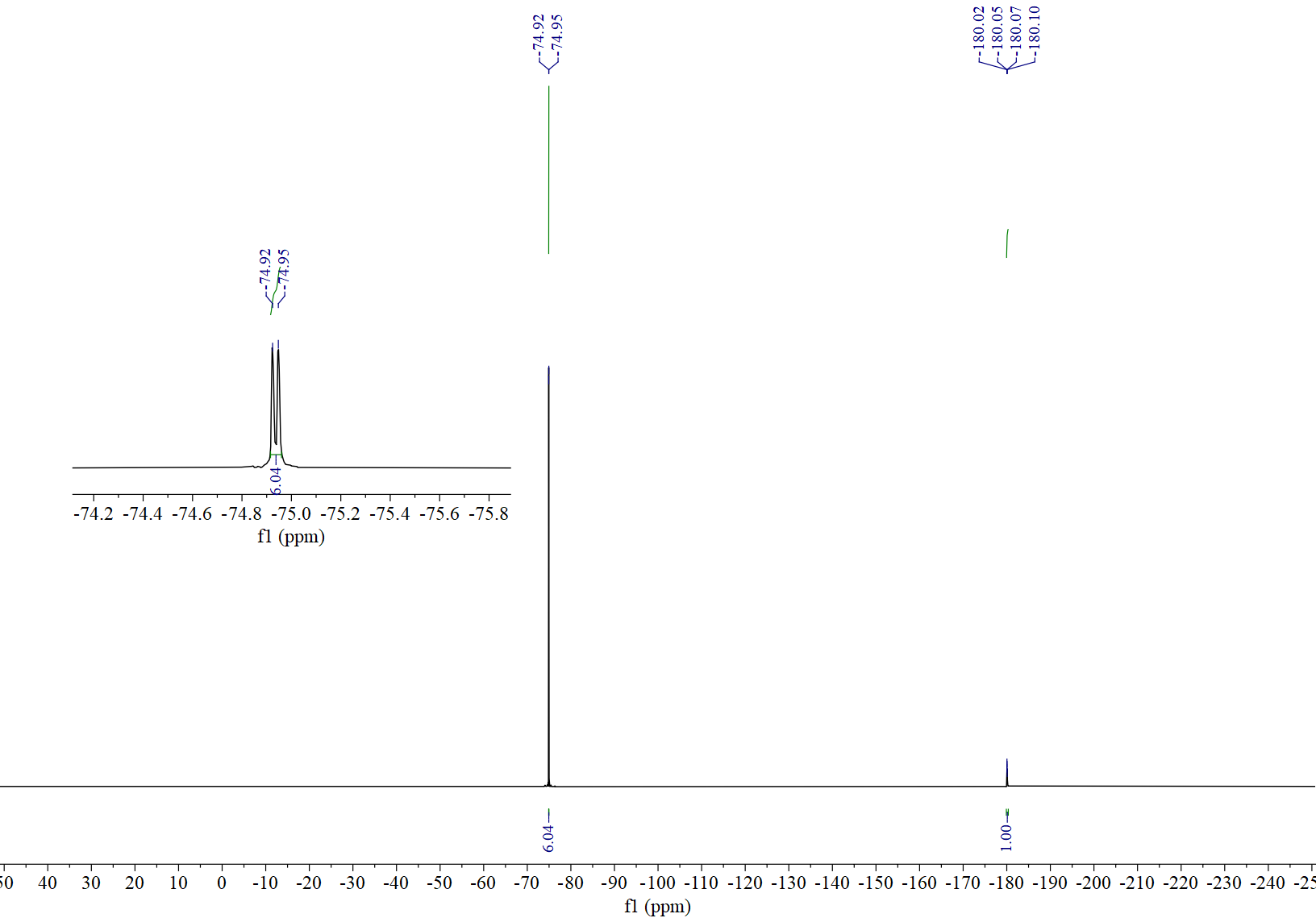

**^19^F NMR** of **10** (376 MHz, Chloroform-*d*, 298 K)


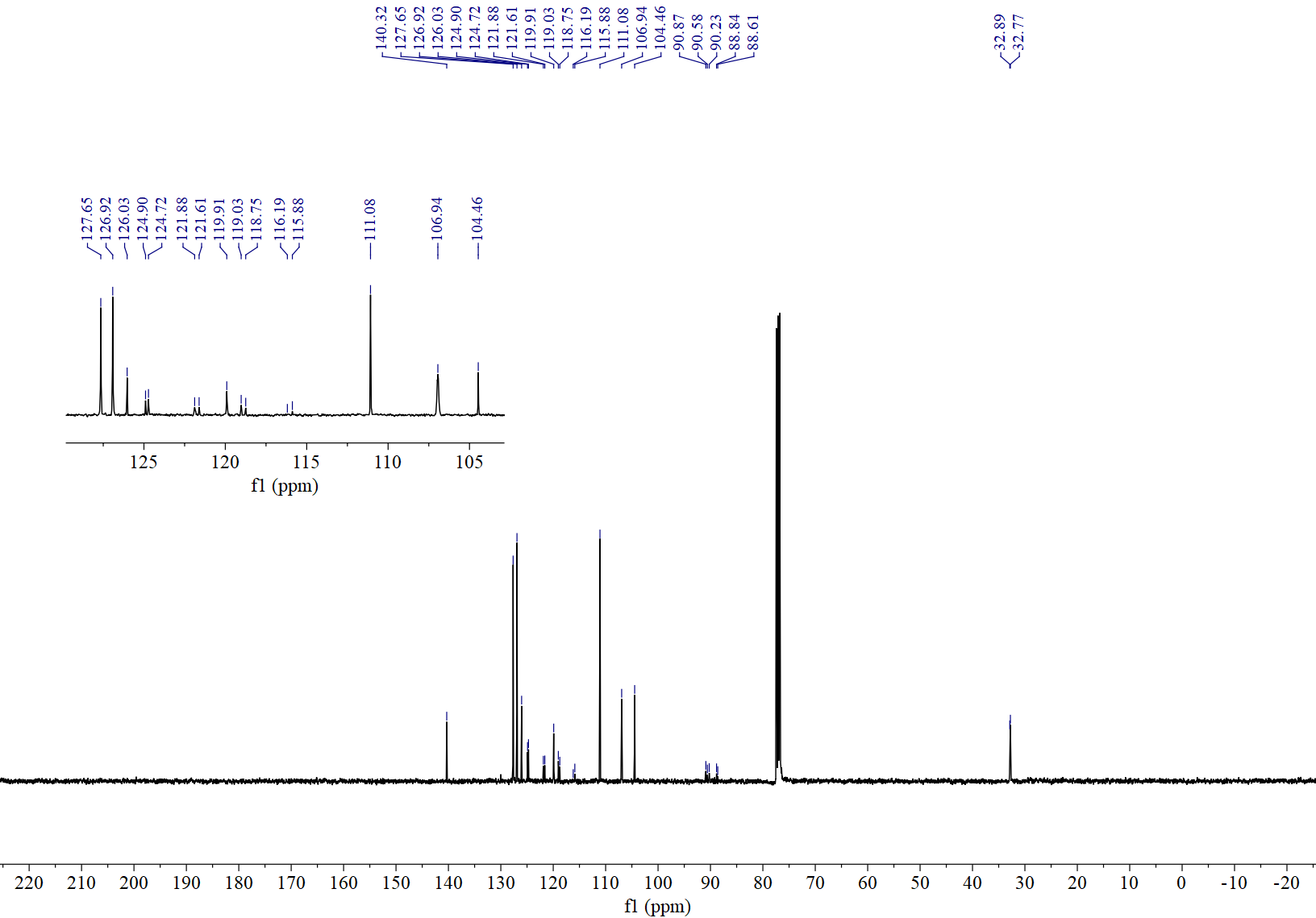

**^13^C NMR** of **10** (101 MHz, Chloroform-*d*, 298 K)


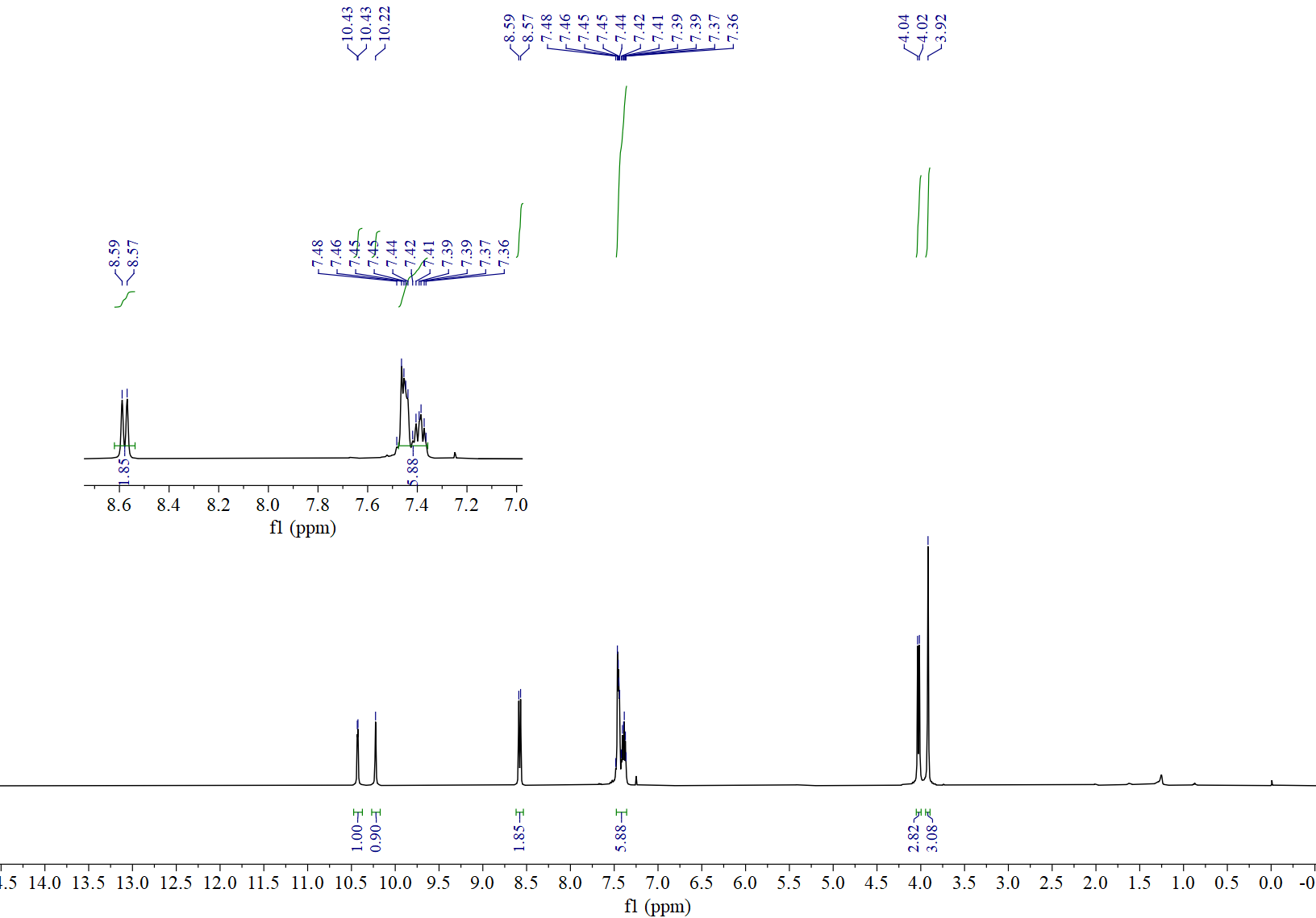

**^1^H NMR** of **11** (400 MHz, Chloroform-*d*, 298 K)


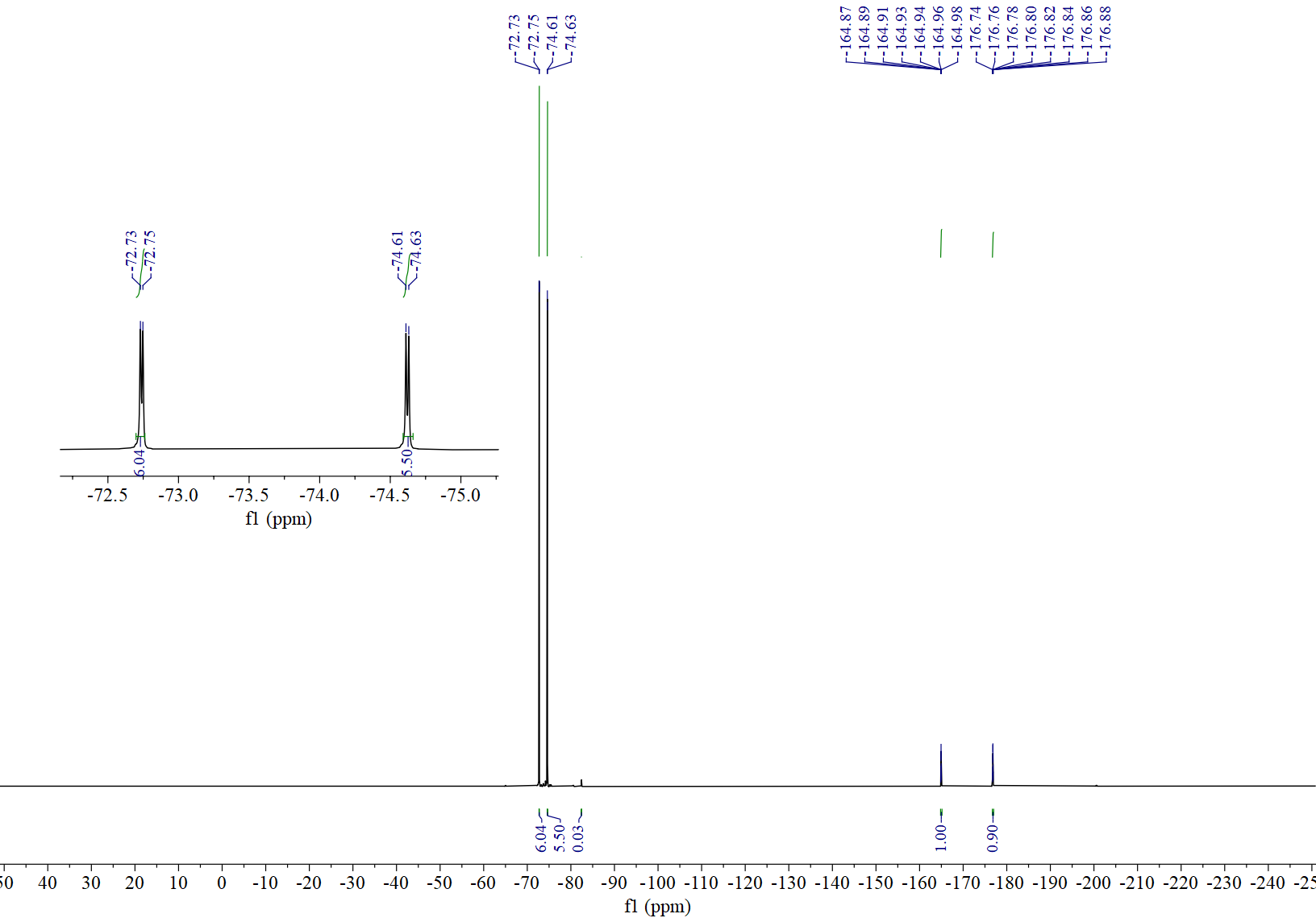

**^19^F NMR** of **11** (376 MHz, Chloroform-*d*, 298 K)


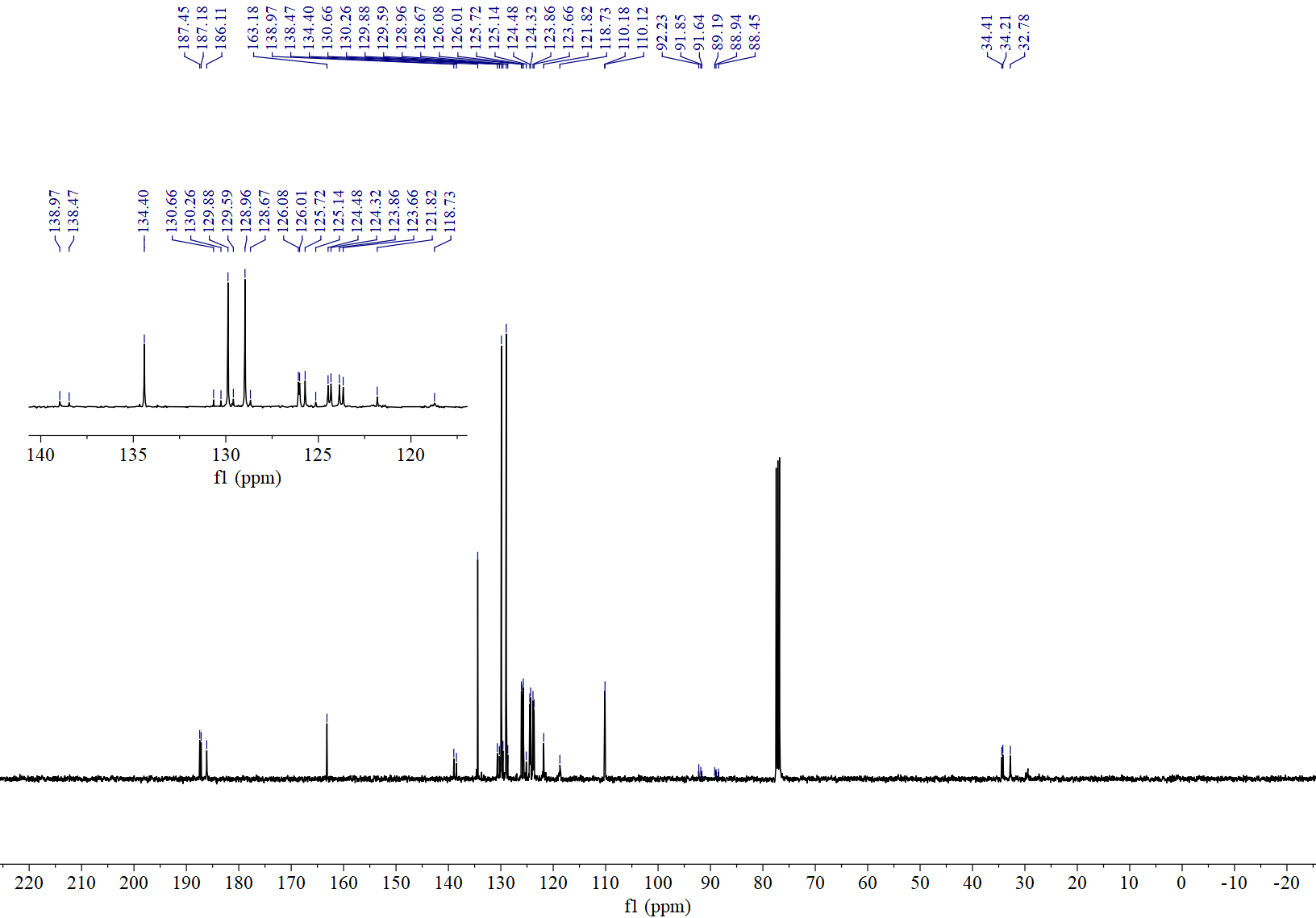

**^13^C NMR** of **11** (101 MHz, Chloroform-*d*, 298 K)


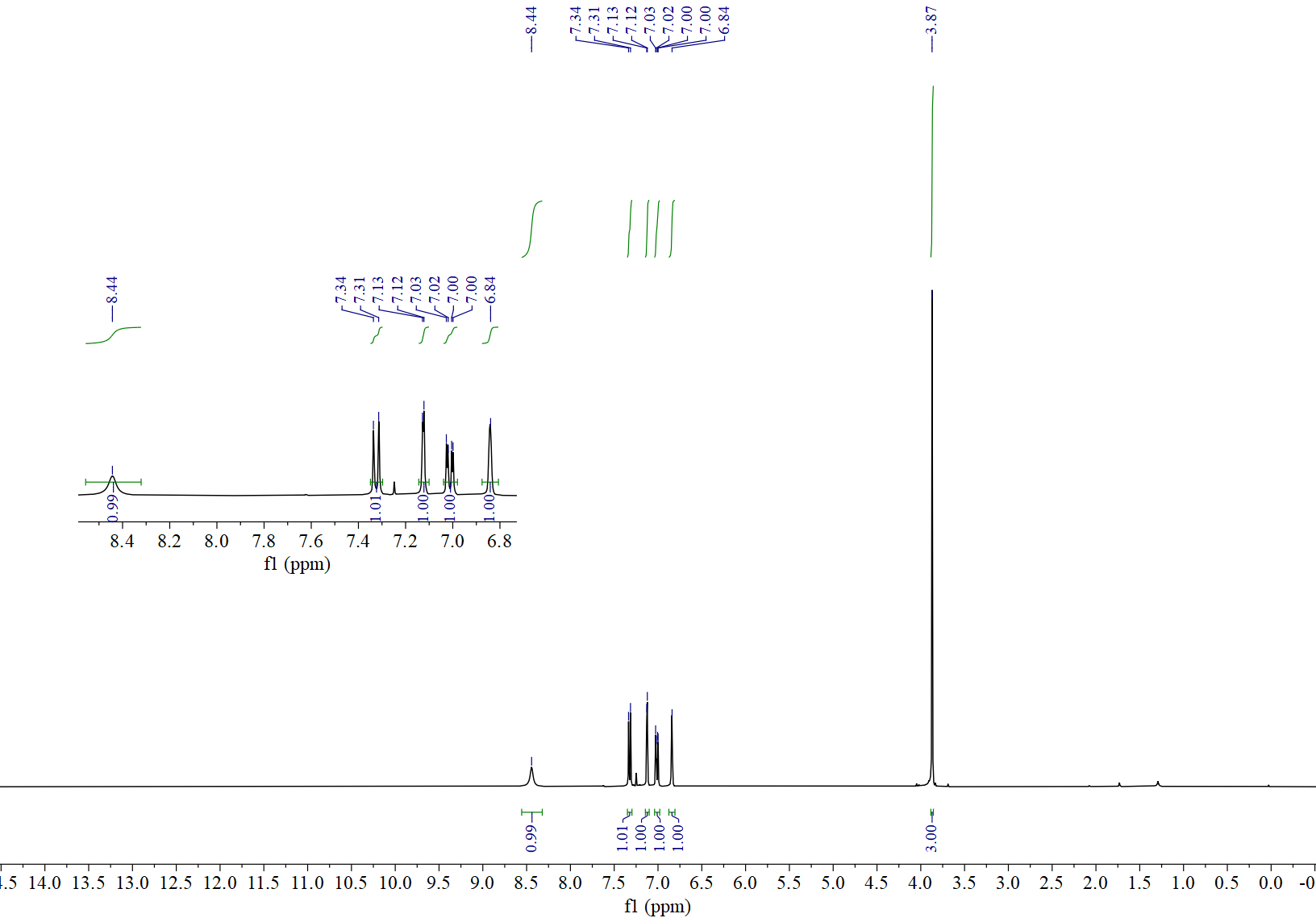

**^1^H NMR** of **12** (400 MHz, Chloroform-*d*, 298 K)


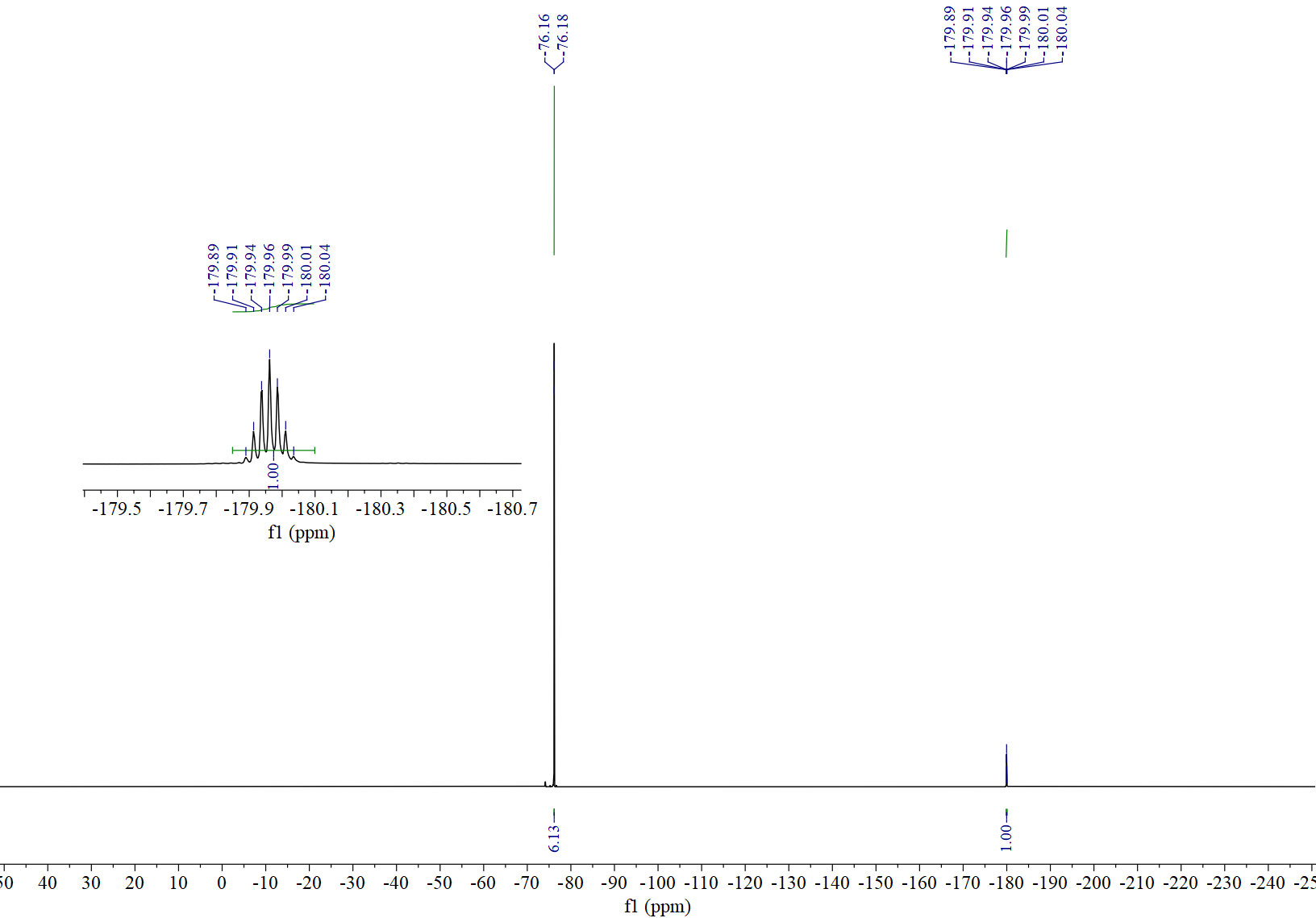

**^19^F NMR** of **12** (376 MHz, Chloroform-*d*, 298 K)


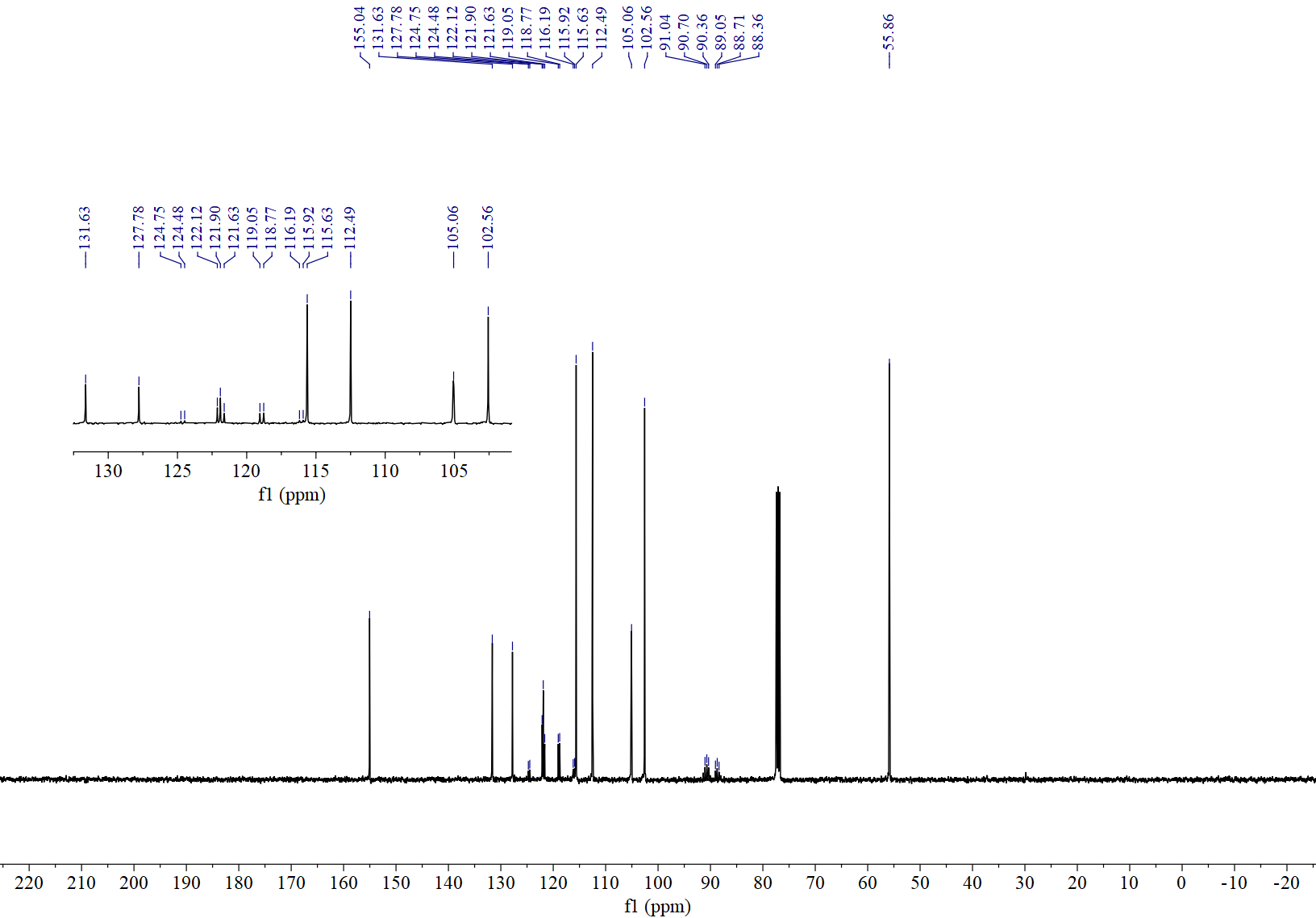

**^13^C NMR** of **12** (101 MHz, Chloroform-*d*, 298 K)


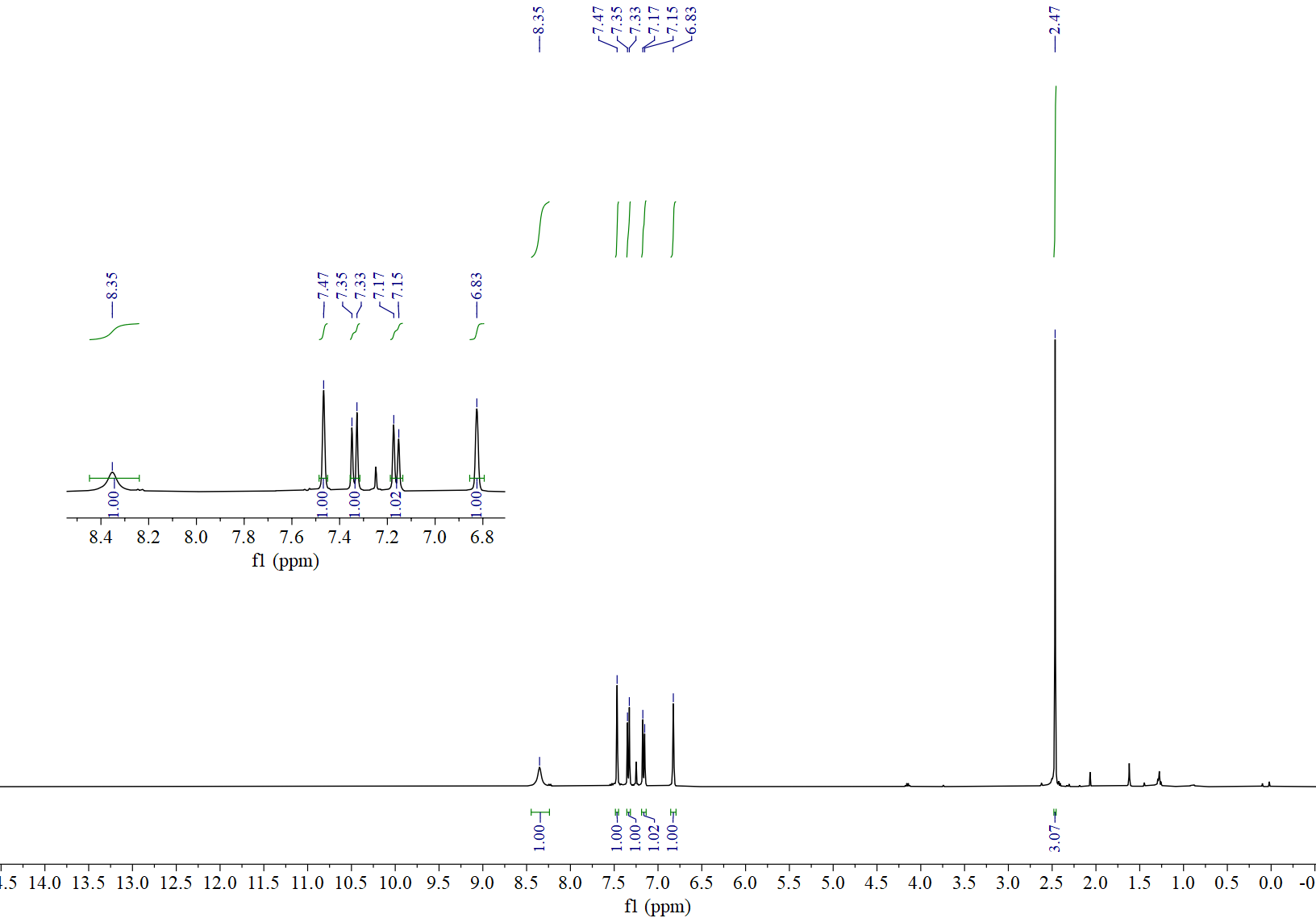

**^1^H NMR** of **13** (400 MHz, Chloroform-*d*, 298 K)


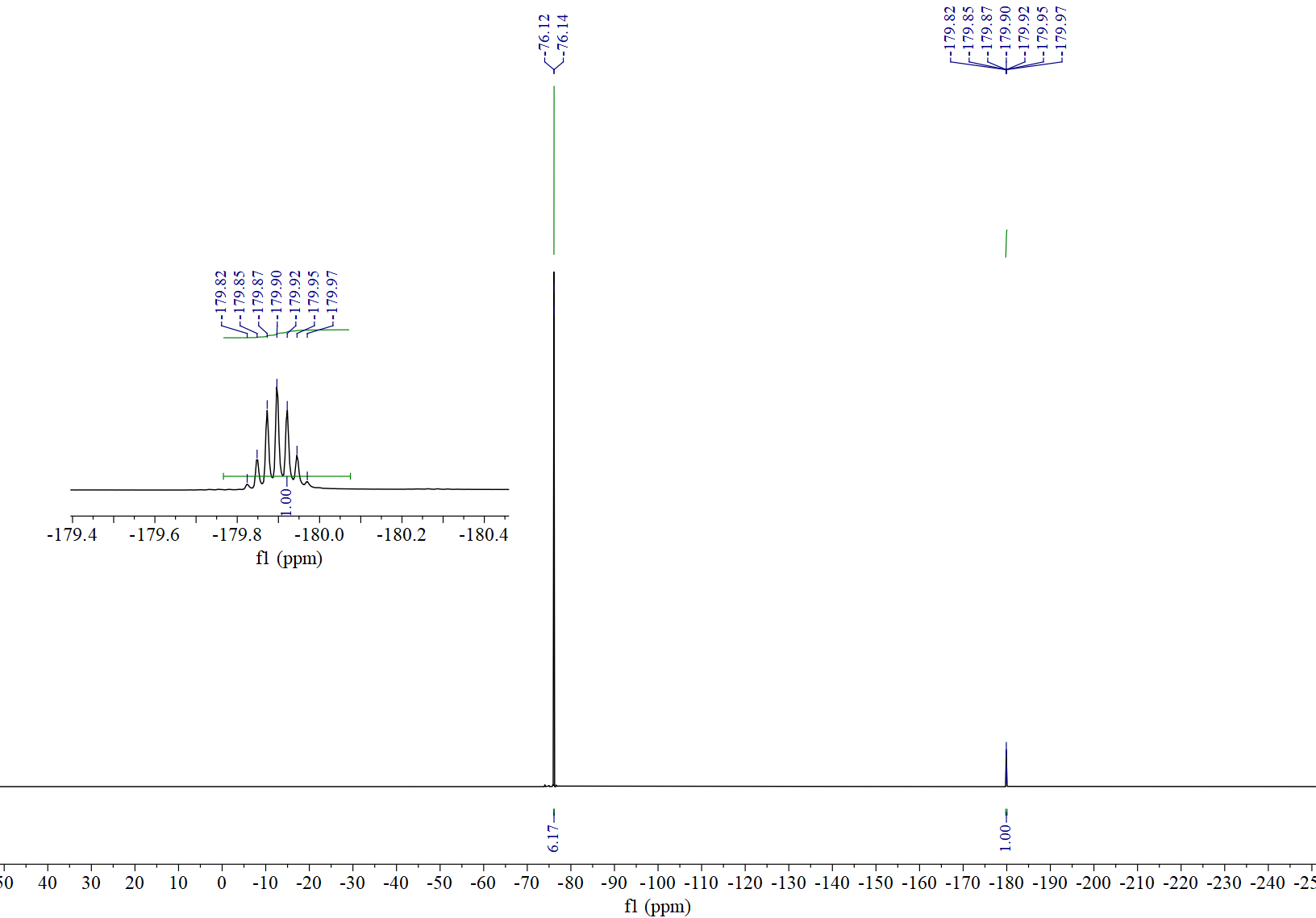

**^19^F NMR** of **13** (376 MHz, Chloroform-*d*, 298 K)


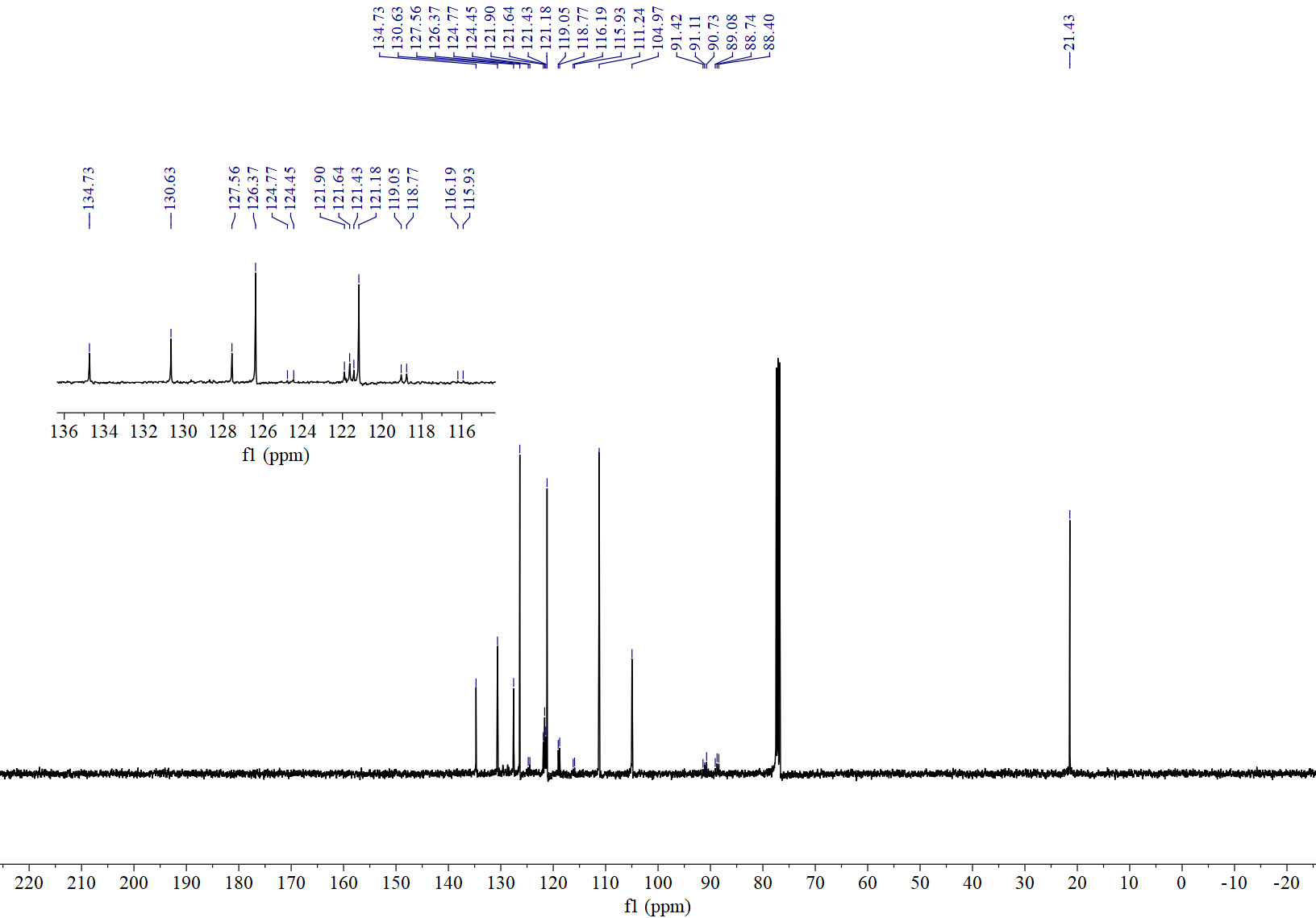

**^13^C NMR** of **13** (101 MHz, Chloroform-*d*, 298 K)


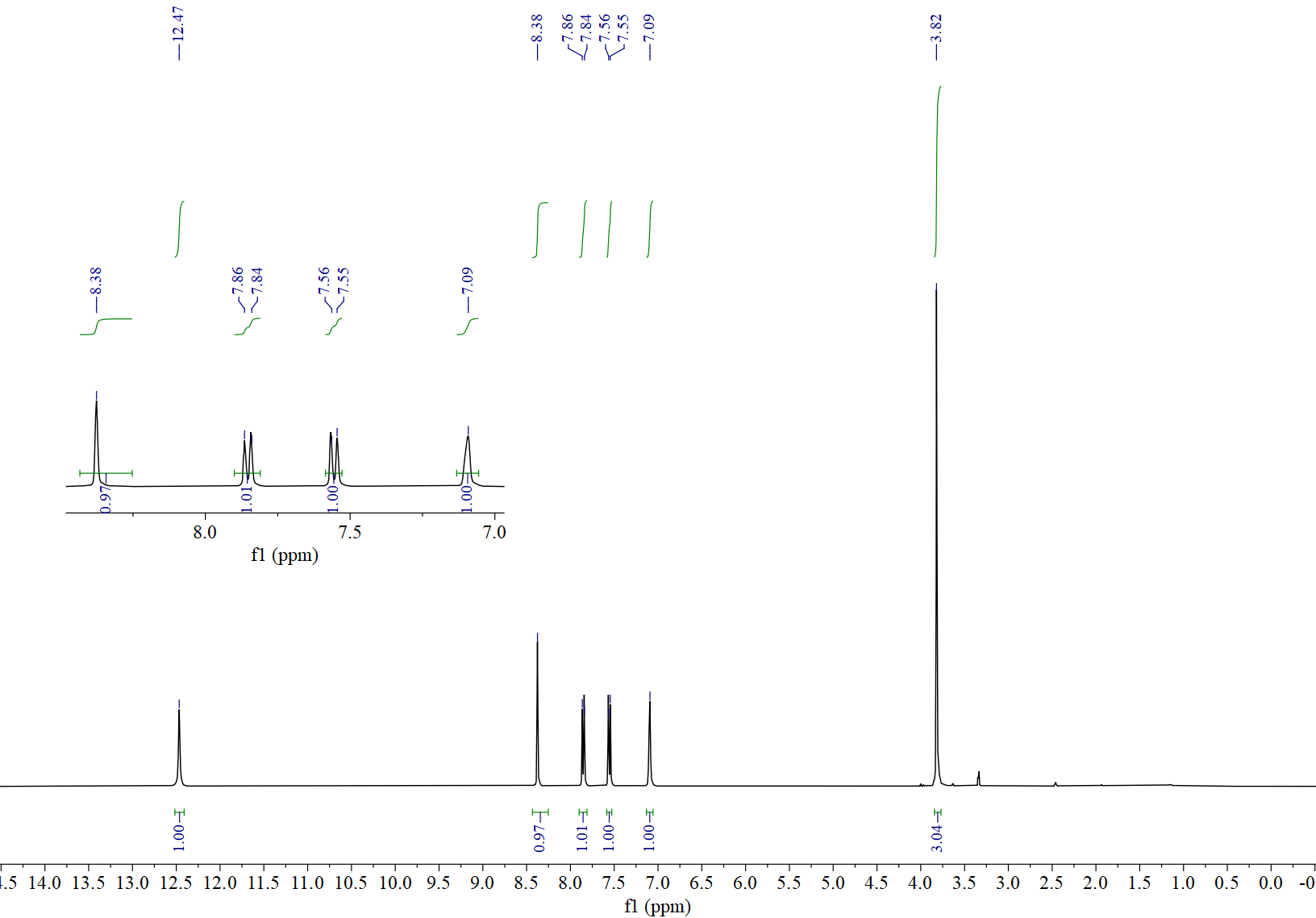

**^1^H NMR** of **14** (400 MHz, Dimethyl sulfoxide-*d_6_*, 298 K)


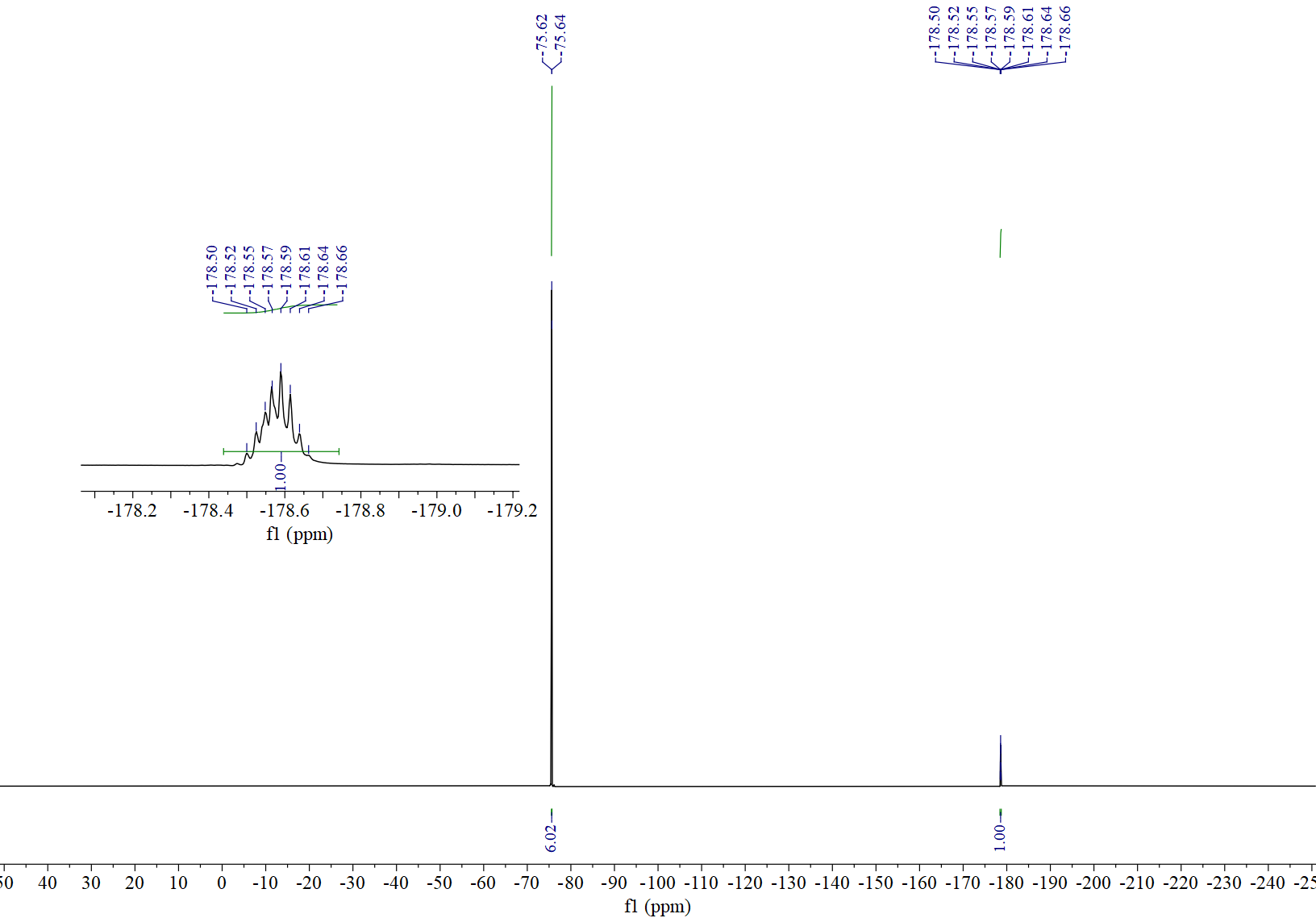

**^19^F NMR** of **14** (376 MHz, Dimethyl sulfoxide-*d_6_*, 298 K)


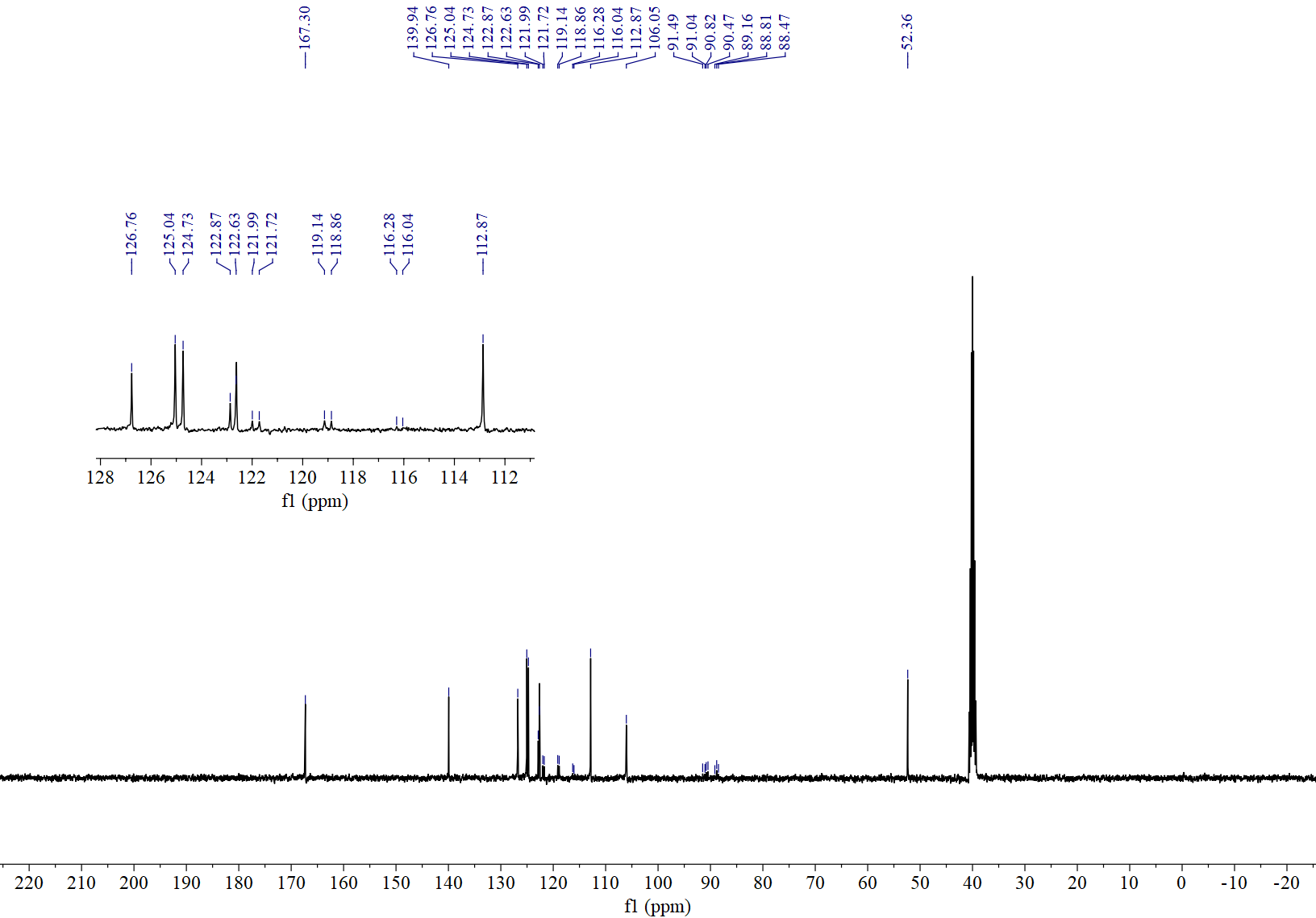

**^13^C NMR** of **14** (101 MHz, Dimethyl sulfoxide-*d_6_*, 298 K)


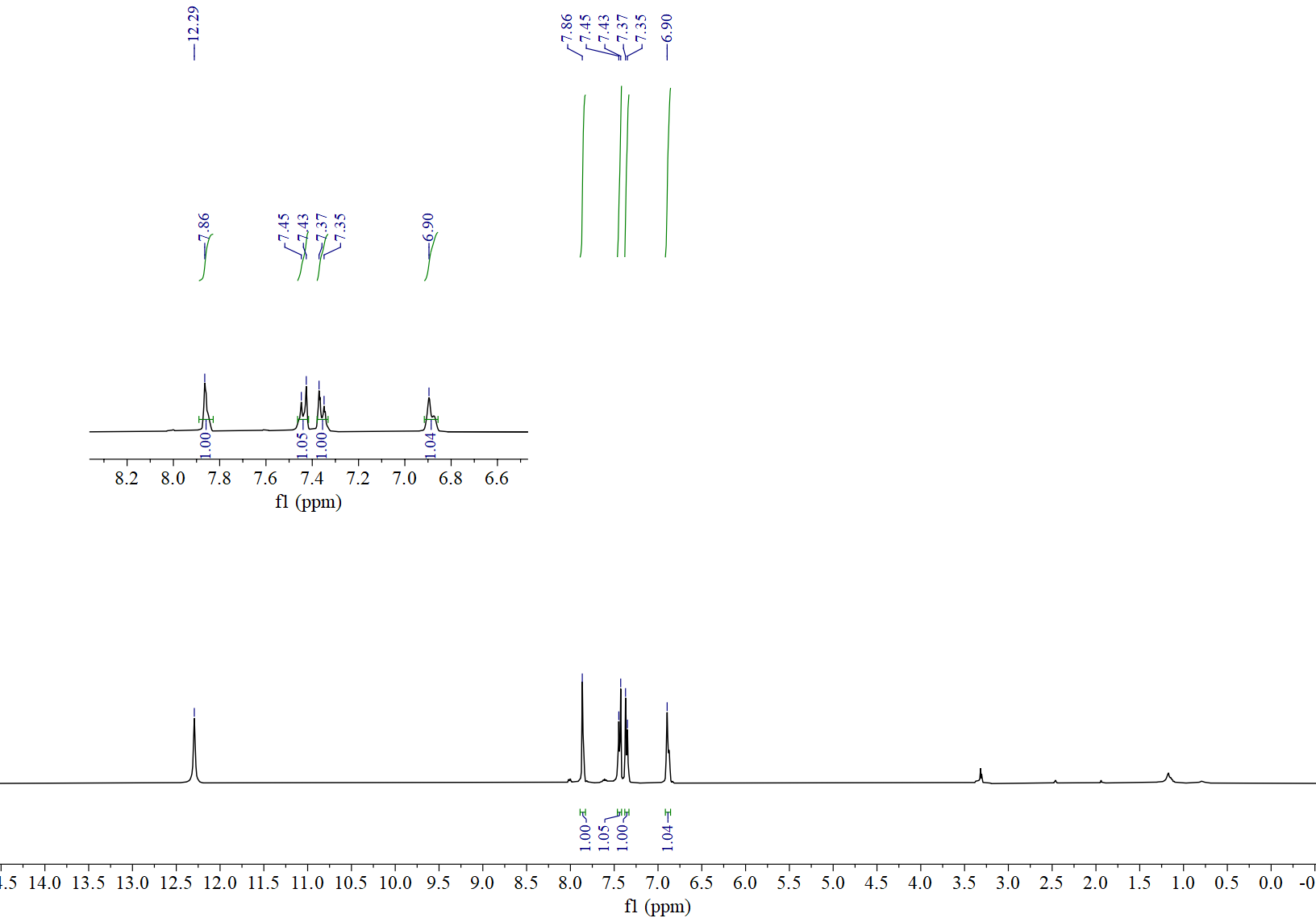

**^1^H NMR** of **15** (400 MHz, Dimethyl sulfoxide-*d_6_*, 298 K)


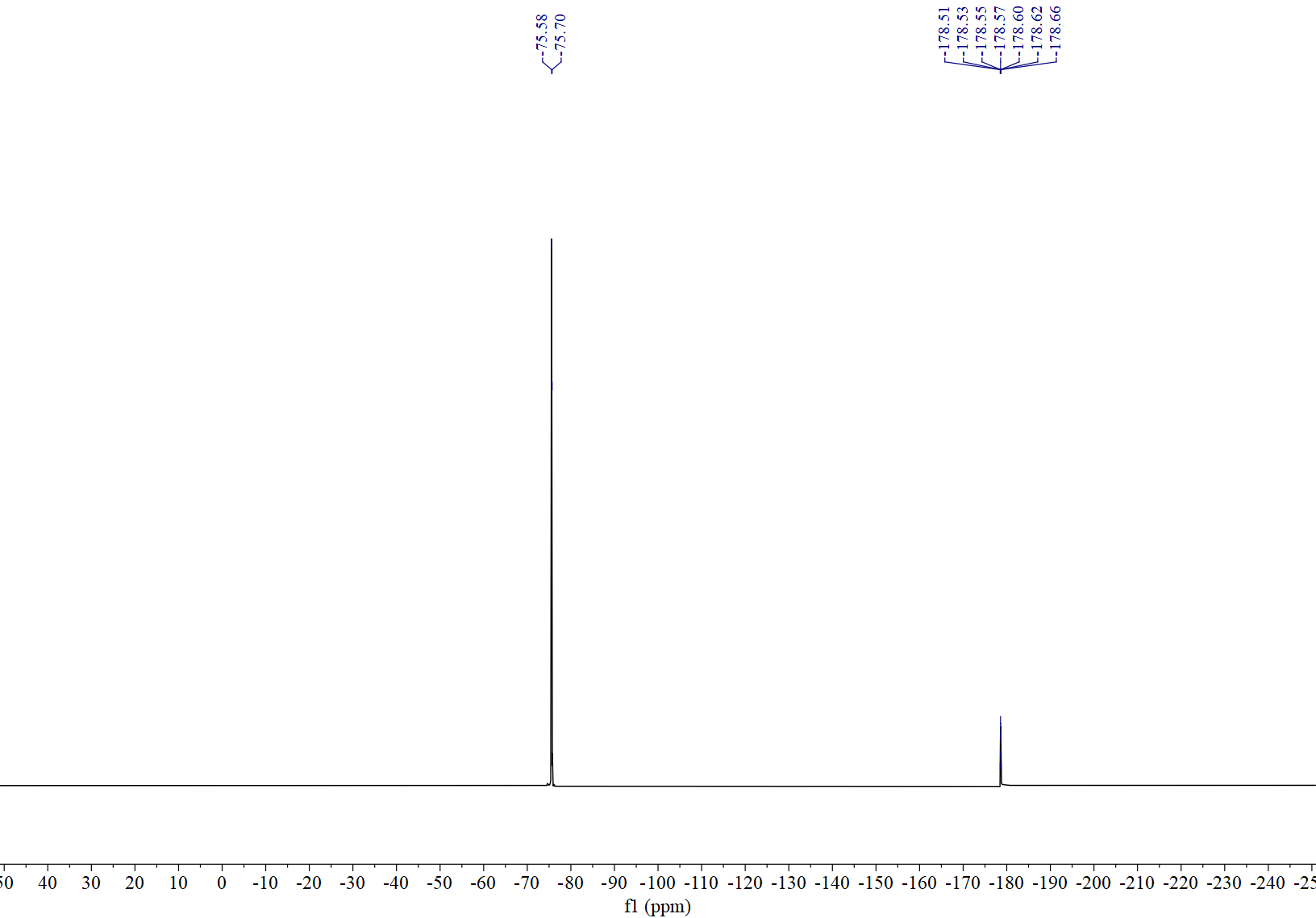

**^19^F NMR** of **15** (376 MHz, Dimethyl sulfoxide-*d_6_*, 298 K)


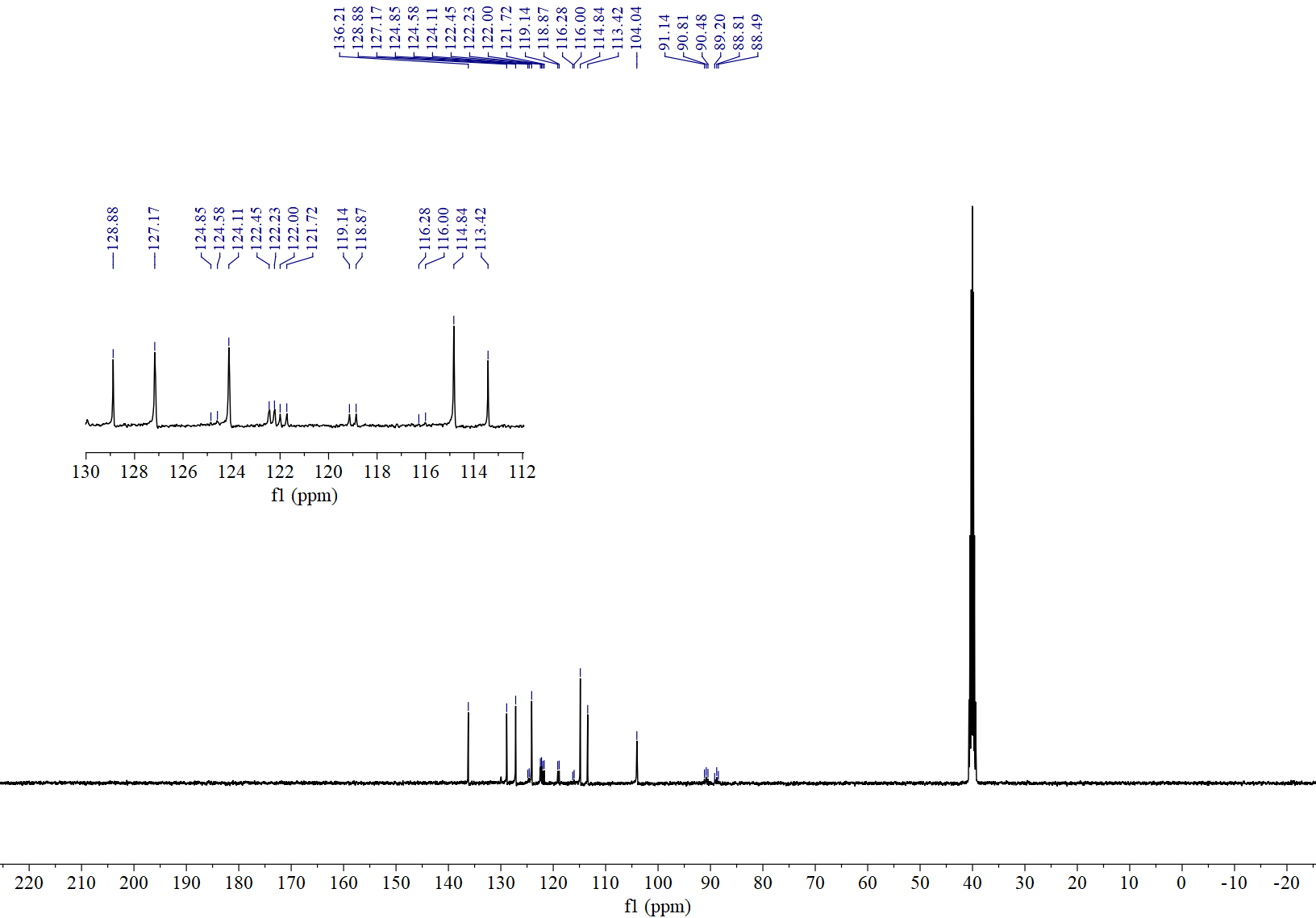

**^13^C NMR** of **15** (101 MHz, Dimethyl sulfoxide-*d_6_*, 298 K)


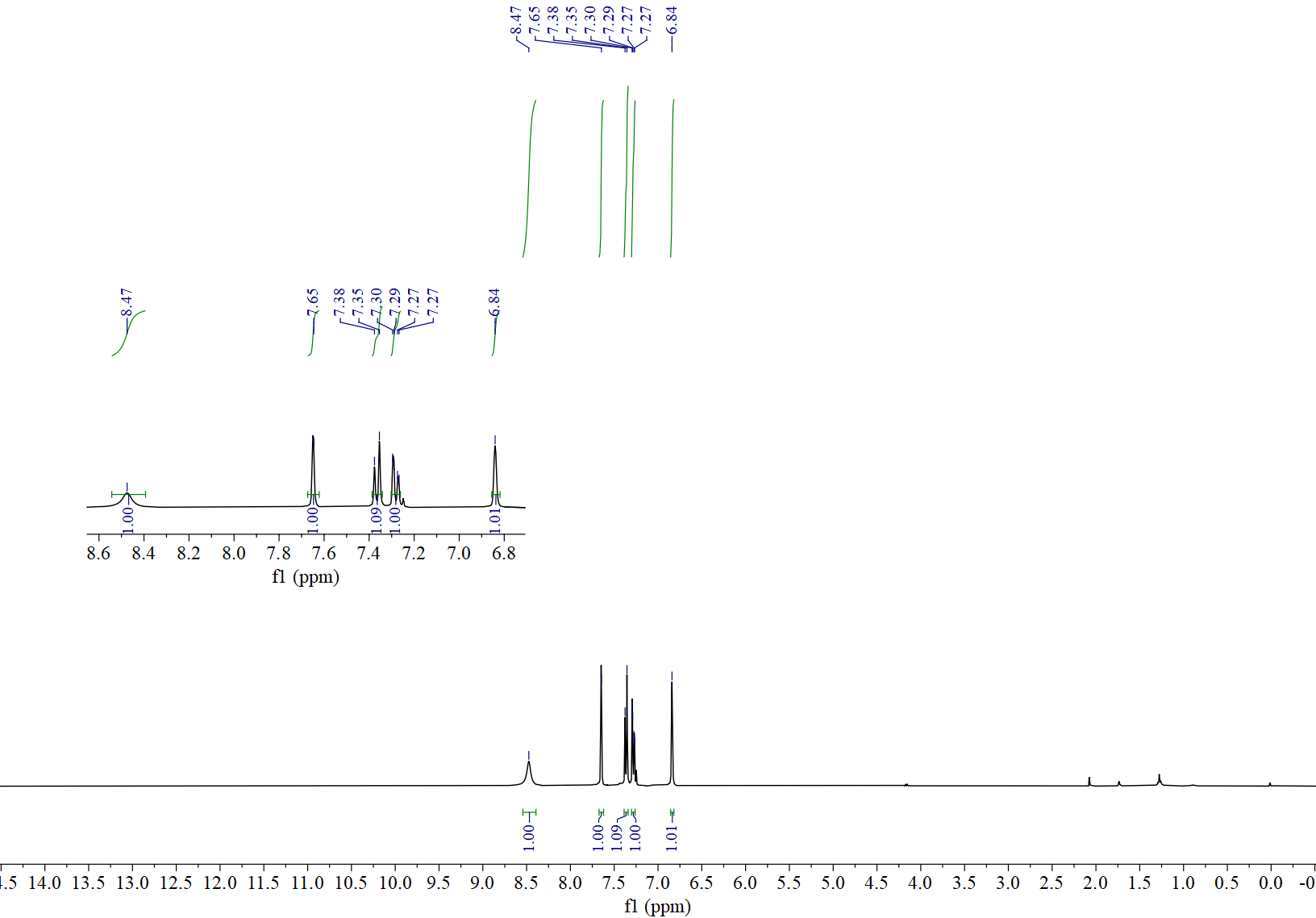

**^1^H NMR** of **16** (400 MHz, Chloroform-*d*, 298 K)


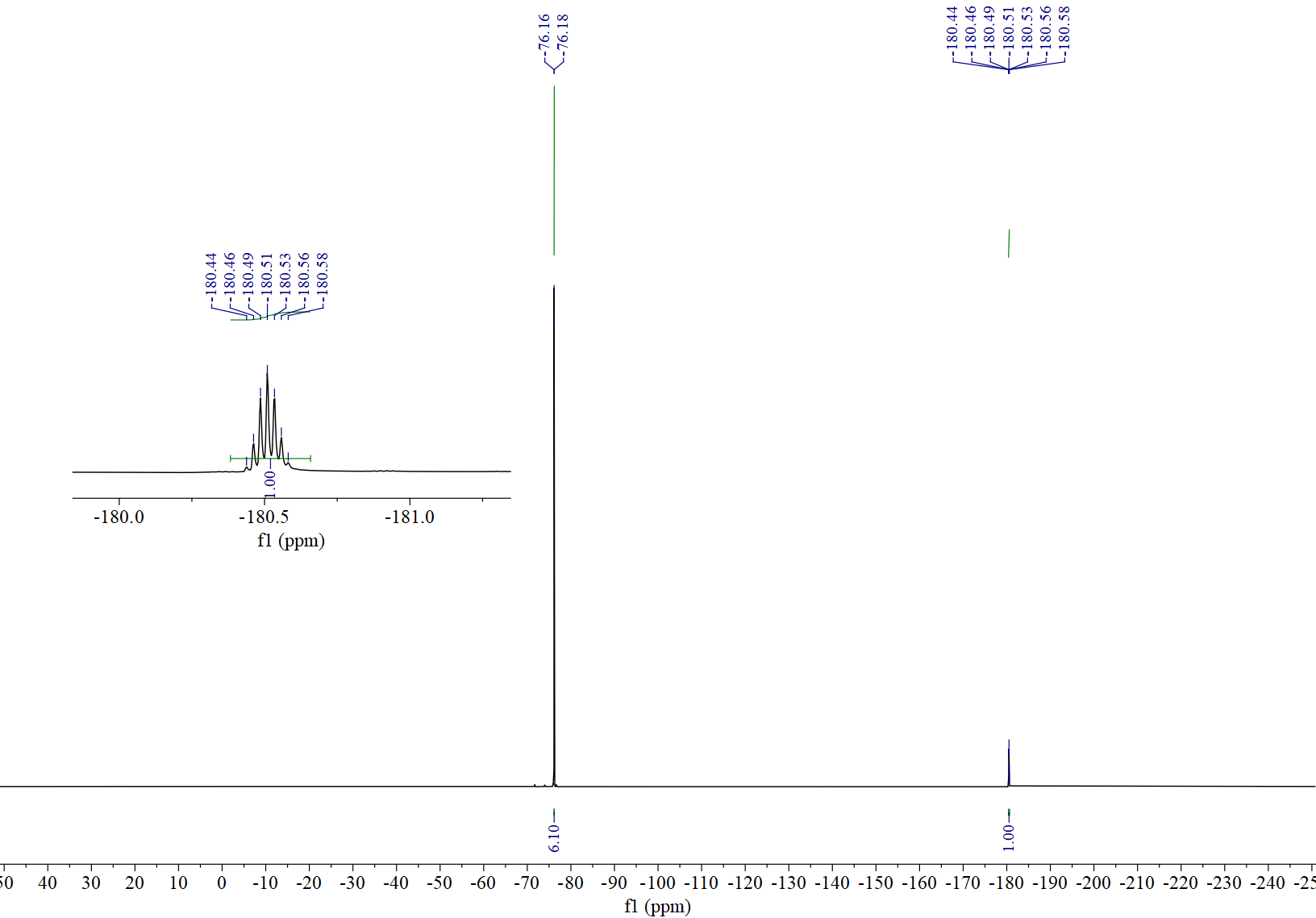

**^19^F NMR** of **16** (376 MHz, Chloroform-*d*, 298 K)


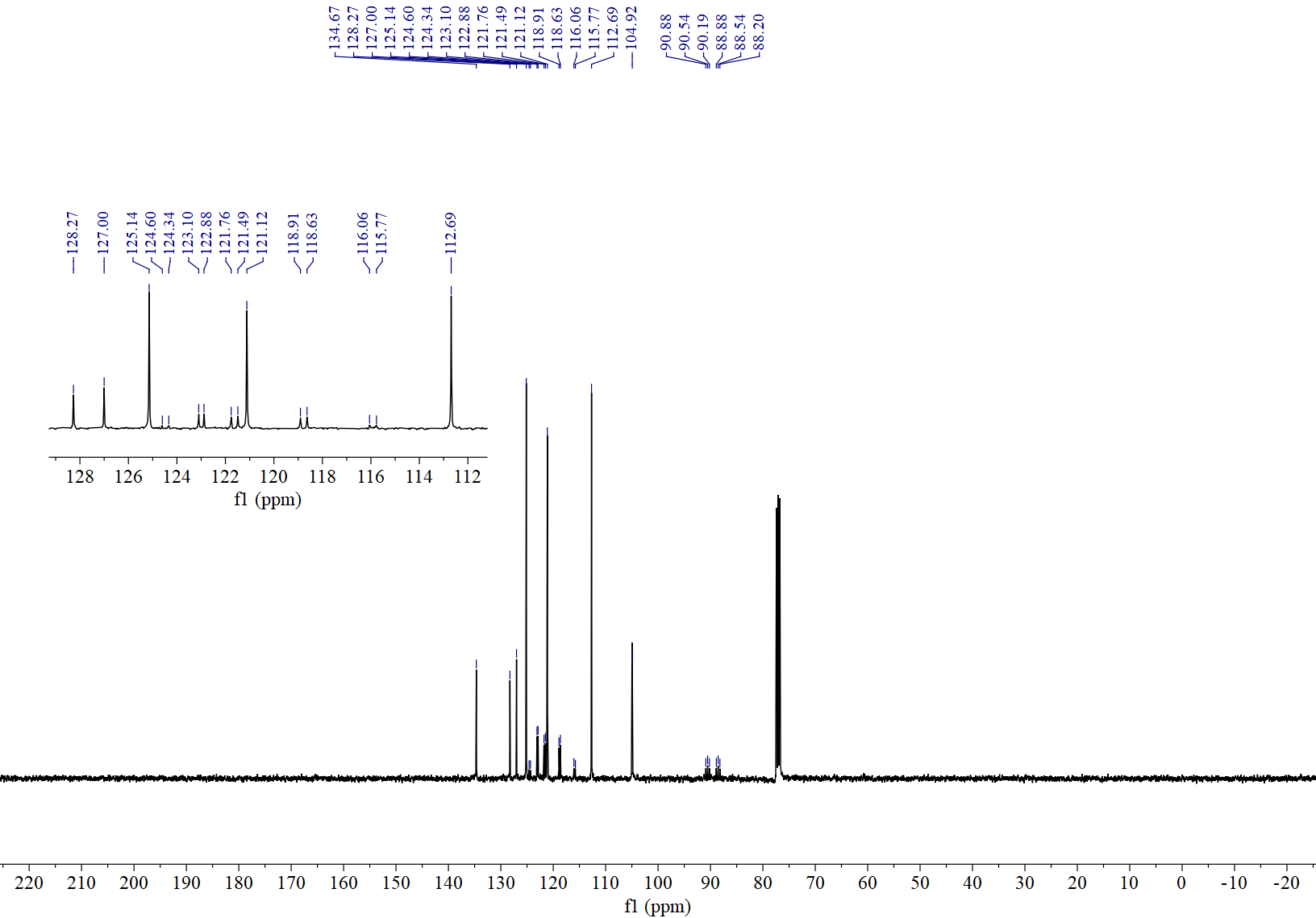

**^13^C NMR** of **16** (101 MHz, Chloroform-*d*, 298 K)


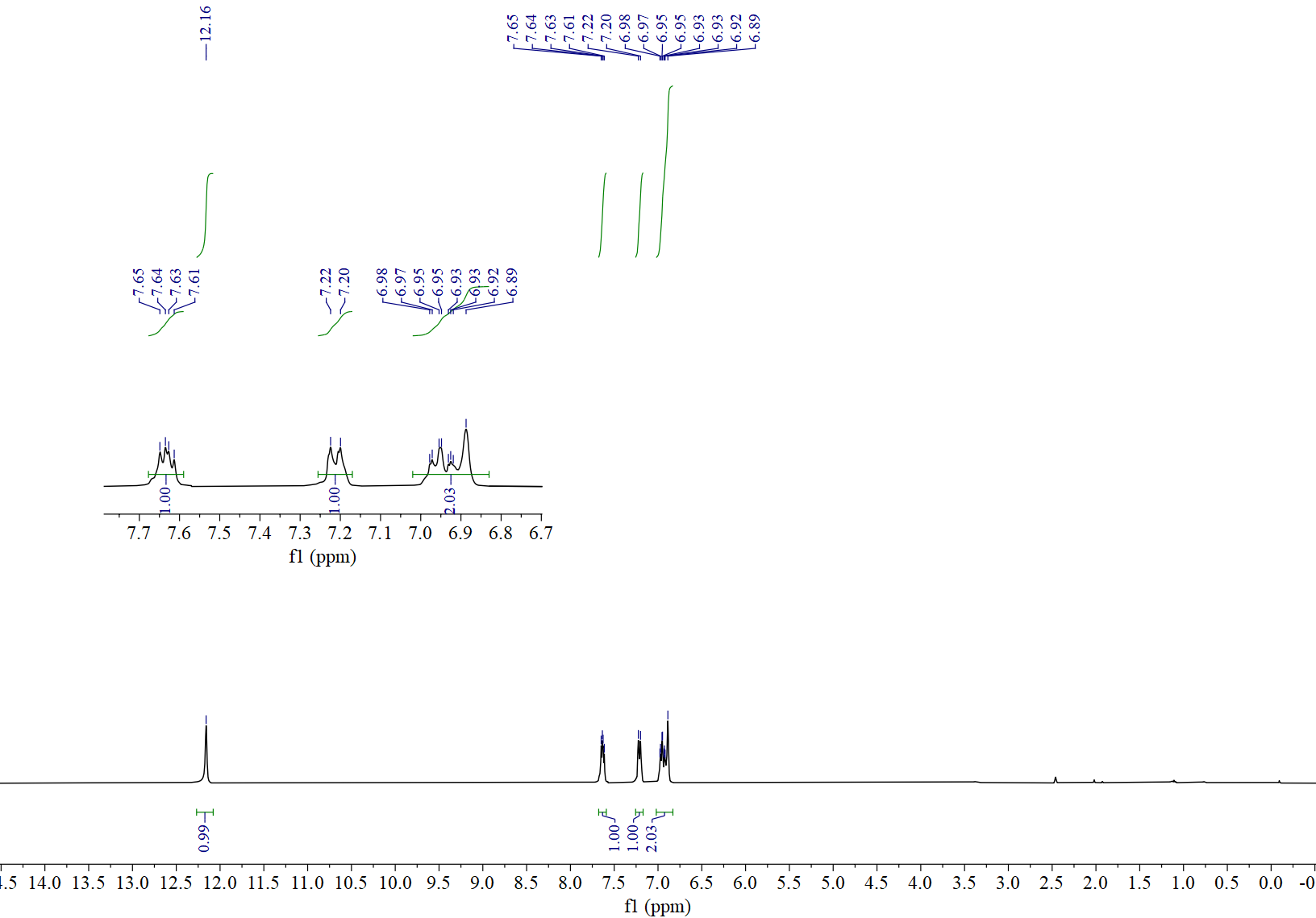

**^1^H NMR** of **17** (400 MHz, Dimethyl sulfoxide-*d_6_*, 298 K)


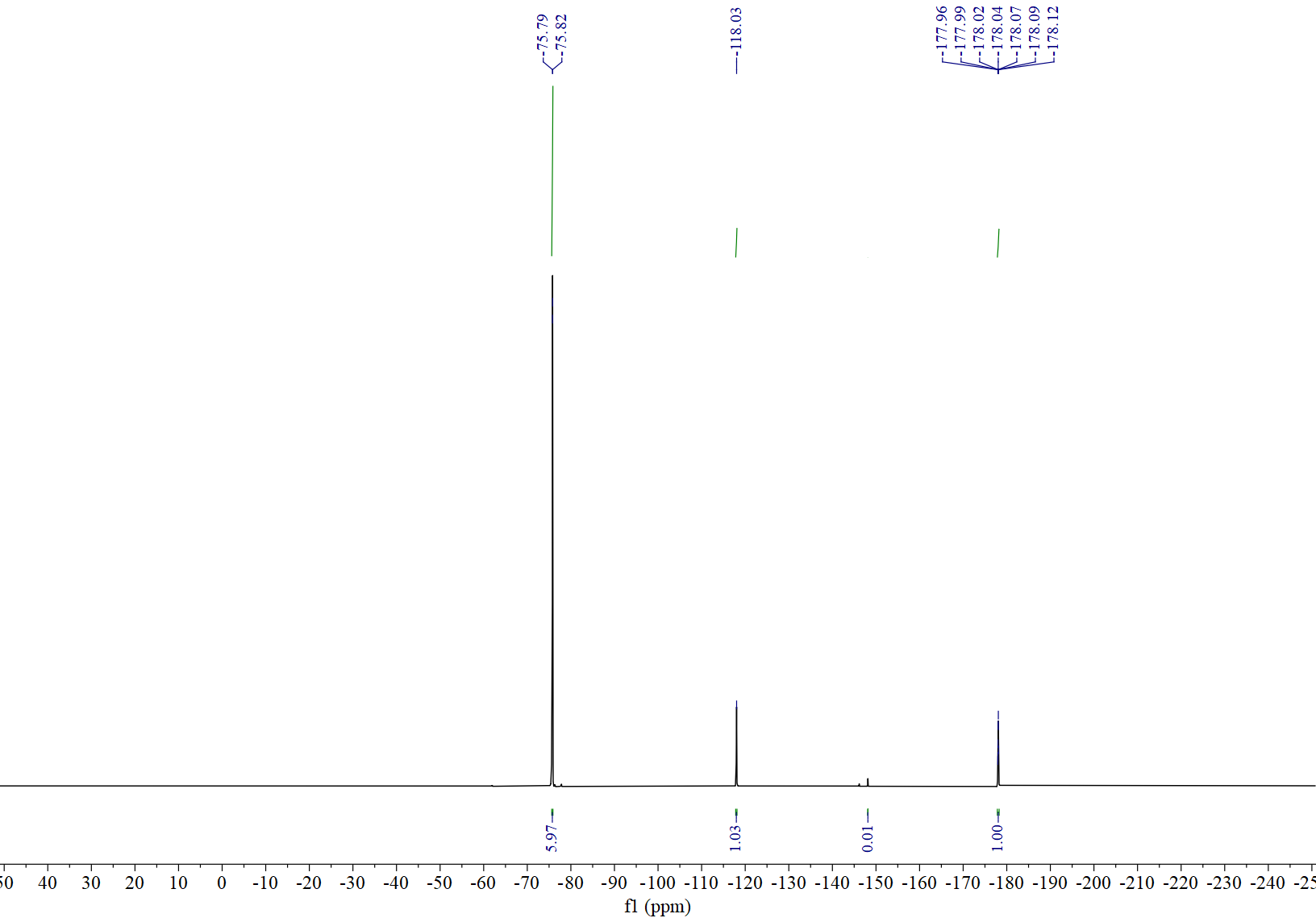

**^19^F NMR** of **17** (376 MHz, Dimethyl sulfoxide-*d_6_*, 298 K)


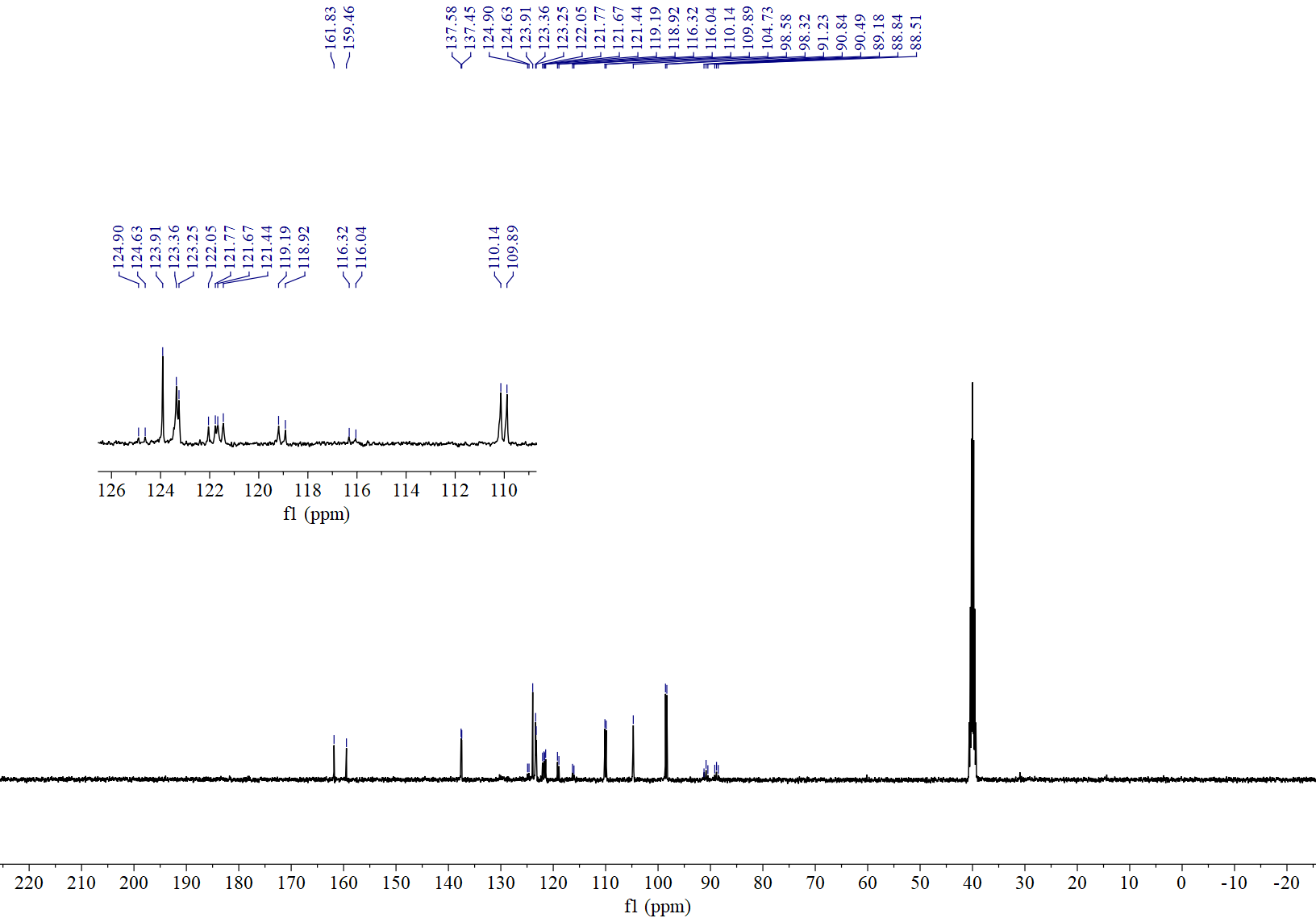

**^13^C NMR** of **17** (101 MHz, Dimethyl sulfoxide-*d_6_*, 298 K)


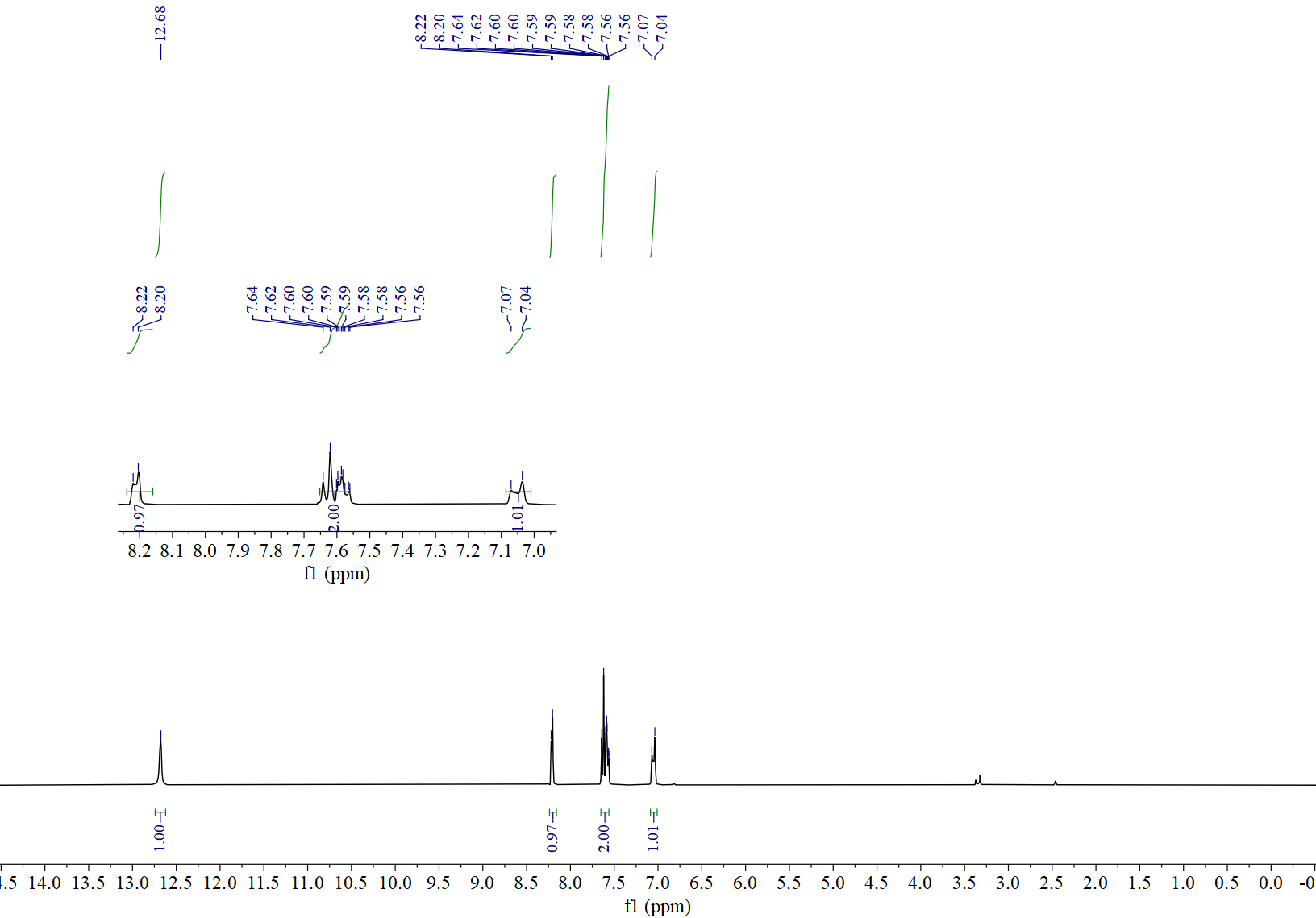

**^1^H NMR** of **18** (400 MHz, Dimethyl sulfoxide-*d_6_*, 298 K)


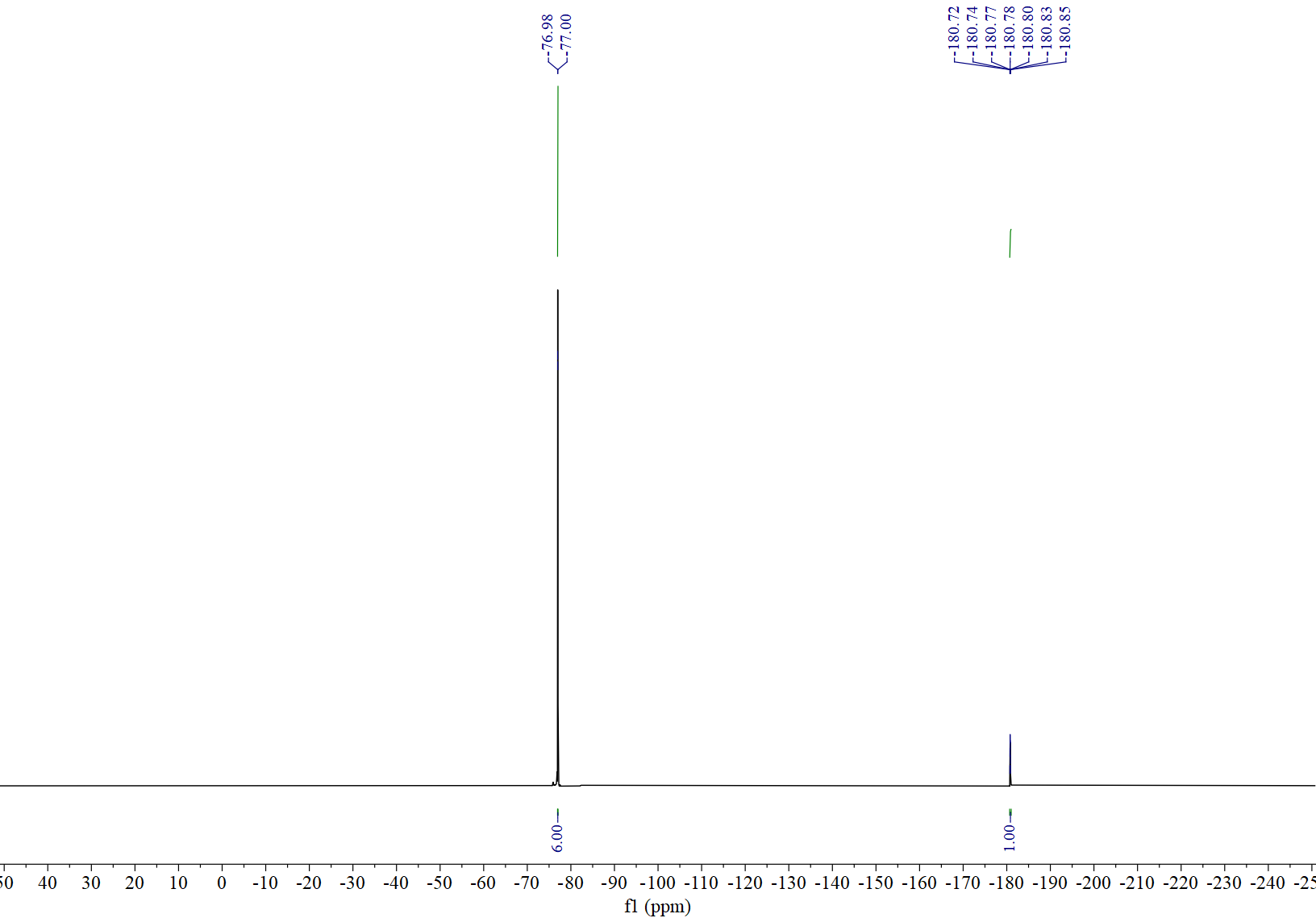

**^19^F NMR** of **18** (376 MHz, Dimethyl sulfoxide-*d_6_*, 298 K)


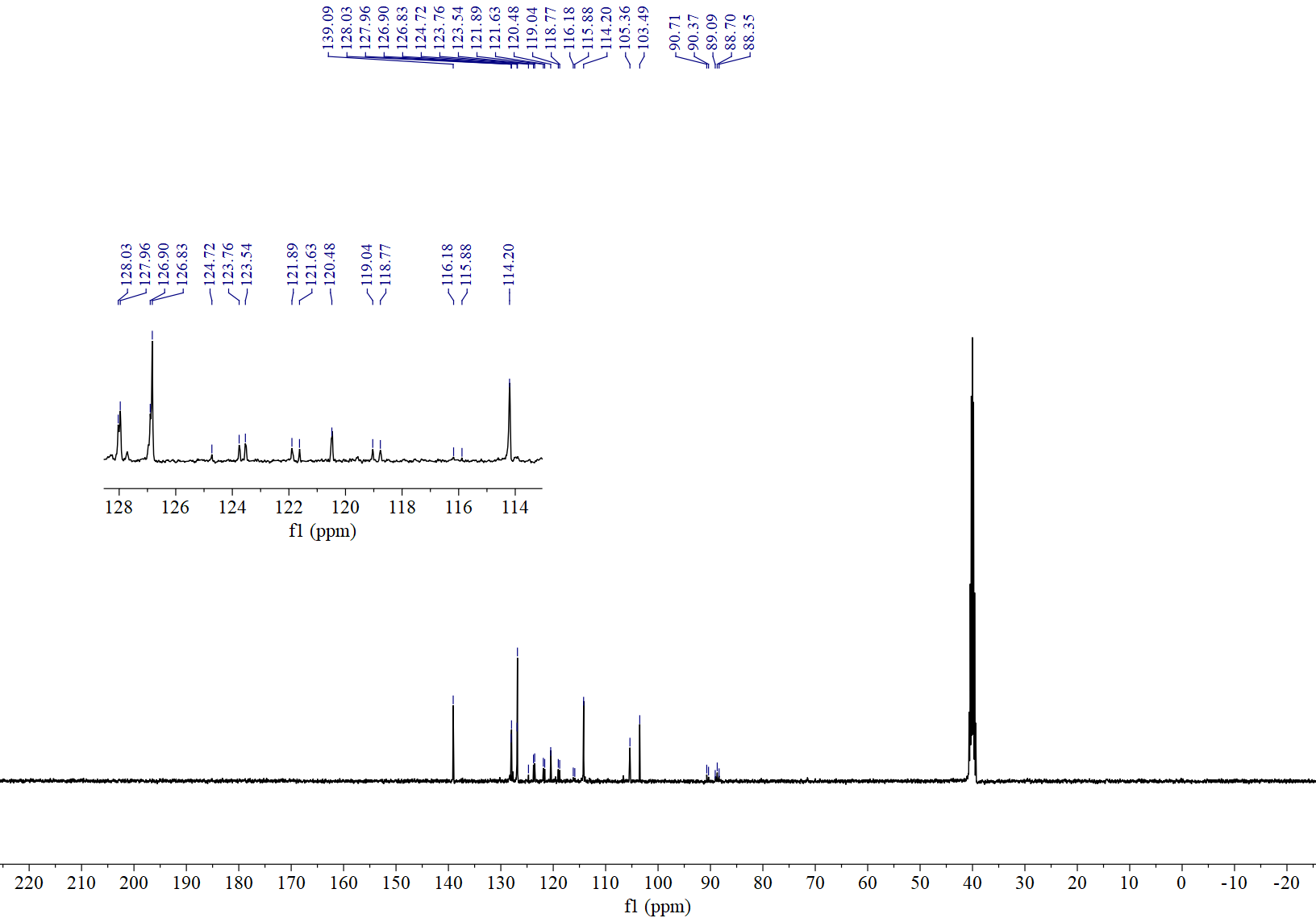

**^13^C NMR** of **18** (101 MHz, Dimethyl sulfoxide-*d_6_*, 298 K)


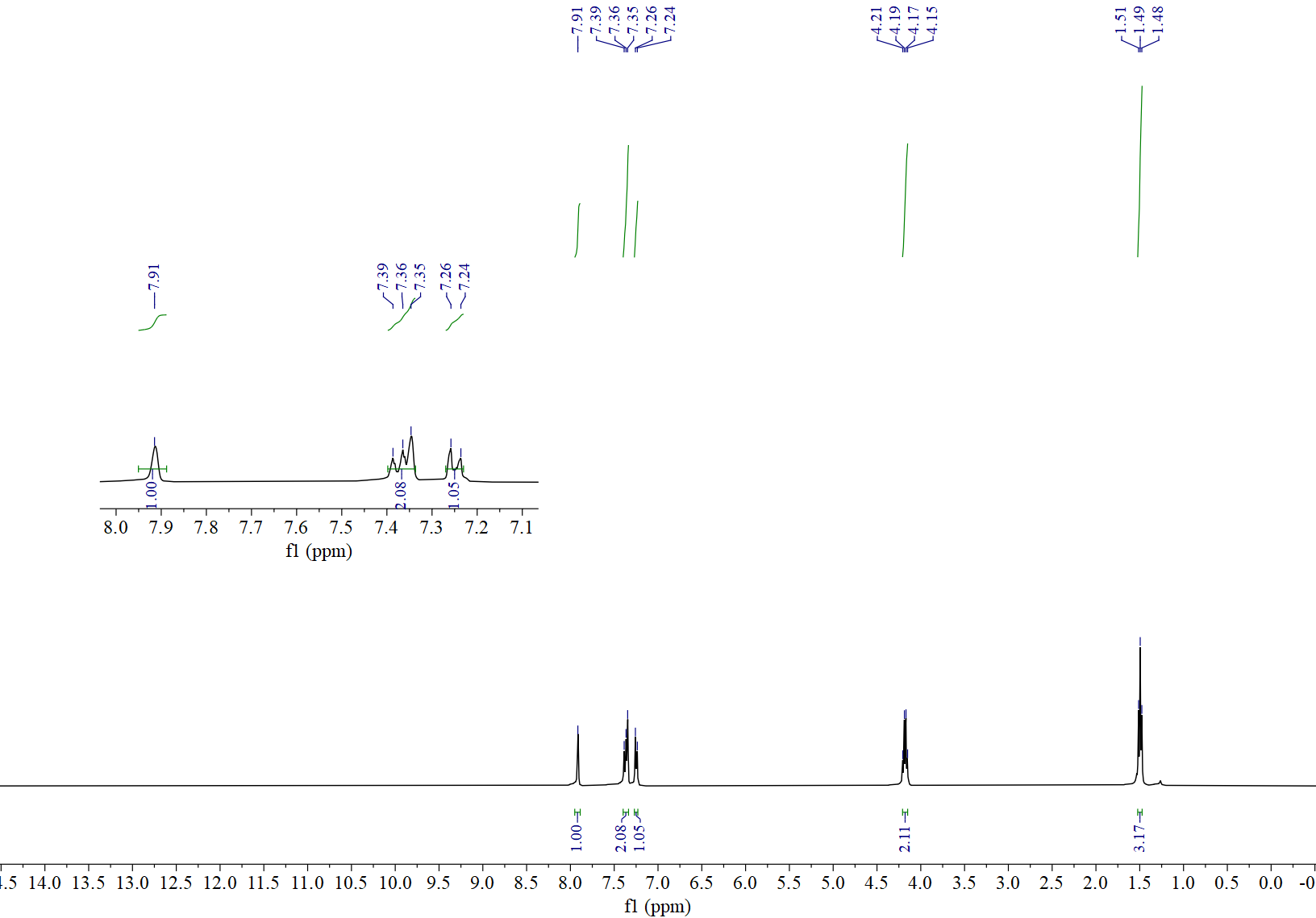

**^1^H NMR** of **19** (400 MHz, Chloroform-*d*, 298 K)


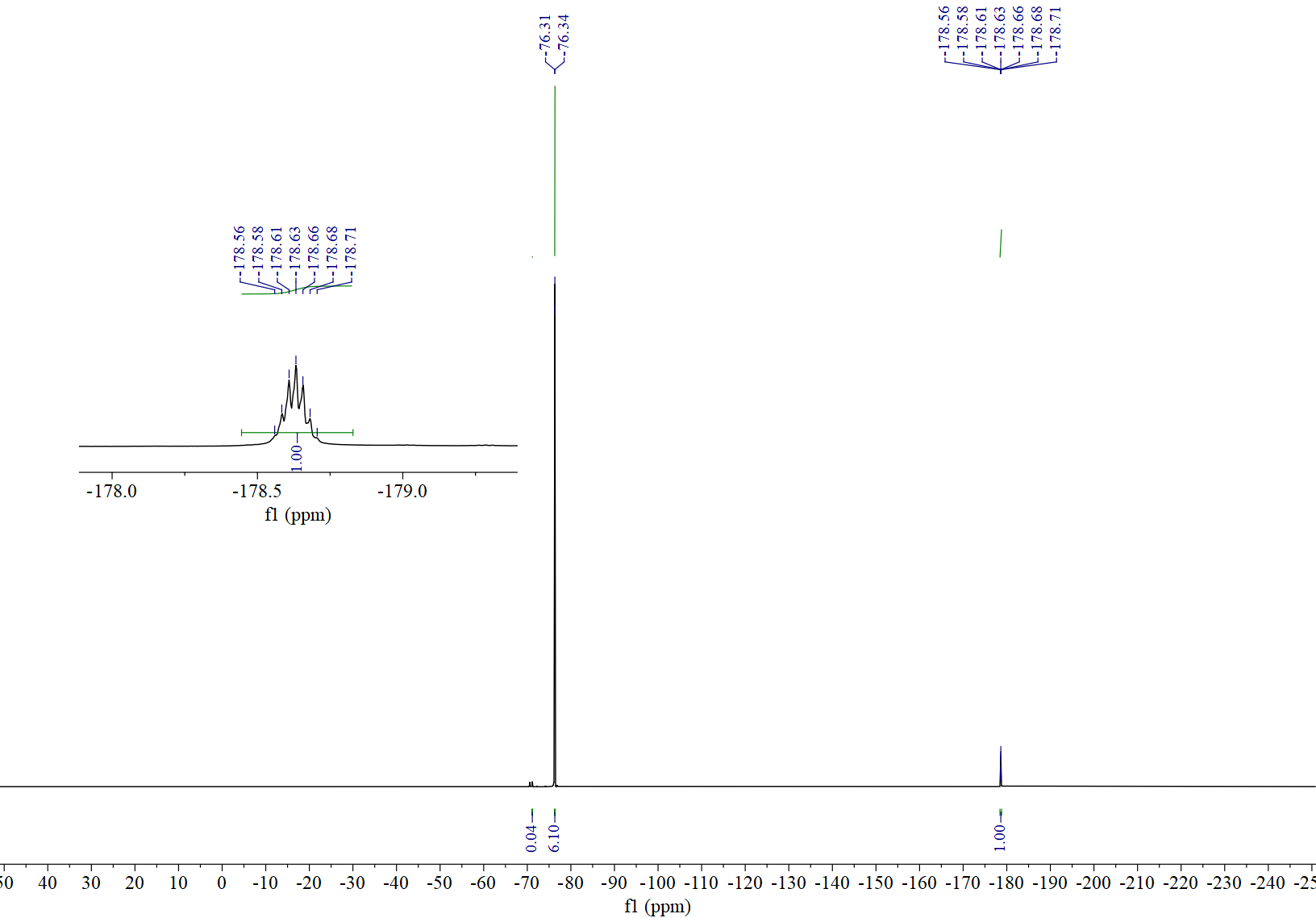

**^19^F NMR** of **19** (376 MHz, Chloroform-*d*, 298 K)


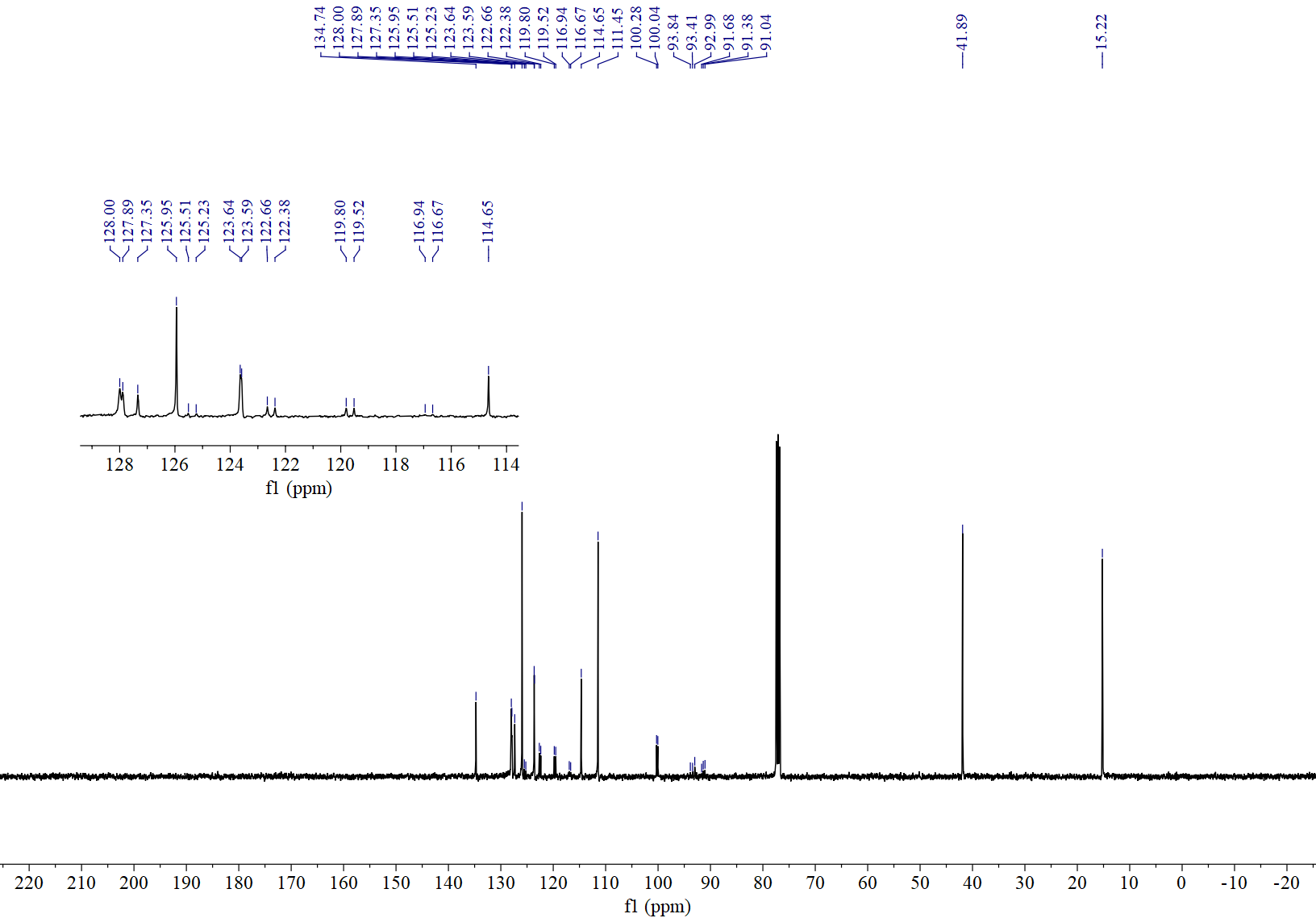

**^13^C NMR** of **19** (101 MHz, Chloroform-*d*, 298 K)


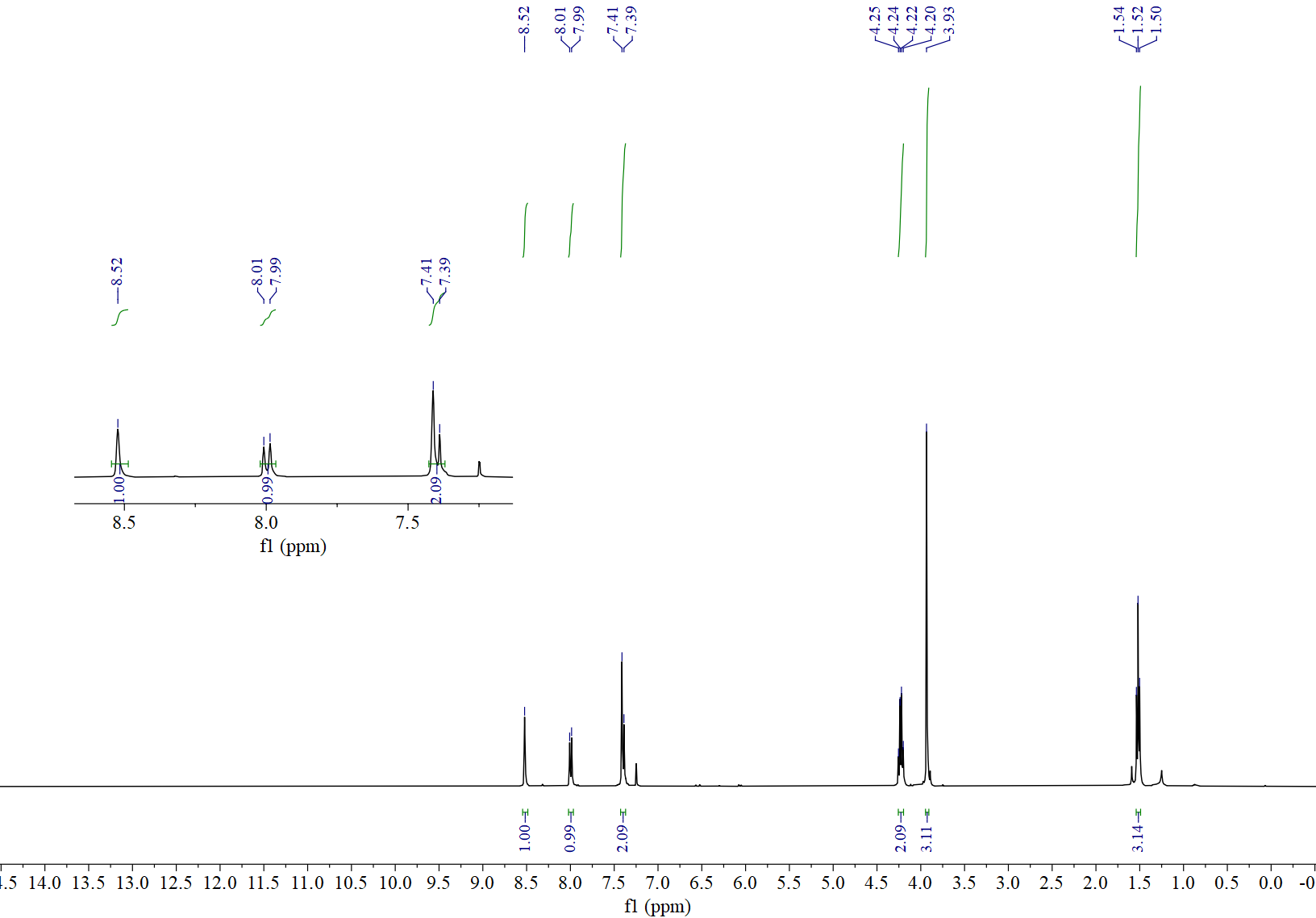

**^1^H NMR** of **20** (400 MHz, Chloroform-*d*, 298 K)


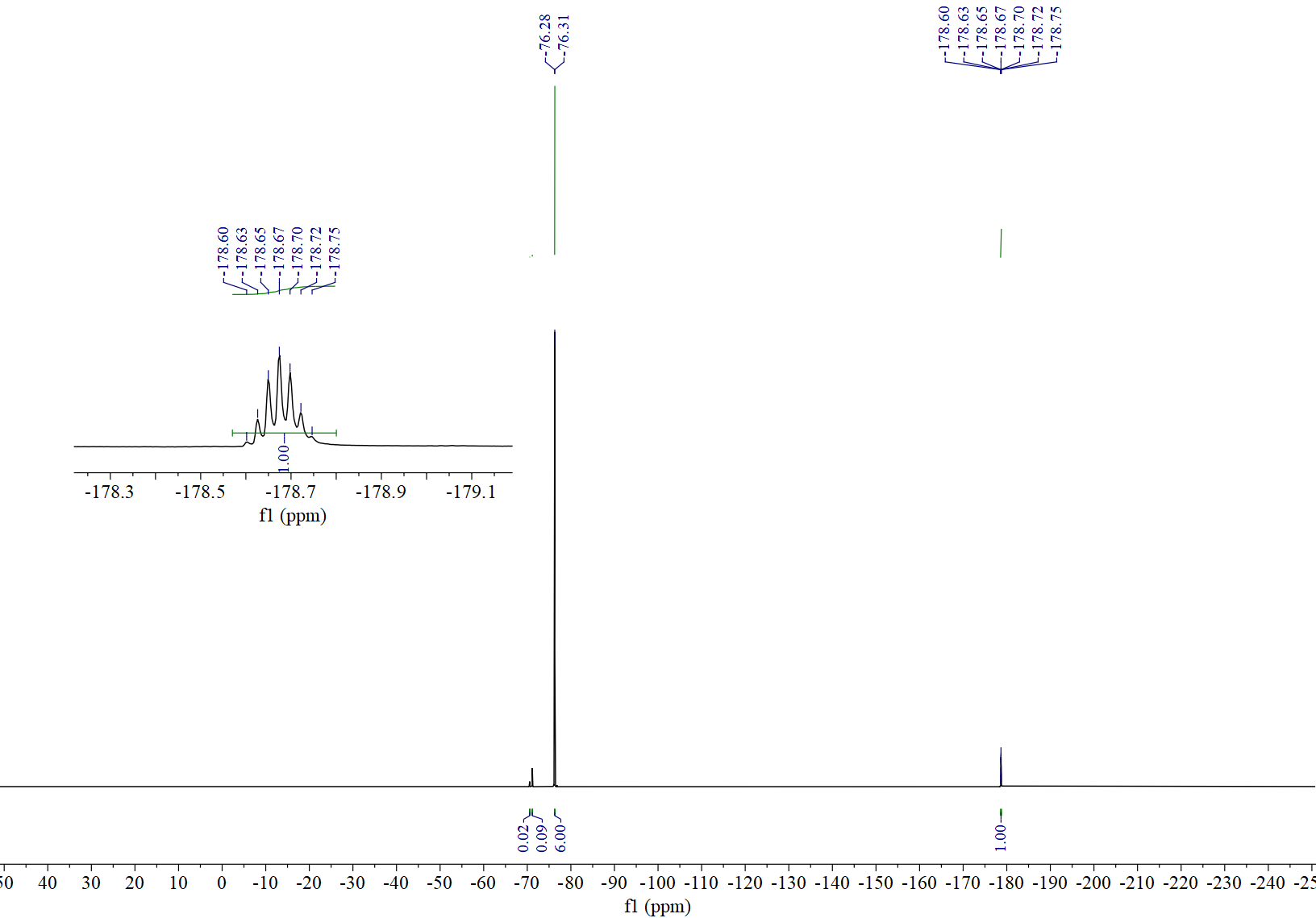

**^19^F NMR** of **20** (376 MHz, Chloroform-*d*, 298 K)


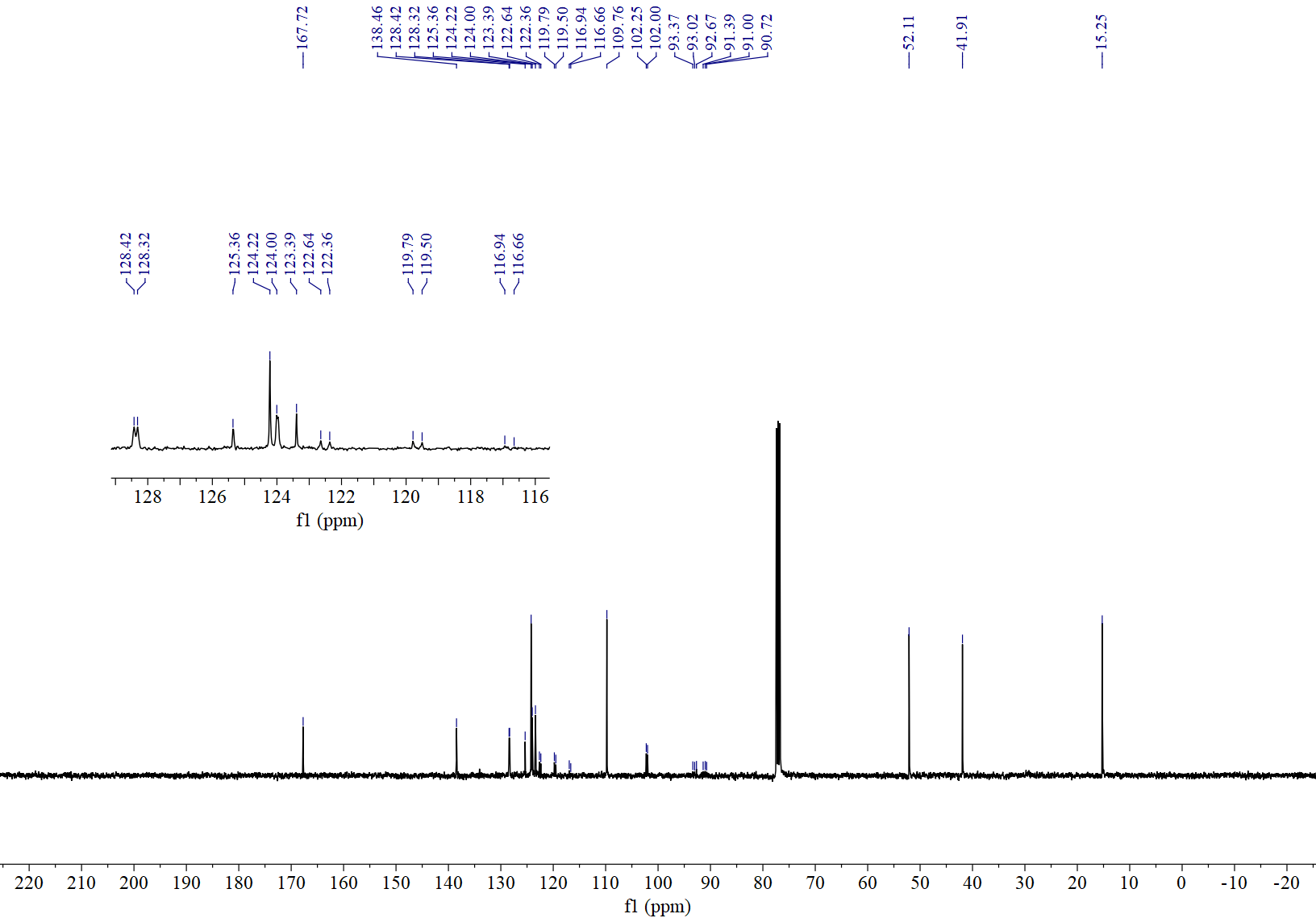

**^13^C NMR** of **20** (101 MHz, Chloroform-*d*, 298 K)


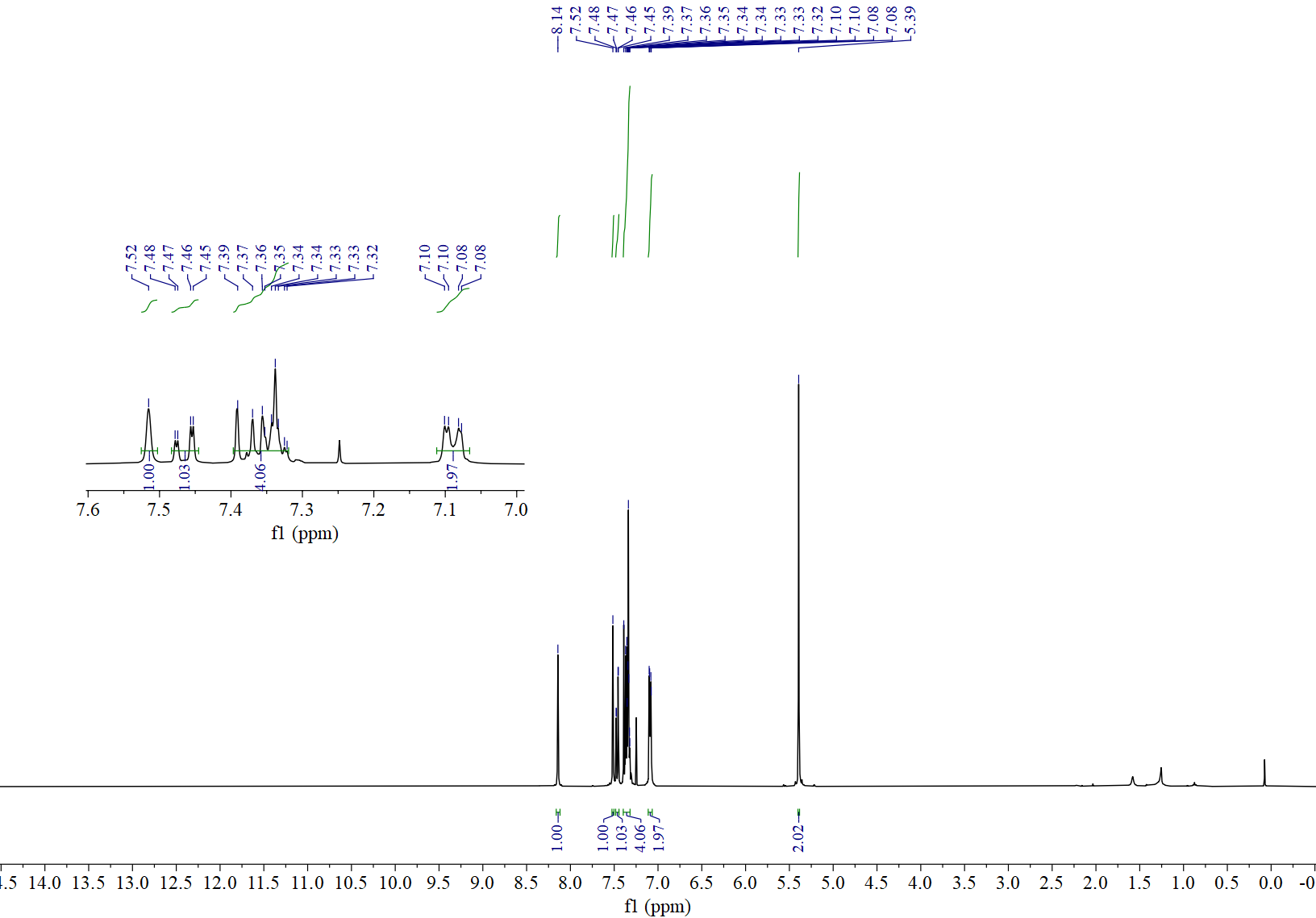

**^1^H NMR** of **21** (400 MHz, Chloroform-*d*, 298 K)


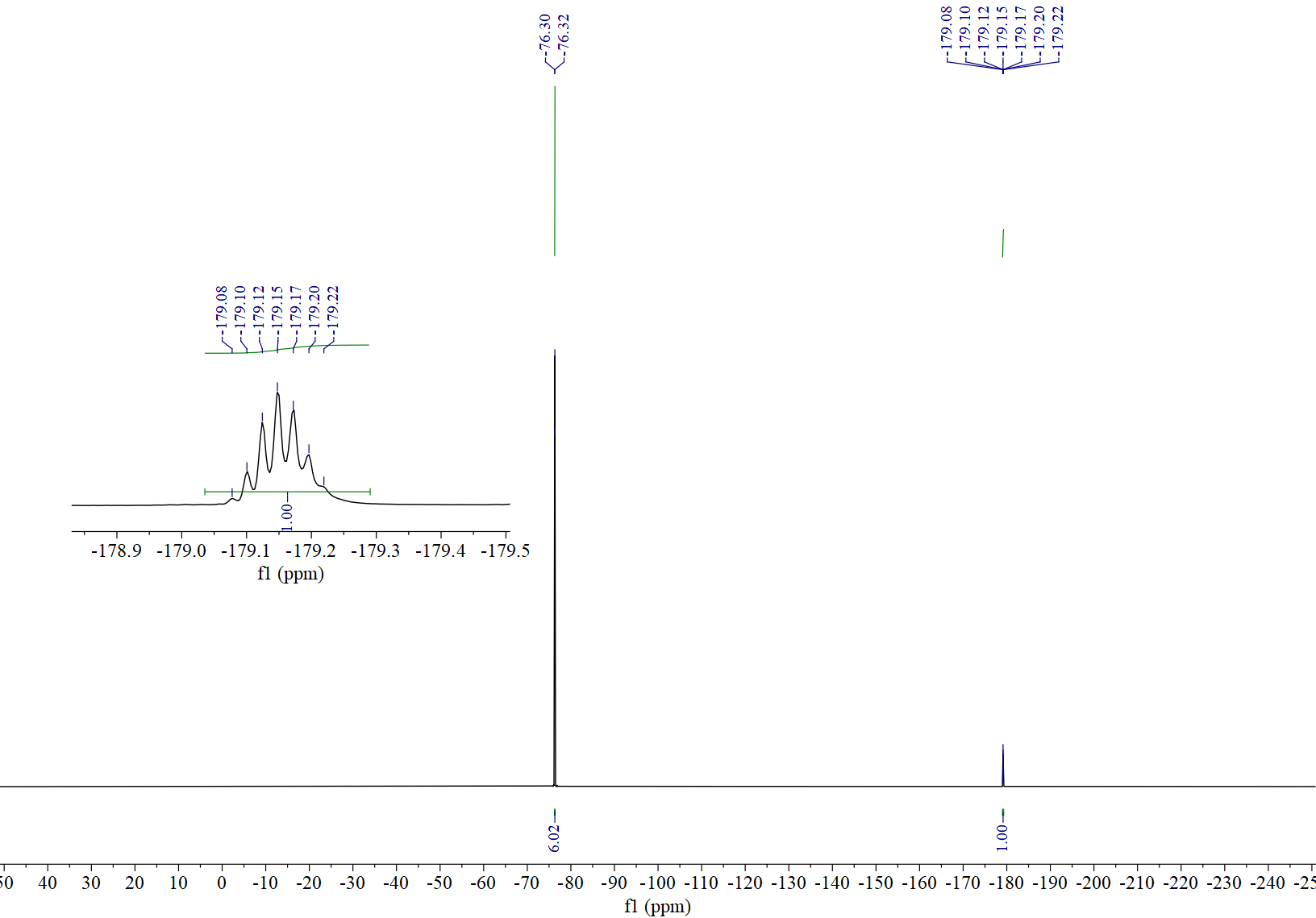

**^19^F NMR** of **21** (376 MHz, Chloroform-*d*, 298 K)


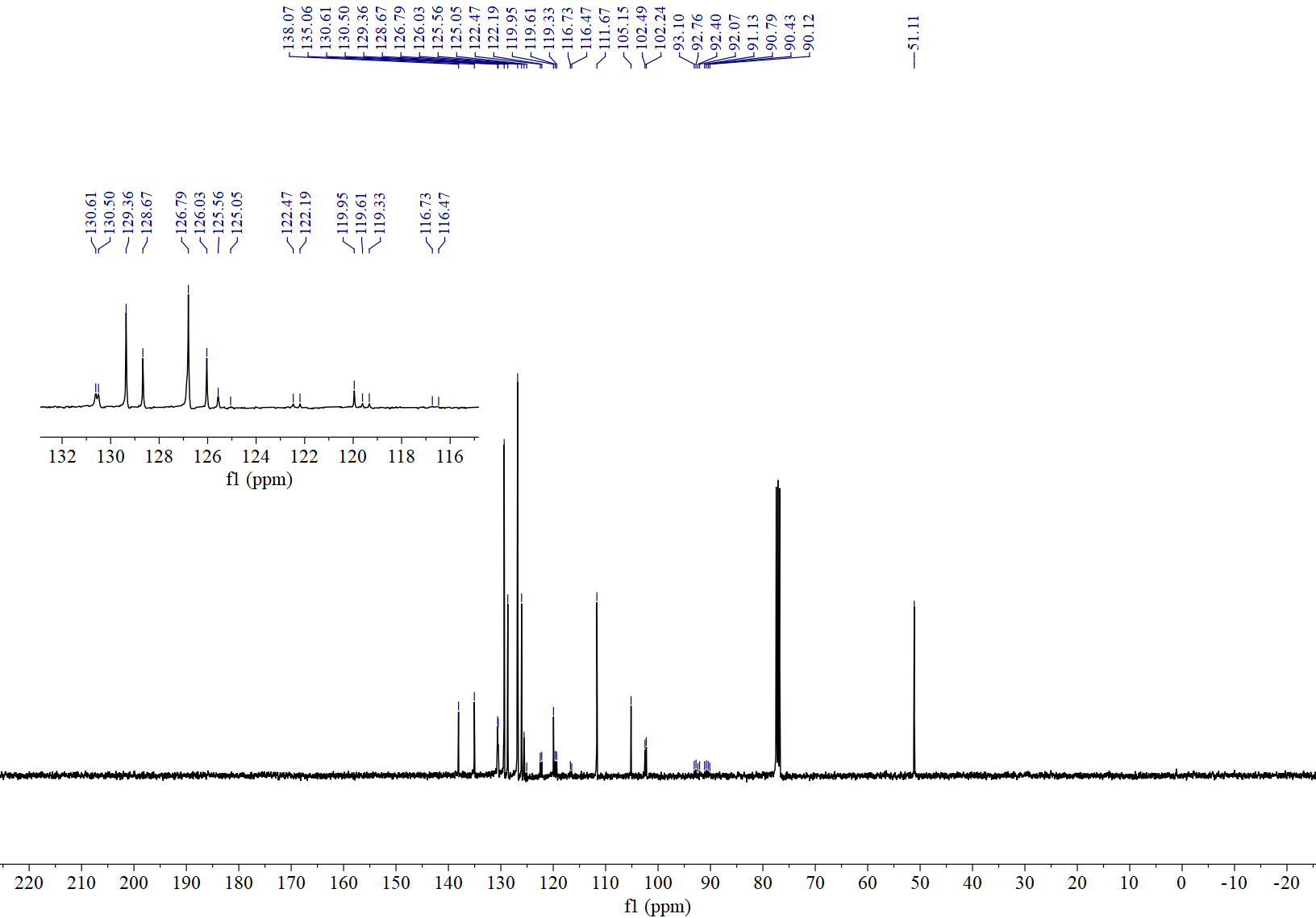

**^13^C NMR** of **21** (101 MHz, Chloroform-*d*, 298 K)


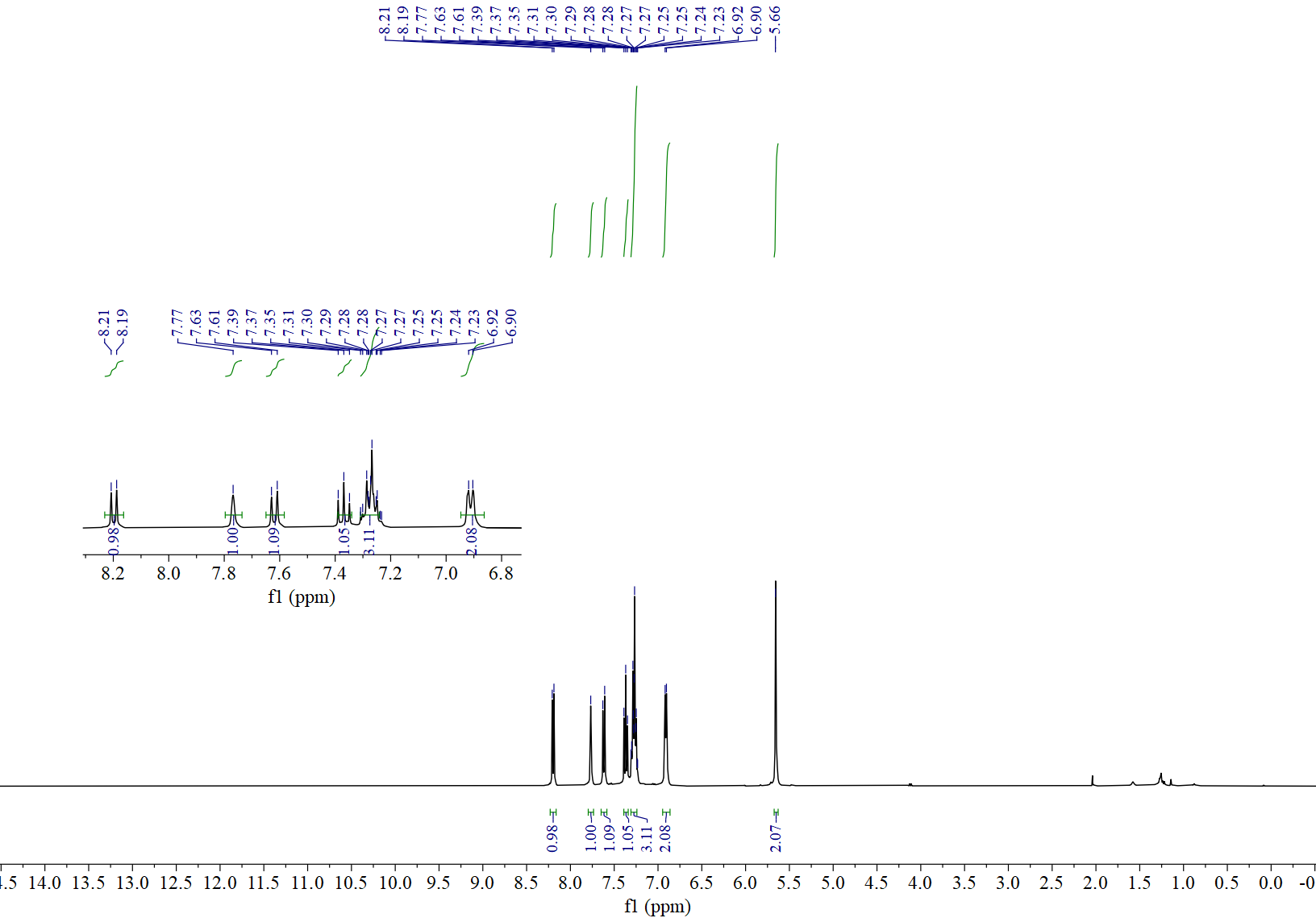

**^1^H NMR** of **22** (400 MHz, Chloroform-*d*, 298 K)


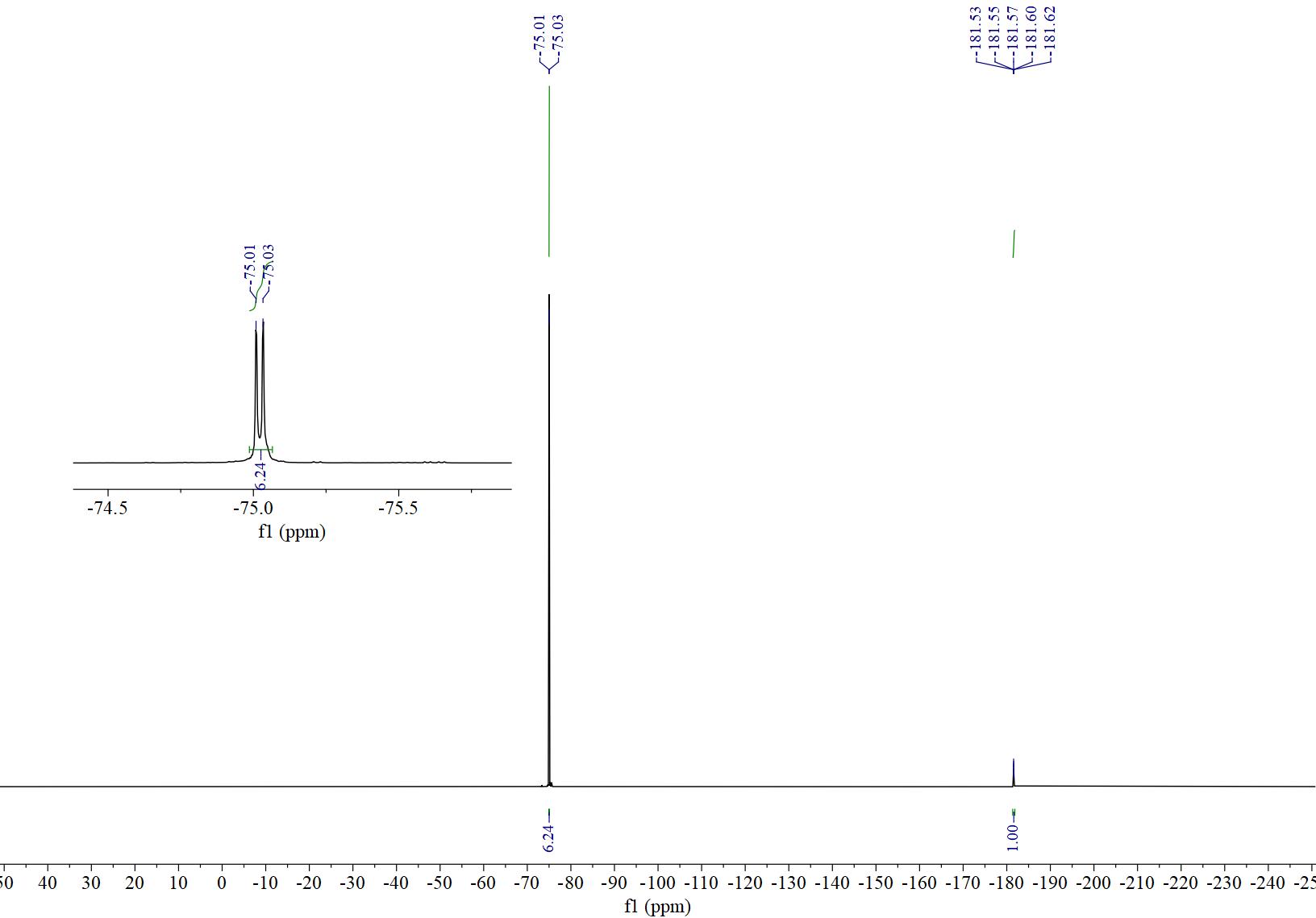

**^19^F NMR** of **22** (376 MHz, Chloroform-*d*, 298 K)


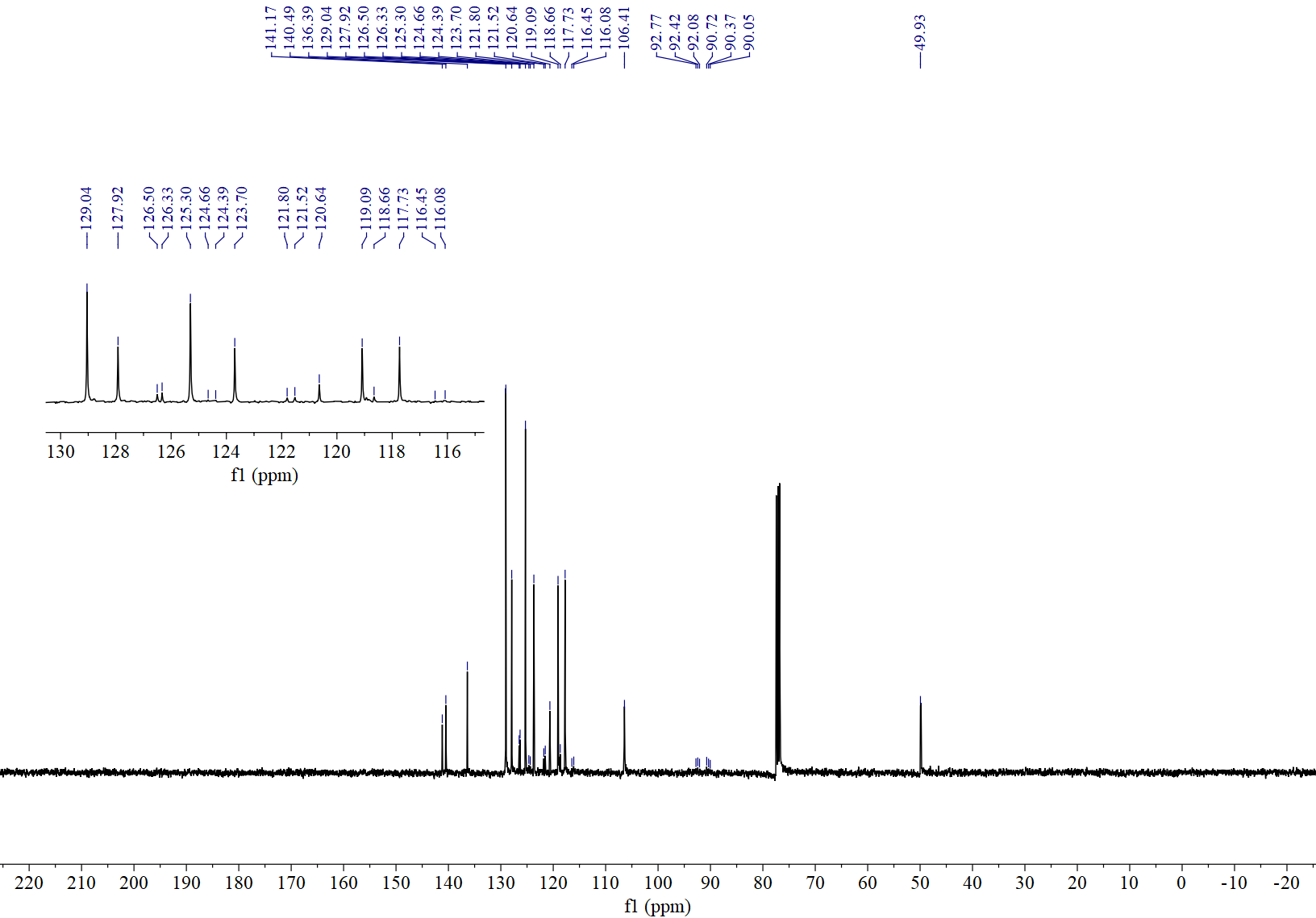

**^13^C NMR** of **22** (101 MHz, Chloroform-*d*, 298 K)


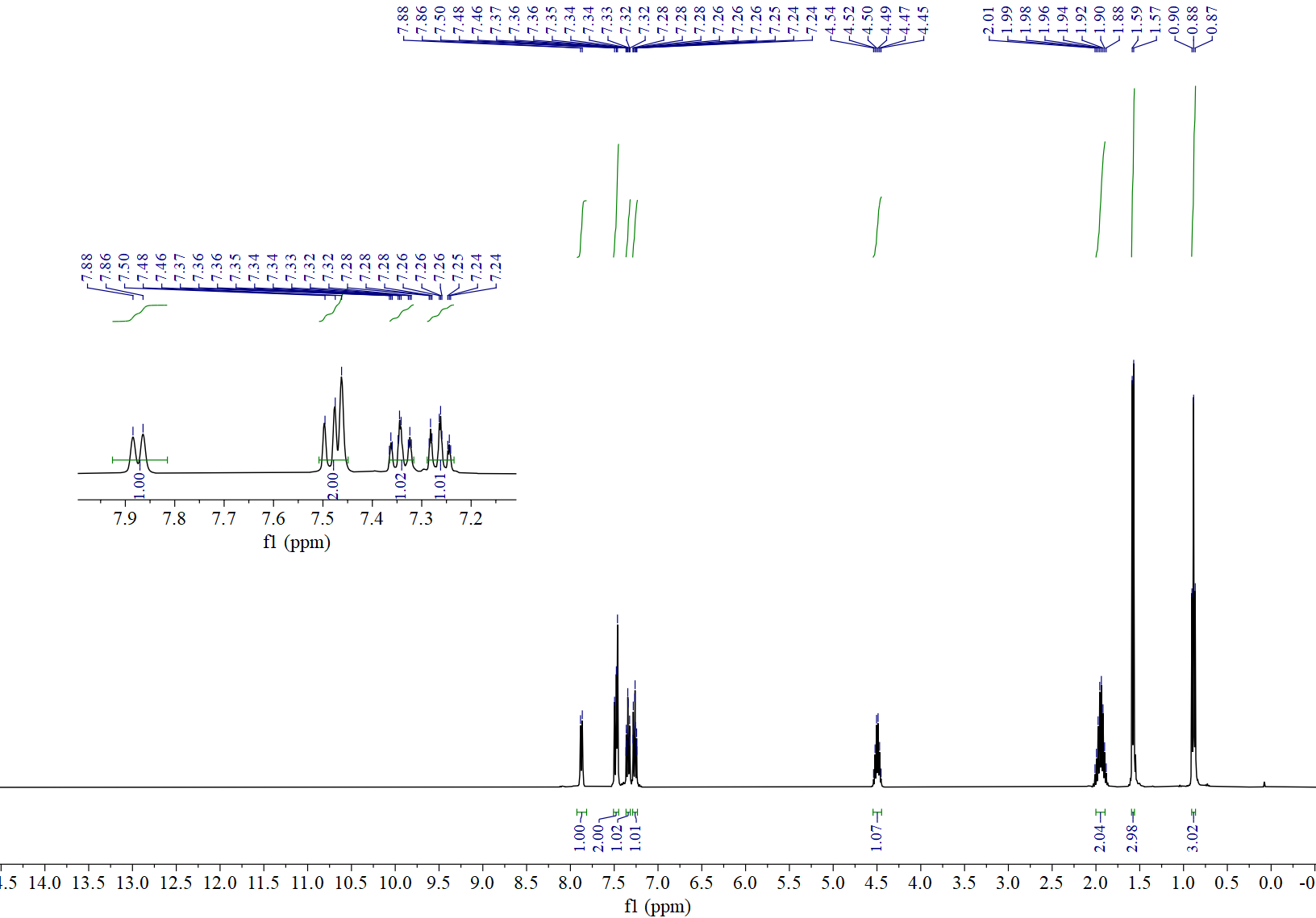

**^1^H NMR** of **23** (400 MHz, Chloroform-*d*, 298 K)


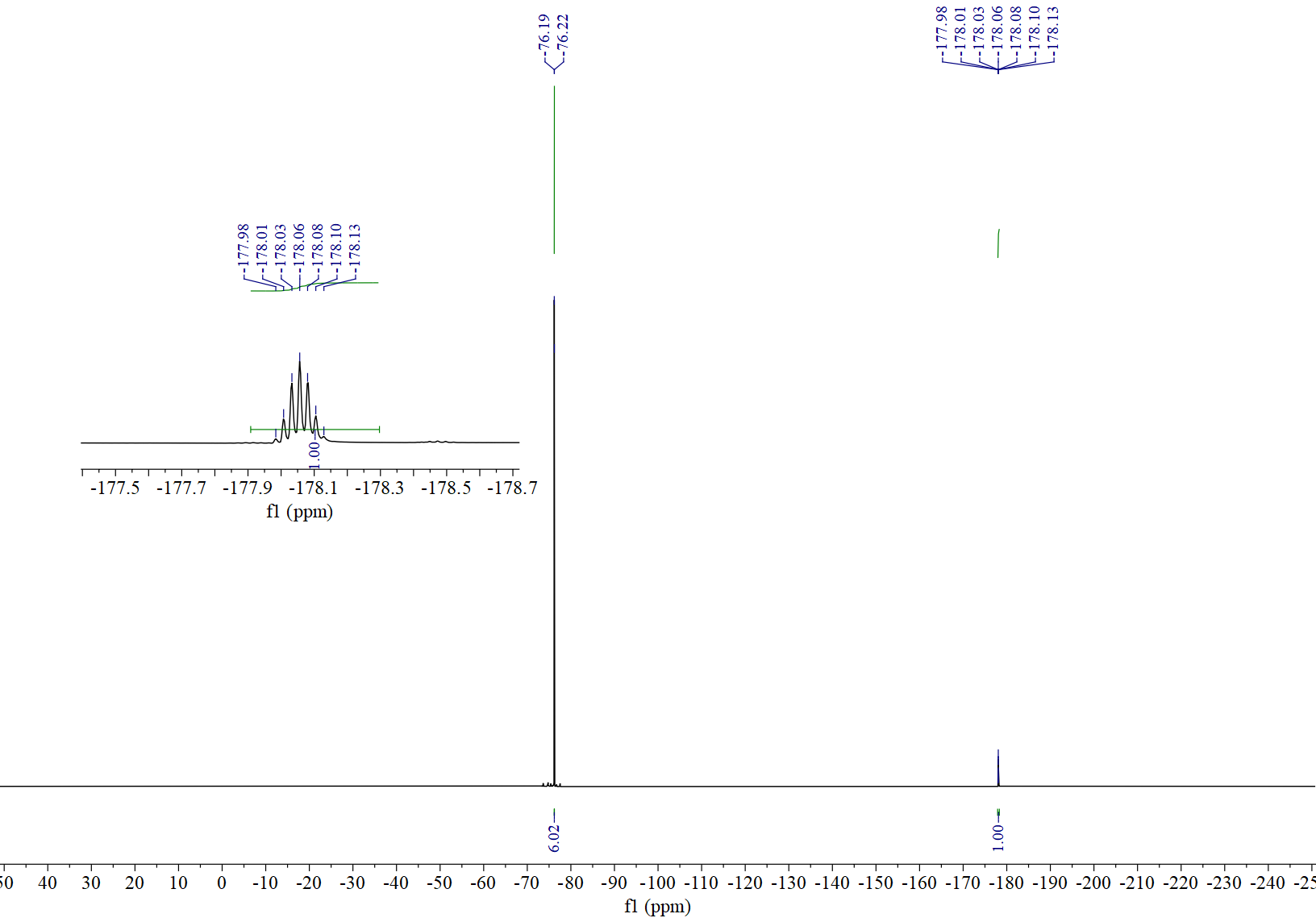

**^19^F NMR** of **23** (376 MHz, Chloroform-*d*, 298 K)


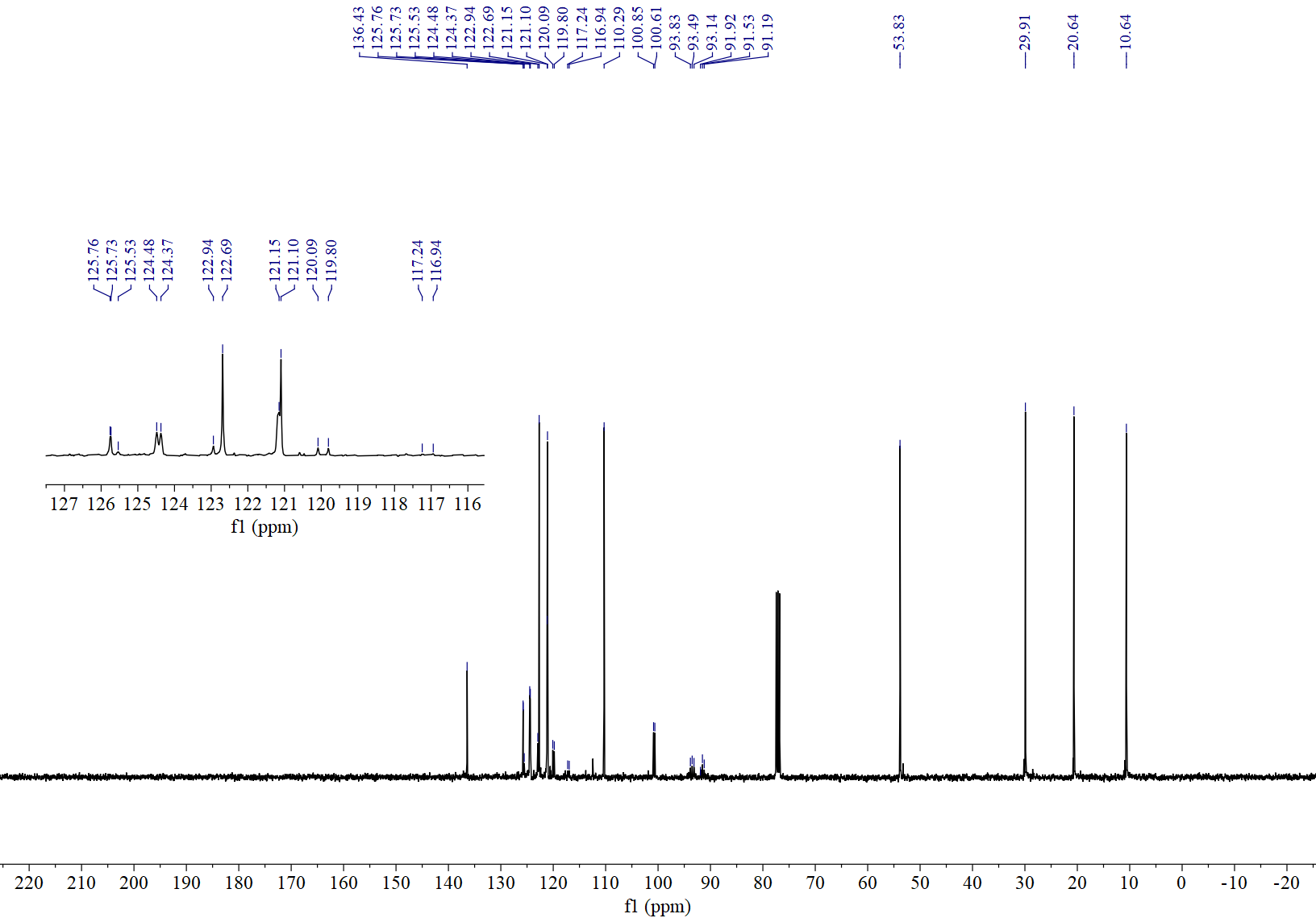

**^13^C NMR** of **23** (101 MHz, Chloroform-*d*, 298 K)


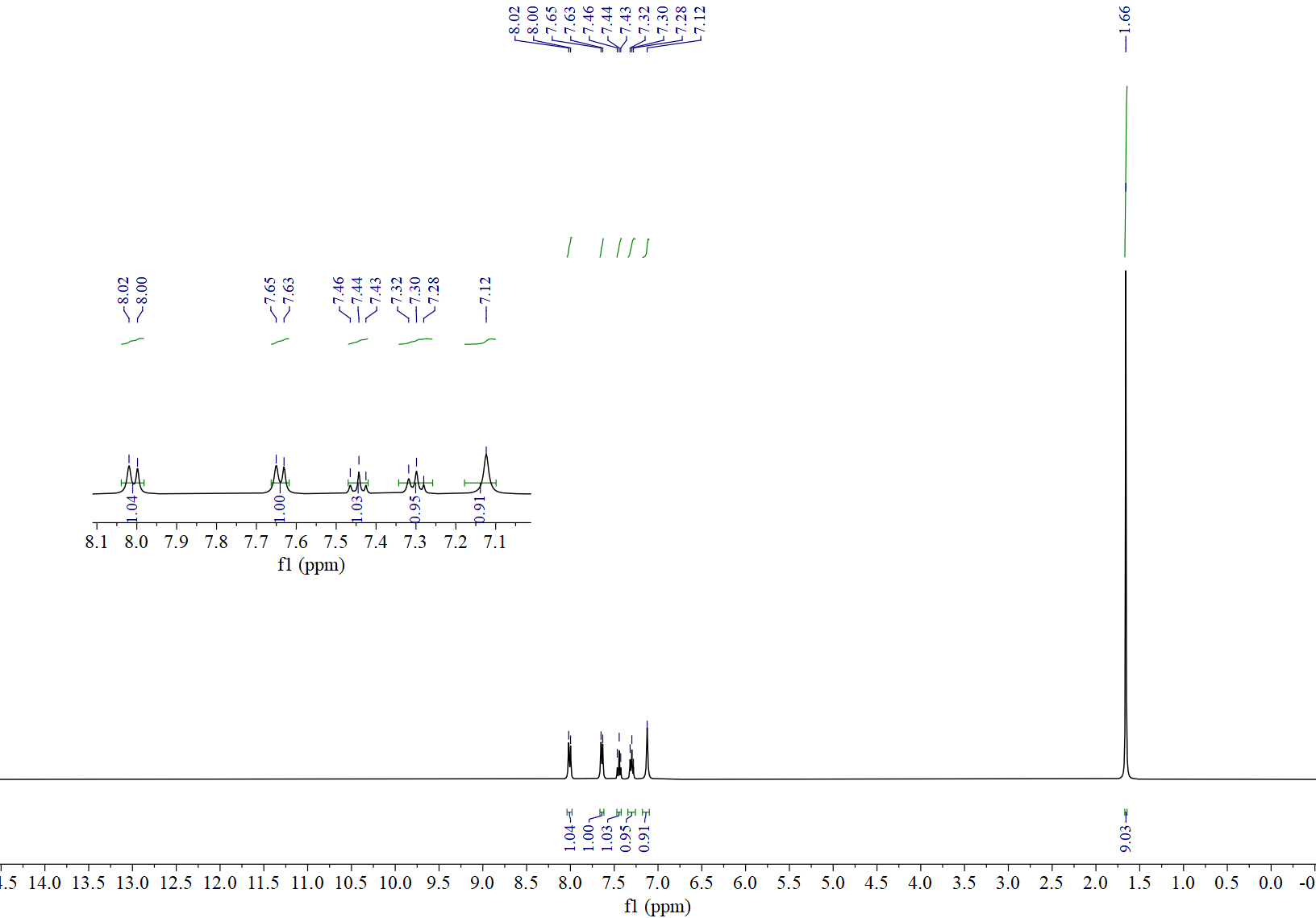

**^1^H NMR** of **24** (400 MHz, Chloroform-*d*, 298 K)


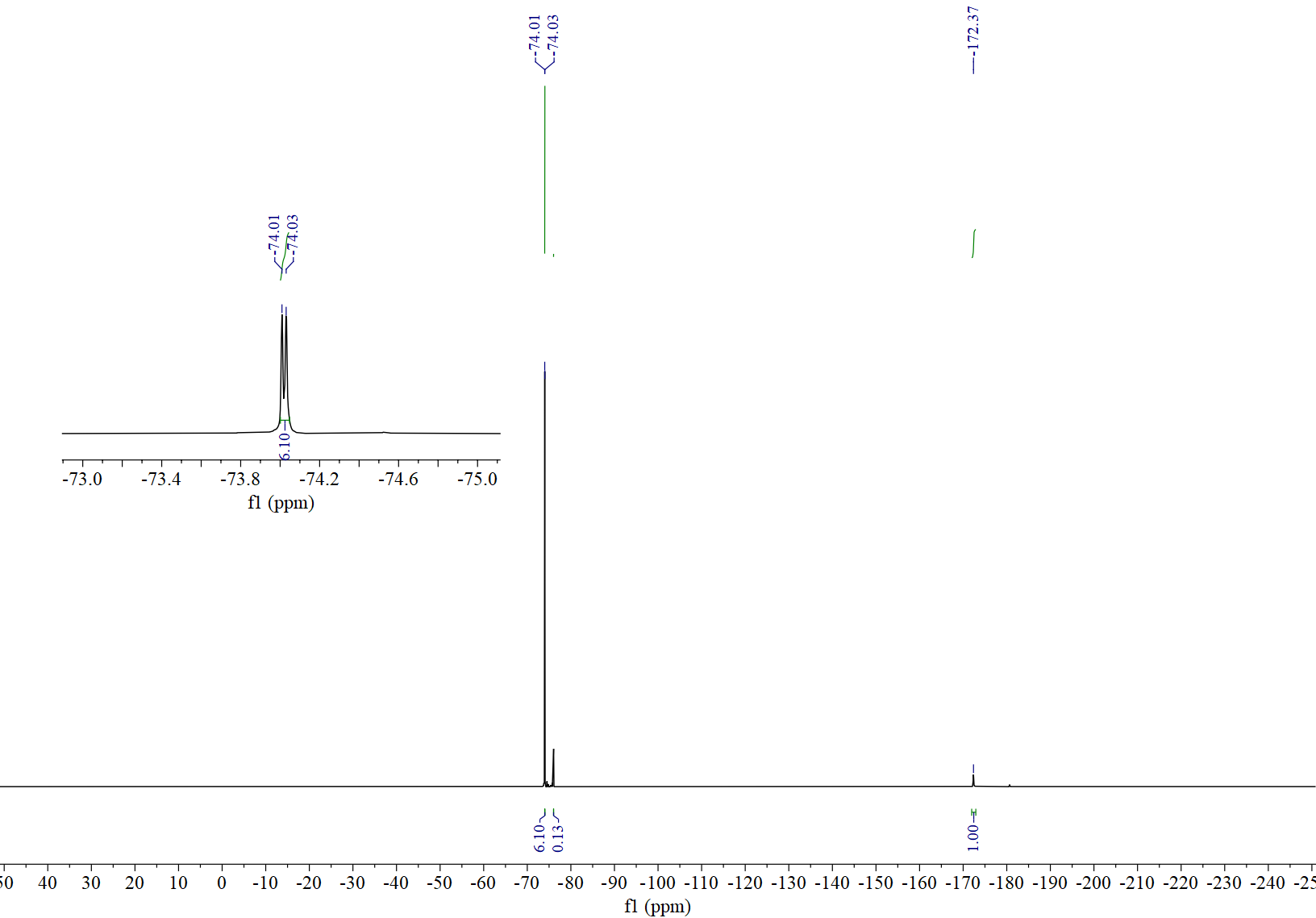

**^19^F NMR** of **24** (376 MHz, Chloroform-*d*, 298 K)


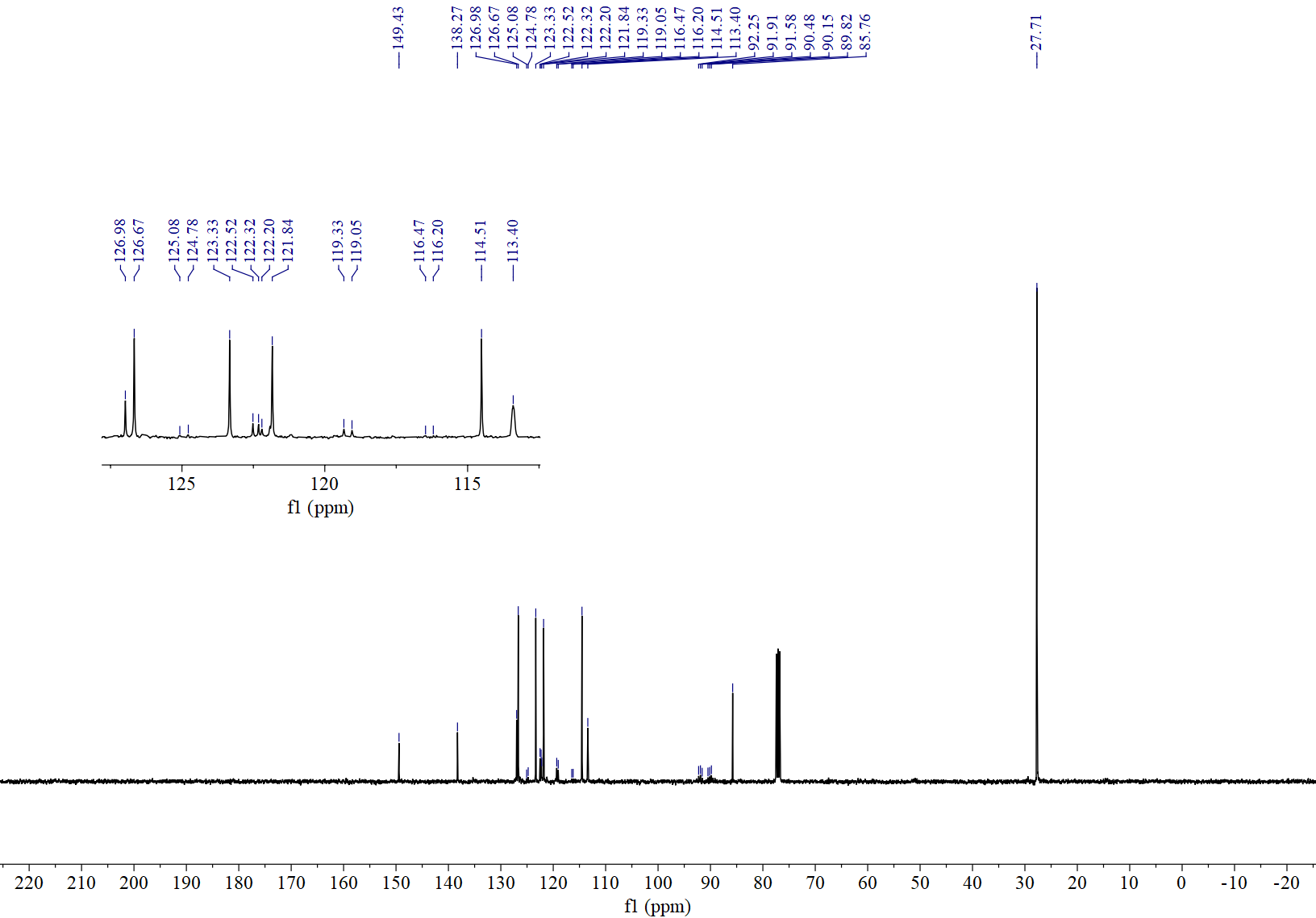

**^13^C NMR** of **24** (101 MHz, Chloroform-*d*, 298 K)


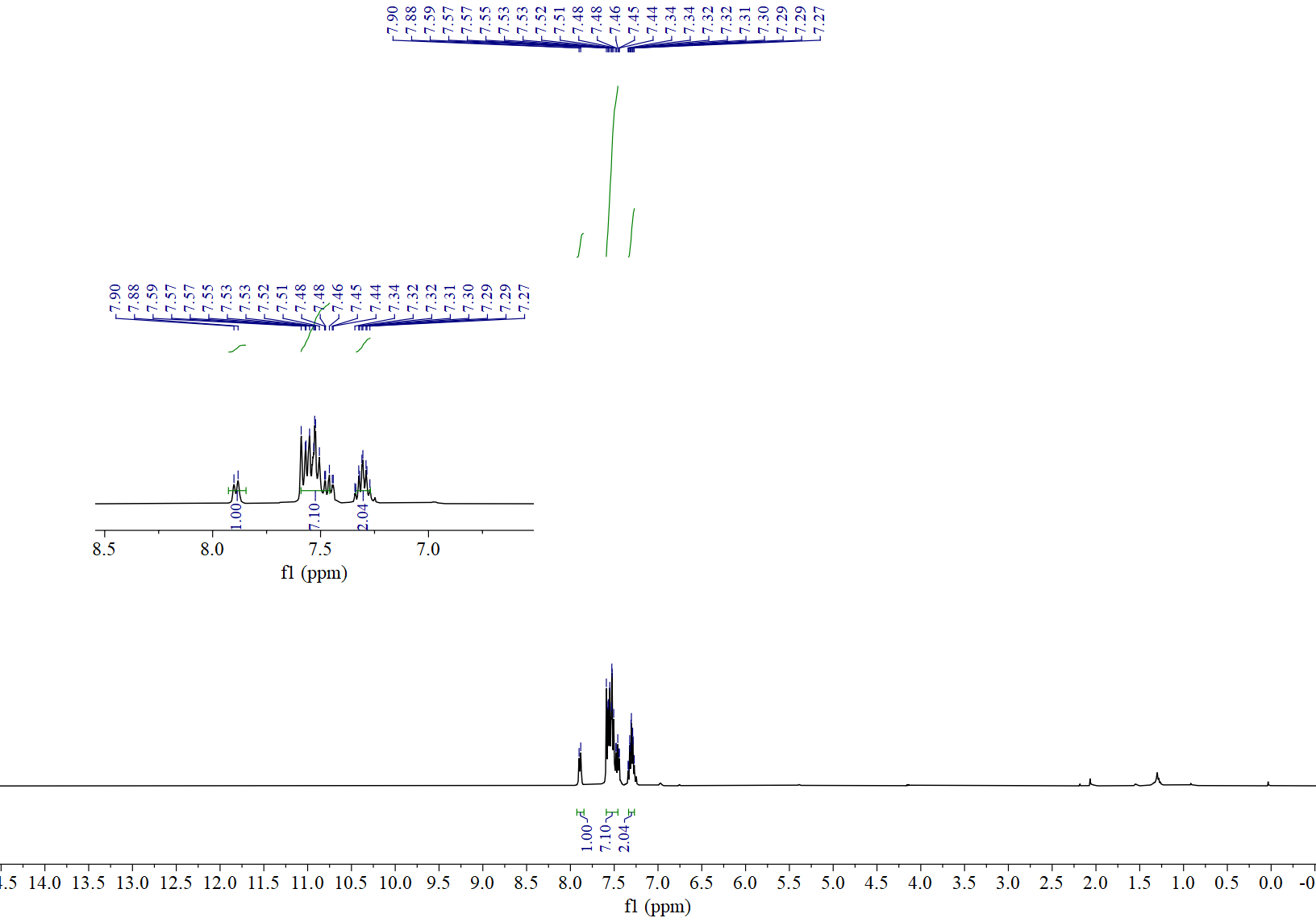

**^1^H NMR** of **25** (400 MHz, Chloroform-*d*, 298 K)


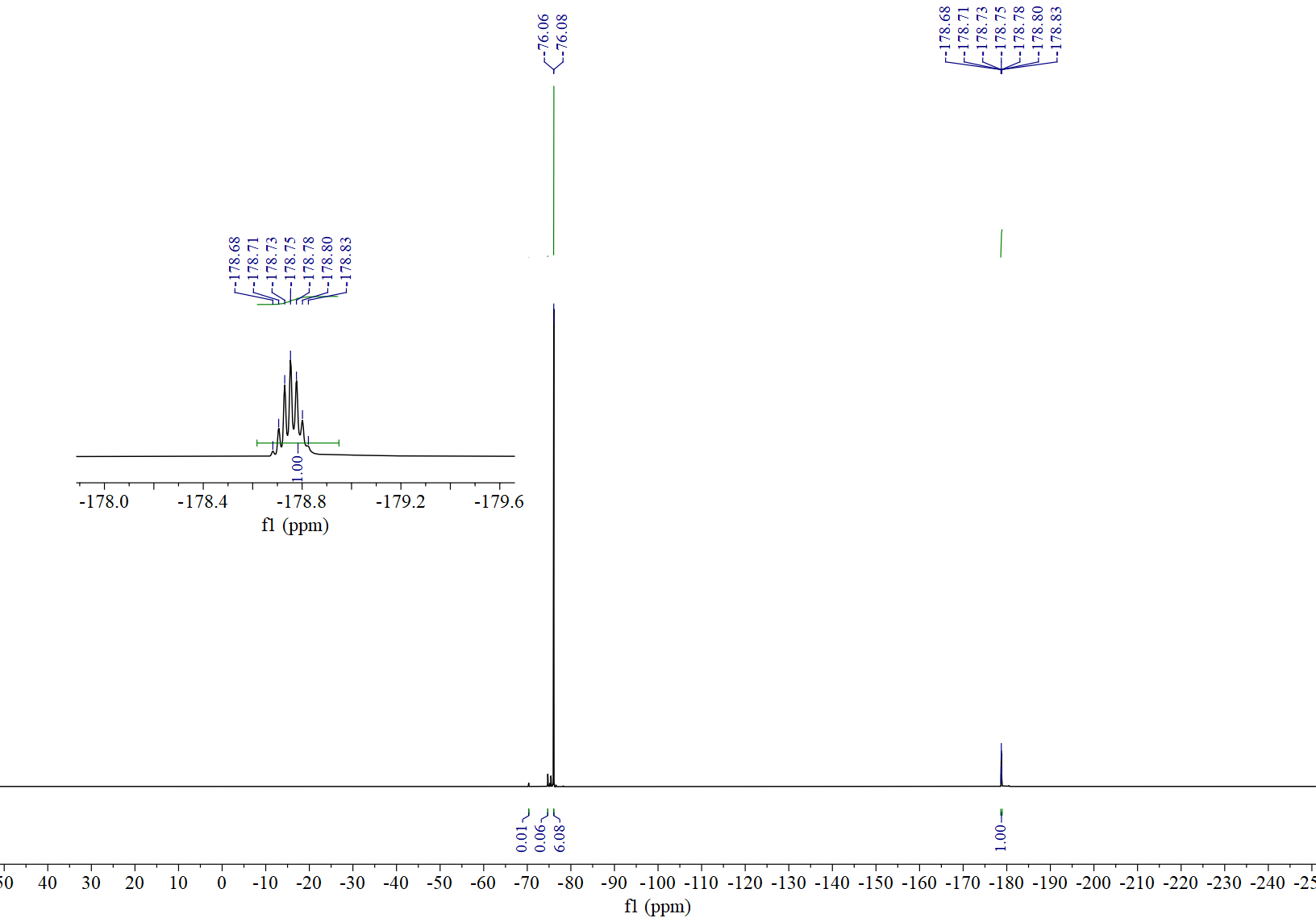

**^19^F NMR** of **25** (376 MHz, Chloroform-*d*, 298 K)


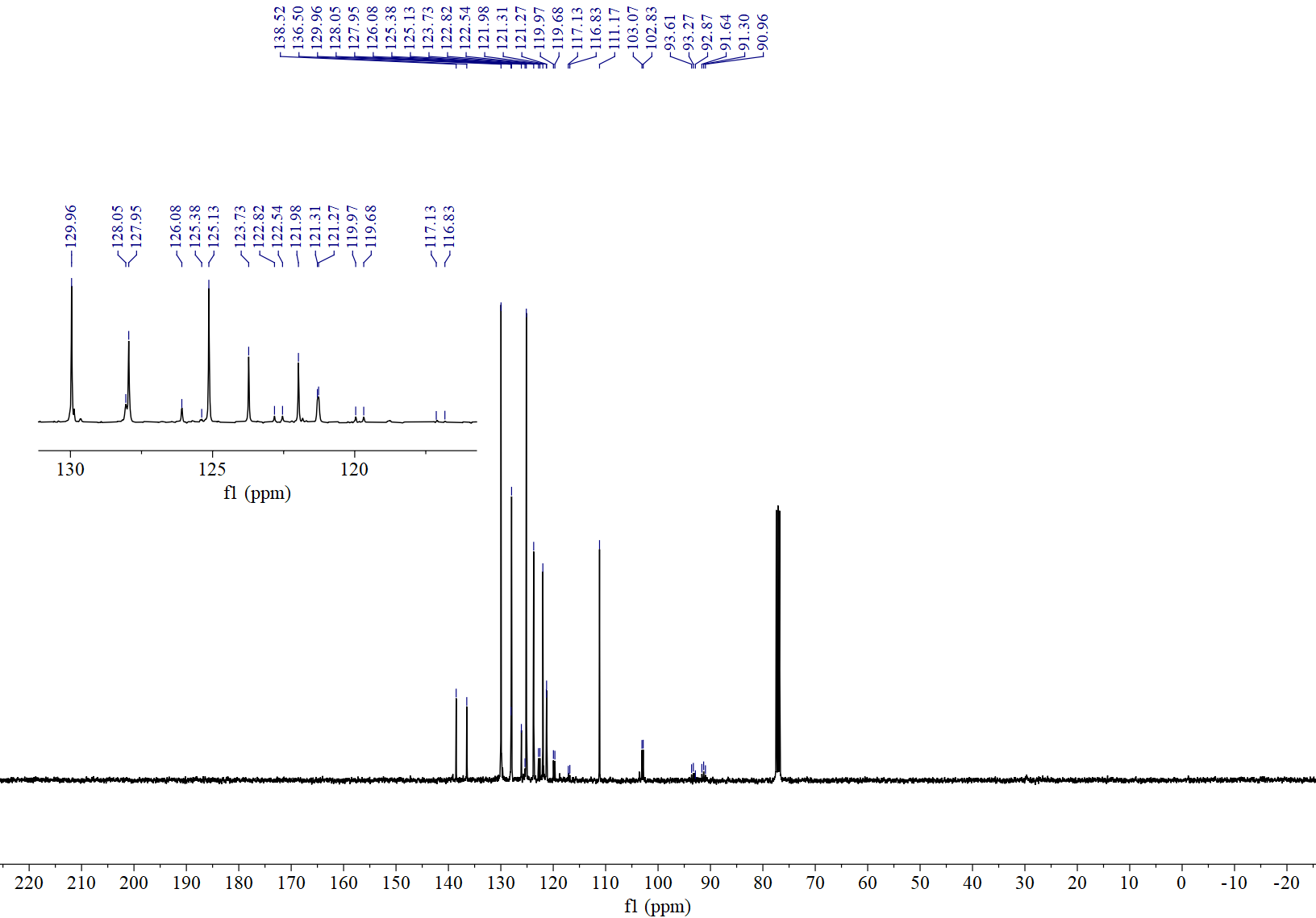

**^13^C NMR** of **25** (100 MHz, Chloroform-*d*, 298 K)


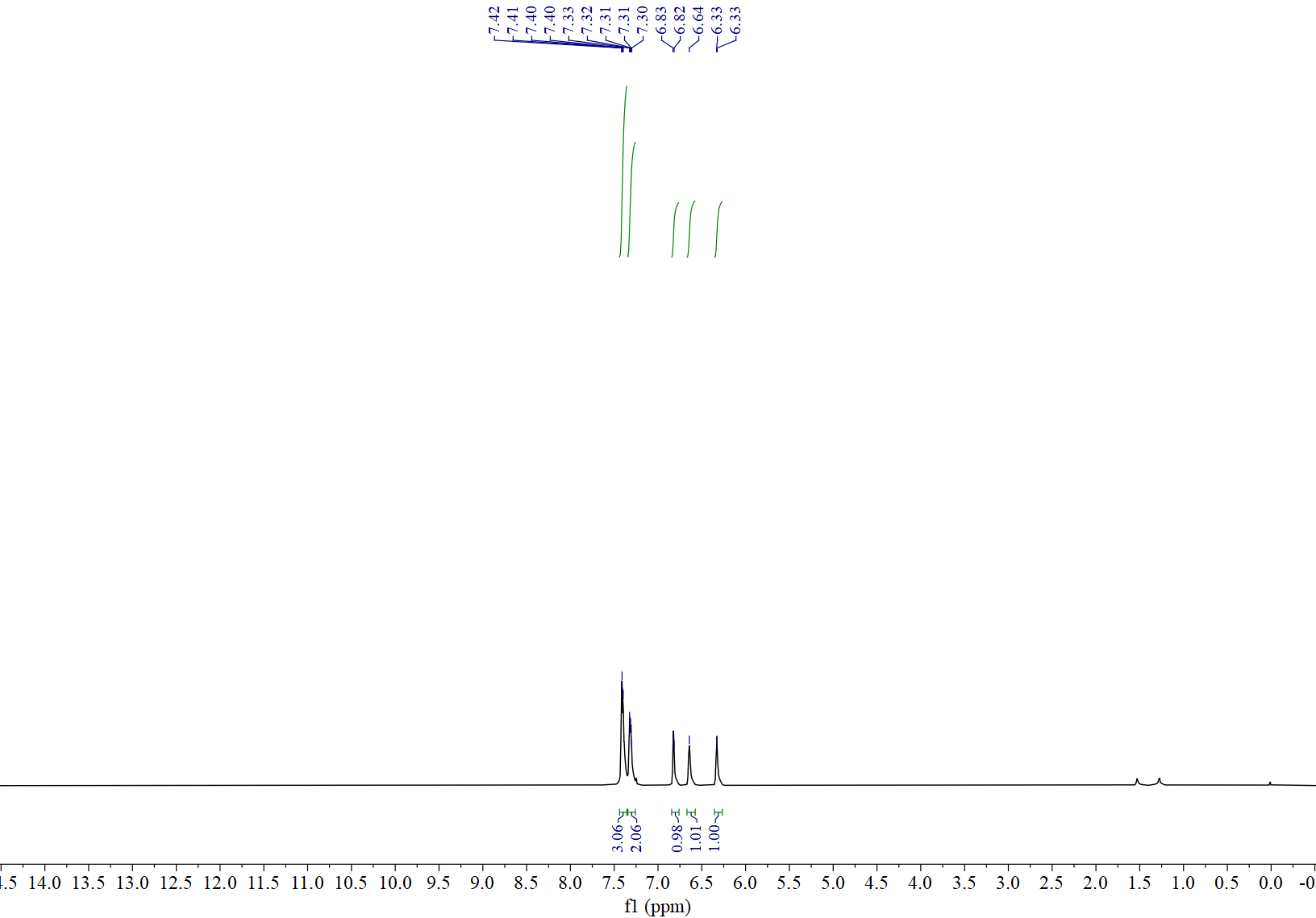

**^1^H NMR** of **26** (400 MHz, Chloroform-*d*, 298 K)


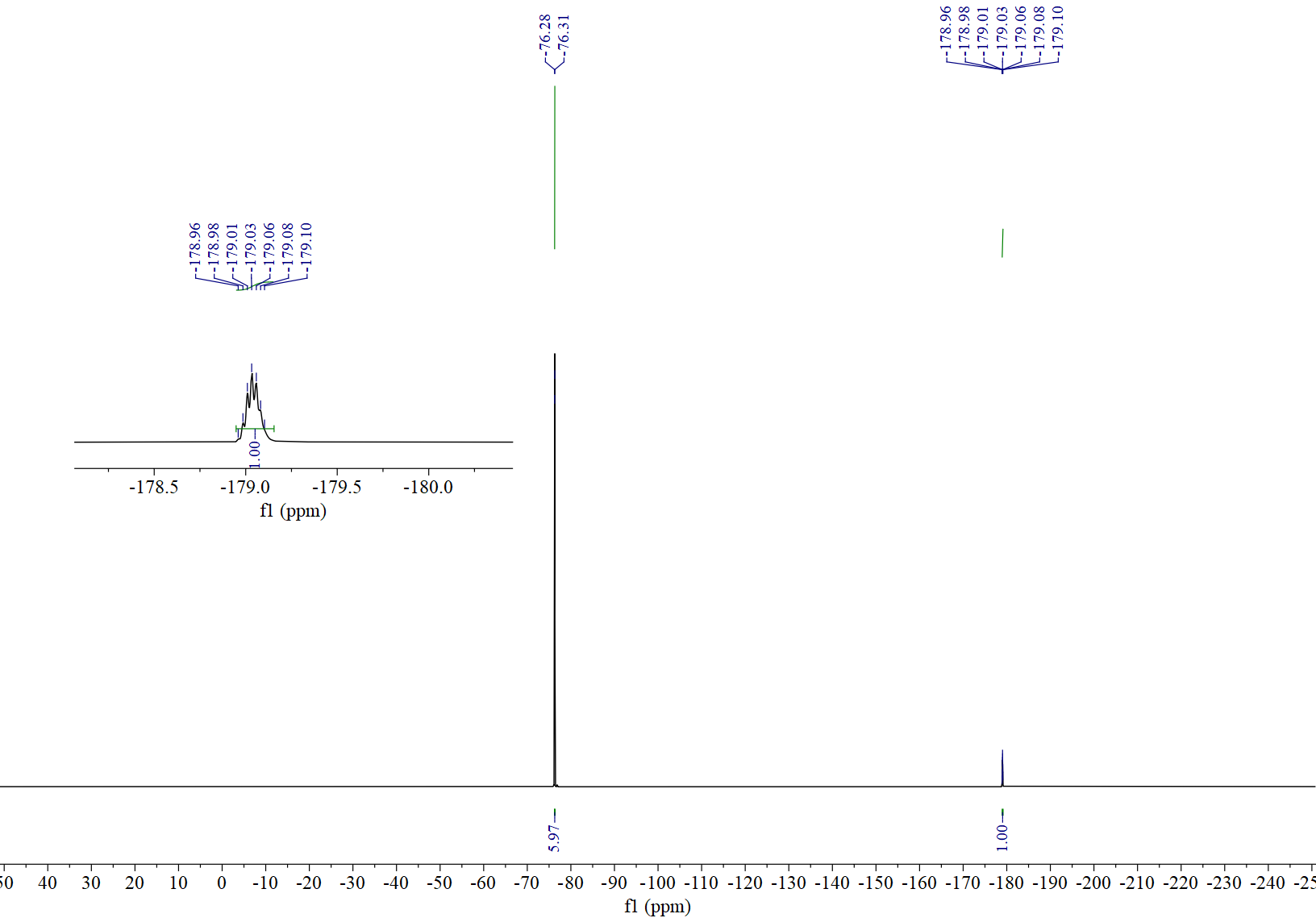

**^19^F NMR** of **26** (376 MHz, Chloroform-*d*, 298 K)


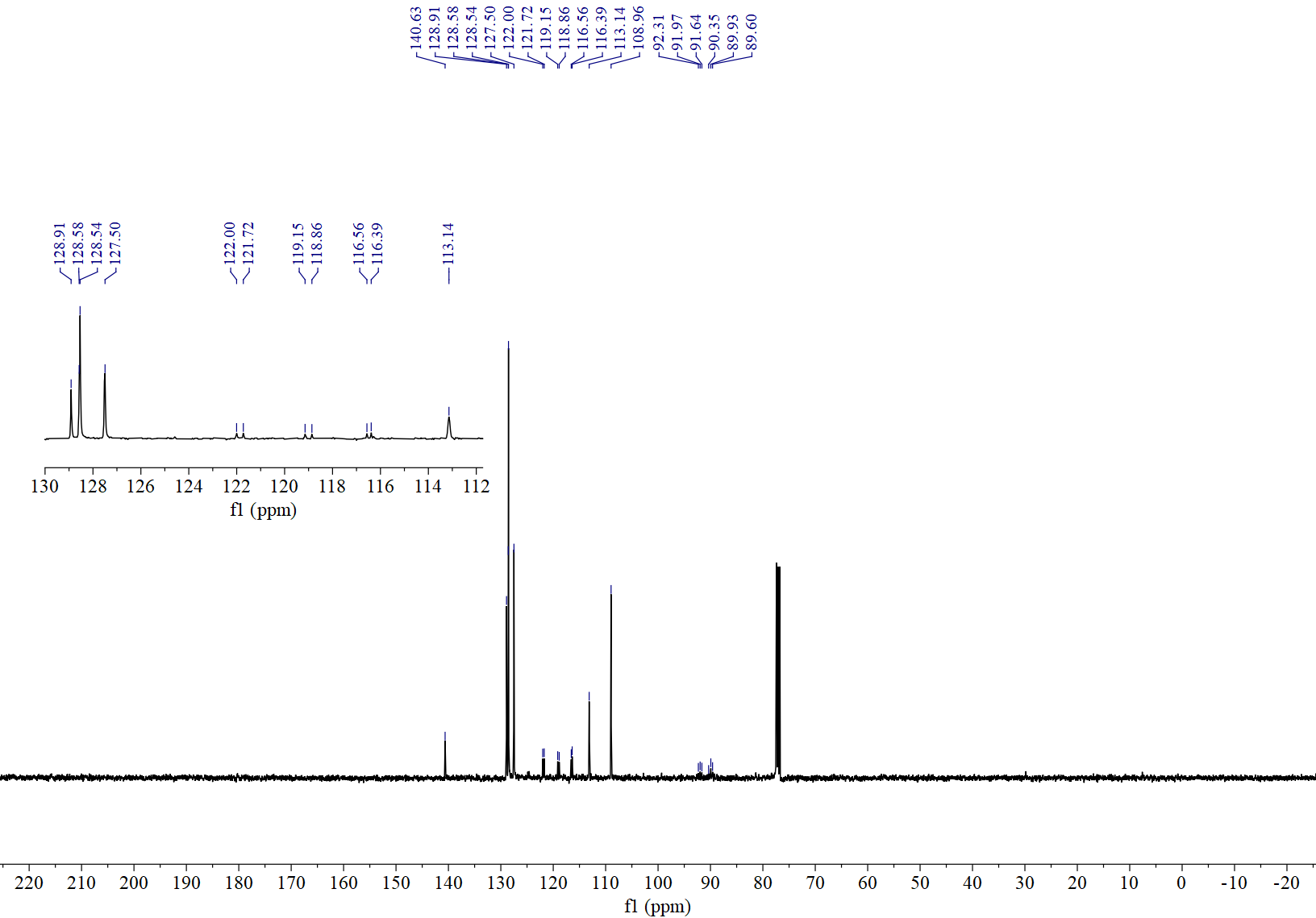

**^13^C NMR** of **26** (101 MHz, Chloroform-*d*, 298 K)


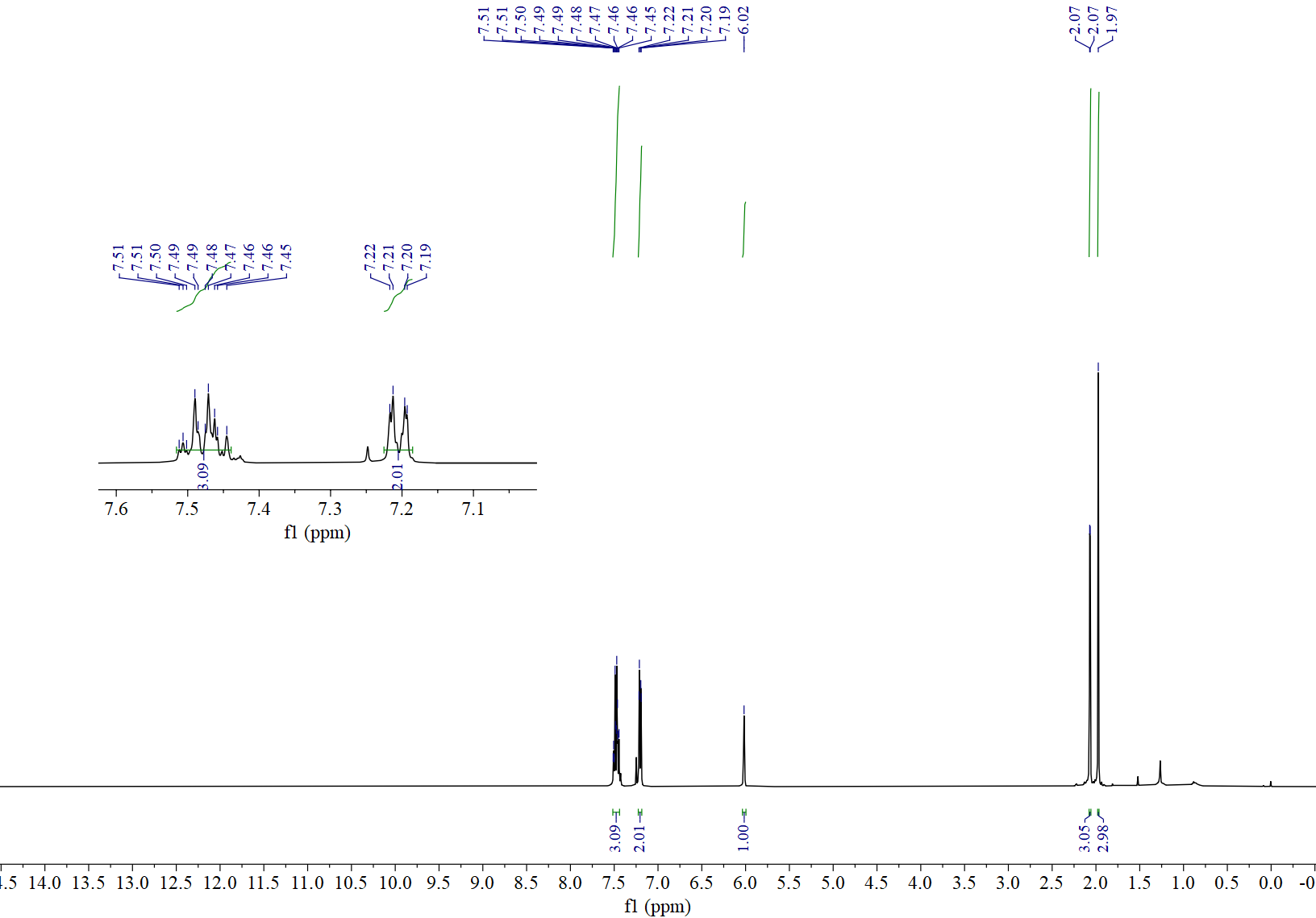

**^1^H NMR** of **27** (400 MHz, Chloroform-*d*, 298 K)


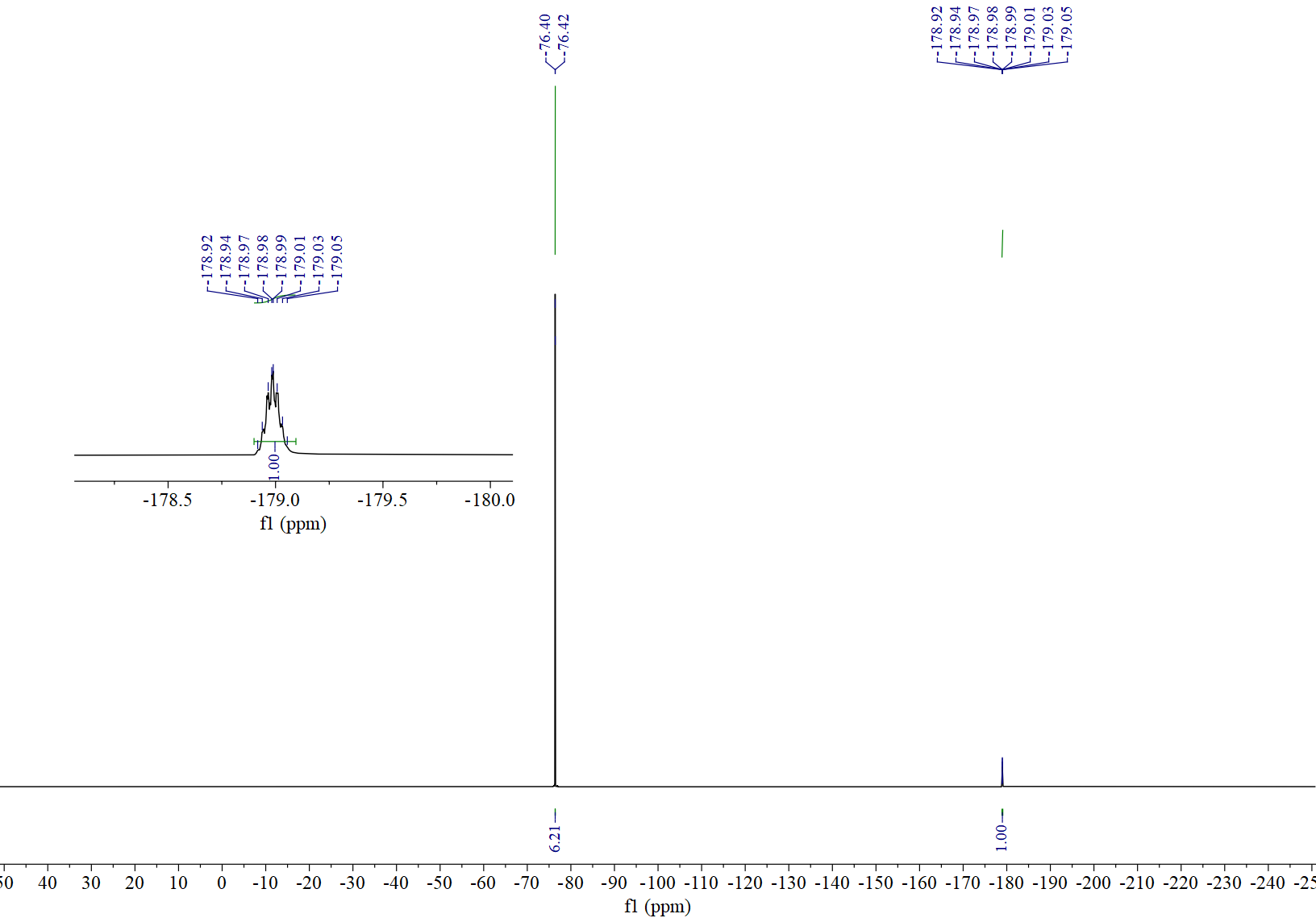

**^19^F NMR** of **27** (376 MHz, Chloroform-*d*, 298 K)


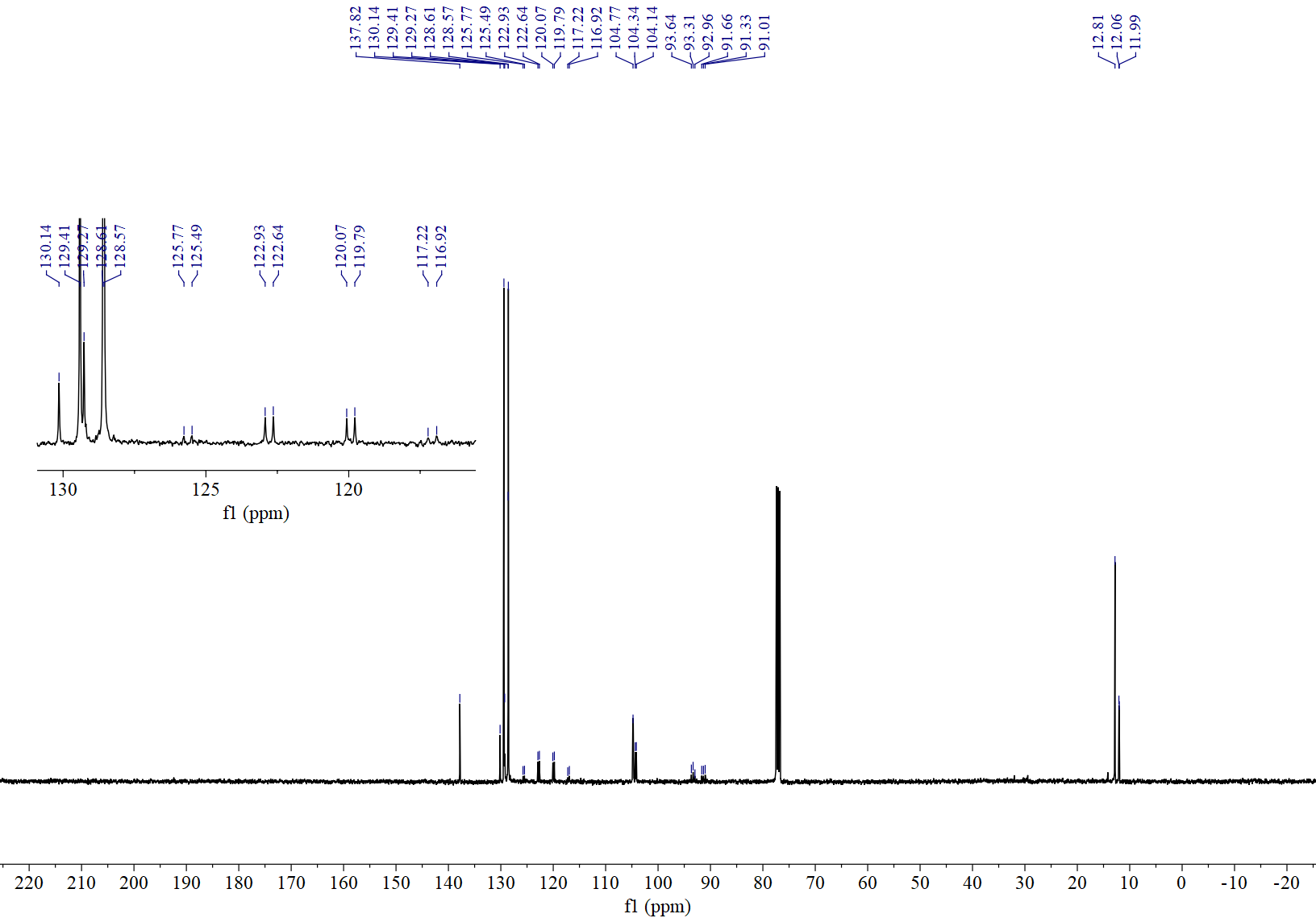

**^13^C NMR** of **27** (101 MHz, Chloroform-*d*, 298 K)


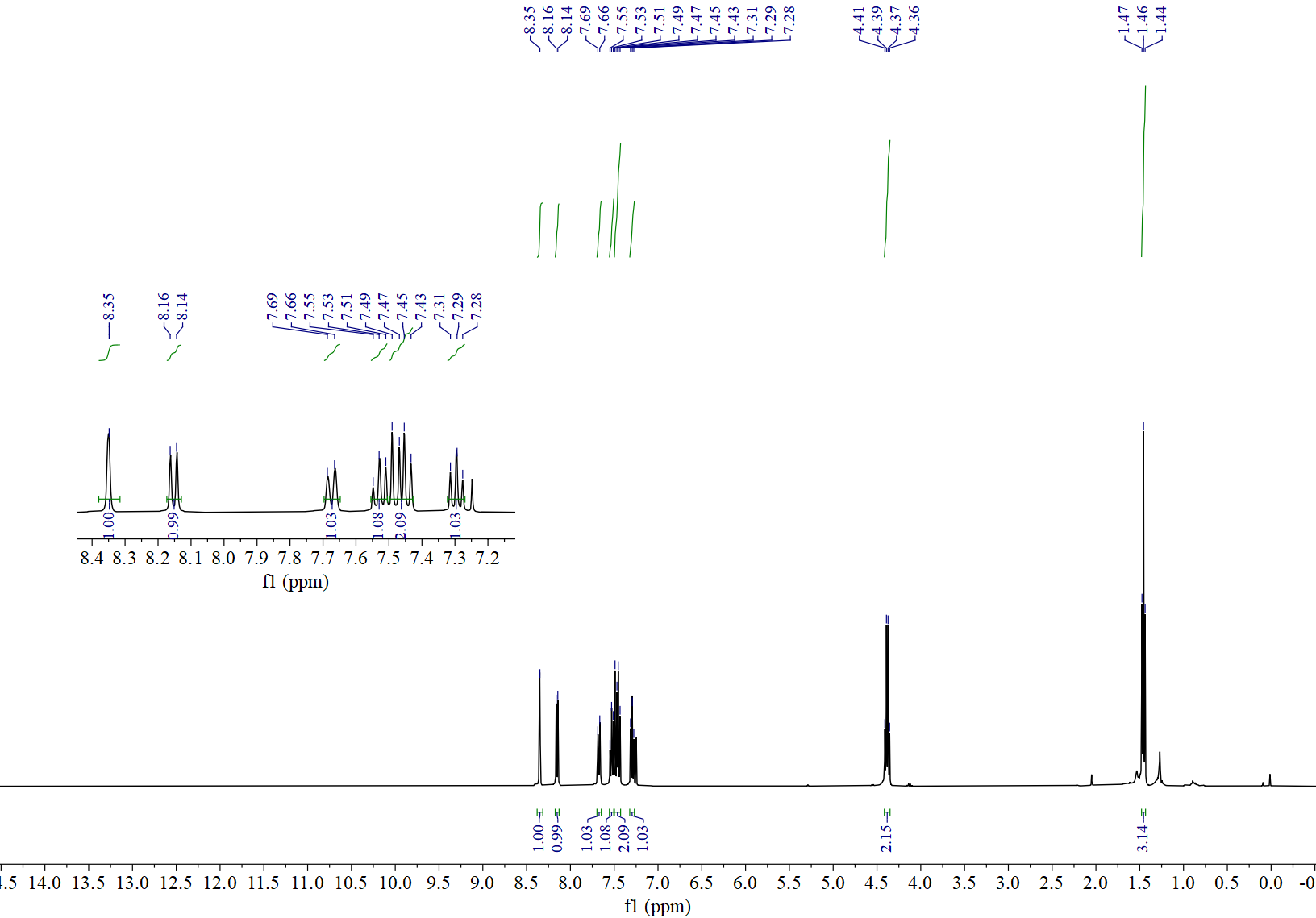

**^1^H NMR** of **28-1** (400 MHz, Chloroform-*d*, 298 K)


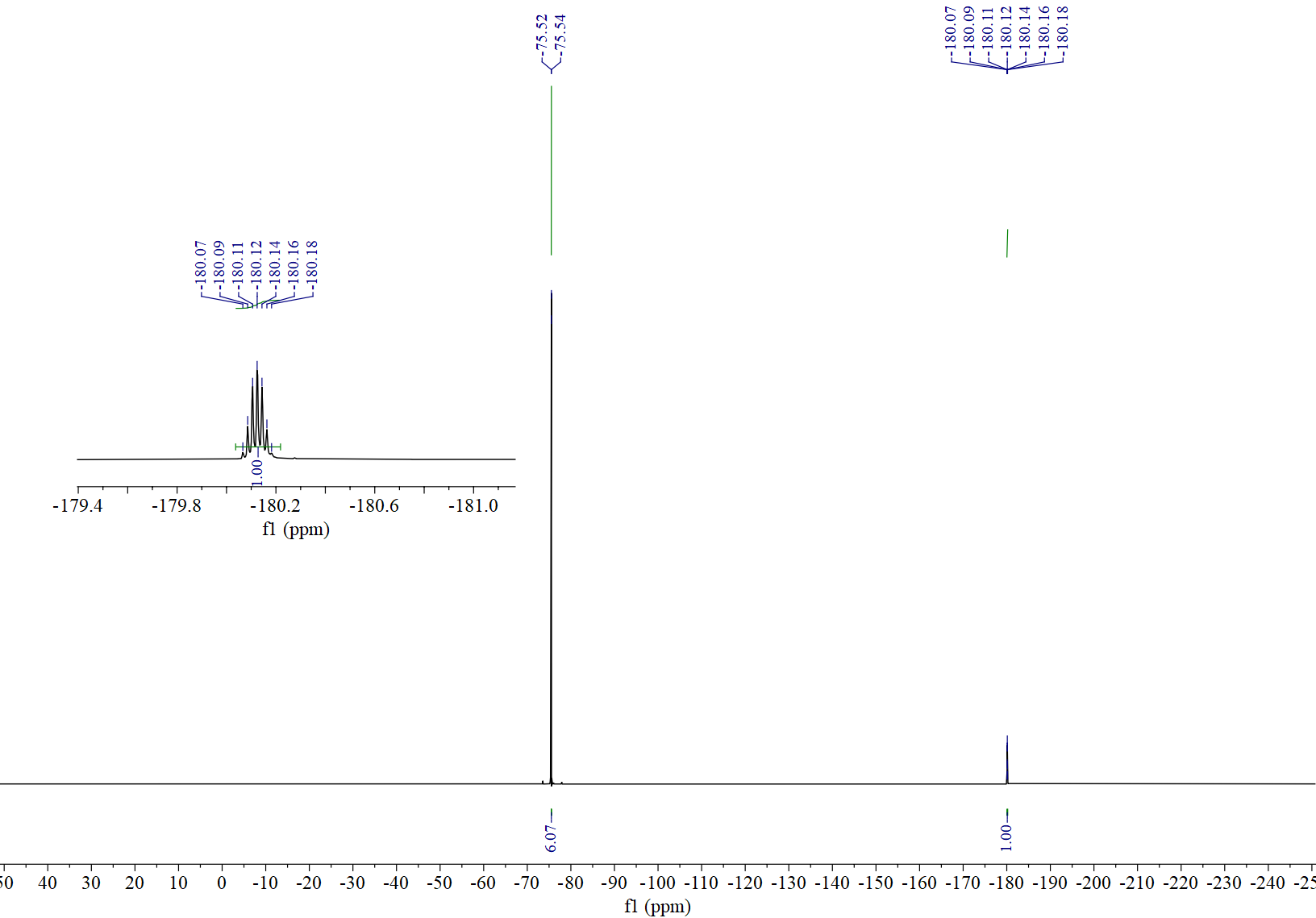

**^19^F NMR** of **28-1** (376 MHz, Chloroform-*d*, 298 K)


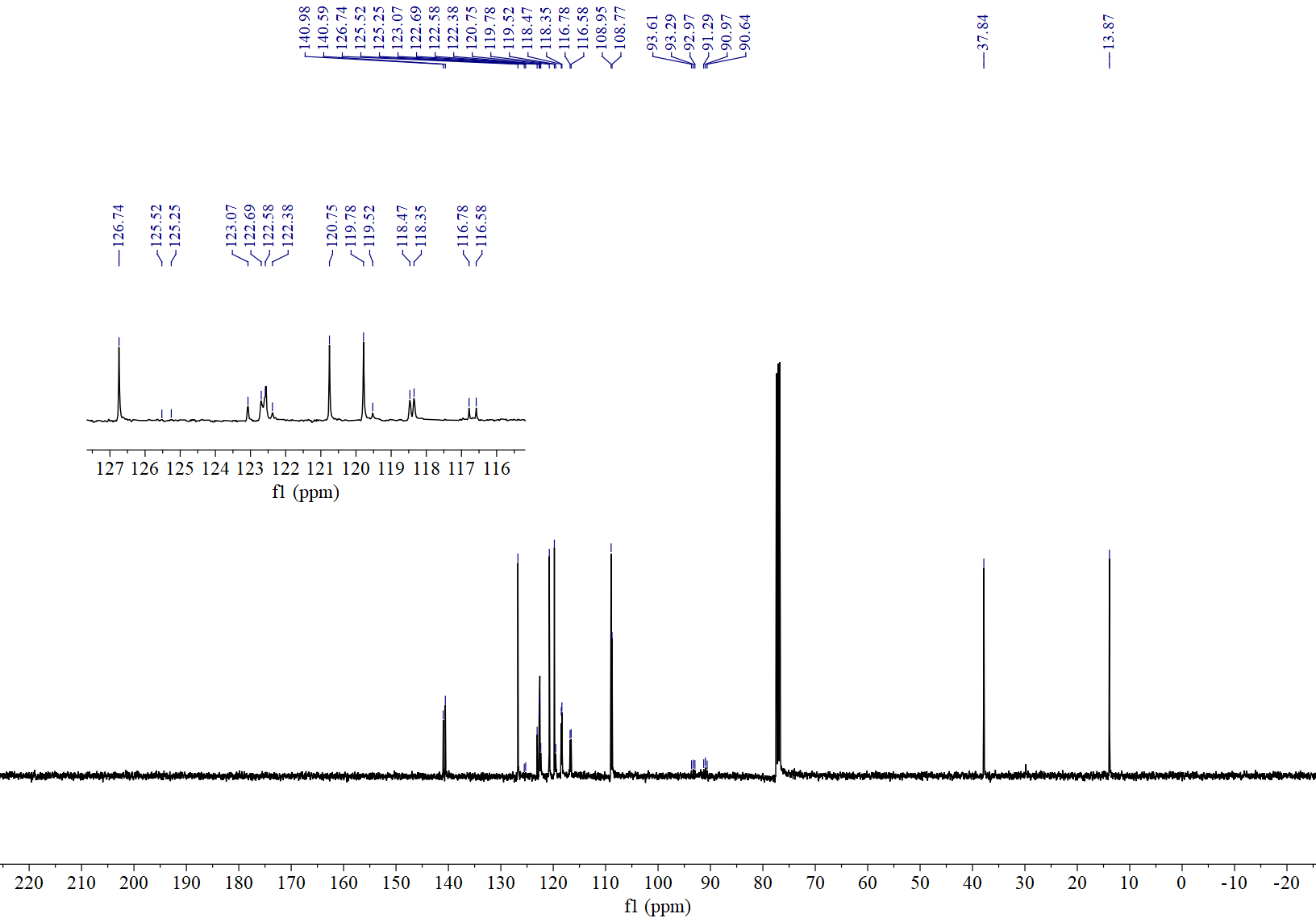

**^13^C NMR** of **28-1** (101 MHz, Chloroform-*d*, 298 K)


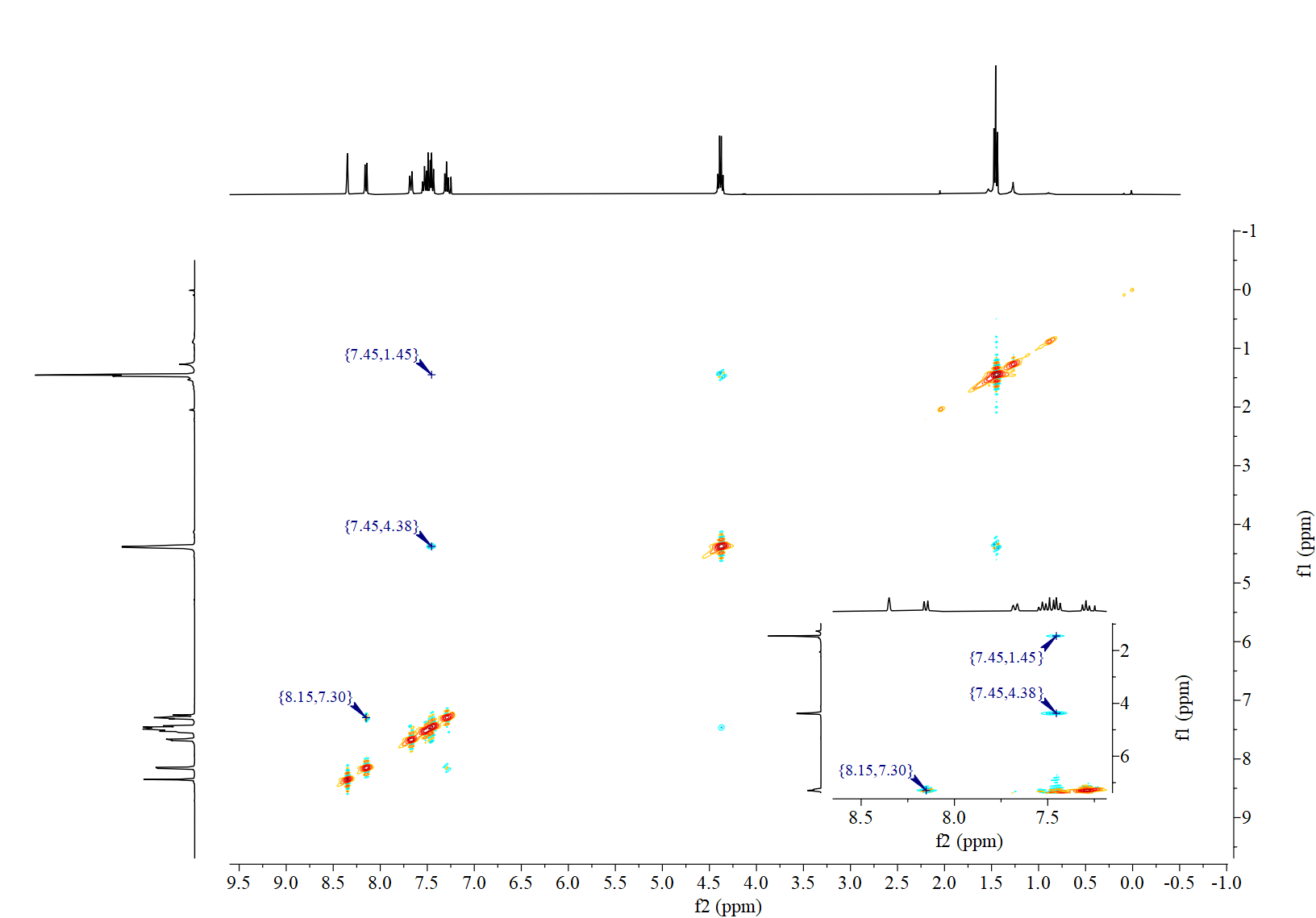
**NOESY NMR** of **28-1** (400 MHz, Chloroform-*d*, 298 K)

**H^1^, H^2^**

**H^3^**

**H^4^**

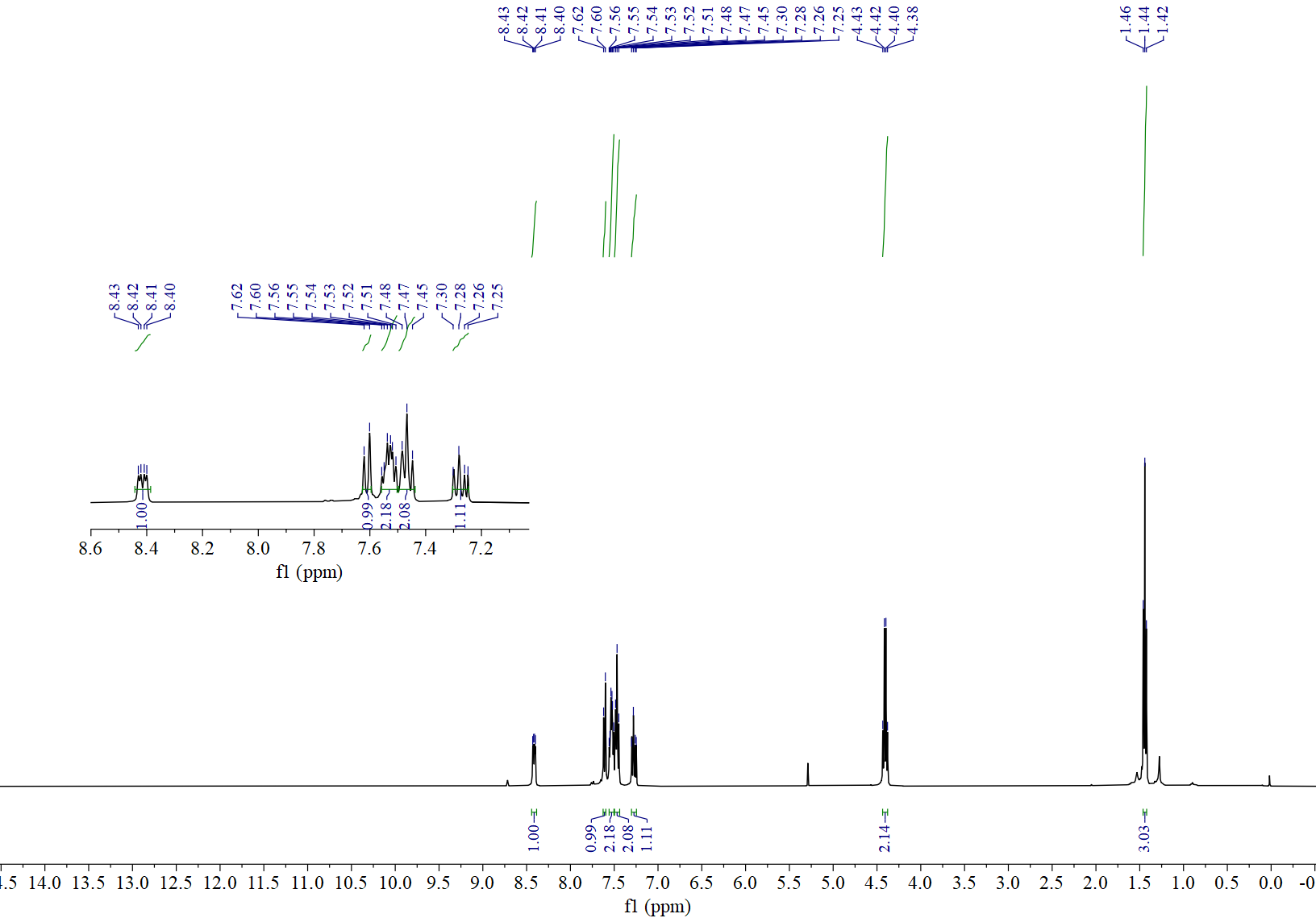

**^1^H NMR** of **28-2** (400 MHz, Chloroform-*d*, 298 K)


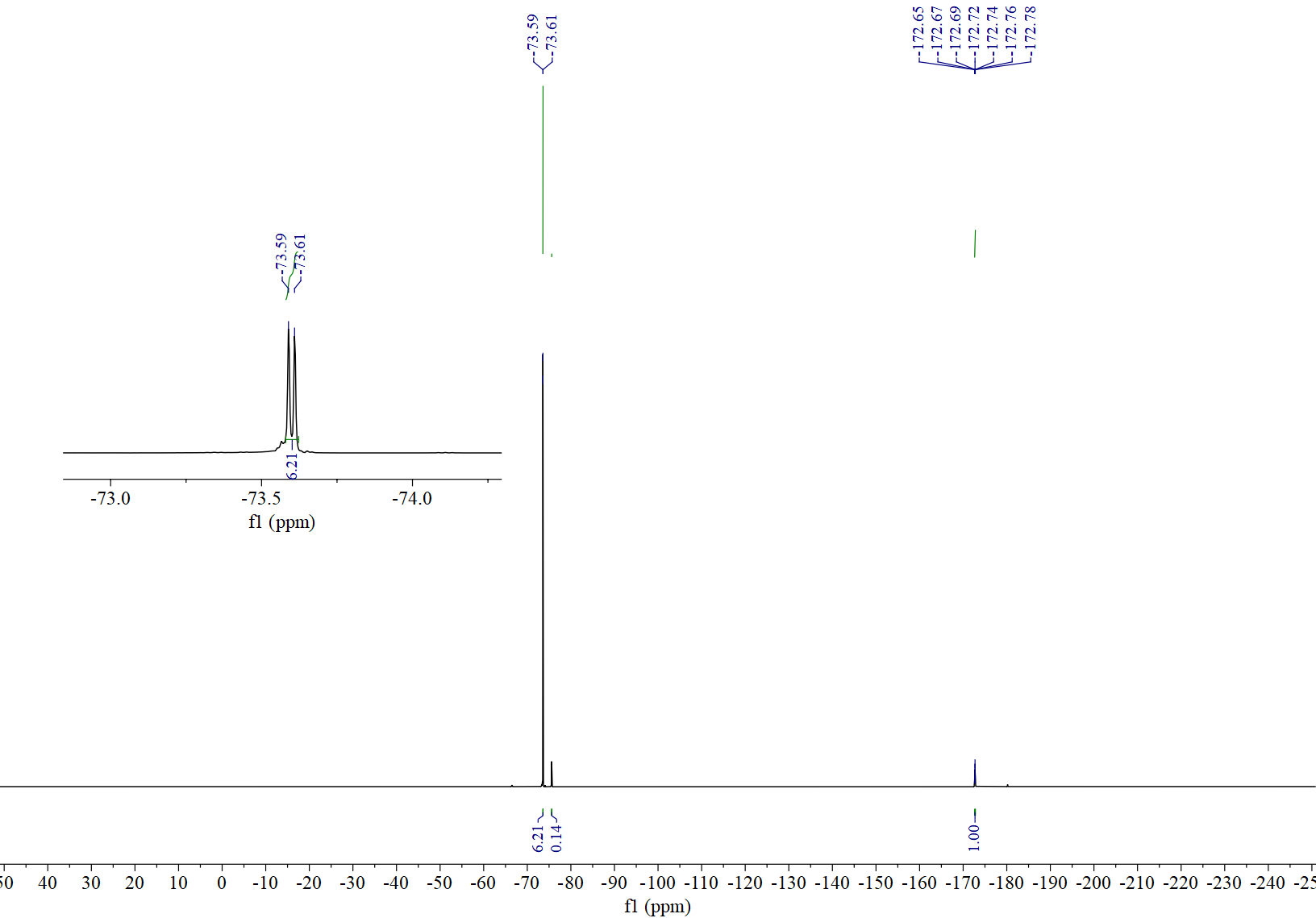

**^19^F NMR** of **28-2** (376 MHz, Chloroform-*d*, 298 K)


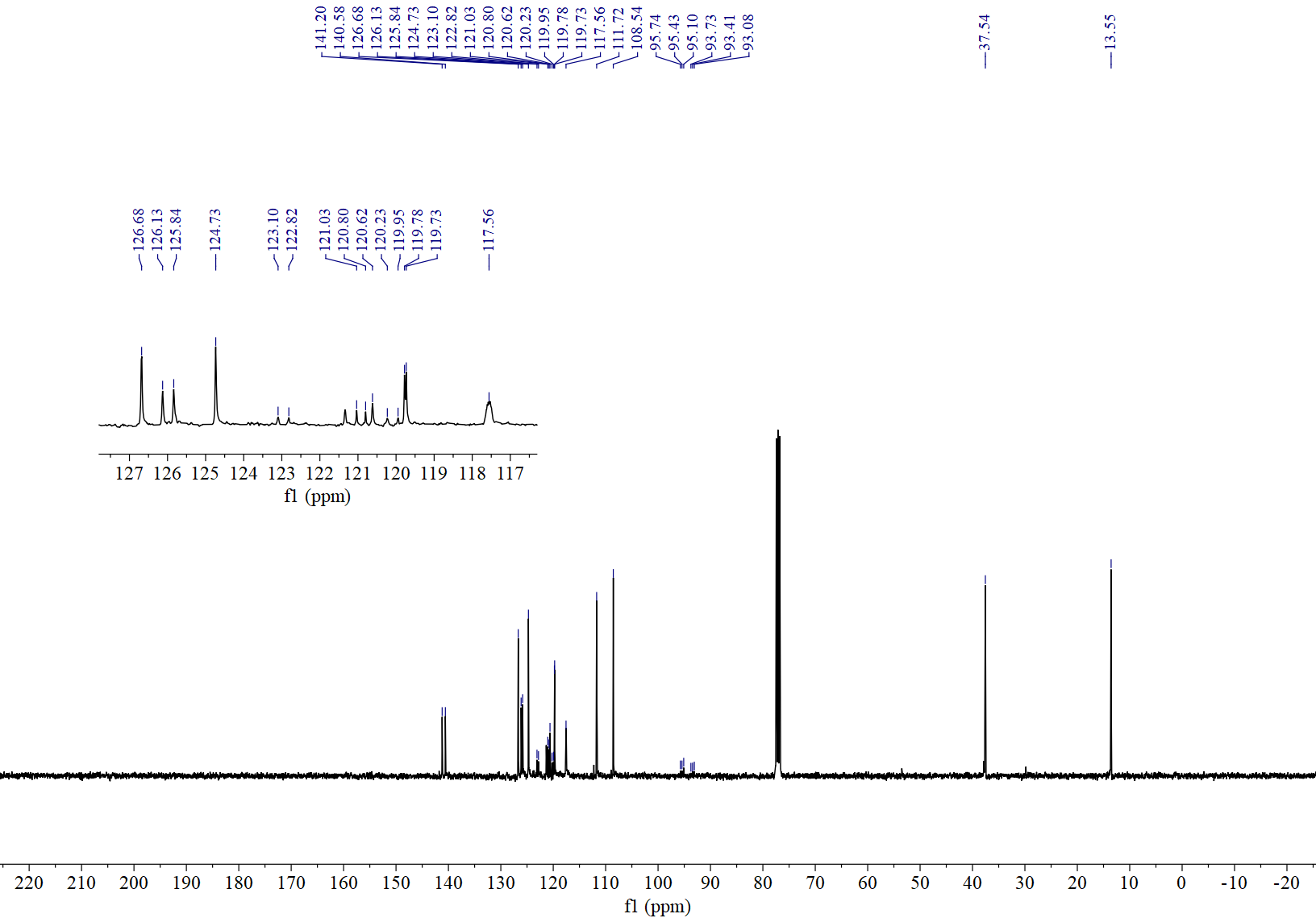

**^13^C NMR** of **28-2** (101 MHz, Chloroform-*d*, 298 K)

**NOESY NMR** of **28-2** (400 MHz, Chloroform-*d*, 298 K)
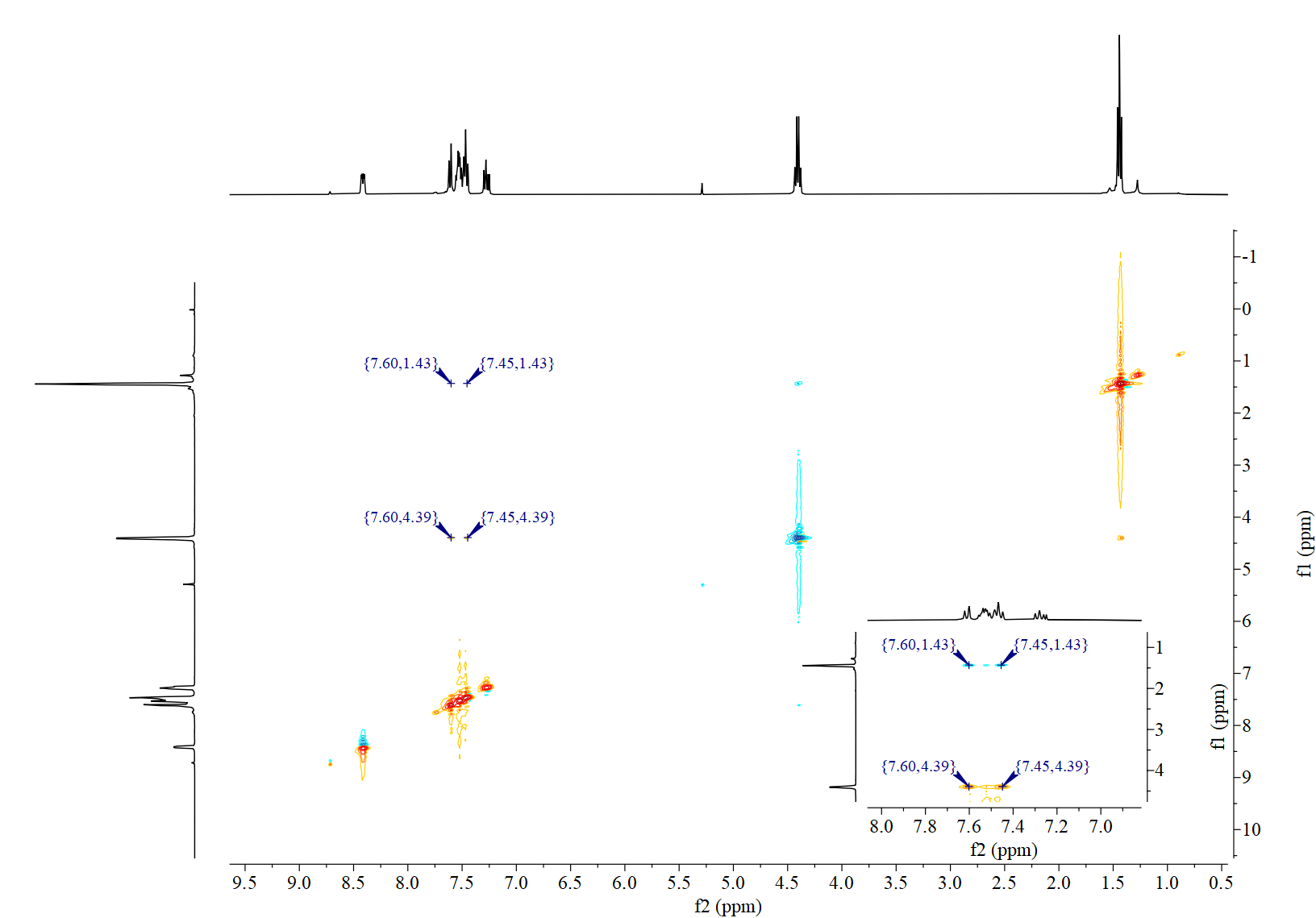


**H^2^**

**H^1^**

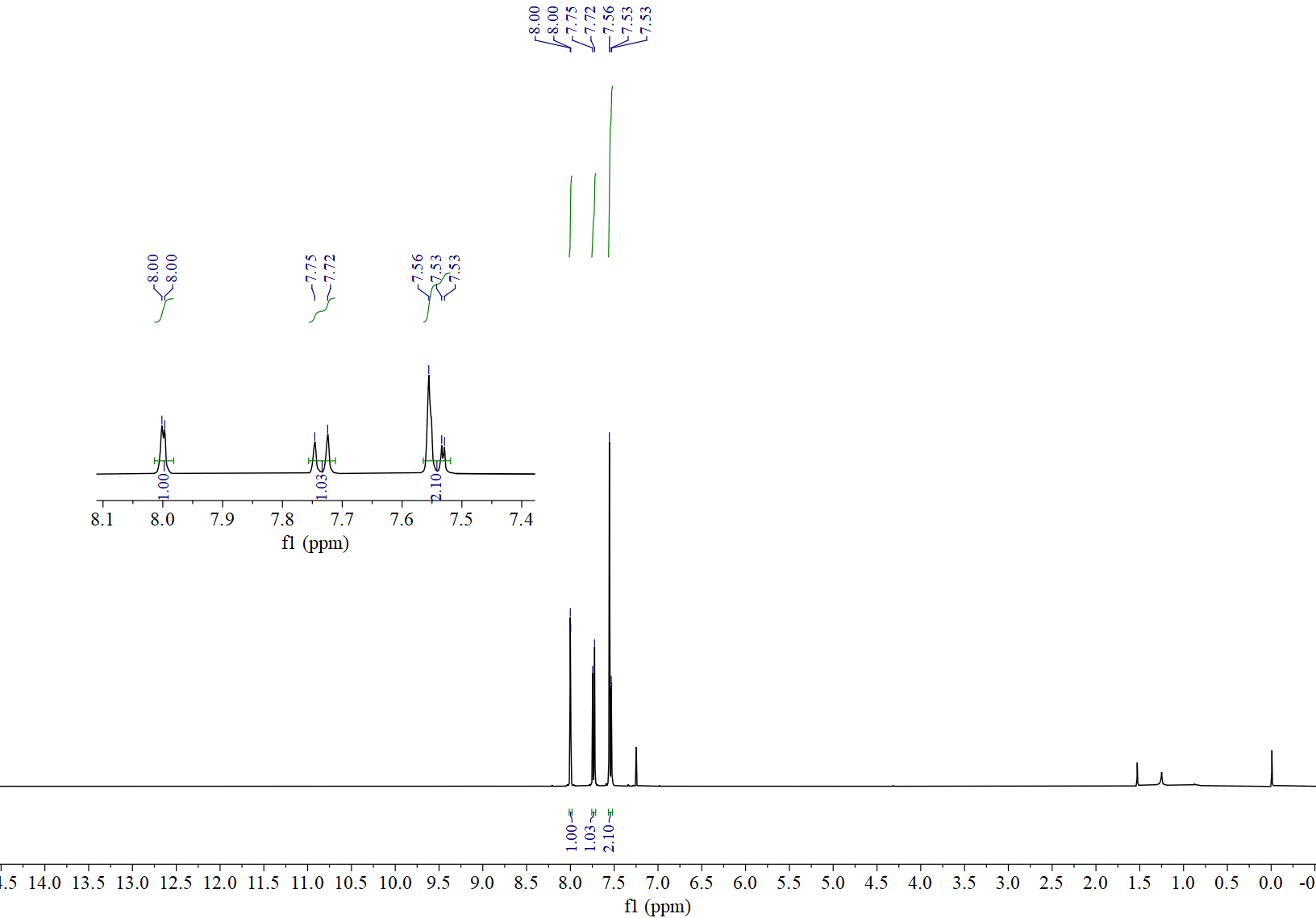

**^1^H NMR** of **29** (400 MHz, Chloroform-*d*, 298 K)


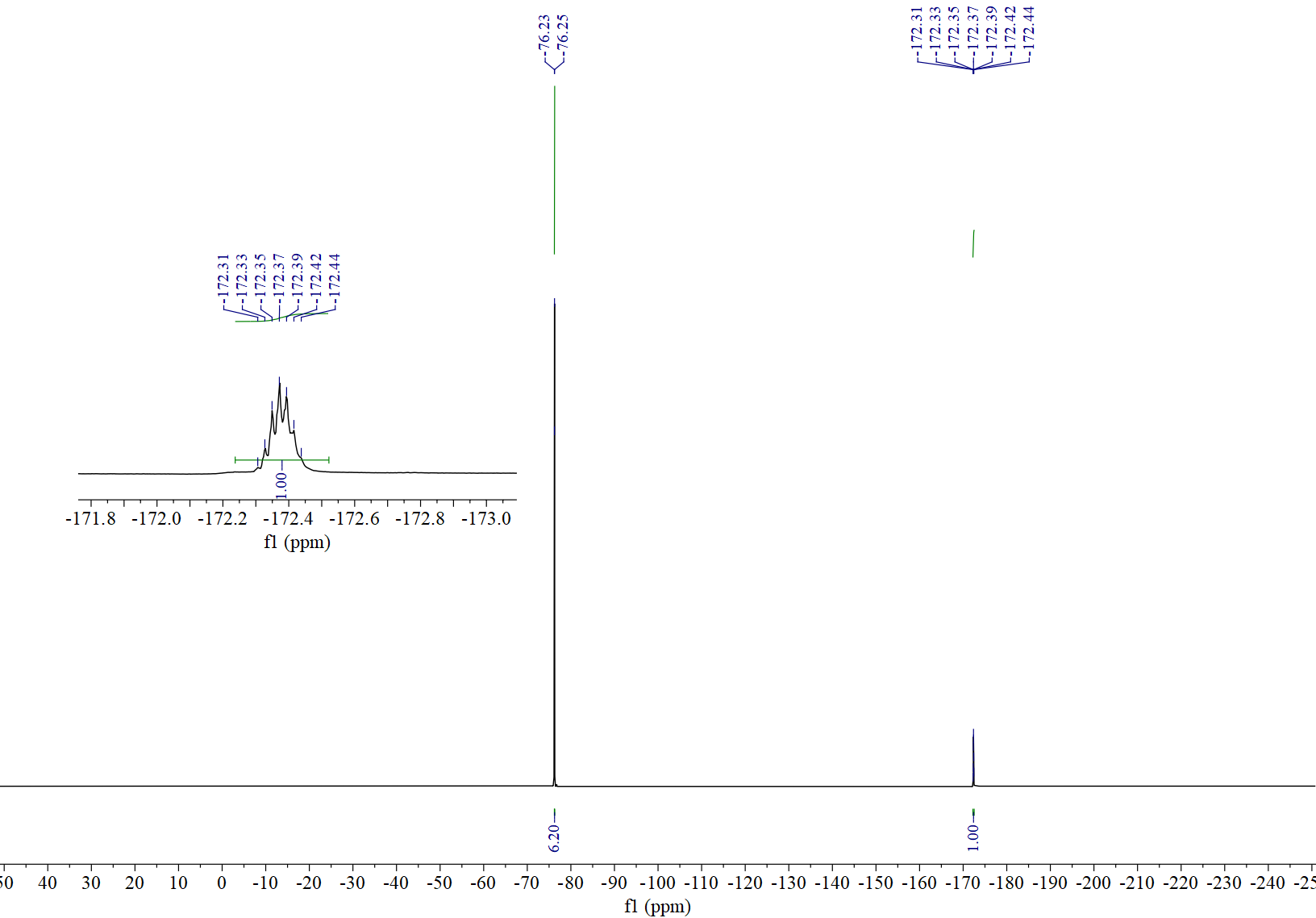

**^19^F NMR** of **29** (376 MHz, Chloroform-*d*, 298 K)


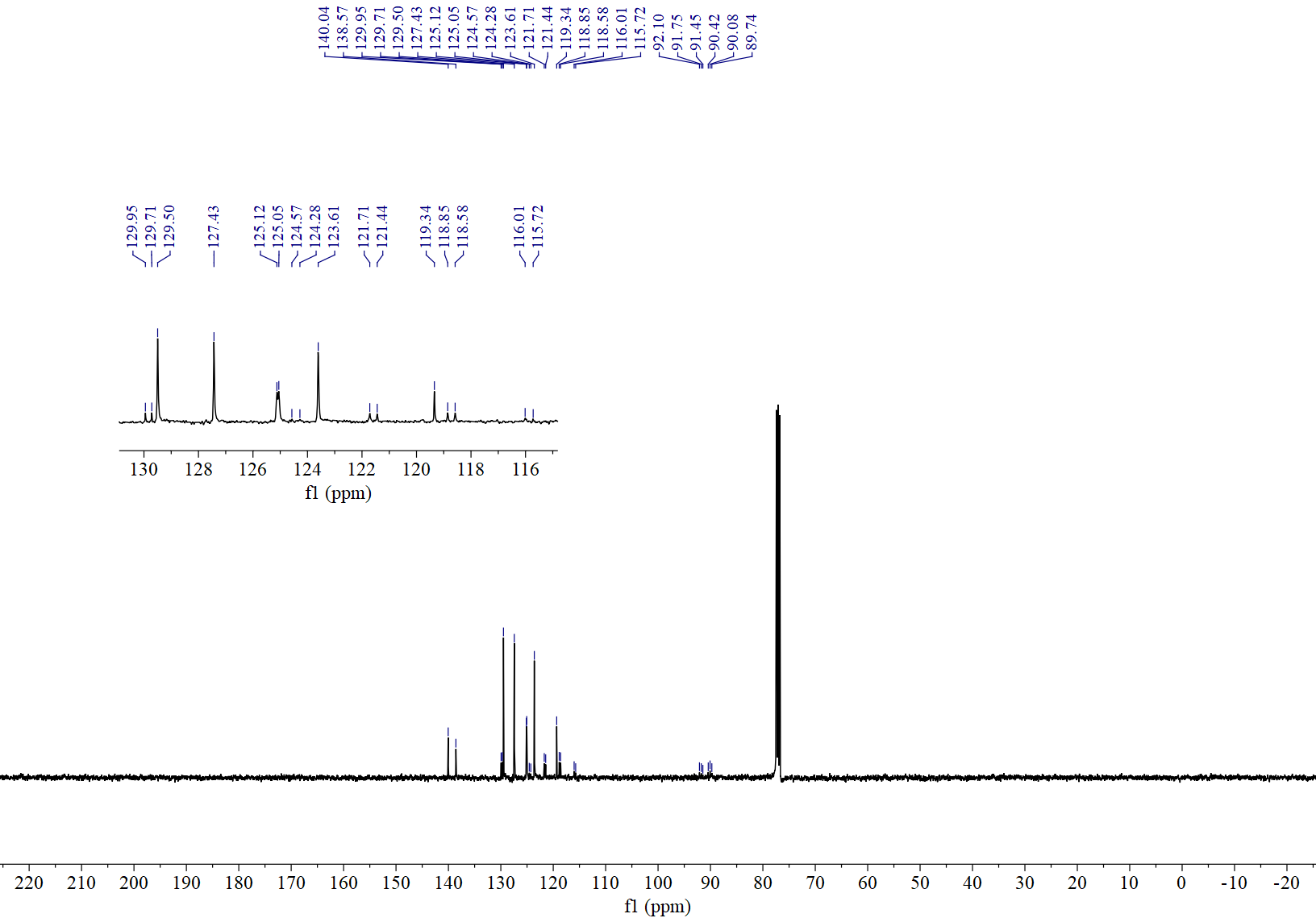

**^13^C NMR** of **29** (101 MHz, Chloroform-*d*, 298 K)


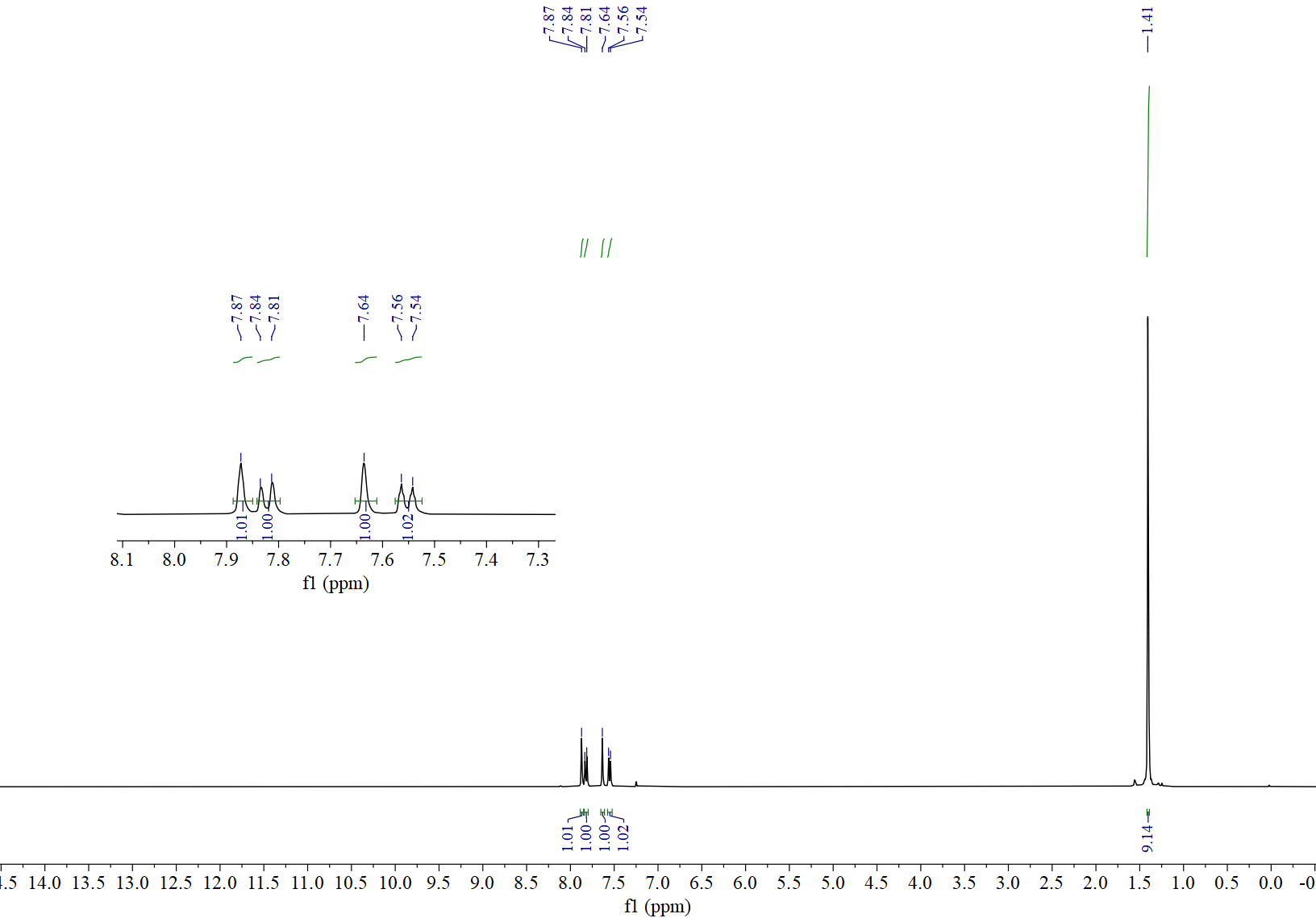

**^1^H NMR** of **30** (400 MHz, Chloroform-*d*, 298 K)


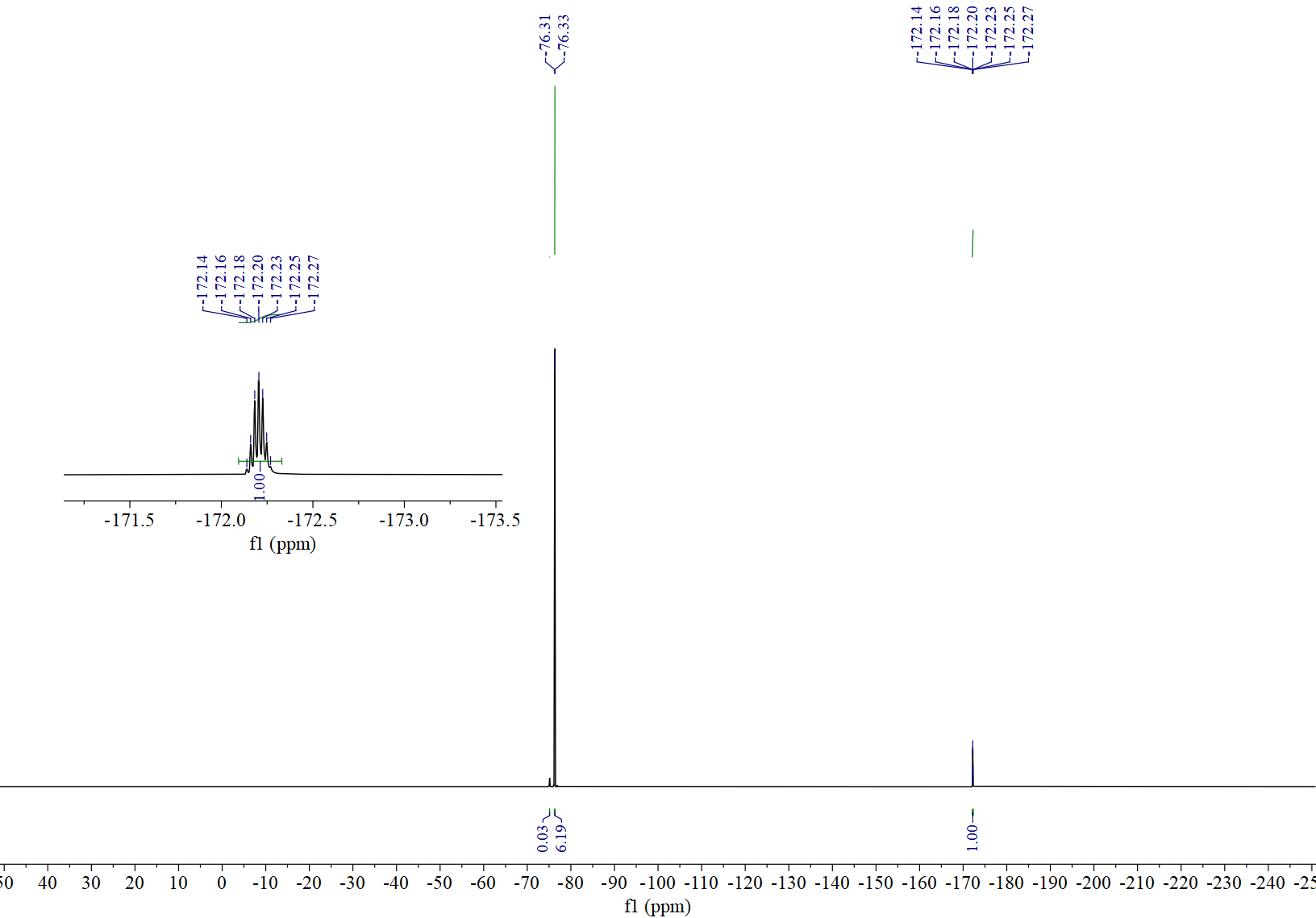

**^19^F NMR** of **30** (376 MHz, Chloroform-*d*, 298 K)


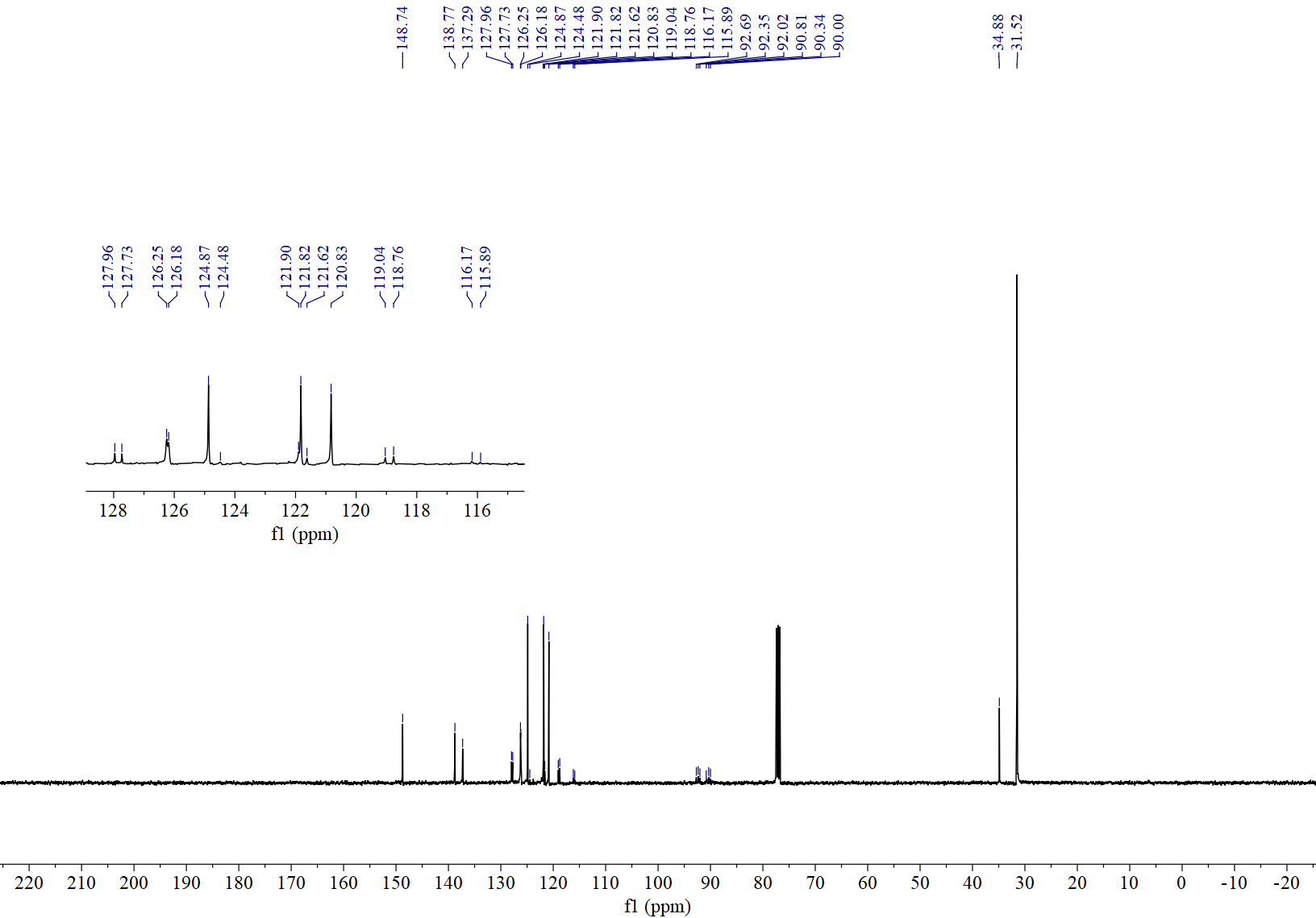

**^13^C NMR** of **30** (101 MHz, Chloroform-*d*, 298 K)


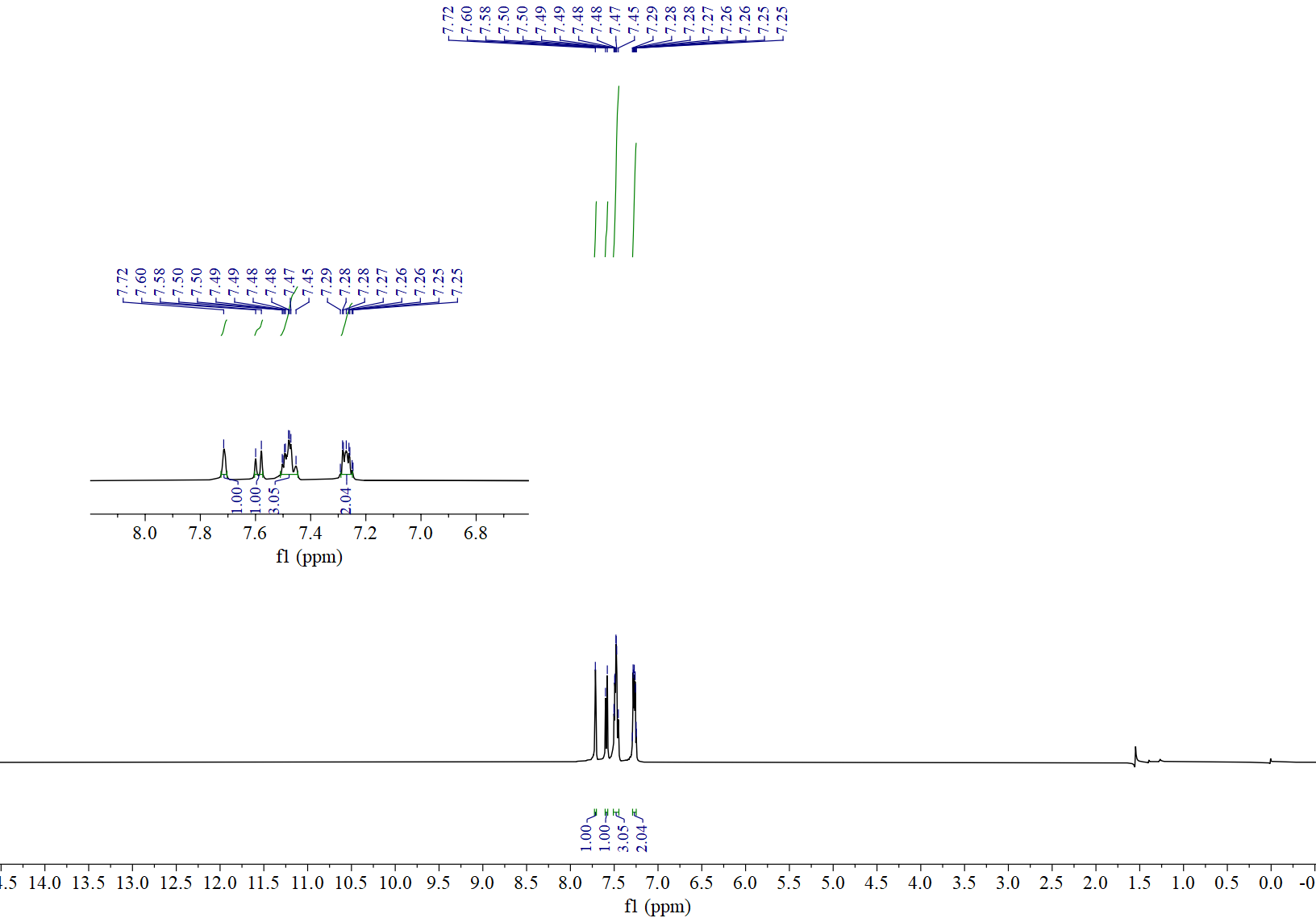

**^1^H NMR** of **31** (400 MHz, Chloroform-*d*, 298 K)


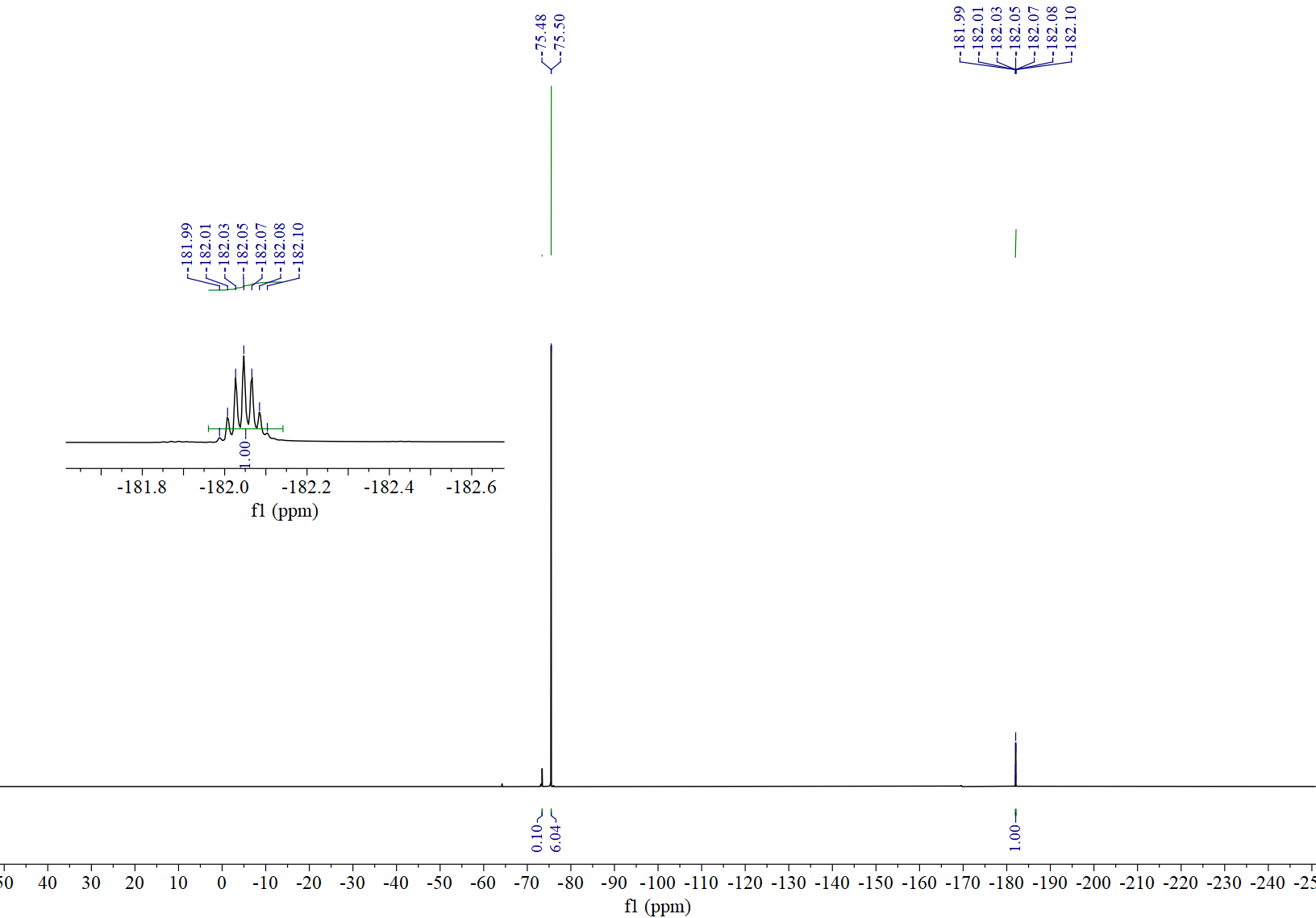

**^19^F NMR** of **31** (376 MHz, Chloroform-*d*, 298 K)


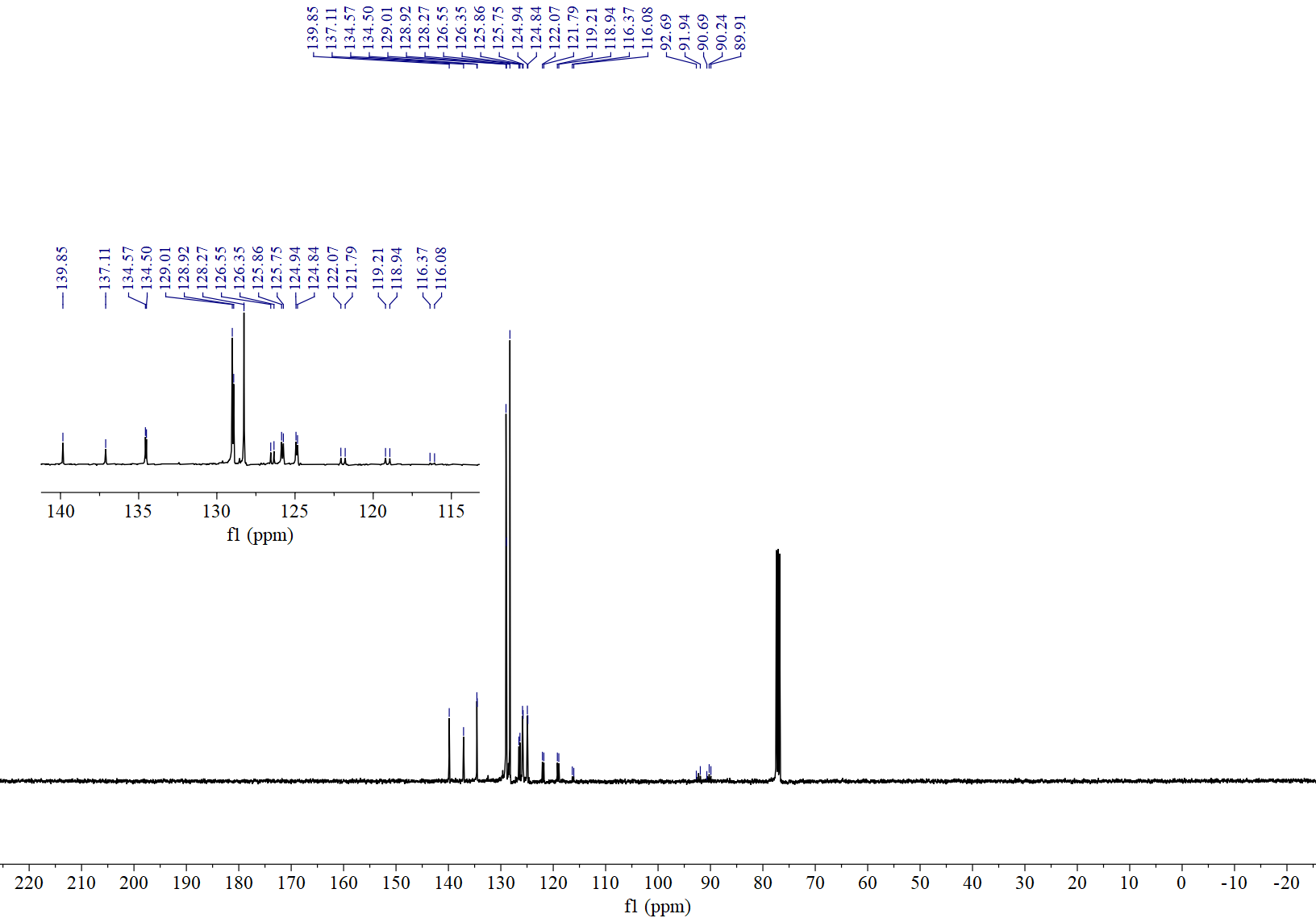

**^13^C NMR** of **31** (101 MHz, Chloroform-*d*, 298 K)


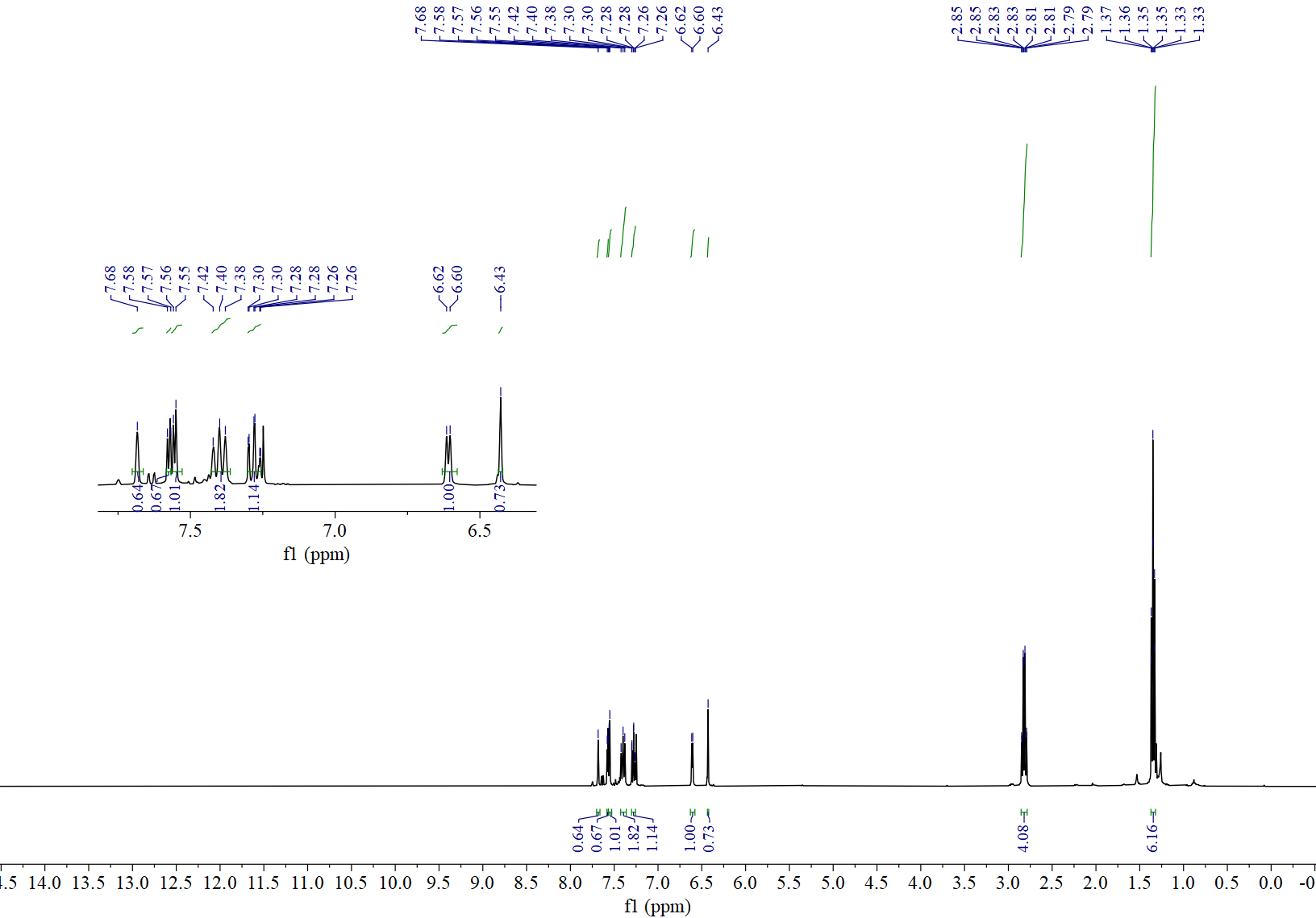

**^1^H NMR** of **32** (400 MHz, Chloroform-*d*, 298 K)


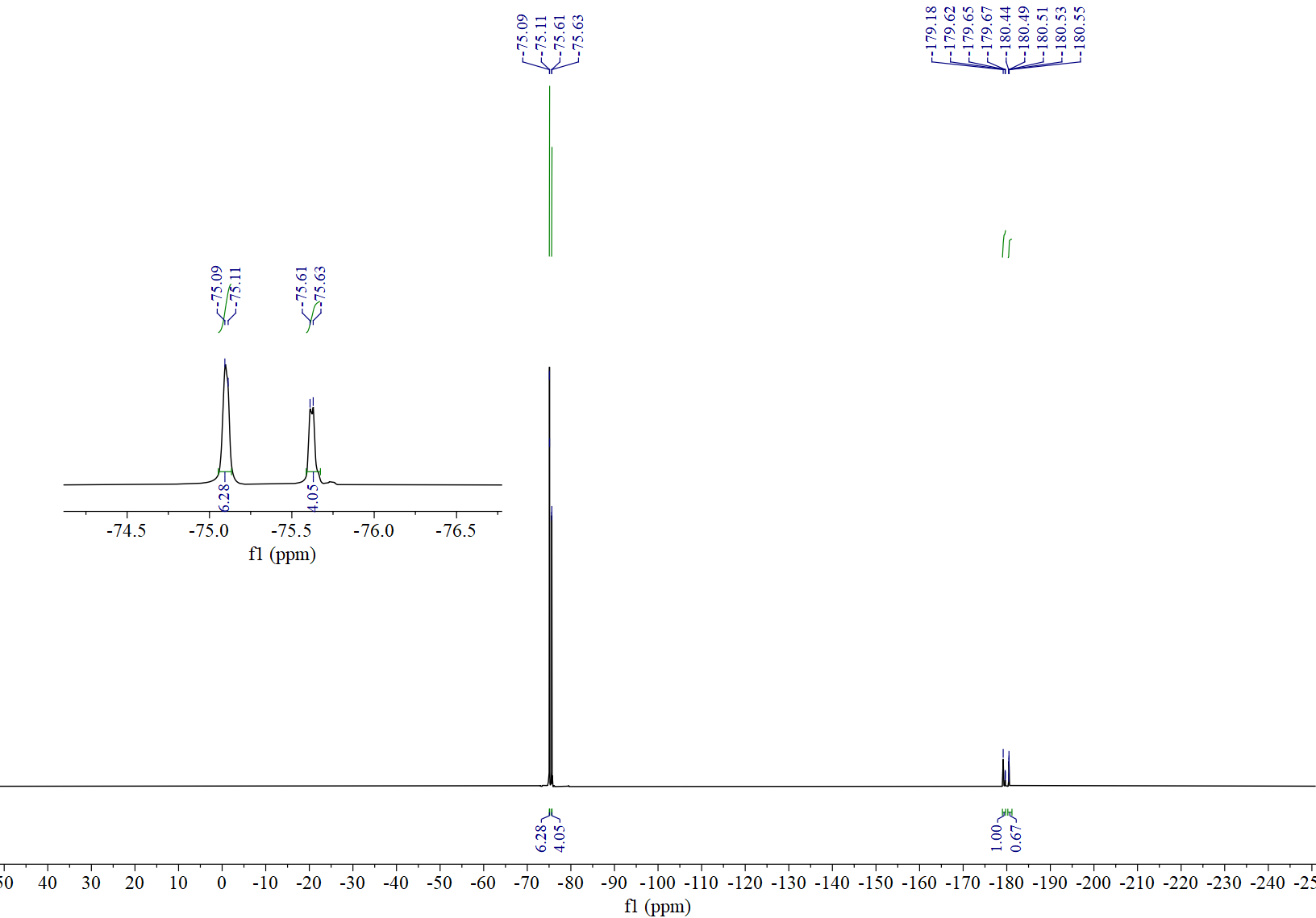

**^19^F NMR** of **32** (376 MHz, Chloroform-*d*, 298 K)


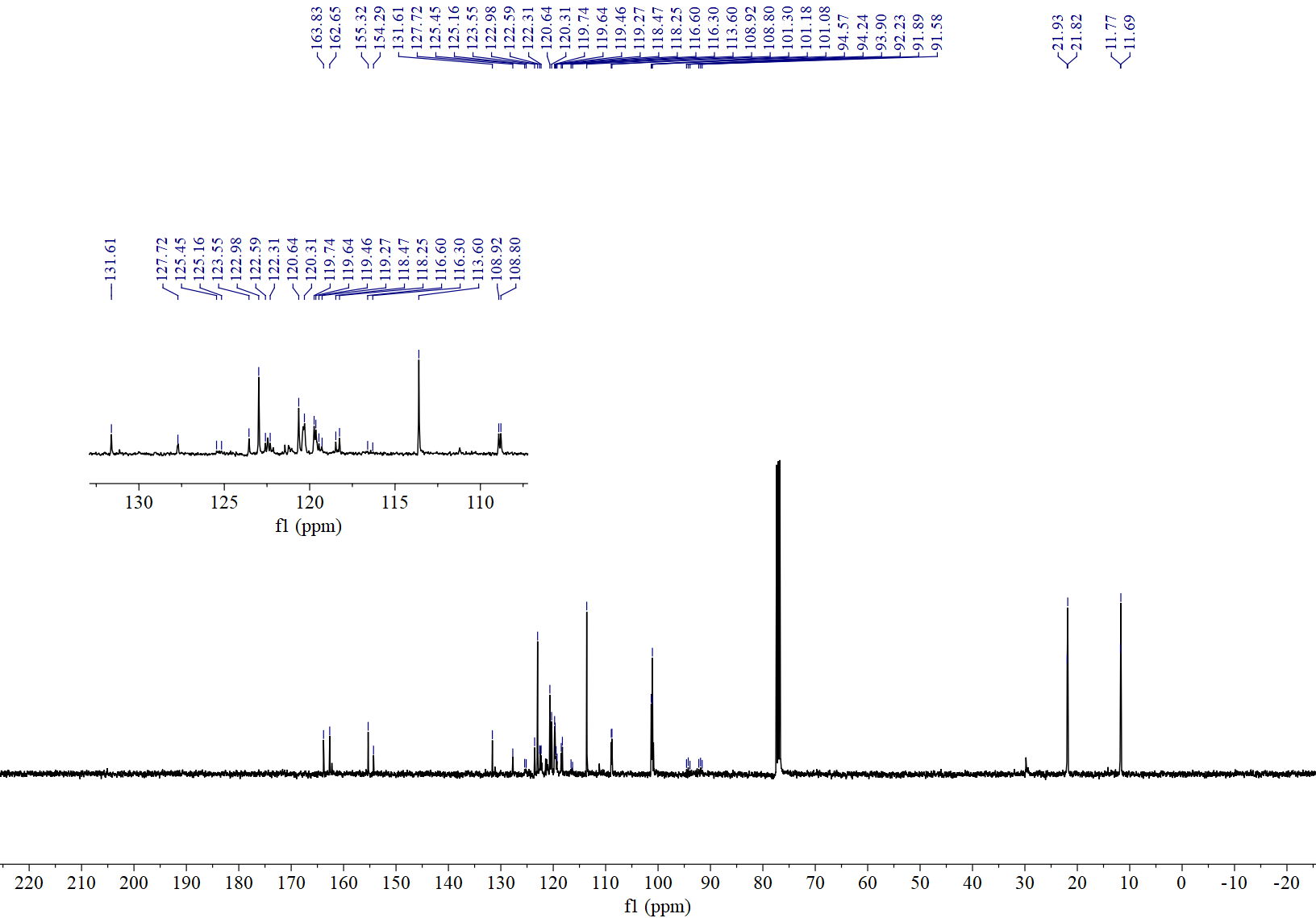

**^13^C NMR** of **32** (101 MHz, Chloroform-*d*, 298 K)


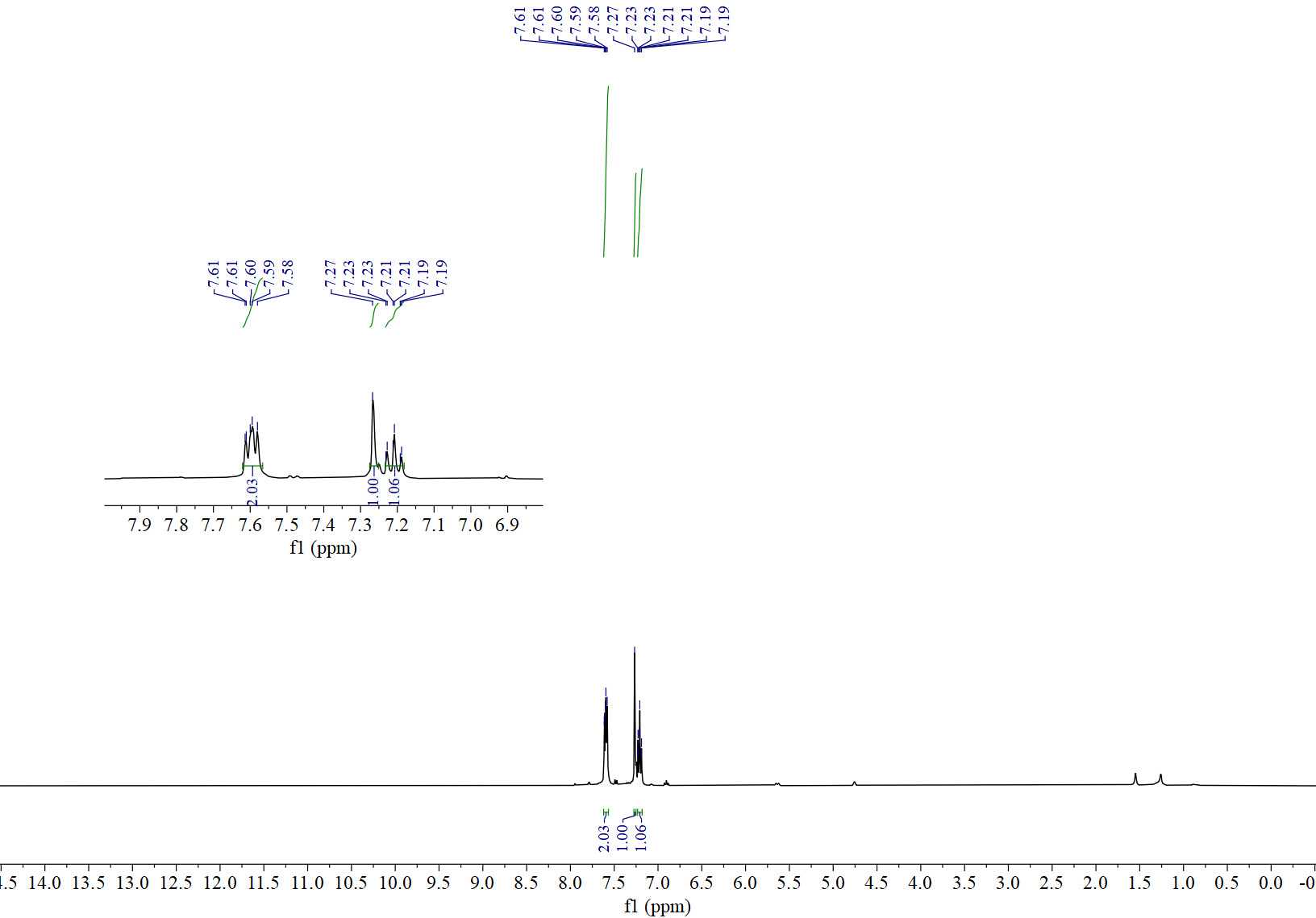

**^1^H NMR** of **33** (400 MHz, Chloroform-*d*, 298 K)


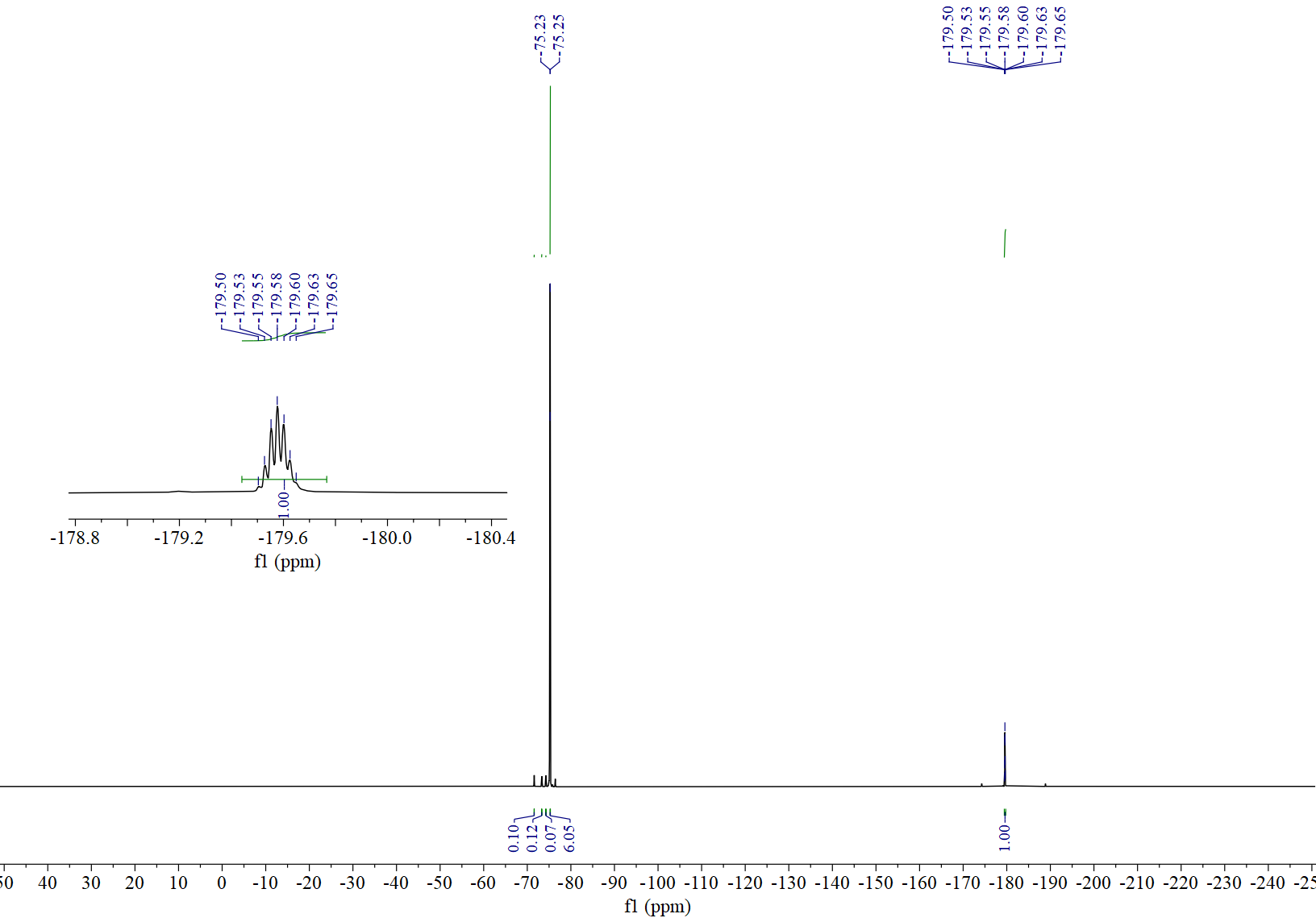


**^19^F NMR** of **33** (376 MHz, Chloroform-*d*, 298 K)

**^13^C NMR** of **33** (101 MHz, Chloroform-*d*, 298 K)

**^1^H NMR** of **34** (400 MHz, Acetonitrile-*d*_3_, 298 K)

**^19^F NMR** of **34** (376 MHz, Acetonitrile-*d*_3_, 298 K)

**^13^C NMR** of **34** (101 MHz, Acetonitrile-*d*_3_, 298 K)

**^1^H NMR** of **35** (400 MHz, Chloroform-*d*, 298 K)

**^19^F NMR** of **35** (376 MHz, Chloroform-*d*, 298 K)

**^13^C NMR** of **35** (101 MHz, Chloroform-*d*, 298 K)

**^1^H NMR** of **36** (400 MHz, Acetonitrile-*d*_3_, 298 K)

**^19^F NMR** of **36** (376 MHz, Acetonitrile-*d*_3_, 298 K)

**^13^C NMR** of **36** (101 MHz, Acetonitrile-*d*_3_, 298 K)

**^1^H NMR** of **37** (400 MHz, Chloroform-*d*, 298 K)

**^19^F NMR** of **37** (376 MHz, Chloroform-*d*, 298 K)

**^13^C NMR** of **37** (101 MHz, Chloroform-*d*, 298 K)

**^1^H NMR** of **38** (400 MHz, Acetonitrile-*d*_3_, 298 K)

**^19^F NMR** of **38** (376 MHz, Acetonitrile-*d*_3_, 298 K)

**^13^C NMR** of **38** (101 MHz, Acetonitrile-*d*_3_, 298 K)

**^1^H NMR** of **45** (400 MHz, Acetonitrile-*d*_3_, 298 K)

**^19^F NMR** of **45** (376 MHz, Acetonitrile-*d*_3_, 298 K)

**^13^C NMR** of **45** (101 MHz, Acetonitrile-*d*_3_, 298 K)

**^1^H NMR** of **46** (400 MHz, Dimethyl sulfoxide-*d_6_*, 298 K)

**^19^F NMR** of **46** (376 MHz, Dimethyl sulfoxide-*d_6_*, 298 K)

**^13^C NMR** of **46** (101 MHz, Dimethyl sulfoxide-*d_6_*, 298 K)

**^1^H NMR** of **47** (400 MHz, Chloroform-*d*, 298 K)

**^19^F NMR** of **47** (376 MHz, Chloroform-*d*, 298 K)

**^13^C NMR** of **47** (101 MHz, Chloroform-*d*, 298 K)

**^1^H NMR** of **48** (400 MHz, Chloroform-*d*, 298 K)

**^19^F NMR** of **48** (376 MHz, Chloroform-*d*, 298 K)

**^13^C NMR** of **48** (101 MHz, Chloroform-*d*, 298 K)

**^1^H NMR** of **49** (400 MHz, Chloroform-*d*, 298 K)

**^19^F NMR** of **49** (376 MHz, Chloroform-*d*, 298 K)

**^13^C NMR** of **49** (101 MHz, Chloroform-*d*, 298 K)

**^1^H NMR** of **50** (400 MHz, Chloroform-*d*, 298 K)

**^19^F NMR** of **50** (376 MHz, Chloroform-*d*, 298 K)

**^13^C NMR** of **50** (101 MHz, Chloroform-*d*, 298 K)

**NOESY NMR** of **50** (400 MHz, Chloroform-*d*, 298 K)

**H^2^**

**H^1^**

**^1^H NMR** of **51** (400 MHz, Chloroform-*d*, 298 K)

**^19^F NMR** of **51** (376 MHz, Chloroform-*d*, 298 K)

**^13^C NMR** of **51** (101 MHz, Chloroform-*d*, 298 K)

**^1^H NMR** of **52** (400 MHz, Chloroform-*d*, 298 K)

**^19^F NMR** of **52** (376 MHz, Chloroform-*d*, 298 K)

**^13^C NMR** of **52** (101 MHz, Chloroform-*d*, 298 K)

**^1^H NMR** of **53** (400 MHz, Chloroform-*d*, 298 K)

**^19^F NMR** of **53** (376 MHz, Chloroform-*d*, 298 K)

**^13^C NMR** of **53** (101 MHz, Chloroform-*d*, 298 K)

**^1^H NMR** of **54** (400 MHz, Chloroform-*d*, 298 K)

**^19^F NMR** of **54** (376 MHz, Chloroform-*d*, 298 K)

**^13^C NMR** of **54** (101 MHz, Chloroform-*d*, 298 K)

**^1^H NMR** of **55** (400 MHz, Chloroform-*d*, 298 K)

**^19^F NMR** of **55** (376 MHz, Chloroform-*d*, 298 K)

**^13^C NMR** of **55** (101 MHz, Chloroform-*d*, 298 K)

**^1^H NMR** of **56** (400 MHz, Chloroform-*d*, 298 K)

**^19^F NMR** of **56** (376 MHz, Chloroform-*d*, 298 K)

**^13^C NMR** of **56** (101 MHz, Chloroform-*d*, 298 K)

**^1^H NMR** of **57** (400 MHz, Chloroform-*d*, 298 K)

**^19^F NMR** of **57** (376 MHz, Chloroform-*d*, 298 K)

**^13^C NMR** of **57** (101 MHz, Chloroform-*d*, 298 K)

**^1^H NMR** of **58** (400 MHz, Chloroform-*d*, 298 K)

**^19^F NMR** of **58** (376 MHz, Chloroform-*d*, 298 K)

**^13^C NMR** of **58** (101 MHz, Chloroform-*d*, 298 K)

**^1^H NMR** of **59** (400 MHz, Chloroform-*d*, 298 K)

**^19^F NMR** of **59** (376 MHz, Chloroform-*d*, 298 K)

**^13^C NMR** of **59** (101 MHz, Chloroform-*d*, 298 K)

**^1^H NMR** of **60** (400 MHz, Chloroform-*d*, 298 K)

**^19^F NMR** of **60** (376 MHz, Chloroform-*d*, 298 K)

**^13^C NMR** of **60** (101 MHz, Chloroform-*d*, 298 K)

**^1^H NMR** of **61** (400 MHz, Chloroform-*d*, 298 K)

**^19^F NMR** of **61** (376 MHz, Chloroform-*d*, 298 K)

**^13^C NMR** of **61** (101 MHz, Chloroform-*d*, 298 K)

**^1^H NMR** of **62** (400 MHz, Chloroform-*d*, 298 K)

**^19^F NMR** of **62** (376 MHz, Chloroform-*d*, 298 K)

**^13^C NMR** of **62** (101 MHz, Chloroform-*d*, 298 K)

**^1^H-^1^H COSY NMR** of **62** (400 MHz, Chloroform-*d*, 298 K)

**H^1^**

**H^2^**

**H^3^**

**H^4^**

**NOESY NMR** of **62** (400 MHz, Chloroform-*d*, 298 K)

**H^3^**

**H^2^**

**^1^H NMR** of **63** (400 MHz, Chloroform-*d*, 298 K)

**^19^F NMR** of **63** (376 MHz, Chloroform-*d*, 298 K)

**^13^C NMR** of **63** (101 MHz, Chloroform-*d*, 298 K)

**^1^H NMR** of **64** (400 MHz, Chloroform-*d*, 298 K)

**^19^F NMR** of **64** (376 MHz, Chloroform-*d*, 298 K)

**^13^C NMR** of **64** (101 MHz, Chloroform-*d*, 298 K)

**^1^H NMR** of **65** (400 MHz, Chloroform-*d*, 298 K)

**^19^F NMR** of **65** (376 MHz, Chloroform-*d*, 298 K)

**^13^C NMR** of **65** (101 MHz, Chloroform-*d*, 298 K)

**^1^H-^1^H COSY NMR** of **65** (400 MHz, Chloroform-*d*, 298 K)

**H^2^**

**H^3^**

**H^1^**

**NOESY NMR** of **65** (400 MHz, Chloroform-*d*, 298 K)

**H^1^**

**H^3^**

**H^2^**

**^1^H NMR** of **66** (101 MHz, Chloroform-*d*, 298 K)

**^19^F NMR** of **66** (376 MHz, Chloroform-*d*, 298 K)

**^13^C NMR** of **66** (101 MHz, Chloroform-*d*, 298 K)

**^1^H NMR** of **67** (400 MHz, Chloroform-*d*, 298 K)

**^19^F NMR** of **67** (376 MHz, Chloroform-*d*, 298 K)

**^13^C NMR** of **67** (101 MHz, Chloroform-*d*, 298 K)

**NOESY NMR** of **67** (400 MHz, Chloroform-*d*, 298 K)

**H^1^**

**H^2^**

**H^3^**

**^1^H NMR** of **68** (400 MHz, Chloroform-*d*, 298 K)

**^19^F NMR** of **68** (376 MHz, Chloroform-*d*, 298 K)

**^13^C NMR** of **68** (101 MHz, Chloroform-*d*, 298 K)
